# Integrated EBV and host DNA methylation profiling identifies distinct candidate EBV–host junction patterns in chronic active EBV infection

**DOI:** 10.1128/spectrum.01881-26

**Published:** 2026-09-25

**Authors:** Ran Wang, Linlin Zhang, Dilara Dilmurat, Meng Zhang, Honghao Ma, Dong Wang, Haijuan Xiao, Jian Ge, Rui Zhang, Gang Liu, Zhengde Xie

**Affiliations:** 1 Laboratory of Infection and Virology, Beijing Pediatric Research Institute, Beijing Children's Hospital, Capital Medical University, National Center for Children's Health 12517, Beijing, China; 2 Beijing Key Laboratory of Core Technologies for the Prevention and Treatment of Emerging Infectious Diseases in Children, Key Laboratory of Major Diseases in Children, Ministry of Education, National Clinical Research Center for Respiratory Diseases, Beijing, China; 3 Research Unit of Critical Infection in Children, 2019RU016, Chinese Academy of Medical Sciences, Beijing, China; 4 Medical Oncology Department, Pediatric Oncology Center, Beijing Children's Hospital, Capital Medical University, National Center for Children's Health 12517, Beijing, China; 5 Department of Pediatric Rehabilitation, Beijing Boai Hospital, School of Rehabilitation Medicine, Capital Medical University, China Rehabilitation Research Center 665503, Beijing, China; 6 Hematology Center, Beijing Children's Hospital, Capital Medical University, National Center for Children's Health 12517, Beijing, China; 7 Department of Infectious Diseases, Beijing Children’s Hospital, Capital Medical University, National Center for Children’s Health 12517, Beijing, China; University of Florida College of Dentistry, Gainesville, Florida, USA

**Keywords:** Epstein–Barr virus (EBV), chronic active EBV infection (CAEBV), DNA methylation, candidate EBV–host junctions

## Abstract

Chronic active Epstein–Barr virus (EBV) infection (CAEBV) is a potentially life-threatening lymphoproliferative disease with limited effective therapies. Host–virus interactions, including epigenetic regulation, may contribute to CAEBV progression. The methylation landscape of EBV and the host genome, and the genomic features of EBV–host junctions in CAEBV remain incompletely characterized. We profiled EBV DNA methylation in peripheral blood mononuclear cells (PBMCs) from patients with CAEBV and infectious mononucleosis (IM) using targeted bisulfite sequencing. Host DNA methylation patterns were assessed by reduced representation bisulfite sequencing in CAEBV, IM, healthy EBV carriers, and EBV-uninfected controls. In parallel, candidate EBV–host junctions were identified by liquid-phase hybridization capture sequencing. Because same-sample junction-specific PCR and Sanger sequencing were not available, the capture-derived junctions were regarded as candidate events. The EBV genome showed global hypermethylation in CAEBV compared with IM, together with distinct latency-associated promoter methylation patterns. CAEBV also exhibited a distinct host methylation signature relative to control groups, with differentially methylated genes enriched in immune-related processes and cancer-associated pathways. Candidate EBV–host junctions were detected more frequently in CAEBV than in IM, with viral-side regions showing recurrent candidate junction detection, including *EBNA-LP*, *EBNA1*, and *BZLF1*. In CAEBV, candidate junctions were enriched in intronic and promoter regions, common fragile sites, and repetitive elements. At the gene level, recurrently targeted host loci included genes implicated in genomic instability and cancer-related pathways. In exploratory longitudinal analyses, allogeneic hematopoietic stem cell transplantation was associated with lower EBV methylation levels and fewer detectable candidate EBV–host junctions, whereas chemotherapy alone was not associated with comparable changes. These data characterize EBV and host methylation landscapes together with candidate EBV–host junction patterns in CAEBV. The findings provide epigenetic and genomic clues to CAEBV pathogenesis and generate hypotheses regarding potential biomarkers of treatment response, but require validation in larger, independent cohorts.

**IMPORTANCE:**

Chronic active Epstein–Barr virus (CAEBV) infection is a rare but severe disease that can progress to life-threatening inflammation or lymphoma, yet its molecular basis remains poorly understood. We found that both the EBV genome and the host genome exhibit distinct DNA methylation patterns in children with CAEBV compared with infectious mononucleosis. We also identified more frequent candidate EBV–host junction signals in CAEBV and observed that these signals became less detectable after successful hematopoietic stem cell transplantation. These findings improve our understanding of host–virus interactions in CAEBV and provide candidate molecular features that may be useful for future studies of disease mechanisms and treatment monitoring.

## INTRODUCTION

Epstein–Barr virus (EBV) is a lymphotropic *Gammaherpesvirinae* virus that infects B lymphocytes and oropharyngeal epithelial cells through salivary transmission (1). EBV has latent and lytic phases and can establish lifelong latency in memory B cells (2). Primary EBV infection usually occurs in childhood and may present as infectious mononucleosis (IM), characterized by fever, pharyngitis, and lymphadenopathy (3). Although IM is generally self-limiting and controlled by vigorous immune responses, a minority of individuals develop chronic or recurrent IM-like symptoms, known as chronic active EBV infection (CAEBV). CAEBV is classified as a lymphoproliferative disease involving T and NK cell lineages (4), and proliferation of EBV-infected T cells is commonly observed in East Asian patients (5). CAEBV is often associated with high peripheral blood EBV-DNA loads (>10^2.5^ copies/µg DNA) and severe complications, including hemophagocytic lymphohistiocytosis and lymphoma, with a mortality rate of approximately 30% (6).

The pathogenesis of CAEBV remains unclear, and allogeneic hematopoietic stem cell transplantation (allo-HSCT) is currently considered the only curative treatment for CAEBV. Therefore, investigating the mechanisms underlying its pathological features is important for understanding CAEBV pathogenesis.

Epigenetic modifications, especially DNA methylation, play important roles in EBV-associated tumors, including nasopharyngeal carcinoma, EBV-associated gastric carcinoma, and EBV-associated lymphoma (7). EBV-encoded latent membrane protein 1 and LMP2A can interact with host DNA methyltransferases to promote tumor metastasis through hypermethylation of tumor suppressor genes and hypomethylation of oncogenes (8–10). Patients with CAEBV have a marked predisposition to T-cell leukemia or lymphoma, suggesting possible oncogenic features such as those of EBV-related tumors (2, 11). Thus, the involvement of DNA methylation in CAEBV pathogenesis warrants further investigation.

Virus–host integration has been proposed to explain aberrant DNA methylation of the human papillomavirus genome and is also considered an important oncogenic mechanism (12, 13). EBV has been reported to integrate near tumor suppressor genes and promote tumorigenesis in nasopharyngeal carcinoma, EBV-associated gastric carcinoma, and NK/T-cell lymphoma (14, 15), but whether integration plays a similar role in CAEBV remains unknown. In addition, hypermethylation-related gene silencing is reversible and can be affected by demethylating agents. DNA hypermethylation of host genes is associated with adult T-cell leukemia/lymphoma, and the demethylating drug decitabine has shown anti-ATL activity (16), suggesting potential therapeutic relevance in diseases with aberrant DNA methylation. Therefore, defining EBV and host DNA methylation profiles and characterizing capture-derived candidate EBV–host junctions may provide further clues to CAEBV pathogenesis.

In this study, we performed EBV and host genome DNA methylation sequencing, together with capture-based analysis of candidate EBV–host junctions, in patients with CAEBV, using IM patients, healthy EBV carriers, and EBV-uninfected children as comparators. We characterized distinct EBV and host methylation profiles, compared host differentially methylated genes across groups, and further characterized the genomic distribution of capture-derived candidate EBV–host junctions in CAEBV. These findings provide an integrated view of EBV and host methylation patterns and genomic junction features in CAEBV, offering epigenetic and genomic clues to its pathogenesis.

## MATERIALS AND METHODS

### Study subjects

Peripheral blood mononuclear cells (PBMCs) were collected from 74 children who visited Beijing Children’s Hospital between January 2019 and May 2021, including 32 patients with CAEBV, 24 patients with IM, nine healthy EBV carriers (HCs), and nine EBV-uninfected controls. The demographic and clinical characteristics are summarized in Table S1. All included cases were confirmed according to the corresponding diagnostic criteria in the expert consensus (17). The EBV-infected cell lineage was determined by pathological examination of lymph-node biopsy specimens (18). Samples used for different sequencing platforms were randomly allocated from the available PBMC samples that met the corresponding minimum input quantity and quality requirements, without selection based on disease severity, treatment status, or other clinical characteristics. Owing to limitations in the blood volume that could be collected from pediatric patients, independent patient cohorts were used for reduced representation bisulfite sequencing (RRBS) and RNA-seq.

### EBV serology and DNA load

The EBV serology profile was measured using the commercial EBV test kit from EUROIMMUN Corporation (Lubeck). EBV DNA load was measured using a PCR-based commercial kit (DAAN Gene). The standard protocols from the manufacturers were followed for these assays.

### EBV genome DNA methylation profiling by targeted bisulfite sequencing (TBS) and data processing

Genomic DNA of PBMC samples was extracted using the Magnetic Universal Genomic DNA Kit (TIANGEN), and 1 μg of genomic DNA was converted using the ZYMO EZ DNA Methylation-Gold Kit (Zymo Research) (19). Barcoded libraries from all samples were sequenced using the Illumina platform (20). The samples used for TBS included 24 patients with CAEBV, 17 patients with IM, and nine HCs. For each sample, 1 μg of total genomic DNA extracted from PBMCs was used as the starting input. The EBV DNA loads listed in Table S1 represent clinical measurements obtained from plasma and whole blood. At the current sequencing depth, the HC samples did not yield sufficient EBV-derived reads for reliable viral methylation analysis.

Sequencing adapters and low-quality sequencing data were removed by Trimmomatic (version 0.36). The methylation level of the CpG site was calculated using the Python program for calculating methylation with BSMAP software. The calculation principle is methylation densities = C-reads/(C-reads + T-reads), where *C-reads* is the number of methylation-supporting reads covering the site (reads with a C at the site) and *T-reads* is the number of reads that do not support methylation covering the site (reads with a T at the site). Finally, according to the methylation densities calculated above, R was used for statistical analysis and visualization, and the two-tailed *t*-test was used to analyze the differences between groups. A difference was considered significant when the *P* value was < 0.05 and the absolute value of the difference in mean methylation between groups was greater than 0.1. The gene-region heatmap was generated based on probe coverage and the distribution of valid reads. Both LMP-2B and EBNA3A span three independently covered regions and are therefore labeled as LMP-2B (1/2/3) and EBNA3A (1/2/3), respectively, representing different sequenced fragments of the same gene.

### DNA methylation profiling by RRBS and data processing

Genomic DNA of PBMC samples from 24 CAEBV (CAEBV 1–24), 17 IM (IM 1–17), nine HC (HC 1–9), and nine EBV-uninfected children (uninfected 1–9) was extracted using DNeasy Blood and Tissue Kit (Qiagen), and 200 ng–1 μg of DNA was subjected to RRBS library preparation for genome-wide DNA methylation profiling. The FastQC tool was used to perform basic statistics on the quality of the raw reads. Sequencing adapters and low-quality sequencing data were removed using Trimmomatic (version 0.36) (21).

BSMAP software was used to map the bisulfite sequence to the reference genome with default parameters (22). The statistical information of the alignment was collected; only uniquely mapped reads were retained for the following analysis, and only cytosines covered by at least five reads were included in the analysis. If the base on the alignment is C, methylation occurs. If the base on the alignment is T, no methylation occurs. The methylation levels of individual cytosines were calculated as the ratio of the sequencing depth of the ascertained methylated CpG cytosines to the total sequencing depth of individual CpG cytosines [ML = mC/(mC + umC)], where *ML* is the methylation level and *mC* and *umC* represent the number of reads supporting methylated C and the number of reads supporting unmethylated C, respectively.

Differentially methylated regions (DMRs) were identified using Metilene (ver. 0.2-7). A DMR was required to contain at least five cytosine sites, with <200 bp between adjacent sites. CG-DMR candidate regions were required to have average methylation level differences of >0.1 between the corresponding populations (23). Finally, regions with a 2D KS-test *P* value < 0.05 and BH (Benjamini and Hochberg)-corrected *P* value < 0.05 were determined to be final DMRs. Promoter methylation was calculated as the averaged DNA methylation values based on GENCODE promoter regions (i.e., 1 kb upstream to 500 bp downstream of the annotated TSSs).

### Gene expression analysis through RNA sequencing (RNA-Seq)

Global gene expression profiling of RNA extracted from eight CAEBV (CAEBV 25–32) and seven IM (IM 18–24) samples was performed using SMART-Seq HT Kit. Paired-end libraries were synthesized by using TruSeq RNA Sample Preparation Kit (Illumina) following TruSeq RNA Sample Preparation Guide. Purified libraries were quantified by Qubit 2.0 Fluorometer (Life Technologies). Library construction and sequencing were performed at Shanghai Biotechnology Corporation. HISAT2 was used (version: 2.0.4) to map the cleaned reads to the human hg38 reference genome with two mismatches (24). *P* values were adjusted for multiple testing to control the false discovery rate (FDR) (25). Fold changes (FCs) were estimated from FPKM values. Differentially expressed genes were selected using an FDR-adjusted *P* value < 0.05 and |log_2_FC| > 1. The RNA-seq and RRBS cohorts did not overlap; therefore, the integrative analysis only compared the direction of group-level changes in CAEBV relative to IM.

### Cluster analysis

Unsupervised hierarchical clustering analysis was performed using R v3.6.3 with the ComplexHeatmap package. The Euclidean distance was used as the distance function both for samples and genes.

### Comparison of methylation profiles of the host genome among different groups

DMRs were screened using thresholds for *P* value, standard deviation, and methylation difference. Principal component analysis and unsupervised hierarchical clustering were performed to assess global methylation patterns across groups. DMRs were annotated to differentially methylated genes (DMGs), with *P* < 0.05 and methylation level difference >0.1 defined as cutoff criteria (23, 26).

### Pathway analysis

Gene Ontology (GO) enrichment analysis of the methylation profile was applied to uncover biological processes of interest. Kyoto Encyclopedia of Genes and Genomes (KEGG) enrichment analysis was used to identify biological pathways enriched among DMR-related genes (27). A *P* value < 0.05 was considered to indicate significant enrichment with DMR-related genes.

### DNA liquid phase hybridization capture sequencing

Genomic DNA was fragmented by sonication to generate fragments predominantly ranging from 180 to 220 bp. The library was constructed using QuarPrep Ultra DNA Library Kit (Dynasty Gene). DNA was captured with a predesigned EBV probe. Qualified libraries were sequenced on the Illumina NovaSeq 6000 sequencing platform, and the sequencing strategy was 150 bp paired-end sequencing. Adaptor sequences and low-quality reads were removed. High-quality reads were aligned to the human (hg19) and EBV (NC_007605.1) genomes using Burrows–Wheeler Aligner (BWA) (28). Alignments were converted from a sequence alignment map format to sorted and indexed binary alignment map (BAM) files (29).

Candidate EBV–host junction-supporting signals, in which one read mapped to the EBV genome and the paired read mapped to the human genome, were extracted. Signals supported by ≥1 read pair were retained as candidate events for exploratory analysis. The HumCFS database was used to annotate candidate junction coordinates within fragile regions (30), and the UCSC Table Browser was used for genomic annotation (31). Representative junction sequences were extracted from capture-derived split reads and aligned to the human and EBV reference genomes for visualization. Throughout this manuscript, these capture-derived signals are therefore referred to as candidate EBV–host junctions.

### Statistical analysis

Two-tailed Student’s *t*-test was used to compare the number of candidate junction signals between groups. The Mann–Whitney *U* test was used to compare continuous variables between groups when data were not normally distributed. All statistical analyses were performed with standard R packages. A *P* value < 0.05 was considered statistically significant.

## RESULTS

### Clinical characteristics of patients with CAEBV

The clinical characteristics of patients with CAEBV are shown in Table 1, and the clinical information of the four groups of participants is summarized in Table S1. The mean age at CAEBV onset was 7.81 years (range, 1–14 years), and the male-to-female ratio was 2.2:1. Thirty patients had T-cell CAEBV, accounting for 93.8% of cases, and two had NK/T-cell CAEBV. Sixteen patients (50.0%) had hemophagocytic lymphohistiocytosis (HLH), and no deaths occurred during follow-up. In the CAEBV group, EBV DNA loads were measured in both plasma and whole blood, with most samples showing relatively high whole-blood EBV DNA levels (Table S1). In the IM group, EBV DNA was measured only in plasma; whole-blood EBV DNA testing was not performed. The mean age at IM onset was 5.63 years (range, 1–10 years), with a male-to-female ratio of 1.7:1. EBV DNA was detected in the plasma of all IM patients (Table S1).

**TABLE 1:** Clinical characteristics of patients with CAEBV^*a*^

	Sex	Age at onset, y	Cell type of CAEBV	Complication
CAEBV 1	M	7	T-cell	HLH
CAEBV 2	M	2	T-cell	HLH
CAEBV 3	M	7	T-cell	HLH
CAEBV 4	M	14	T-cell	–
CAEBV 5	M	5	T-cell	–
CAEBV 6	F	10	NK/T-cell	HLH
CAEBV 7	F	10	T-cell	–
CAEBV 8	M	7	T-cell	–
CAEBV 9	F	11	T-cell	–
CAEBV 10	M	13	T-cell	–
CAEBV 11	M	12	T-cell	HLH
CAEBV 12	M	8	T-cell	HLH
CAEBV 13	M	9	T-cell	–
CAEBV 14	F	7	T-cell	HLH
CAEBV 15	M	8	T-cell	HLH
CAEBV 16	F	9	T-cell	HLH
CAEBV 17	F	7	T-cell	–
CAEBV 18	M	2	T-cell	–
CAEBV 19	F	6	T-cell	HLH
CAEBV 20	M	12	T-cell	HLH
CAEBV 21	M	8	T-cell	–
CAEBV 22	M	1	T-cell	HLH
CAEBV 23	M	5	T-cell	HLH
CAEBV 24	M	5	T-cell	–
CAEBV 25	M	10	NK/T-cell	HLH
CAEBV 26	F	7	T-cell	–
CAEBV 27	M	11	T-cell	HLH
CAEBV 28	F	4	T-cell	HLH
CAEBV 29	M	11	T-cell	–
CAEBV 30	M	6	T-cell	–
CAEBV 31	M	11	T-cell	–
CAEBV 32	F	5	T-cell	–

^*a*^ CAEBV, chronic active Epstein–Barr virus infection; M, male; F, female; HLH, hemophagocytic lymphohistiocytosis; –, none.

### DNA methylation patterns of EBV and the host genome in CAEBV

#### The EBV genome exhibited global hypermethylation in CAEBV

**Fig 1:**
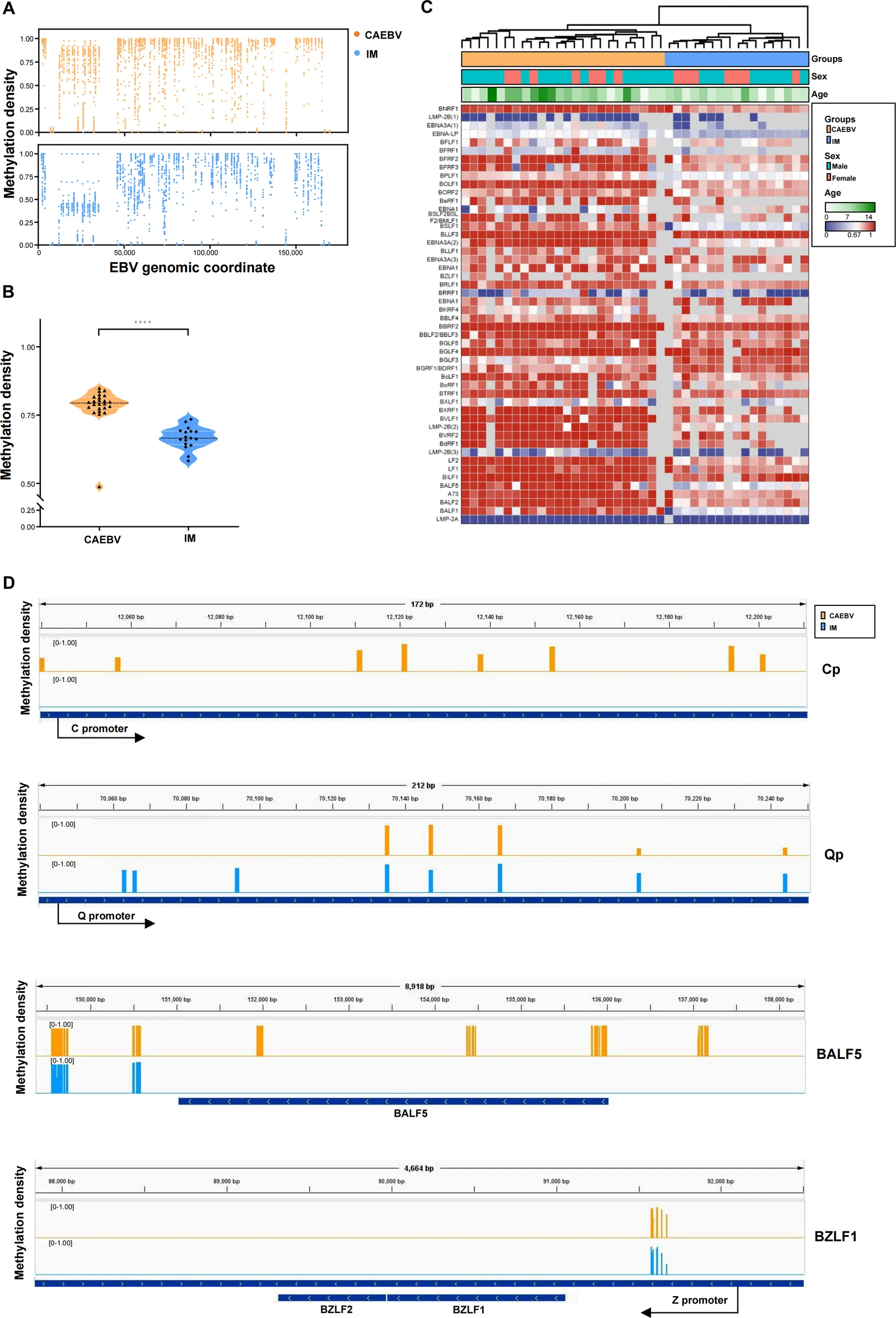
DNA methylation of EBV genomes in cases of CAEBV and IM. (**A**) DNA methylation densities of CpG loci across the EBV genome from the pooled sequencing data of the 24 cases of CAEBV and the pooled data of the 17 cases of IM. Each dot (orange and blue) shows the methylation density at a corresponding CpG site. (**B**) EBV DNA methylation densities of CAEBV and IM patients. Each point represents the mean level of methylation of a patient. ^****^P < 0.0001. (**C**) Unsupervised hierarchical clustering analysis of the EBV DNA methylome for the 41 samples from patients with different EBV-associated diseases. Each vertical bar represents one sample. Each horizontal bar represents the mean methylation density of each EBV coding gene in the viral genome across all 41 samples. The corresponding methylation density of each region for all samples is represented in different colors. Samples from patients with the same EBV-associated disease clustered together. (**D**) DNA methylation of the latent promoters Cp and Qp and lytic genes BZLF1 and BALF5 of EBV. Each bar represents a CpG dinucleotide plotted versus its genome coordinate. The methylation density is displayed on the left y-axis. For *LMP-2B* and *EBNA3A*, which span multiple probe-covered regions, (1), (2), and (3) in the heatmap indicate different analyzed fragments of the same gene. In the color scale, red indicates higher methylation and blue indicates lower methylation.

We profiled EBV genome-wide methylation in CAEBV and IM samples. The mean EBV methylation level was higher in CAEBV than in IM (78.4% vs 66.7%, *P* < 0.0001; Fig. 1A and B). At the current TBS sequencing depth, the PBMC samples from HCs did not yield sufficient EBV-derived reads for reliable EBV methylation analysis, although this does not demonstrate the absence of EBV DNA in PBMCs. Unsupervised hierarchical clustering showed distinct EBV methylation profiles between CAEBV and IM, with samples from the same disease group clustering together (Fig. 1C).

We further analyzed methylation of the *BamHI W* promoter (Wp), *BamHI C* promoter (Cp), *BamHI Q* promoter (Qp), and annotated viral coding regions. In CAEBV samples, Qp, which regulates EBNA1 expression, was hypomethylated, whereas Cp and Wp were hypermethylated (Fig. 1D). The coding regions of *LMP-2A* and *LMP-2B* showed only low levels of methylation (Fig. 1C; Fig. S1A). This profile was consistent with a “Cp off” type II latency pattern in CAEBV. Among lytic genes, little methylation was detected in the immediate early gene *BZLF1*, whereas other lytic genes, including *BcLF1*, *BcRF1*, *BALF5*, and *BLLF1*, showed higher methylation. In IM samples, Cp, the *LMP* promoter (LMPp), and several lytic gene regions, including *BZLF1*, *BALF5*, and *BcRF1*, were predominantly hypomethylated, consistent with a Cp-on latency III pattern. The distributions of Cp-, Qp-, *BALF5*-, and *BZLF1*-associated sites are shown in Fig. 1D, whereas LMPp and the other lytic gene regions are shown in Fig. 1C and Fig. S1A. PCA comparing CAEBV patients with and without HLH showed a clear clustering pattern (Fig. S1B).

#### Different DNA methylation patterns of the host genome between the CAEBV and control groups

**Fig 2:**
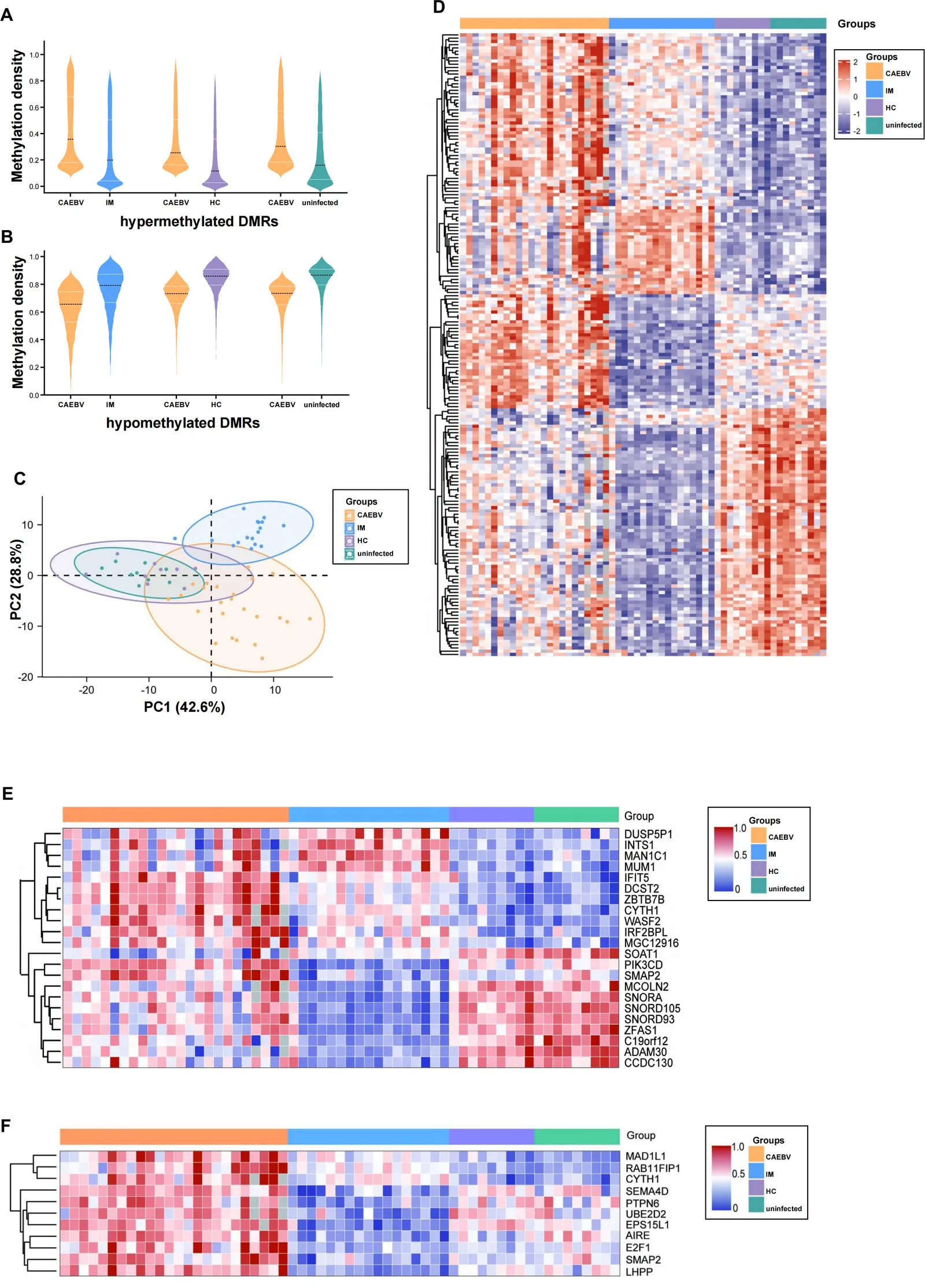
DNA methylation patterns of the host genome in CAEBV. (**A**) Violin plots showing differentially hypermethylated CpG regions for the CAEBV, IM, HC, and uninfected groups for comparison. (**B**) Violin plots showing the differentially hypomethylated CpG regions for the CAEBV, IM, HC, and uninfected groups for comparison. (**C**) PCA plot of DMRs in the four groups. (**D**) Hierarchical clustering with Euclidean distance based on methylation levels of the 191 most variable DMRs across samples from patients with CAEBV or IM, healthy EBV carriers, and EBV-uninfected controls. Each row represents a DMR, and each column represents a sample. Colors represent methylation levels standardized as z scores across samples for each DMR. (**E**) Heatmap of the promoter methylation pattern. (**F**) Heatmap of representative genes.

We next examined host genome methylation in CAEBV, IM, HC, and EBV-uninfected individuals. DMRs were identified between CAEBV and each control group (Fig. 2A and B), and the numbers of DMRs are shown in Table S2. PCA based on co-annotated DMRs separated CAEBV from IM, whereas HC and EBV-uninfected samples clustered together (Fig. 2C). Unsupervised clustering of the 191 most variable DMRs further showed that CAEBV methylation profiles differed from the other three groups, while HC and uninfected samples remained similar (Fig. 2D). CAEBV patients with or without HLH clustered into the same subgroup (Fig. S1C).

#### Functional analysis of differentially methylated genes

GO and KEGG enrichment analyses were performed for annotated DMGs. GO analysis showed enrichment in biological processes including somatic diversification of T-cell receptor genes, myeloid cell development, and immune system processes, as well as in the molecular function of protein binding (Fig. S2A). KEGG analysis indicated enrichment in infectious disease, cancer-related, and endocytosis pathways (Fig. S2B and C).

Because promoter CpG methylation may affect gene expression, we further analyzed promoter-associated DMGs (Fig. 2E). These genes were enriched in the regulation of NK/T-cell proliferation and activation and cholesterol O-acyltransferase activity (Fig. S2D). Representative oncogenes and myeloid differentiation-related genes, including *E2F1*, *PTPN6*, and *AIRE,* were hypermethylated at intronic CpG sites in CAEBV samples (Fig. 2F).

**Fig 3:**
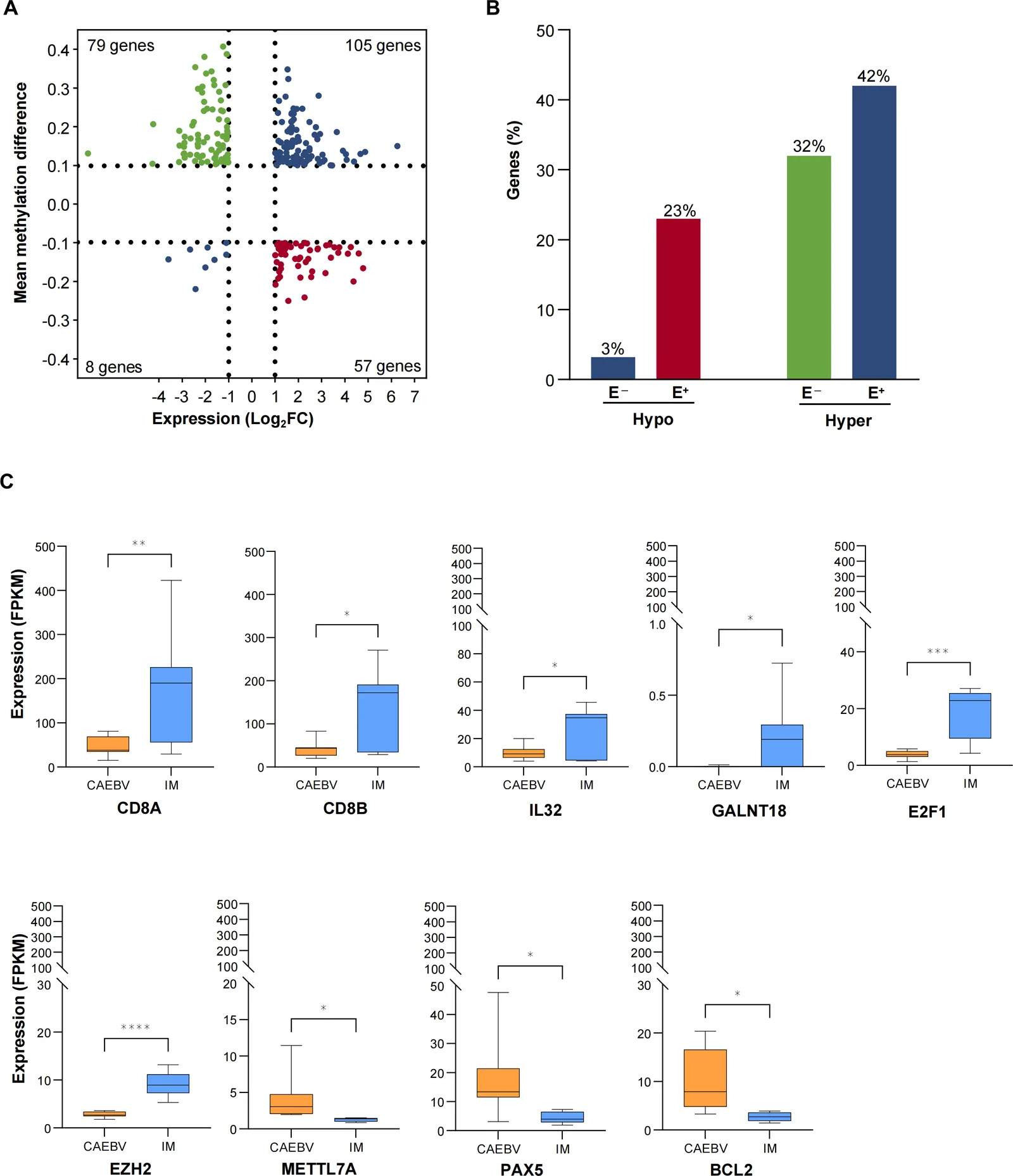
Group-level relationships between DNA methylation and gene expression in CAEBV and IM. (**A** and **B**) A nine-quadrant plot and a bar chart showing the comparison between the DNA methylome and transcriptome. Genes with differences in both DNA methylation levels (|mean methylation difference| > 0.1) and expression levels (|log_2_FC| > 1) were included in the comparison. In CAEBV relative to IM, 136 genes showed inverse group-level changes: 79 were hypermethylated and downregulated (green), and 57 were hypomethylated and upregulated (red). The remaining genes showed concordant changes, comprising 105 hypermethylated and upregulated genes and eight hypomethylated and downregulated genes (both shown in blue). (**C**) Normalized gene expression levels (FPKM) and statistics of representative genes in samples from CAEBV (*n* = 8) and IM (*n* = 7). ^*^P < 0.05; ^**^P < 0.01; ^***^P < 0.001; ^****^P < 0.0001. RRBS and RNA-seq were performed in different patient cohorts; therefore, the nine-quadrant cross-omics comparison represents only the directions of group-level differences in CAEBV relative to IM.

To assess the relationship between methylation and gene expression, RNA-seq and supervised analyses were performed in CAEBV and IM samples. RNA-seq identified 249 significantly differentially expressed genes between CAEBV and IM (Table S3). KEGG analysis highlighted hematopoietic cell lineage, B-cell receptor signaling, and pathways in cancer (Fig. S2E). Among differentially methylated and expressed genes, 136 showed inverse changes in methylation and expression at the group level, including 79 hypermethylated and downregulated genes and 57 hypomethylated and upregulated genes (Fig. 3A and B). Immunomodulatory genes *CD8A*, *CD8B*, and *IL32*, as well as cancer-related genes *GALNT18*, *E2F1*, and *EZH2*, were hypermethylated and expressed at lower levels in CAEBV than in IM; by contrast, the B-cell differentiation-related *PAX5* and methyltransferase *METTL7A* were hypomethylated and more highly expressed in CAEBV (Fig. 3C). Because RRBS and RNA-seq were performed using samples from different patients, these findings represent a group-level comparison of the directions of methylation and expression differences. Differences in genes such as *CD8A* and *CD8B* may also partly reflect variation in the cellular composition of bulk PBMCs resulting from the marked expansion of CD8^+^ T cells in IM.

### Candidate EBV–host junctions

#### Differences in detectable candidate EBV–host junctions between CAEBV and IM

**Fig 4:**
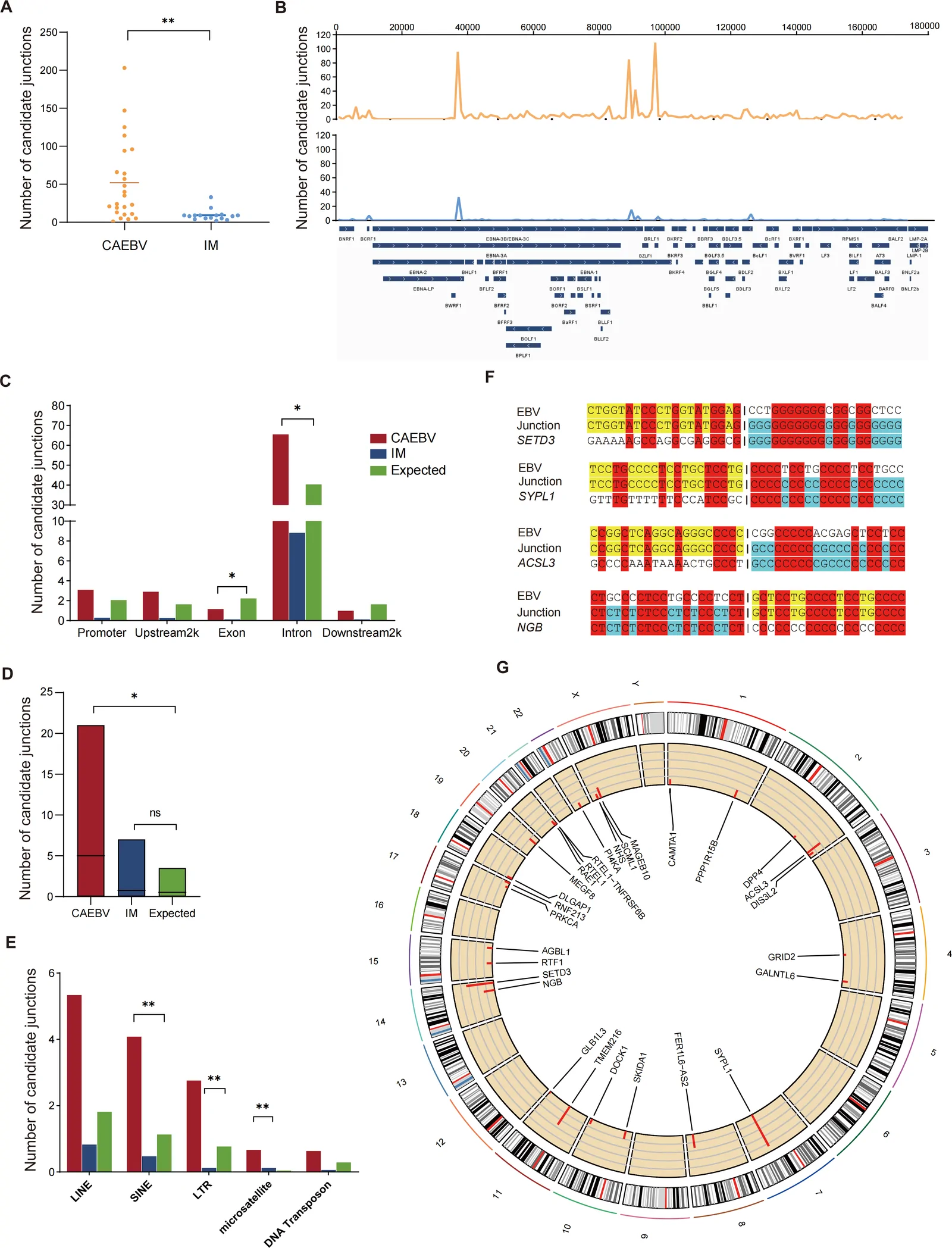
Distribution of capture-derived candidate EBV–host junctions across the EBV and human genomes. (**A**) Dot plot showing the number of candidate EBV–host junctions per sample in CAEBV and IM. (**B**) Distribution plot of junction signals across the EBV genome. The number of junction signals in each 1,000-bp interval is shown as a line plot. (**C**) Numbers of candidate EBV–host junctions in promoter, 2-kb upstream, exonic, intronic, and 2-kb downstream regions of the human genome. (**D**) Numbers of candidate EBV–host junctions within common fragile sites. (**E**) Numbers of candidate EBV–host junctions within repetitive elements, including LINEs, SINEs, LTRs, microsatellite repeats, and DNA transposons. In panels **C–E**, red and blue bars represent the observed numbers of candidate junctions in CAEBV and IM, respectively, and green bars represent the expected numbers based on genomic background. (**F**) Alignment of the sequence around the candidate junction site between the human genome and the EBV genome. Junction boundaries are shown as vertical dashed lines. *SETD3*, *SYPL1*, *ACSL3,* and *NGB* are recurrently targeted host genes associated with candidate EBV–host junctions. All EBV sequences are from the reference strand. Yellow, upstream partner; blue, downstream partner; red, nucleotides that align to both reference sequences (MHs). (**G**) Genomic distribution of recurrently targeted genes (RTGs) in CAEBV. The outer ring depicts chromosomes 1–22, X, and Y. Each red bar indicates the number of CAEBV samples with at least one candidate EBV–host junction overlapping the corresponding host gene. Each concentric interval represents three samples. Selected recurrently targeted genes are labeled. *P* values for panel A were calculated using a two-tailed Student’s *t*-test, whereas *P* values for panels **C–E** were calculated using exact binomial tests. *P < 0.05; **P < 0.01; ns, not significant. LINE, long interspersed nuclear element; SINE, short interspersed nuclear element; LTR, long terminal repeat.

To explore genomic features associated with EBV and host methylation differences, we analyzed capture-derived candidate EBV–host junctions in CAEBV and IM samples. A total of 1,245 candidate EBV–host junction signals were identified in CAEBV PBMC samples (24/24), compared with 148 candidate signals in IM samples (16/17). CAEBV samples contained significantly more detectable candidate junction signals than IM samples; 20 CAEBV samples contained more than 10 junction signals, and one patient had 203 junction signals. Among the 16 IM samples with detectable candidate junctions, counts ranged from 2 to 33 (Fig. 4A).

Viral-side candidate junction coordinates overlapped regions annotated to 62 EBV genes in CAEBV and 34 EBV genes in IM (Fig. S3A and B; Table S4). Junction signals were distributed across the EBV genome with several regions showing relatively frequent detection. In CAEBV PBMCs, candidate junctions were relatively enriched in *EBNA-LP* (34,536–37,678), *EBNA1* (83,123–88,834 and 95,666–97,584), and *BZLF1* (89,231–90,378), whereas relatively frequent candidate junction detection in IM PBMCs was observed in regions involving *EBNA-LP*, *EBNA1,* and *BcLF1* (Fig. 4B).

Human-side candidate junction coordinates mapped to 421 annotated host genes across 20 chromosomes in CAEBV and to 40 host genes in IM, mainly on chromosomes 1, 4–7, 9, 11–14, 16, 17, 20, 22, X, and Y (Table S5). Stratified analysis showed that CAEBV junctions were enriched in intronic and promoter regions but not exons (*P* = 0.0108, Fig. 4C). IM-derived junctions were enriched in introns, whereas the observed number of junctions in promoter regions was lower than expected based on genomic background (*P* = 0.0036). CAEBV junctions were also enriched near common fragile sites (*P* = 0.0104, Fig. 4D). Analysis of repeat elements showed enrichment of junctions within SINE and microsatellite repeats in CAEBV (Fig. 4E). Frequent microhomology was observed between the human- and EBV-derived portions of capture reads spanning candidate junctions (Fig. 4F).

We then calculated the frequency of host genes overlapped by candidate junctions across samples. A total of 29 host genes were recurrently overlapped by candidate EBV–host junctions in CAEBV and IM samples and were designated recurrently targeted genes (RTGs). Five were detected in at least five samples, three were detected in four samples, five were detected in three samples, and 16 were detected in two samples (Fig. S3C; Table S6). Candidate junctions involving *SYPL1*, *SETD3*, *ACSL3*, and *NGB* were detected in 13, 10, six, and four CAEBV samples, respectively. Gene-level recurrence of candidate junctions was more frequently observed in CAEBV, with 19 genes recurrent only in CAEBV compared with one recurrent only in IM. Functional annotation of the 29 RTGs showed enrichment in chromosome organization, regulation of telomere maintenance in response to DNA damage, and protein-histidine N-methyltransferase activity (Fig. S3D). Six of the 29 RTGs (21%) had been reported as cancer-related genes.

#### Genomic overlap between methylation alterations and candidate EBV–host junctions

To describe the genomic overlap between candidate EBV–host junctions and methylation alterations, we identified genes overlapping between the host methylation data set and candidate EBV–host junction annotations in CAEBV and IM. A total of 218 unique overlapping genes were detected; among them, 143 (65.6%) were hypermethylated and 75 (34.4%) were hypomethylated in CAEBV (Table S7). Candidate junctions overlapped the gene bodies of several differentially methylated genes, including *SETD3*, *TET1*, *DNMT3A*, and *DNMT3B*, which showed differential methylation between CAEBV and IM.

We also descriptively compared EBV genome methylation data with viral-side candidate junction annotations and identified 47 overlapping viral genes (Table S8). Twenty genes showed higher methylation levels in CAEBV than in IM. Among them, *BOLF1*, *A73*, and *BALF5* contained viral-side candidate junctions in more than 14 CAEBV samples.

### Exploratory post-treatment observations of EBV and host methylation and detectable candidate EBV–host junctions in PBMCs

**Fig 5:**
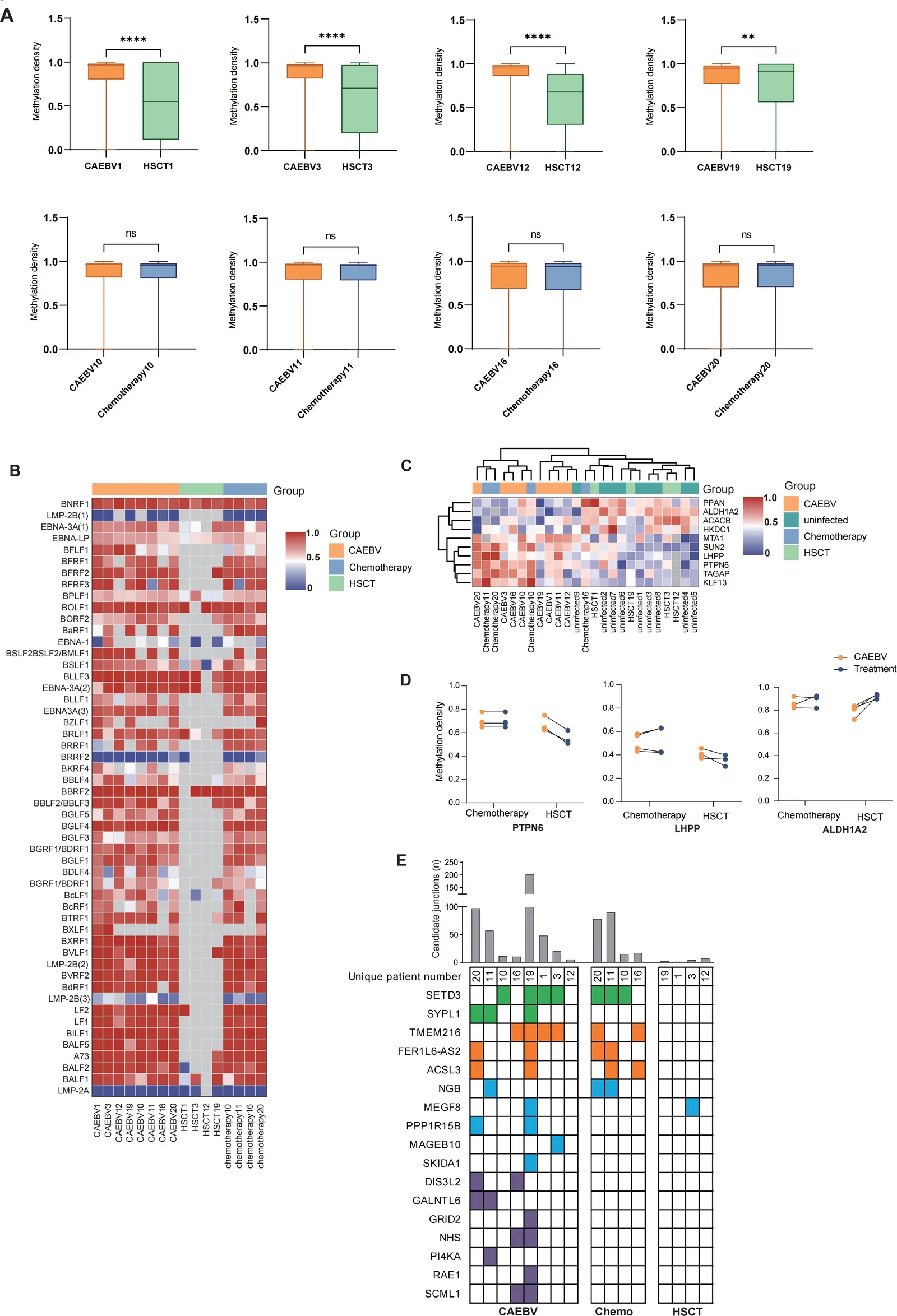
Exploratory post-treatment changes in EBV and host DNA methylation and in detectable candidate EBV–host junctions in patients with CAEBV. (**A**) Changes in EBV DNA methylation levels in patients with CAEBV after treatment. ^**^P < 0.01; ^****^P < 0.0001; ns, not significant. (**B**) Unsupervised hierarchical clustering analysis of EBV DNA methylation in patients with CAEBV after treatment. (**C** and **D**) Clustering analysis of host DNA methylation patterns and paired changes in the methylation levels of representative genes before and after treatment. (**E**) Changes in the number of detectable candidate EBV–host junctions and recurrently targeted genes (RTGs) before and after treatment. All post-treatment comparisons were exploratory observations based on small sample sizes and non-standardized follow-up time points.

Four patients underwent allo-HSCT, and four received cytotoxic chemotherapy alone by the last follow-up (Table S9). All four patients who underwent HSCT achieved clinical complete remission at the last follow-up (at 3, 4, 12, and 6 months after transplantation, respectively), whereas two chemotherapy-treated patients achieved partial remission and two had stable disease. At follow-up time points that varied among patients, EBV DNA methylation levels in post-HSCT PBMC samples were lower than those at baseline, whereas changes of a similar magnitude were not observed after chemotherapy (Fig. 5A and B). We also analyzed host methylation changes before and after treatment. In this exploratory analysis, clustering based on selected DMG patterns separated post-HSCT samples from pretreatment CAEBV samples, and clustering showed that post-HSCT PBMC samples clustered more closely with EBV-uninfected samples at the available follow-up time points, whereas chemotherapy samples continued to cluster with CAEBV samples (Fig. 5C). Tumor-associated genes *LHPP* and *PTPN6* were hypermethylated in CAEBV and chemotherapy samples but hypomethylated in HSCT and uninfected samples; conversely, *ALDH1A2* was hypomethylated in CAEBV and chemotherapy samples but hypermethylated in HSCT and uninfected samples (Fig. 5D). In this exploratory analysis, the number of detectable candidate EBV–host junctions was lower at the available post-HSCT follow-up time points but did not change significantly after chemotherapy (Fig. 5E). RTG analysis showed that candidate junctions involving *SETD3*, *TMEM216*, and *ACSL3* remained detectable after chemotherapy but were not detected in the available post-HSCT PBMC samples (Fig. 5E). Allo-HSCT reconstitutes the immune system with donor-derived hematopoietic cells and may eliminate or replace recipient-derived EBV-infected cells. Therefore, donor chimerism and changes in PBMC cellular composition may largely account for the shift in methylation profiles toward those of uninfected controls and the reduction in detectable candidate EBV–host junctions after HSCT.

## DISCUSSION

In this study, we characterized EBV and host genome methylation profiles together with capture-derived candidate EBV–host junction patterns in patients with CAEBV. EBV showed global hypermethylation in CAEBV compared with IM, with distinct viral methylation features between the two diseases. EBV DNA is generally unmethylated in viral particles (32), whereas latent EBV genomes in host cells are usually methylated (33, 34). EBV promoter activity is inversely related to methylation status, and latent gene expression is regulated by Cp, Wp, and Qp (7). In CAEBV, Cp hypermethylation and Qp hypomethylation were consistent with a “Cp off” type II latency pattern, whereas Cp hypomethylation and Qp hypermethylation in IM were consistent with a “Cp on” type III latency pattern. These findings agree with previous reports in both diseases (35, 36).

Notably, *BALF5* was hypermethylated in CAEBV but hypomethylated in IM. *BALF5* encodes the viral DNA polymerase catalytic subunit. Previous studies showed that *BALF5* deletion may prevent activation of late lytic-cycle genes, which are transcribed from newly replicated EBV genomes during the early lytic phase (37). Okuno et al. (38) reported *BALF5* deletion in EBV genomes from CAEBV patients, which promoted early lytic activation and lymphomagenesis in a xenograft model. These findings suggest that *BALF5* alterations may be relevant to CAEBV biology, although further studies are needed to clarify their functional significance and therapeutic implications.

EBV expresses a limited set of viral genes and miRNAs that can regulate host gene expression through DNA methylation, especially in tumor suppressor genes, thereby contributing to tumorigenesis and malignant transformation (8, 39–41). At the group level, comparison of the independent RRBS and RNA-seq cohorts showed that *E2F1* and *PTPN6* exhibited inverse directions of methylation and expression differences between CAEBV and IM. *PTPN6* has been reported to be hypermethylated in NK/T-cell lymphoma (42), whereas PI3Kδ-dependent *E2F1* activation has been implicated in EBNA1-induced B-cell lymphoma (43). These findings suggest that the methylation profile of CAEBV shares features with certain neoplastic diseases and that abnormal methylation of tumor-associated genes may be associated with CAEBV pathogenesis. Exploratory treatment-related analysis showed DMG patterns that separated post-HSCT PBMC samples from pretreatment samples at the available follow-up time points. *PTPN6* methylation decreased after HSCT, whereas *ALDH1A2* methylation increased; these changes were not evident after chemotherapy alone. Thus, these DMGs may represent exploratory candidate epigenetic markers associated with the observed disease and treatment status, although validation in larger longitudinal cohorts is required. Because hypermethylation-related gene silencing can be reversible, and DNA demethylating agents such as decitabine are used for hematologic malignancies including MDS and AML (44), future studies should evaluate whether demethylating strategies have adjunctive value in CAEBV. However, the differences in DMGs observed before and after HSCT may primarily reflect donor-derived hematopoietic reconstitution, a reduction in recipient-derived infected cells, and changes in cellular composition. Therefore, these findings are insufficient to interpret the DMGs as epigenetic alterations directly induced by HSCT.

EBV does not encode DNMTs, yet EBV and multiple host genes become methylated during latent infection (32). The underlying mechanism remains unclear. In HPV-related disease, viral integration has been proposed as a mechanism contributing to aberrant viral genome methylation and oncogenesis (12, 13). In our study, capture-derived candidate EBV–host junctions were distributed across the EBV genome, with several regions showing relatively frequent detection. This distribution is compatible with heterogeneous genomic accessibility but does not establish the underlying mechanism. Xu et al. (15) reported frequent EBV breakpoints at oriP and terminal repeats in EBV-associated tumors, including NPC and EBVaGC, which differed from the breakpoint regions observed in CAEBV. This difference may reflect disease- or cell-type-related variation, as well as differences in sample composition and sequencing methodology. Nevertheless, both studies identified microhomology near EBV–host junctions and enrichment in fragile genomic regions. The enrichment of candidate junctions near common fragile sites and repetitive elements is compatible with, but does not establish, the involvement of microhomology-associated repair processes, as reported for other oncogenic viruses such as HBV and HPV (45, 46).

EBV fragments have been detected in host genomes of EBV-associated tumors and lymphoblastoid cell lines (47, 48), but the affected genes vary across diseases. In CAEBV, host genes recurrently overlapped by candidate junctions were enriched in annotations related to DNA-damage responses and cancer-associated pathways. *SETD3*, a protein histidine N-methyltransferase, regulates gene expression, cell division, proliferation, and the cell cycle (49). *SETD3* overexpression has been reported to promote hepatocellular carcinoma cell proliferation and migration (50). *ACSL3* has also been implicated in tumor growth and proliferation in prostate cancer (51). The observed coexistence of dispersed candidate junctions and gene-level recurrence is compatible with, but does not demonstrate, potential contributions from genomic accessibility or selective processes during disease evolution (52–54). Based on our data, candidate EBV–host junctions showed genomic overlap with host methylation alterations in CAEBV, but the causal and temporal relationships between these features remain unresolved.

Several limitations should be noted. First, analyses were performed using bulk PBMCs, which cannot define the precise cellular origin of the observed epigenetic patterns and are susceptible to confounding by differences in cellular composition. In particular, the marked expansion of CD8^+^ T cells in IM may partly account for the methylation and expression differences in genes such as *CD8A* and *CD8B* between CAEBV and IM. Future cell-type-resolved studies are therefore needed, especially given the involvement of T/NK cells in CAEBV. Second, RRBS and RNA-seq were performed in independent patient cohorts; therefore, the integrative analysis reflects only the directions of group-level differences. Third, functional studies are required to validate the prioritized genes, clarify the potential relationships between candidate EBV–host junctions and methylation alterations, and assess the therapeutic value of demethylating strategies in CAEBV. Fourth, the treatment analysis included only four patients per group, and post-treatment sampling times were not standardized (3–12 months). Moreover, three HSCT recipients were followed for only 3, 4, and 6 months, respectively. These findings should therefore be regarded as exploratory observations. Fifth, donor-derived hematopoietic reconstitution and the elimination or replacement of recipient-derived EBV-infected cells after allo-HSCT alter the origin and cellular composition of bulk PBMCs. Consequently, the post-HSCT changes in methylation profiles and detectable EBV–host junctions cannot be interpreted as epigenetic reversal within the same recipient cells or direct elimination of the originally detected candidate junction signals. Sixth, we were unable to independently validate the original sequencing events in the same samples using junction-specific PCR and Sanger sequencing. These events should therefore be regarded as candidate EBV–host junctions, and future prospective studies should retain and use the original genomic DNA for orthogonal validation.

In summary, this study mapped EBV and host genome methylation profiles in CAEBV and identified EBV hypermethylation, distinct host methylation patterns, and candidate EBV–host junctions enriched in regions associated with chromosomal instability and repetitive elements. Several tumor-associated genes showed aberrant methylation, and exploratory longitudinal analyses indicated that PBMC methylation patterns after HSCT appeared closer to those of EBV-uninfected samples. Collectively, these findings provide epigenetic and genomic clues to CAEBV pathogenesis and highlight candidate biomarkers and therapeutic hypotheses for further investigation, although their functional relevance and clinical utility require validation in larger, independent, and cell-type-resolved studies.

## Data Availability

All data supporting the findings of this study are available within the article and its supplemental tables.

## SUPPLEMENTAL MATERIAL

Supplemental figures spectrum.01881-26-s0001.pdf Fig. S1 to S3.

Supplemental legends spectrum.01881-26-s0002.docx Legends for Fig. S1 to S3 and Tables S1 to S9.

Supplemental methods spectrum.01881-26-s0003.docx Supplemental methods.

Supplemental tables spectrum.01881-26-s0004.pdf Tables S1 to S9.

ASM does not own the copyrights to Supplemental Material that may be linked to, or accessed through, an article. The authors have granted ASM a non-exclusive, world-wide license to publish the Supplemental Material files. Please contact the corresponding author directly for reuse.
