# Supplementary material for: Integrated EBV and host DNA methylation profiling identifies distinct candidate EBV–host junction patterns in chronic active EBV infection
Source: Microbiol Spectr. 2026 Sep 25;14(11):e01881-26. doi: 10.1128/spectrum.01881-26 (PMC13729105; doi:10.1128/spectrum.01881-26)

supplemental Figure 1

A

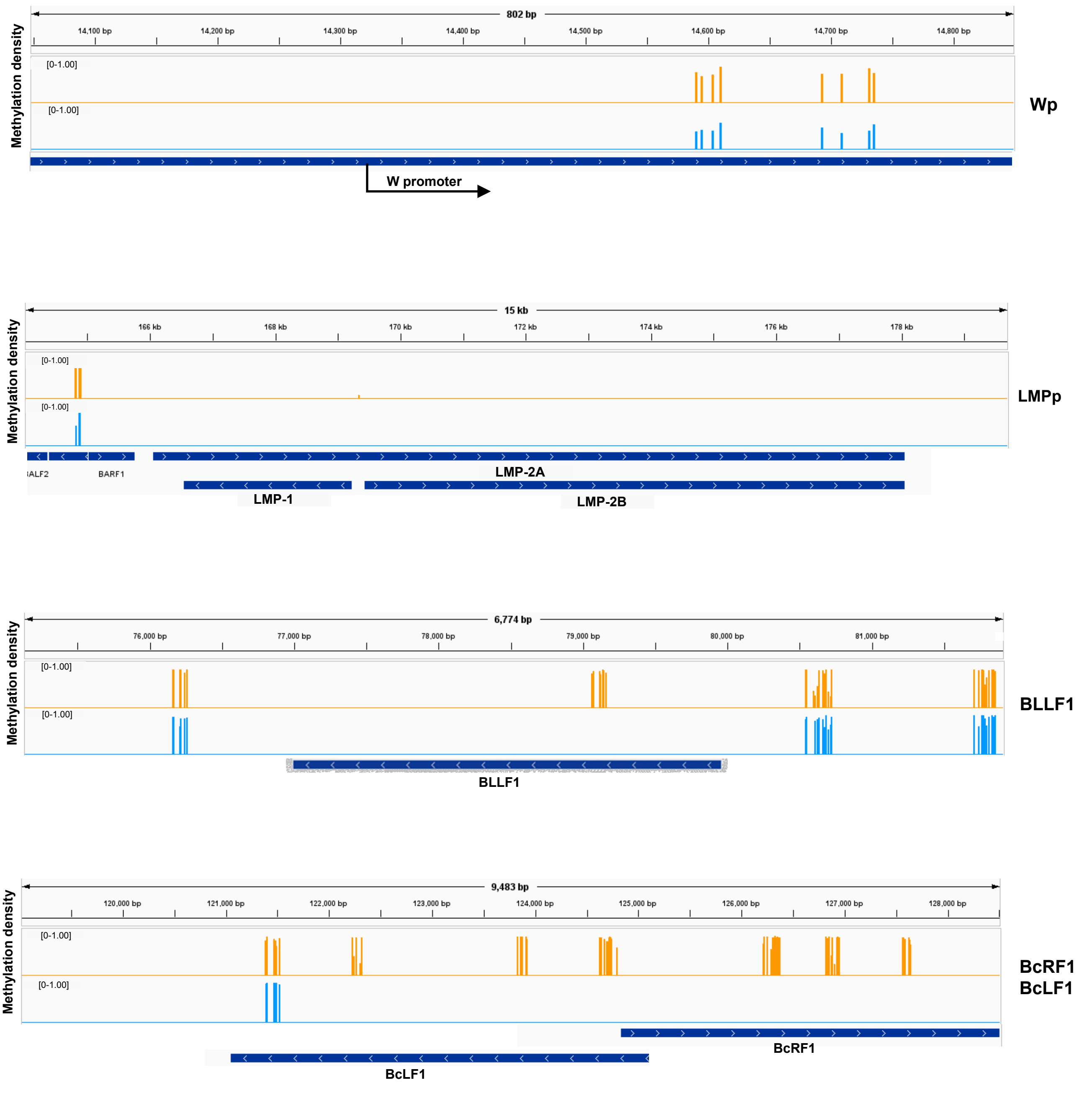

B

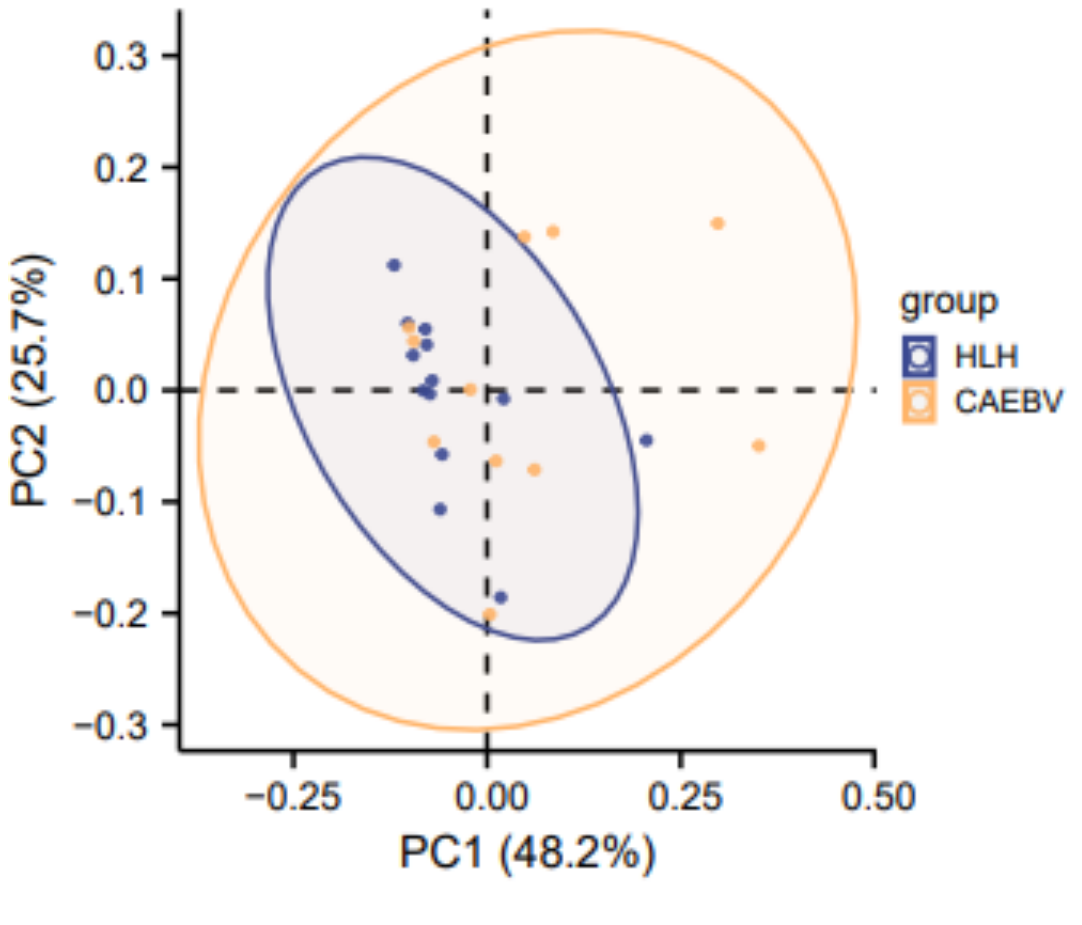

C

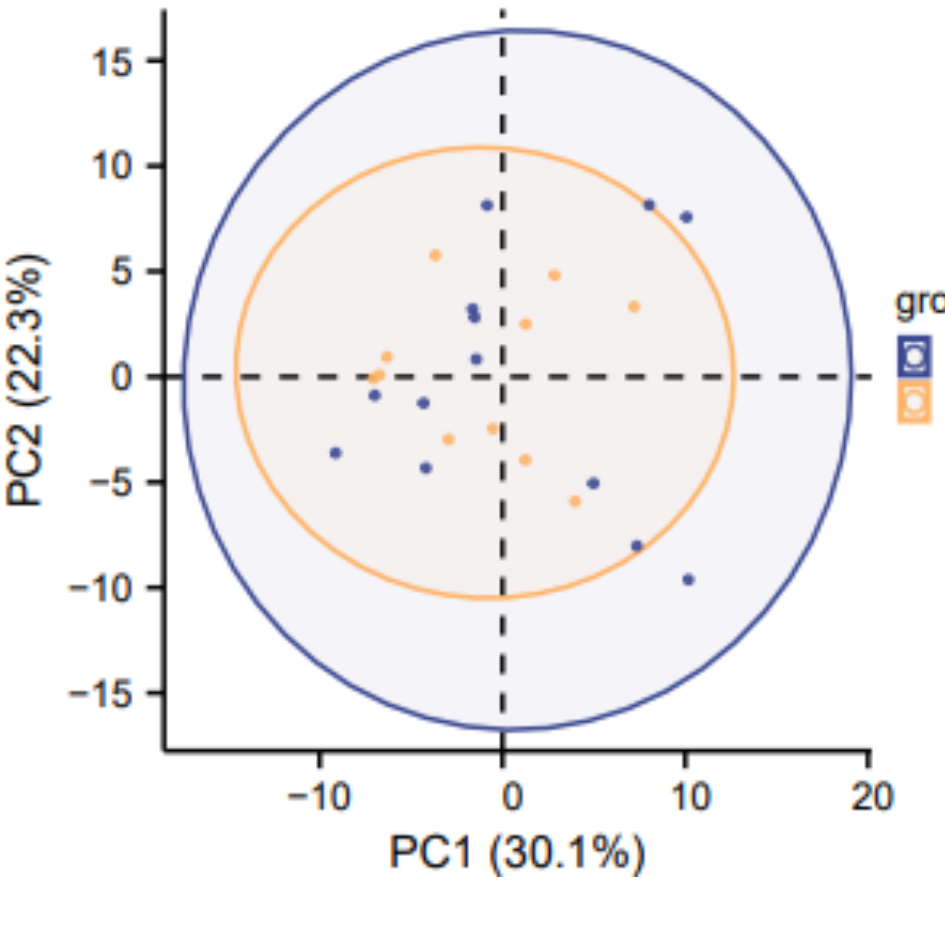

supplemental Figure 2

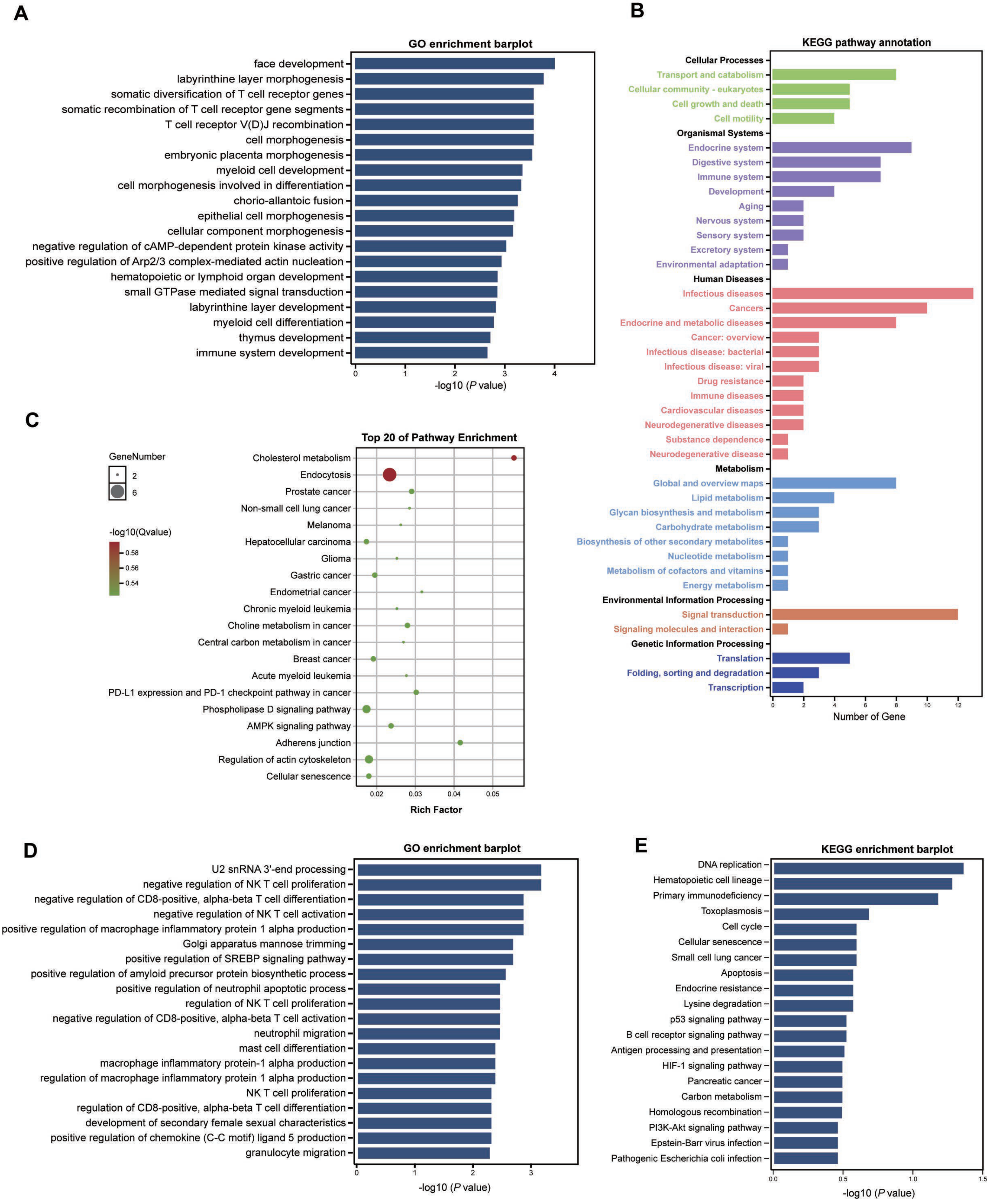

supplemental Figure 3

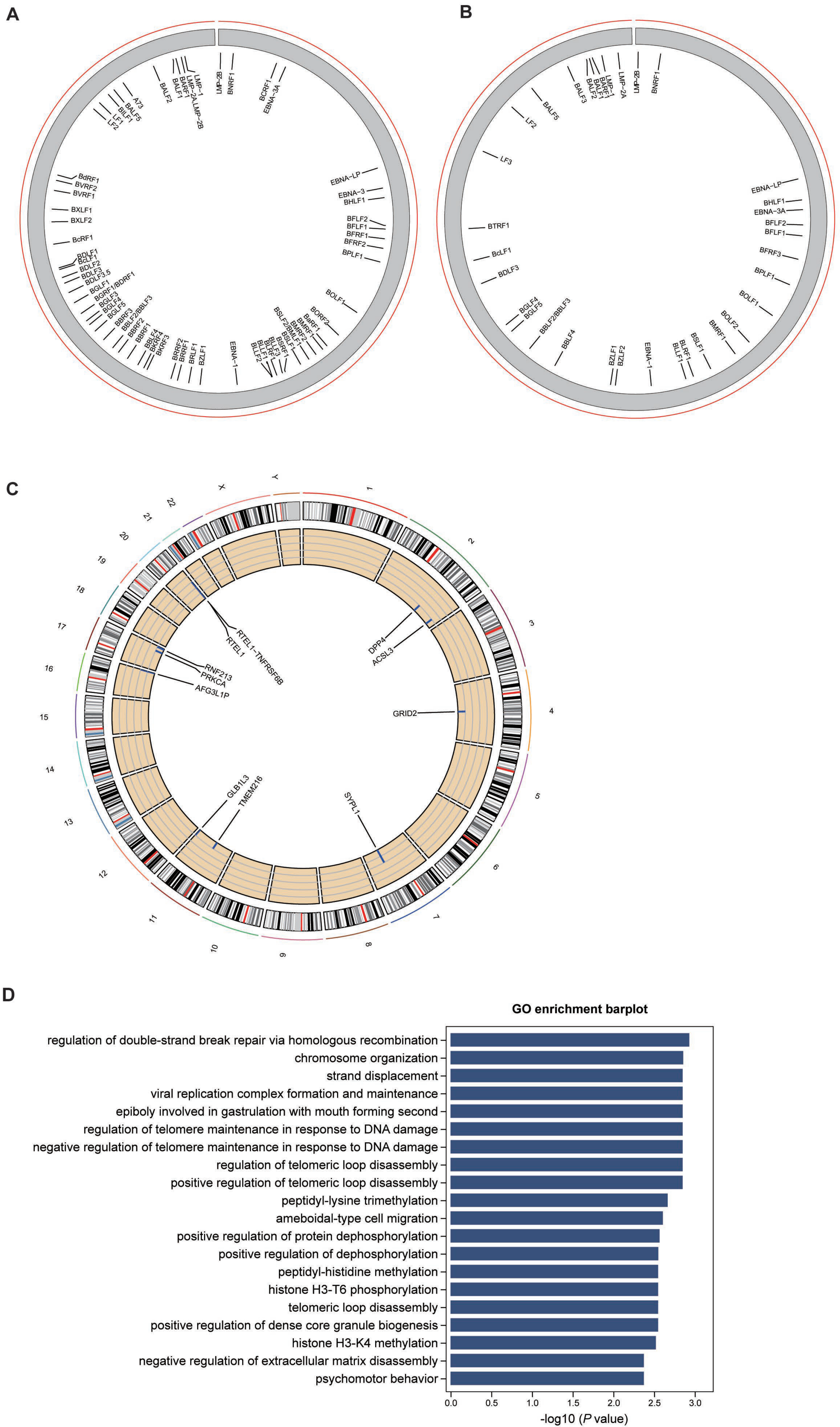

Supplement: Supplementary file 1 [file spectrum.01881-26-s0001.pdf]
