# Supplementary material for: Integrated EBV and host DNA methylation profiling identifies distinct candidate EBV–host junction patterns in chronic active EBV infection
Source: Microbiol Spectr. 2026 Sep 25;14(11):e01881-26. doi: 10.1128/spectrum.01881-26 (PMC13729105; doi:10.1128/spectrum.01881-26)
Supplement: Supplementary file 2 [file spectrum.01881-26-s0004.pdf]

Table S1. Examination parameters of patients with CAEBV, IM HC and uninfected groups

Group	Patient	Sexuality	Age at onset, y	Anti EBV antibody				EBV loads in plasma (copies/mL)	EBV loads in whole blood (copies/mL)
				VCA-IgM	VCA-IgG	EBNA1-IgG	Affinity of VCA-IgG antibody（H/L）		
CAEBV	CAEBV 1	M	7	–	+	+	H	3×10^3	3.49×10^6
	CAEBV 2	M	2	–	+	+	H	1.32×10^3	6.86×10^6
	CAEBV 3	M	7	–	+	+	H	2.23×10^4	3.76×10^6
	CAEBV 4	M	14	–	+	+	H	<500	6.19×10^6
	CAEBV 5	M	5	–	+	+	H	1.67×10^3	1.17×10^7
	CAEBV 6	F	10	–	+	+	H	5×10^2	1.89×10^7
	CAEBV 7	F	10	–	+	+	H	1.85×10^3	1.21×10^7
	CAEBV 8	M	7	–	+	+	H	9.42×10^2	1.86×10^7
	CAEBV 9	F	11	–	+	+	H	<500	1.28×10^7
	CAEBV 10	M	13	–	+	+	H	1.3×10^3	1.65×10^6
	CAEBV 11	M	12	–	+	+	H	4.15×10^3	6.59×10^6
	CAEBV 12	M	8	–	+	+	H	5.02×10^4	9.08×10^5
	CAEBV 13	M	9	–	+	+	H	<500	5.62×10^6
	CAEBV 14	F	7	–	+	+	H	3.54×10^3	3.29×10^6
	CAEBV 15	M	8	–	+	+	H	6.37×10^2	5.61×10^6
	CAEBV 16	F	9	–	+	+	H	1.72×10^3	6.52×10^5
	CAEBV 17	F	7	–	+	+	H	<500	7.41×10^6
	CAEBV 18	M	2	–	+	+	H	5.96×10^2	4.55×10^6
	CAEBV 19	F	6	–	+	+	H	<500	5.37×10^6
	CAEBV 20	M	12	–	+	+	H	6.24×10^4	1.4×10^7
	CAEBV 21	M	8	–	+	+	H	<500	4.14×10^6
	CAEBV 22	M	1	–	+	+	H	<500	4.99×10^4
	CAEBV 23	M	5	–	+	+	H	1.46×10^5	2.56×10^5
	CAEBV 24	M	5	–	+	+	H	<500	1.78×10^3
	CAEBV 25	M	10	–	+	+	H	9.44×10^3	1.26×10^6
	CAEBV 26	F	7	–	+	+	H	6.33×10^2	1.29×10^5
	CAEBV 27	M	11	–	+	+	H	<500	6.83×10^5
	CAEBV 28	F	4	–	+	+	H	1.60×10^3	2.28×10^7
	CAEBV 29	M	11	–	+	+	H	<500	2.99×10^6
	CAEBV 30	M	6	–	+	+	H	9.58×10^2	7.96×10^6
	CAEBV 31	M	11	–	+	+	H	<500	7.17×10^5
	CAEBV 32	F	5	–	+	+	H	<500	9.01×10^6
IM	IM 1	M	7	+	+	–	L	5×10^2	-
	IM 2	F	8	+	+	–	L	5×10^2	-
	IM 3	F	3	+	+	–	L	6.46×10^2	-
	IM 4	F	6	+	+	–	L	7.25×10^2	-

	**IM 5**	M	8	+	+	–	L	2.37×10^3	-
	**IM 6**	M	4	+	+	–	L	7.49×10^3	-
	**IM 7**	M	6	+	+	–	L	1.75×10^3	-
	**IM 8**	F	6	+	+	–	L	5.25×10^2	-
	**IM 9**	F	4	+	±	–	L	1.21×10^3	-
	**IM 10**	F	10	+	±	–	L	7.69×10^2	-
	**IM 11**	M	6	+	+	–	L	5×10^2	-
	**IM 12**	M	1	+	+	+	L	1.74×10^3	-
	**IM 13**	M	7	+	+	–	L	4.82×10^3	-
	**IM 14**	M	2	+	+	–	L	2.34×10^3	-
	**IM 15**	M	8	+	+	–	L	1.34×10^3	-
	**IM 16**	F	4	+	±	–	L	5×10^2	-
	**IM 17**	M	5	+	+	–	L	7.07×10^2	-
	**IM 18**	M	8	+	+	–	L	5×10^2	-
	**IM 19**	F	4	+	+	–	L	5×10^2	-
	**IM 20**	M	2	+	+	–	L	5×10^2	-
	**IM 21**	M	4	+	+	–	L	5×10^2	-
	**IM 22**	F	7	+	+	–	L	5×10^2	-
	**IM 23**	M	6	+	+	–	L	1.43×10^3	-
	**IM 24**	M	9	+	+	–	L	5×10^2	-
	**HC 1**	M	11	–	+	+	H	<500	-
	**HC 2**	F	6	–	+	+	H	<500	-
	**HC 3**	F	11	–	+	+	H	<500	-
	**HC 4**	M	4	–	+	+	H	<500	-
**HC**	**HC 5**	M	4	–	+	+	H	<500	-
	**HC 6**	M	12	–	+	+	H	<500	-
	**HC 7**	F	8	–	+	+	H	<500	-
	**HC 8**	M	2	–	+	+	H	<500	-
	**HC 9**	M	1	–	+	+	H	<500	-
	**uninfected 1**	F	2	–	–	–	-	-	-
	**uninfected 2**	M	1	–	–	–	-	-	-
	**uninfected 3**	M	1	–	–	–	-	-	-
	**uninfected 4**	M	2	–	–	–	-	-	-
**uninfected**	**uninfected 5**	M	5	–	–	–	-	-	-
	**uninfected 6**	F	4	–	–	–	-	-	-
	**uninfected 7**	M	1	–	–	–	-	-	-
	**uninfected 8**	M	1	–	–	–	-	-	-
	**uninfected 9**	M	1	–	–	–	-	-	-

CAEBV, chronic active Epstein–Barr virus infection; IM, infectious mononucleosis; HC, healthy carriers with EBV; uninfected, EBV uninfected children; M, male; F, Female; VCA, virus capsid antigen; EBNA, EBV nuclear antigen; "–", not tested; "<500", below the limit of quantification or detection of the clinical assay.

Table S2. Numbers of DMRs detected in CAEBV versus IM, HC and uninfected groups

	CAEBV versus IM	CAEBV versus HC	CAEBV versus uninfected group
Hypermethylated DMRs	21,638 (75.46%)	17,446 (57.81%)	12,506 (33.3%)
Hypomethylated DMRs	7,036 (24.54%)	12,731 (42.19%)	25,045 (66.7%)
total	28,674	30,177	37,551

DMRs, differentially methylated regions; CAEBV, chronic active Epstein–Barr virus disease; IM,infectious mononucleosis; HC, healthy carriers with EBV; uninfected, EBV uninfected children.

Table S3

Sheet: Sheet1

	Refseq_gene	Mean methylation difference	Expression (log2FC)
	ACOT7	0.107025	-2.16463076949
	AHCY	0.12113	-1.12200345658
	CD248	0.336987	-1.96502738452
	CD8A	0.38032	-2.04663932906
	CD8B	0.342989	-1.73705755304
	CDCA7	0.167288	-2.30889079224
	CDT1	0.189044	-3.12001508286
	CENPN	0.30753	-1.62199495011
	CHEK1	0.155001	-2.32581120613
	CLSPN	0.173971	-2.88498706405
	CTPS1	0.128051	-1.04021336879
	CYTOR	0.175239	-1.2498107993
	DBN1	0.151782	-1.25372004962
	DMC1	0.151421	-3.11142155328
	DNA2	0.130857	-1.53308637593
	E2F1	0.2977	-2.32046096891
	EZH2	0.244648	-1.69384224939
	FBXW4P1, BCR	0.120558	-1.56502323133
Hyper E-	GALNT18	0.131148	-7.04696373527
79 genes	GAPDH	0.199542	-1.06758416994
	GMNN	0.160253	-2.07457611879
	GTSE1	0.121296	-2.24797356137
	HADH	0.108979	-1.29239655492
	HK2	0.127827	-2.65530098863
	HMGA1	0.108156	-1.13199638793
	HMGB3	0.182522	-1.48846390066
	HSP90AA1	0.108158	-1.01925965461
	HSPE1-MOB4, HSPE1	0.387421	-1.07740226795
	IDH2	0.218814	-1.41821323904
	IL32	0.268298	-1.3344635684
	KIF18B	0.111241	-2.44280447338
	KLF5	0.118189	-1.58500738936
	LAG3	0.320735	-1.64100591835
	LMNB1	0.239895	-1.2455505238
	LMNB2	0.173581	-1.0817257808
	MAP1LC3A	0.24189	-1.32011734372
	MCM10	0.147796	-2.92445554085
	MCM2	0.126993	-2.35970045801

	Refseq_gene	Mean methylation difference	Expression (log2FC)
	MCM5	0.102643	-1.55099071167
	MCM6	0.171836	-1.93178425446
	MELK	0.169752	-2.63385039529
	MIR4435-2HG	0.407212	-1.24195253046
	MME	0.104393	-4.27699387748
	MTHFD1	0.154847	-1.45529558039
	MYBL2	0.303471	-2.13211633471
	NASP	0.138813	-1.41310325914
	NBN	0.183194	-1.05300162098
	NCAPH	0.131068	-2.90585643844
	NDC80	0.109109	-1.92102661139
	NSD2	0.118095	-1.05272855855
	OGDHL	0.206465	-4.23978260351
	PCLAF	0.158169	-2.90433611995
	PLIN2	0.129538	-2.92863586084
	POC1A	0.148843	-2.3201084011
	RAD51	0.212267	-2.49522400633
	RAD54L	0.178964	-2.32272577473
	RCC1	0.290022	-1.4057151502
	RDM1	0.108549	-3.13114292256
	RECQL4	0.115353	-1.30174699324
	RFC4	0.109199	-1.52822219033
	RTP5	0.24619	-1.92613068879
	SAE1	0.189455	-1.05751536014
	SFXN1	0.307374	-1.12937219315
	SHMT1	0.112654	-1.16055588059
	SIT1	0.16746	-1.09428350888
	SLA2	0.111577	-1.31841254565
	SLBP	0.216558	-1.06102809329
	SLC2A3	0.145741	-1.24979355712
	SPAG5	0.117964	-1.60553120864
	SPC24	0.20354	-2.56398479423
	STIL	0.288401	-2.14037359241
	TOX2	0.264153	-2.05136924394
	TUBB	0.140415	-1.74084450964
	UBE2T	0.125415	-2.12081361866
	UHRF1	0.353503	-2.43192490589
	VEGFA	0.120231	-2.88826570391
	WDR34	0.104723	-1.51019129376
	WDR62	0.240496	-2.06340625208
	YWHAE	0.185291	-1.0429931894
	ADAM12	-0.141446	2.42468296379
	AHNAK	-0.131836	1.00827894163
	AK8	-0.125493	3.71759960964

	Refseq_gene	Mean methylation difference	Expression (log2FC)
	ANK1	-0.178283	3.16711553273
	ARHGAP5	-0.130986	1.44100780992
	B4GALT6	-0.241267	2.26422906901
	BANK1	-0.11788	1.4103739564
	BCL2	-0.141735	1.86498168533
	BCL2L12	-0.10179	1.89806217765
	C16orf74	-0.101024	1.40476560162
	CD79B	-0.156415	1.25023357143
	CLCC1	-0.121846	1.28203421755
	CNTNAP2	-0.101728	2.36849410307
	EPPK1	-0.117155	2.82617643306
	FAM117A	-0.166899	1.2531966588
	FHDC1	-0.199759	4.37225461795
	GMPR	-0.174173	2.59089485182
	GNG7	-0.189641	2.09011483529
	GPC3	-0.127407	4.59917415765
	HIPK2	-0.192735	1.13718205935
	HOXA10-HOXA9, HOXA10	-0.105075	3.33319646148
	HVCN1	-0.12965	1.25611121053
	IGHG4	-0.111925	3.75837746532
	IGHGP	-0.106427	3.19353904437
	IGLV3-19	-0.138277	2.07021623466
	IL9RP3	-0.10864	2.11623342027
	LARGE1	-0.142531	2.01473017133
	METTL7A	-0.250399	1.56532376119
	MTCL1	-0.149941	2.31950833571
	NEK6	-0.119309	1.23891848349
	NOL4L	-0.149922	1.06669636524
	NSG1	-0.100888	1.44174048449
	PAX5	-0.160304	1.99394514858
	PCDH9	-0.100625	2.27510058319
	PLCH2	-0.108144	1.6250855415
Hypo E+	PLEC	-0.188059	1.20794528438
57 genes	PLVAP	-0.138171	3.39767868881
	PNOC	-0.18834	2.55189476738
	POU2F2	-0.128741	1.39101395903
	PTPN13	-0.114725	2.84202834001
	SEL1L3	-0.101957	1.28952800037
	SIPA1L3	-0.105278	1.09407674615
	SKI	-0.100345	1.15160501945
	SLC12A7	-0.208295	1.01935784421
	SLC44A1	-0.174741	1.17052895808
	SLC4A1	-0.165769	4.78841262834
	SLC6A8	-0.128492	4.12861961225

	Refseq_gene	Mean methylation difference	Expression (log2FC)
	SOGA1	-0.111371	1.67099119653
	SPECC1	-0.115487	1.15620625483
	SYNPO	-0.111443	4.25903186696
	TLE1	-0.112711	1.43279198991
	TMEM178B	-0.111635	3.54919423751
	TMOD2	-0.110319	1.46462279969
	TSPAN15	-0.11941	2.50321041845
	WDFY4	-0.113794	1.45211512663
	ZNF532	-0.106444	1.25454121442
	ZNF717	-0.102519	1.41952366498
	ASF1B	-0.220034	-2.42585597336
	CASZ1	-0.130546	-1.10553945765
	CHAF1A	-0.144337	-1.60464412651
Hypo E-	PSMA8, DHFRP1	-0.163775	-1.99636957075
8 genes	FBXO43	-0.117344	-2.65529716291
	GINS3	-0.112152	-1.90438753868
	MRPL1	-0.100456	-1.10369430713
	TNFAIP6, LOC101929319	-0.142707	-3.58615866195
	ADAM19	0.277402	1.44586942158
	ADCY9	0.116282	1.30000430322
	ADD2	0.156921	1.66780324382
	ADGRB2	0.17909	2.70266032507
	AKR1E2	0.12773	3.03420163665
	AKT3	0.211965	1.28827391591
	ANK3	0.232798	1.70191772397
	ATP8B2	0.134165	1.18016223324
	BMP8B	0.191649	1.91102026647
	C11orf21	0.107543	1.14897928524
	CBFA2T2	0.132782	1.21815063671
	CCDC85C	0.144343	1.49178223163
	CDH20	0.100011	3.45359826649
Hyper E+	CDK5R1	0.19398	1.6334715886
105 genes	CIITA	0.110105	1.3494259773
	CLCN4	0.110695	1.80921917755
	CLEC17A	0.112541	1.9122318957
	CNR2	0.212896	1.69748948437
	CNTNAP1	0.111817	2.05056724327
	CR2	0.117032	2.01795090916
	CRTC1	0.233936	1.80290828935
	DACT1	0.11689	2.47392230991
	DBNDD1	0.245834	1.92904504629
	DDR1	0.115221	1.34807970495
	DENND5B-AS1, DENND5B	0.113857	2.1180992474
	DISP1	0.150202	1.71539465812

	Refseq_gene	Mean methylation difference	Expression (log2FC)
	DLEC1	0.126756	2.21837687528
	DLL1	0.104067	1.8462484245
	DMTN	0.113643	2.38746123108
	DNAJC6	0.129161	4.04650389646
	FBXL16	0.213849	1.72993275655
	FCER2	0.348152	1.53900159048
	FKBP8	0.100547	1.95562375191
	FOXP3	0.164902	1.93714089884
	FRMD4A	0.18619	3.65262217022
	HAGH	0.145823	1.54058752149
	HRK	0.219427	1.79429047111
	IGF2BP2	0.126414	1.91094963999
	IGFBP3	0.100522	3.38316226363
	IGHV4-4	0.1378	3.92139195359
	IGHV4-59	0.113565	3.12018865285
	IL17RE	0.173531	1.98395716618
	ITGA6	0.132472	1.47704874013
	KANK1	0.124599	2.364126462
	KCNA2	0.167516	2.81381369048
	KCNG1	0.143885	2.47554626102
	KIF5C	0.164473	1.76018801207
	KLHL14	0.124115	2.5625190307
	L1CAM, LCA10	0.10988	2.33113495128
	LAMC1	0.153263	1.72199870461
	LDLRAP1	0.247058	1.79229692511
	LRRC3	0.160981	1.89023830123
	LTK	0.185772	1.68602618618
	MAML2	0.120697	1.11973018893
	MICAL3	0.162985	2.01701757711
	MOSPD2	0.119631	1.07795331157
	MYO1B	0.107827	2.2229375053
	MYO1D	0.157744	2.33050464235
	MYO1E	0.121771	1.52057398337
	MZB1	0.108747	2.1002271651
	NFIX	0.164587	1.95943760297
	NLRP7	0.134765	4.87198973097
	OSBP2	0.280106	2.86504376995
	PARM1	0.11705	2.8326007936
	TRAPPC9, PEG13	0.137696	1.99277153492
	POU2AF1	0.159645	1.50469631215
	PRDM11	0.199482	1.71003886337
	PRDM2	0.235216	1.71599177389
	PRKXP1	0.115576	1.84451551105
	RAB11FIP5	0.120647	1.77685476925

	Refseq_gene	Mean methylation difference	Expression (log2FC)
	RAPGEF5	0.109815	3.06039706206
	RGMB	0.180941	2.93497414768
	RHOB	0.159601	1.2347158316
	RNF182	0.14966	6.25788606282
	RNF207	0.134251	2.28888199229
	SARM1	0.127868	1.08121270013
	SCN3A	0.212042	2.4735520853
	SIGLEC17P, LOC101928517	0.139253	2.47755665563
	SLC25A43	0.106927	1.66573578266
	SNX22	0.102227	2.26133426794
	SOCS1	0.188303	1.53088505632
	ST6GAL1	0.235332	1.1209254346
	SUSD4	0.235097	1.73378883649
	SYNPO2L	0.246322	2.17356762261
	TMEM30B	0.146032	1.52884424545
	TMEM63A	0.266925	1.15950343464
	TMEM65	0.101443	1.05434079863
	TMOD1	0.129644	4.67169668046
	TNFRSF13B	0.148853	1.70556325666
	TOM1L2	0.323216	1.5700920134
	TRIM58	0.105605	2.91468781165
	TSPAN18	0.198757	2.75059993911
	TSPAN32	0.13207	1.00399972143
	TTBK2	0.163527	1.14811839861
	TTC9	0.125035	1.19144112191
	TUNAR	0.114475	4.07360483161
	UHRF1BP1	0.107545	1.53198994727
	ZDHHC19	0.109693	4.38875939751
	ZDHHC23	0.111988	2.06097930841
	ZNF347	0.147778	1.40194251461
	ZNF441	0.195098	1.67472229958
	ZNF696	0.104641	1.61128404993
	ZNF844	0.118066	1.2735379583
	ZNF862	0.128413	1.17622629916
	ZSCAN9	0.118961	1.38222903704

Table S4

Sheet: CAEBV

Row 2: gene = A73

gene: A73

sample: CAEBV1, CAEBV11, CAEBV13, CAEBV14, CAEBV18, CAEBV19, CAEBV2, CAEBV20-1, CAEBV21, CAEBV3, CAEBV5, CAEBV6, CAEBV7, CAEBV8, CAEBV9

pos: gammaEBV4-153617, gammaEBV4-153639, gammaEBV4-153725, gammaEBV4-153763, gammaEBV4-153860, gammaEBV4-153864, gammaEBV4-153952, gammaEBV4-154055, gammaEBV4-154299, gammaEBV4-154429, gammaEBV4-154453, gammaEBV4-154654, gammaEBV4-154720, gammaEBV4-154982, gammaEBV4-155166, gammaEBV4-155263, gammaEBV4-155584, gammaEBV4-155832, gammaEBV4-156147, gammaEBV4-156363, gammaEBV4-156375, gammaEBV4-156562, gammaEBV4-156606, gammaEBV4-157058, gammaEBV4-157364, gammaEBV4-157434, gammaEBV4-157853, gammaEBV4-159158, gammaEBV4-159376, gammaEBV4-160788, gammaEBV4-161004, gammaEBV4-161324, gammaEBV4-161348, gammaEBV4-161445, gammaEBV4-161533, gammaEBV4-162029, gammaEBV4-162140, gammaEBV4-162204, gammaEBV4-162480

Row 3: gene = BALF1

gene: BALF1

sample: CAEBV1, CAEBV10, CAEBV11, CAEBV19, CAEBV2, CAEBV20-1, CAEBV21, CAEBV5, CAEBV6, CAEBV8, CAEBV9

pos: gammaEBV4-162480, gammaEBV4-162703, gammaEBV4-162758, gammaEBV4-163569, gammaEBV4-163668, gammaEBV4-163804, gammaEBV4-163829, gammaEBV4-164100, gammaEBV4-164161, gammaEBV4-164382, gammaEBV4-164387, gammaEBV4-164551, gammaEBV4-164586, gammaEBV4-164676, gammaEBV4-164692, gammaEBV4-164787, gammaEBV4-164982, gammaEBV4-165051, gammaEBV4-165155, gammaEBV4-165639, gammaEBV4-165807, gammaEBV4-166017, gammaEBV4-166116, gammaEBV4-166237, gammaEBV4-166370, gammaEBV4-166656, gammaEBV4-166695, gammaEBV4-166813

Row 4: gene = BALF2

gene: BALF2

sample: CAEBV1, CAEBV19, CAEBV2, CAEBV20, CAEBV21, CAEBV5, CAEBV6, CAEBV7, CAEBV8, CAEBV9

pos: gammaEBV4-159158, gammaEBV4-159376, gammaEBV4-160788, gammaEBV4-161004, gammaEBV4-161324, gammaEBV4-161348, gammaEBV4-161445, gammaEBV4-161533, gammaEBV4-162029, gammaEBV4-162140, gammaEBV4-162204, gammaEBV4-162480, gammaEBV4-162703, gammaEBV4-162758, gammaEBV4-163569, gammaEBV4-163668, gammaEBV4-163804, gammaEBV4-163829, gammaEBV4-164100, gammaEBV4-164161, gammaEBV4-164382, gammaEBV4-164387, gammaEBV4-164551, gammaEBV4-164586, gammaEBV4-164676, gammaEBV4-164692, gammaEBV4-164787, gammaEBV4-164982, gammaEBV4-165051, gammaEBV4-165155, gammaEBV4-165639, gammaEBV4-165807, gammaEBV4-166017, gammaEBV4-166116, gammaEBV4-166237

Row 5: gene = BALF5

gene: BALF5

sample: CAEBV1, CAEBV11, CAEBV13, CAEBV14, CAEBV17, CAEBV18, CAEBV19, CAEBV2, CAEBV20, CAEBV3, CAEBV5, CAEBV6, CAEBV8, CAEBV9

pos: gammaEBV4-151271, gammaEBV4-151272, gammaEBV4-152545, gammaEBV4-152557, gammaEBV4-152613, gammaEBV4-153004, gammaEBV4-153077, gammaEBV4-153117, gammaEBV4-153180, gammaEBV4-153617, gammaEBV4-153639, gammaEBV4-153725, gammaEBV4-153763, gammaEBV4-153860, gammaEBV4-153864, gammaEBV4-153952, gammaEBV4-154055, gammaEBV4-154299, gammaEBV4-154429, gammaEBV4-154453, gammaEBV4-154654, gammaEBV4-154720, gammaEBV4-154982, gammaEBV4-155166, gammaEBV4-155263, gammaEBV4-155584, gammaEBV4-155832, gammaEBV4-156147, gammaEBV4-156363, gammaEBV4-156375, gammaEBV4-156562, gammaEBV4-156606, gammaEBV4-157058, gammaEBV4-157364, gammaEBV4-157434, gammaEBV4-157853

Row 6: gene = BaRF1

gene: BaRF1

sample: CAEBV1, CAEBV11, CAEBV19, CAEBV2, CAEBV20, CAEBV5, CAEBV6, CAEBV7, CAEBV8, CAEBV9

pos: gammaEBV4-64795, gammaEBV4-64976, gammaEBV4-65127, gammaEBV4-65205, gammaEBV4-65433, gammaEBV4-65450, gammaEBV4-65685, gammaEBV4-65718, gammaEBV4-65785, gammaEBV4-65950, gammaEBV4-66061, gammaEBV4-66069, gammaEBV4-66363, gammaEBV4-66405, gammaEBV4-66612, gammaEBV4-66809, gammaEBV4-67279, gammaEBV4-67485, gammaEBV4-67505, gammaEBV4-67749, gammaEBV4-68057, gammaEBV4-68261, gammaEBV4-68279, gammaEBV4-68497, gammaEBV4-68583, gammaEBV4-68589, gammaEBV4-68612, gammaEBV4-68646, gammaEBV4-68657, gammaEBV4-68729, gammaEBV4-68748, gammaEBV4-69148, gammaEBV4-69192, gammaEBV4-69276

Row 7: gene = BARF1

gene: BARF1

sample: CAEBV1, CAEBV10, CAEBV11, CAEBV13, CAEBV19, CAEBV2, CAEBV20, CAEBV21, CAEBV5, CAEBV6, CAEBV8, CAEBV9

pos: gammaEBV4-163569, gammaEBV4-163668, gammaEBV4-163804, gammaEBV4-163829, gammaEBV4-164100, gammaEBV4-164161, gammaEBV4-164382, gammaEBV4-164387, gammaEBV4-164551, gammaEBV4-164586, gammaEBV4-164676, gammaEBV4-164692, gammaEBV4-164787, gammaEBV4-164982, gammaEBV4-165051, gammaEBV4-165155, gammaEBV4-165639, gammaEBV4-165807, gammaEBV4-166017, gammaEBV4-166116, gammaEBV4-166237, gammaEBV4-166370, gammaEBV4-166656, gammaEBV4-166695, gammaEBV4-166813, gammaEBV4-167362, gammaEBV4-167370, gammaEBV4-167399, gammaEBV4-167404, gammaEBV4-167437, gammaEBV4-167488, gammaEBV4-167506

Row 8: gene = BBLF2/BBLF3

gene: BBLF2/BBLF3

sample: CAEBV11, CAEBV14, CAEBV18, CAEBV19, CAEBV2, CAEBV20, CAEBV21, CAEBV5, CAEBV6, CAEBV7, CAEBV9

pos: gammaEBV4-102548, gammaEBV4-102833, gammaEBV4-102972, gammaEBV4-103167, gammaEBV4-103346, gammaEBV4-103649, gammaEBV4-103838, gammaEBV4-104080, gammaEBV4-104243, gammaEBV4-104986, gammaEBV4-104994, gammaEBV4-105325, gammaEBV4-105501, gammaEBV4-105542, gammaEBV4-105598, gammaEBV4-106045, gammaEBV4-106231, gammaEBV4-106388, gammaEBV4-106430, gammaEBV4-107019, gammaEBV4-107473, gammaEBV4-107738, gammaEBV4-108135, gammaEBV4-108164, gammaEBV4-108165, gammaEBV4-108219, gammaEBV4-108226, gammaEBV4-108228

Row 9: gene = BBLF4

gene: BBLF4

sample: CAEBV1, CAEBV11, CAEBV14, CAEBV16, CAEBV18, CAEBV19, CAEBV2, CAEBV20, CAEBV21, CAEBV5, CAEBV6, CAEBV8, CAEBV9

pos: gammaEBV4-100012, gammaEBV4-100015, gammaEBV4-100017, gammaEBV4-100032, gammaEBV4-100470, gammaEBV4-100929, gammaEBV4-101078, gammaEBV4-101090, gammaEBV4-101143, gammaEBV4-101244, gammaEBV4-101896, gammaEBV4-101996, gammaEBV4-102132, gammaEBV4-102279, gammaEBV4-102548, gammaEBV4-102833, gammaEBV4-102972, gammaEBV4-103167, gammaEBV4-103346, gammaEBV4-103649, gammaEBV4-103838, gammaEBV4-97584, gammaEBV4-97632, gammaEBV4-98321, gammaEBV4-98485, gammaEBV4-98607, gammaEBV4-98649, gammaEBV4-98857, gammaEBV4-99257, gammaEBV4-99261, gammaEBV4-99275, gammaEBV4-99399, gammaEBV4-99402, gammaEBV4-99405, gammaEBV4-99422, gammaEBV4-99423, gammaEBV4-99440, gammaEBV4-99441, gammaEBV4-99535

Row 10: gene = BBRF1

gene: BBRF1

sample: CAEBV11, CAEBV14, CAEBV16, CAEBV18, CAEBV19, CAEBV2, CAEBV20, CAEBV21, CAEBV5, CAEBV6, CAEBV9

pos: gammaEBV4-100012, gammaEBV4-100015, gammaEBV4-100017, gammaEBV4-100032, gammaEBV4-100470, gammaEBV4-100929, gammaEBV4-101078, gammaEBV4-101090, gammaEBV4-101143, gammaEBV4-101244, gammaEBV4-101896, gammaEBV4-101996, gammaEBV4-102132, gammaEBV4-102279, gammaEBV4-102548, gammaEBV4-102833, gammaEBV4-102972, gammaEBV4-103167, gammaEBV4-103346, gammaEBV4-103649, gammaEBV4-103838, gammaEBV4-104080, gammaEBV4-104243, gammaEBV4-104986, gammaEBV4-104994, gammaEBV4-105325, gammaEBV4-105501, gammaEBV4-105542, gammaEBV4-105598

Row 11: gene = BBRF2

gene: BBRF2

sample: CAEBV11, CAEBV14, CAEBV18, CAEBV19, CAEBV2, CAEBV20, CAEBV21, CAEBV5, CAEBV6, CAEBV9

pos: gammaEBV4-101896, gammaEBV4-101996, gammaEBV4-102132, gammaEBV4-102279, gammaEBV4-102548, gammaEBV4-102833, gammaEBV4-102972, gammaEBV4-103167, gammaEBV4-103346, gammaEBV4-103649, gammaEBV4-103838, gammaEBV4-104080, gammaEBV4-104243, gammaEBV4-104986, gammaEBV4-104994, gammaEBV4-105325, gammaEBV4-105501, gammaEBV4-105542, gammaEBV4-105598, gammaEBV4-106045, gammaEBV4-106231, gammaEBV4-106388, gammaEBV4-106430

Row 12: gene = BBRF3

gene: BBRF3

sample: CAEBV11, CAEBV14, CAEBV2, CAEBV20, CAEBV21, CAEBV5, CAEBV6, CAEBV7, CAEBV9

pos: gammaEBV4-104986, gammaEBV4-104994, gammaEBV4-105325, gammaEBV4-105501, gammaEBV4-105542, gammaEBV4-105598, gammaEBV4-106045, gammaEBV4-106231, gammaEBV4-106388, gammaEBV4-106430, gammaEBV4-107019, gammaEBV4-107473, gammaEBV4-107738, gammaEBV4-108135, gammaEBV4-108164, gammaEBV4-108165, gammaEBV4-108219, gammaEBV4-108226, gammaEBV4-108228, gammaEBV4-109037, gammaEBV4-109074

Row 13: gene = BcLF1

gene: BcLF1

sample: CAEBV1, CAEBV10, CAEBV11, CAEBV11, CAEBV12, CAEBV13, CAEBV14, CAEBV17, CAEBV18, CAEBV19, CAEBV2, CAEBV20, CAEBV21, CAEBV22, CAEBV23, CAEBV3, CAEBV5, CAEBV6, CAEBV7, CAEBV8, CAEBV9

pos: gammaEBV4-119450, gammaEBV4-119719, gammaEBV4-119738, gammaEBV4-119764, gammaEBV4-119766, gammaEBV4-120040, gammaEBV4-120837, gammaEBV4-121112, gammaEBV4-121131, gammaEBV4-122449, gammaEBV4-122641, gammaEBV4-123006, gammaEBV4-123073, gammaEBV4-123123, gammaEBV4-123468, gammaEBV4-123550, gammaEBV4-123573, gammaEBV4-123594, gammaEBV4-123722, gammaEBV4-123726, gammaEBV4-123773, gammaEBV4-123786, gammaEBV4-123790, gammaEBV4-123855, gammaEBV4-124007, gammaEBV4-124331, gammaEBV4-124333, gammaEBV4-124335, gammaEBV4-124336, gammaEBV4-124337, gammaEBV4-124338, gammaEBV4-124339, gammaEBV4-124340, gammaEBV4-124342, gammaEBV4-124343, gammaEBV4-124344, gammaEBV4-124345, gammaEBV4-124346, gammaEBV4-124347, gammaEBV4-124348, gammaEBV4-124349, gammaEBV4-124350, gammaEBV4-124351, gammaEBV4-124352, gammaEBV4-124356, gammaEBV4-124357, gammaEBV4-124373, gammaEBV4-124681, gammaEBV4-124988, gammaEBV4-125072, gammaEBV4-125106, gammaEBV4-125211, gammaEBV4-125323, gammaEBV4-125330, gammaEBV4-125786, gammaEBV4-125876, gammaEBV4-125881, gammaEBV4-126135, gammaEBV4-126219, gammaEBV4-126402

Row 14: gene = BCRF1

gene: BCRF1

sample: CAEBV10, CAEBV11, CAEBV12, CAEBV13, CAEBV17, CAEBV18, CAEBV19, CAEBV2, CAEBV20, CAEBV23, CAEBV5, CAEBV6, CAEBV8

pos: gammaEBV4-10539, gammaEBV4-10920, gammaEBV4-11339, gammaEBV4-11955, gammaEBV4-7691, gammaEBV4-7705, gammaEBV4-8048, gammaEBV4-8244, gammaEBV4-8500, gammaEBV4-8555, gammaEBV4-8901, gammaEBV4-9007, gammaEBV4-9391, gammaEBV4-9426, gammaEBV4-9427, gammaEBV4-9428, gammaEBV4-9430, gammaEBV4-9432, gammaEBV4-9433, gammaEBV4-9434, gammaEBV4-9437, gammaEBV4-9438, gammaEBV4-9439, gammaEBV4-9442, gammaEBV4-9444, gammaEBV4-9446, gammaEBV4-9447, gammaEBV4-9518, gammaEBV4-9679, gammaEBV4-9712

Row 15: gene = BcRF1

gene: BcRF1

sample: CAEBV1, CAEBV10, CAEBV11, CAEBV12, CAEBV13, CAEBV14, CAEBV17, CAEBV18, CAEBV19, CAEBV2, CAEBV20, CAEBV21, CAEBV22, CAEBV23, CAEBV3, CAEBV5, CAEBV6, CAEBV7, CAEBV8, CAEBV9

pos: gammaEBV4-123468, gammaEBV4-123550, gammaEBV4-123573, gammaEBV4-123594, gammaEBV4-123722, gammaEBV4-123726, gammaEBV4-123773, gammaEBV4-123786, gammaEBV4-123790, gammaEBV4-123855, gammaEBV4-124007, gammaEBV4-124331, gammaEBV4-124333, gammaEBV4-124335, gammaEBV4-124336, gammaEBV4-124337, gammaEBV4-124338, gammaEBV4-124339, gammaEBV4-124340, gammaEBV4-124342, gammaEBV4-124343, gammaEBV4-124344, gammaEBV4-124345, gammaEBV4-124346, gammaEBV4-124347, gammaEBV4-124348, gammaEBV4-124349, gammaEBV4-124350, gammaEBV4-124351, gammaEBV4-124352, gammaEBV4-124356, gammaEBV4-124357, gammaEBV4-124373,

gammaEBV4-124681, gammaEBV4-124988, gammaEBV4-125072, gammaEBV4-125106, gammaEBV4-125211, gammaEBV4-125323, gammaEBV4-125330, gammaEBV4-125786, gammaEBV4-125876, gammaEBV4-125881, gammaEBV4-126135, gammaEBV4-126219, gammaEBV4-126402, gammaEBV4-127266, gammaEBV4-127347, gammaEBV4-127645, gammaEBV4-127663, gammaEBV4-127669, gammaEBV4-127827, gammaEBV4-128010, gammaEBV4-128770, gammaEBV4-128837, gammaEBV4-129213, gammaEBV4-129395

Row 16: gene = BDLF1

gene: BDLF1

sample: CAEBV14, CAEBV19, CAEBV2, CAEBV20, CAEBV5, CAEBV6, CAEBV9

pos: gammaEBV4-118378, gammaEBV4-118380, gammaEBV4-118437, gammaEBV4-118443, gammaEBV4-118497, gammaEBV4-118554, gammaEBV4-118559, gammaEBV4-119450, gammaEBV4-119719, gammaEBV4-119738, gammaEBV4-119764, gammaEBV4-119766, gammaEBV4-120040, gammaEBV4-120837, gammaEBV4-121112, gammaEBV4-121131, gammaEBV4-122449, gammaEBV4-122641, gammaEBV4-123006

Row 17: gene = BDLF2

gene: BDLF2

sample: CAEBV14, CAEBV19, CAEBV2, CAEBV20, CAEBV5, CAEBV6, CAEBV9

pos: gammaEBV4-117372, gammaEBV4-117462, gammaEBV4-117583, gammaEBV4-117880, gammaEBV4-117936, gammaEBV4-117941, gammaEBV4-118378, gammaEBV4-118380, gammaEBV4-118437, gammaEBV4-118443, gammaEBV4-118497, gammaEBV4-118554, gammaEBV4-118559, gammaEBV4-119450, gammaEBV4-119719, gammaEBV4-119738, gammaEBV4-119764, gammaEBV4-119766, gammaEBV4-120040, gammaEBV4-120837, gammaEBV4-121112, gammaEBV4-121131

Row 18: gene = BDLF3

gene: BDLF3

sample: CAEBV14, CAEBV19, CAEBV2, CAEBV20-1, CAEBV6, CAEBV9

pos: gammaEBV4-116738, gammaEBV4-117372, gammaEBV4-117462, gammaEBV4-117583, gammaEBV4-117880, gammaEBV4-117936, gammaEBV4-117941, gammaEBV4-118378, gammaEBV4-118380, gammaEBV4-118437, gammaEBV4-118443, gammaEBV4-118497, gammaEBV4-118554, gammaEBV4-118559, gammaEBV4-119450, gammaEBV4-119719, gammaEBV4-119738, gammaEBV4-119764, gammaEBV4-119766, gammaEBV4-120040

Row 19: gene = BDLF3.5

gene: BDLF3.5

sample: CAEBV11, CAEBV14, CAEBV18, CAEBV19, CAEBV2, CAEBV20, CAEBV6, CAEBV9

pos: gammaEBV4-114785, gammaEBV4-115055, gammaEBV4-115222, gammaEBV4-115343, gammaEBV4-115861, gammaEBV4-115891, gammaEBV4-115990, gammaEBV4-116738, gammaEBV4-117372, gammaEBV4-117462, gammaEBV4-117583, gammaEBV4-117880, gammaEBV4-117936, gammaEBV4-117941, gammaEBV4-118378, gammaEBV4-118380, gammaEBV4-118437, gammaEBV4-118443, gammaEBV4-118497, gammaEBV4-118554, gammaEBV4-118559

Row 20: gene = BdRF1

gene: BdRF1

sample: CAEBV11, CAEBV15, CAEBV18, CAEBV19, CAEBV2, CAEBV20, CAEBV4, CAEBV5, CAEBV6, CAEBV8, CAEBV9

pos: gammaEBV4-134460, gammaEBV4-134537, gammaEBV4-134765, gammaEBV4-134834, gammaEBV4-135676, gammaEBV4-135687, gammaEBV4-135692, gammaEBV4-135852, gammaEBV4-135865, gammaEBV4-136075, gammaEBV4-136560, gammaEBV4-136578, gammaEBV4-136653, gammaEBV4-136754, gammaEBV4-136896, gammaEBV4-136927, gammaEBV4-137017, gammaEBV4-137032, gammaEBV4-137120, gammaEBV4-137135, gammaEBV4-137142, gammaEBV4-137148, gammaEBV4-137151, gammaEBV4-137170, gammaEBV4-137227, gammaEBV4-137268, gammaEBV4-138016, gammaEBV4-138281, gammaEBV4-138618, gammaEBV4-138625, gammaEBV4-138872, gammaEBV4-138960, gammaEBV4-139071, gammaEBV4-139128, gammaEBV4-139154, gammaEBV4-139166, gammaEBV4-139196, gammaEBV4-139198, gammaEBV4-139202, gammaEBV4-139204, gammaEBV4-139207, gammaEBV4-139209, gammaEBV4-139214, gammaEBV4-139216, gammaEBV4-139438

Row 21: gene = BFLF1

gene: BFLF1

sample: CAEBV1, CAEBV11, CAEBV13, CAEBV19, CAEBV2, CAEBV21, CAEBV3, CAEBV5, CAEBV6, CAEBV7

pos: gammaEBV4-43009, gammaEBV4-43140, gammaEBV4-43141, gammaEBV4-43142, gammaEBV4-43145, gammaEBV4-43146, gammaEBV4-43373, gammaEBV4-43465, gammaEBV4-43477, gammaEBV4-43509, gammaEBV4-43624, gammaEBV4-43684, gammaEBV4-43981, gammaEBV4-44109, gammaEBV4-44155, gammaEBV4-44242, gammaEBV4-44658, gammaEBV4-45678, gammaEBV4-45790, gammaEBV4-45805, gammaEBV4-45860, gammaEBV4-46042, gammaEBV4-46062, gammaEBV4-46257, gammaEBV4-46472, gammaEBV4-46706, gammaEBV4-46912, gammaEBV4-47220, gammaEBV4-47236, gammaEBV4-47240, gammaEBV4-47908, gammaEBV4-47955, gammaEBV4-48227

Row 22: gene = BFLF2

gene: BFLF2

sample: CAEBV1, CAEBV11, CAEBV13, CAEBV19, CAEBV21, CAEBV3, CAEBV5, CAEBV6, CAEBV7

pos: gammaEBV4-41794, gammaEBV4-41821, gammaEBV4-43009, gammaEBV4-43140, gammaEBV4-43141, gammaEBV4-43142, gammaEBV4-43145, gammaEBV4-43146, gammaEBV4-43373, gammaEBV4-43465, gammaEBV4-43477, gammaEBV4-43509, gammaEBV4-43624, gammaEBV4-43684, gammaEBV4-43981, gammaEBV4-44109, gammaEBV4-44155, gammaEBV4-44242, gammaEBV4-44658, gammaEBV4-45678, gammaEBV4-45790, gammaEBV4-45805, gammaEBV4-45860, gammaEBV4-46042, gammaEBV4-46062, gammaEBV4-46257, gammaEBV4-46472

Row 23: gene = BFRF1

gene: BFRF1

sample: CAEBV10, CAEBV11, CAEBV13, CAEBV14, CAEBV19, CAEBV2, CAEBV20, CAEBV21, CAEBV5, CAEBV6, CAEBV7

pos: gammaEBV4-44658, gammaEBV4-45678, gammaEBV4-45790, gammaEBV4-45805, gammaEBV4-45860, gammaEBV4-46042, gammaEBV4-46062, gammaEBV4-46257, gammaEBV4-46472, gammaEBV4-46706, gammaEBV4-46912, gammaEBV4-47220, gammaEBV4-47236, gammaEBV4-47240, gammaEBV4-47908, gammaEBV4-47955, gammaEBV4-48227, gammaEBV4-48251, gammaEBV4-48335, gammaEBV4-48519, gammaEBV4-48797, gammaEBV4-48983, gammaEBV4-49102

Row 24: gene = BFRF1A

gene: BFRF1A

sample: CAEBV10, CAEBV11, CAEBV13, CAEBV19, CAEBV2, CAEBV21, CAEBV5, CAEBV6, CAEBV7

pos: gammaEBV4-44242, gammaEBV4-44658, gammaEBV4-45678, gammaEBV4-45790, gammaEBV4-45805, gammaEBV4-45860, gammaEBV4-46042, gammaEBV4-46062, gammaEBV4-46257, gammaEBV4-46472, gammaEBV4-46706, gammaEBV4-46912, gammaEBV4-47220, gammaEBV4-47236, gammaEBV4-47240, gammaEBV4-47908, gammaEBV4-47955, gammaEBV4-48227, gammaEBV4-48251, gammaEBV4-48335, gammaEBV4-48519

Row 25: gene = BFRF2

gene: BFRF2

sample: CAEBV10, CAEBV11, CAEBV13, CAEBV14, CAEBV19, CAEBV2, CAEBV20, CAEBV21, CAEBV5, CAEBV6, CAEBV7

pos: gammaEBV4-45678, gammaEBV4-45790, gammaEBV4-45805, gammaEBV4-45860, gammaEBV4-46042, gammaEBV4-46062, gammaEBV4-46257, gammaEBV4-46472, gammaEBV4-46706, gammaEBV4-46912, gammaEBV4-47220, gammaEBV4-47236, gammaEBV4-47240, gammaEBV4-47908, gammaEBV4-47955, gammaEBV4-48227, gammaEBV4-48251, gammaEBV4-48335, gammaEBV4-48519, gammaEBV4-48797, gammaEBV4-48983, gammaEBV4-49102, gammaEBV4-49719, gammaEBV4-50133, gammaEBV4-50242, gammaEBV4-51166

Row 26: gene = BGLF1

gene: BGLF1

sample: CAEBV11, CAEBV14, CAEBV18, CAEBV19, CAEBV2, CAEBV20, CAEBV5, CAEBV6, CAEBV9

pos: gammaEBV4-112741, gammaEBV4-113146, gammaEBV4-113587, gammaEBV4-113631, gammaEBV4-113886, gammaEBV4-113897, gammaEBV4-114033, gammaEBV4-114134, gammaEBV4-114178, gammaEBV4-114596, gammaEBV4-114785, gammaEBV4-115055, gammaEBV4-115222, gammaEBV4-115343, gammaEBV4-115861, gammaEBV4-115891, gammaEBV4-115990, gammaEBV4-116738, gammaEBV4-117372, gammaEBV4-117462, gammaEBV4-117583, gammaEBV4-117880, gammaEBV4-117936, gammaEBV4-117941

Row 27: gene = BGLF3

gene: BGLF3

sample: CAEBV11, CAEBV14, CAEBV18, CAEBV19, CAEBV2, CAEBV20, CAEBV5, CAEBV6, CAEBV7, CAEBV9

pos: gammaEBV4-110375, gammaEBV4-110393, gammaEBV4-110468, gammaEBV4-110538, gammaEBV4-110756, gammaEBV4-110937, gammaEBV4-111238, gammaEBV4-112229, gammaEBV4-112312, gammaEBV4-112741, gammaEBV4-113146, gammaEBV4-113587, gammaEBV4-113631, gammaEBV4-113886, gammaEBV4-113897, gammaEBV4-114033, gammaEBV4-114134, gammaEBV4-114178, gammaEBV4-114596

Row 28: gene = BGLF3.5

gene: BGLF3.5

sample: CAEBV11, CAEBV14, CAEBV2, CAEBV20, CAEBV5, CAEBV6, CAEBV7, CAEBV9

pos: gammaEBV4-110375, gammaEBV4-110393, gammaEBV4-110468, gammaEBV4-110538, gammaEBV4-110756, gammaEBV4-110937, gammaEBV4-111238, gammaEBV4-112229, gammaEBV4-112312, gammaEBV4-112741, gammaEBV4-113146, gammaEBV4-113587, gammaEBV4-113631

Row 29: gene = BGLF4

gene: BGLF4

sample: CAEBV11, CAEBV14, CAEBV2, CAEBV20, CAEBV5, CAEBV6, CAEBV7, CAEBV9

pos: gammaEBV4-108135, gammaEBV4-108164, gammaEBV4-108165, gammaEBV4-108219, gammaEBV4-108226, gammaEBV4-108228, gammaEBV4-109037, gammaEBV4-109074, gammaEBV4-110375, gammaEBV4-110393, gammaEBV4-110468, gammaEBV4-110538, gammaEBV4-110756, gammaEBV4-110937, gammaEBV4-111238, gammaEBV4-112229, gammaEBV4-112312, gammaEBV4-112741, gammaEBV4-113146

Row 30: gene = BGLF5

gene: BGLF5

sample: CAEBV11, CAEBV2, CAEBV20, CAEBV21, CAEBV6, CAEBV7, CAEBV9

pos: gammaEBV4-107019, gammaEBV4-107473, gammaEBV4-107738, gammaEBV4-108135, gammaEBV4-108164, gammaEBV4-108165, gammaEBV4-108219, gammaEBV4-108226, gammaEBV4-108228, gammaEBV4-109037, gammaEBV4-109074, gammaEBV4-110375, gammaEBV4-110393, gammaEBV4-110468, gammaEBV4-110538, gammaEBV4-110756, gammaEBV4-110937, gammaEBV4-111238

Row 31: gene = BGRF1/BDRF1

gene: BGRF1/BDRF1

sample: CAEBV11, CAEBV14, CAEBV18, CAEBV19, CAEBV2, CAEBV20, CAEBV5, CAEBV6, CAEBV7, CAEBV9

pos: gammaEBV4-110756, gammaEBV4-110937, gammaEBV4-111238, gammaEBV4-112229, gammaEBV4-112312, gammaEBV4-112741, gammaEBV4-113146, gammaEBV4-113587, gammaEBV4-113631, gammaEBV4-113886, gammaEBV4-113897, gammaEBV4-114033, gammaEBV4-114134, gammaEBV4-114178, gammaEBV4-114596, gammaEBV4-114785, gammaEBV4-115055, gammaEBV4-115222, gammaEBV4-115343, gammaEBV4-115861, gammaEBV4-115891, gammaEBV4-115990, gammaEBV4-116738, gammaEBV4-117372, gammaEBV4-117462, gammaEBV4-117583, gammaEBV4-117880, gammaEBV4-117936, gammaEBV4-117941, gammaEBV4-118378, gammaEBV4-118380, gammaEBV4-118437, gammaEBV4-118443, gammaEBV4-118497, gammaEBV4-118554, gammaEBV4-118559, gammaEBV4-119450, gammaEBV4-119719, gammaEBV4-119738, gammaEBV4-119764, gammaEBV4-119766, gammaEBV4-120040

Row 32: gene = BHLF1

gene: BHLF1

sample: CAEBV1, CAEBV10, CAEBV11, CAEBV12, CAEBV13, CAEBV14, CAEBV15, CAEBV16, CAEBV17, CAEBV18, CAEBV19, CAEBV2, CAEBV20, CAEBV21, CAEBV22, CAEBV23, CAEBV24, CAEBV3, CAEBV5, CAEBV6, CAEBV7, CAEBV8, CAEBV9

pos: gammaEBV4-36307, gammaEBV4-36315, gammaEBV4-36322, gammaEBV4-36355, gammaEBV4-36356, gammaEBV4-36360, gammaEBV4-36368, gammaEBV4-36389, gammaEBV4-36390, gammaEBV4-36391, gammaEBV4-36393, gammaEBV4-36394, gammaEBV4-36397, gammaEBV4-36399, gammaEBV4-36401, gammaEBV4-36402, gammaEBV4-36403, gammaEBV4-36405, gammaEBV4-36406, gammaEBV4-36408, gammaEBV4-36410, gammaEBV4-36411, gammaEBV4-36414, gammaEBV4-36415, gammaEBV4-36417, gammaEBV4-36420, gammaEBV4-36421, gammaEBV4-36423, gammaEBV4-36424, gammaEBV4-36425, gammaEBV4-36429, gammaEBV4-36432, gammaEBV4-36433, gammaEBV4-36435, gammaEBV4-36436, gammaEBV4-36437, gammaEBV4-36438, gammaEBV4-36439, gammaEBV4-36441, gammaEBV4-36442, gammaEBV4-36445, gammaEBV4-36446, gammaEBV4-36447, gammaEBV4-36448, gammaEBV4-36449, gammaEBV4-36450, gammaEBV4-36451, gammaEBV4-36453, gammaEBV4-36454, gammaEBV4-36455, gammaEBV4-36457, gammaEBV4-36458, gammaEBV4-36459, gammaEBV4-36460, gammaEBV4-36462, gammaEBV4-36463, gammaEBV4-36464, gammaEBV4-36465, gammaEBV4-36466, gammaEBV4-36468, gammaEBV4-36469, gammaEBV4-36470, gammaEBV4-36472, gammaEBV4-36475, gammaEBV4-36476, gammaEBV4-36477, gammaEBV4-36478, gammaEBV4-36479, gammaEBV4-36481, gammaEBV4-36482, gammaEBV4-36483, gammaEBV4-36486, gammaEBV4-36487, gammaEBV4-36489, gammaEBV4-36490, gammaEBV4-36491, gammaEBV4-36492, gammaEBV4-36495, gammaEBV4-36497, gammaEBV4-36498, gammaEBV4-36499, gammaEBV4-36501, gammaEBV4-36502, gammaEBV4-36507, gammaEBV4-36521, gammaEBV4-36528, gammaEBV4-36543, gammaEBV4-36558, gammaEBV4-36564, gammaEBV4-36710, gammaEBV4-36712, gammaEBV4-36745, gammaEBV4-36781, gammaEBV4-36801, gammaEBV4-36816, gammaEBV4-36834, gammaEBV4-36855, gammaEBV4-36856, gammaEBV4-36860, gammaEBV4-36865, gammaEBV4-36869, gammaEBV4-36882, gammaEBV4-36885, gammaEBV4-36897, gammaEBV4-36924, gammaEBV4-36940, gammaEBV4-36942, gammaEBV4-36946, gammaEBV4-36947, gammaEBV4-36961, gammaEBV4-36965, gammaEBV4-36993, gammaEBV4-37009, gammaEBV4-37019, gammaEBV4-37033, gammaEBV4-37044, gammaEBV4-37087, gammaEBV4-37113, gammaEBV4-37116, gammaEBV4-37130, gammaEBV4-37270, gammaEBV4-37490, gammaEBV4-37678, gammaEBV4-37924, gammaEBV4-37979, gammaEBV4-38989, gammaEBV4-39482, gammaEBV4-39873, gammaEBV4-39899, gammaEBV4-39903, gammaEBV4-39908, gammaEBV4-39915, gammaEBV4-39925, gammaEBV4-40194, gammaEBV4-40213, gammaEBV4-40219, gammaEBV4-40220, gammaEBV4-40224, gammaEBV4-40225, gammaEBV4-40263, gammaEBV4-40282, gammaEBV4-41499, gammaEBV4-41553, gammaEBV4-41648, gammaEBV4-41794, gammaEBV4-41821

Row 33: gene = BILF1

gene: BILF1

sample: CAEBV1, CAEBV11, CAEBV13, CAEBV17, CAEBV18, CAEBV19, CAEBV22, CAEBV5, CAEBV6, CAEBV8, CAEBV9

pos: gammaEBV4-149760, gammaEBV4-150546, gammaEBV4-150551, gammaEBV4-150702, gammaEBV4-150749, gammaEBV4-151271, gammaEBV4-151272, gammaEBV4-152545, gammaEBV4-152557, gammaEBV4-152613, gammaEBV4-153004, gammaEBV4-153077, gammaEBV4-153117, gammaEBV4-153180, gammaEBV4-153617, gammaEBV4-153639, gammaEBV4-153725, gammaEBV4-153763, gammaEBV4-153860, gammaEBV4-153864, gammaEBV4-153952, gammaEBV4-154055, gammaEBV4-154299, gammaEBV4-154429, gammaEBV4-154453

Row 34: gene = BKRF3

gene: BKRF3

sample: CAEBV1, CAEBV10, CAEBV11, CAEBV13, CAEBV14, CAEBV15, CAEBV16, CAEBV17, CAEBV18, CAEBV19, CAEBV2, CAEBV20, CAEBV21, CAEBV3, CAEBV5, CAEBV6, CAEBV7, CAEBV8, CAEBV9

pos: gammaEBV4-100012, gammaEBV4-100015, gammaEBV4-100017, gammaEBV4-100032, gammaEBV4-100470, gammaEBV4-96064, gammaEBV4-96065, gammaEBV4-96070, gammaEBV4-96073, gammaEBV4-96078, gammaEBV4-96096, gammaEBV4-96114, gammaEBV4-96117, gammaEBV4-96118, gammaEBV4-96123, gammaEBV4-96127, gammaEBV4-96128, gammaEBV4-96142, gammaEBV4-96181, gammaEBV4-96193, gammaEBV4-96197, gammaEBV4-96199, gammaEBV4-96203, gammaEBV4-96205, gammaEBV4-96207, gammaEBV4-96208, gammaEBV4-96240, gammaEBV4-96254, gammaEBV4-96259, gammaEBV4-96266, gammaEBV4-96276, gammaEBV4-96279, gammaEBV4-96280, gammaEBV4-96281, gammaEBV4-96289, gammaEBV4-96296, gammaEBV4-96298, gammaEBV4-96302, gammaEBV4-96306, gammaEBV4-96310, gammaEBV4-96313, gammaEBV4-96314, gammaEBV4-96315, gammaEBV4-96316, gammaEBV4-96319, gammaEBV4-96328, gammaEBV4-96333, gammaEBV4-96334, gammaEBV4-96337, gammaEBV4-96346, gammaEBV4-96349, gammaEBV4-96357, gammaEBV4-96364, gammaEBV4-96365, gammaEBV4-96367, gammaEBV4-96370, gammaEBV4-96373, gammaEBV4-96378, gammaEBV4-96379, gammaEBV4-96380, gammaEBV4-96382, gammaEBV4-96387, gammaEBV4-96388, gammaEBV4-96394, gammaEBV4-96396, gammaEBV4-96399, gammaEBV4-96400, gammaEBV4-96403, gammaEBV4-96405, gammaEBV4-96406, gammaEBV4-96410, gammaEBV4-96414, gammaEBV4-96415, gammaEBV4-96416, gammaEBV4-96417, gammaEBV4-96419, gammaEBV4-96422, gammaEBV4-96424, gammaEBV4-96425, gammaEBV4-96430, gammaEBV4-96436, gammaEBV4-96440, gammaEBV4-96442, gammaEBV4-96445, gammaEBV4-96446, gammaEBV4-96459, gammaEBV4-96462, gammaEBV4-96464, gammaEBV4-96466, gammaEBV4-96467, gammaEBV4-96468, gammaEBV4-96470, gammaEBV4-96472, gammaEBV4-96473, gammaEBV4-96481, gammaEBV4-96505, gammaEBV4-96521, gammaEBV4-96522, gammaEBV4-96542, gammaEBV4-96546, gammaEBV4-96554, gammaEBV4-96565, gammaEBV4-96566, gammaEBV4-96569, gammaEBV4-96575, gammaEBV4-96582, gammaEBV4-96586, gammaEBV4-96587, gammaEBV4-96588, gammaEBV4-96591, gammaEBV4-96592, gammaEBV4-96594, gammaEBV4-96595, gammaEBV4-96596, gammaEBV4-96598, gammaEBV4-96599, gammaEBV4-96601, gammaEBV4-96604, gammaEBV4-96607, gammaEBV4-96618, gammaEBV4-96622, gammaEBV4-96633, gammaEBV4-96634, gammaEBV4-96636, gammaEBV4-96637, gammaEBV4-96638, gammaEBV4-96640, gammaEBV4-96653, gammaEBV4-96675, gammaEBV4-96677, gammaEBV4-96685, gammaEBV4-96701, gammaEBV4-96703, gammaEBV4-96714, gammaEBV4-96719, gammaEBV4-96722, gammaEBV4-96796, gammaEBV4-97303, gammaEBV4-97310, gammaEBV4-97457, gammaEBV4-97485, gammaEBV4-97584, gammaEBV4-97632, gammaEBV4-98321, gammaEBV4-98485, gammaEBV4-98607, gammaEBV4-98649, gammaEBV4-98857, gammaEBV4-99257, gammaEBV4-99261, gammaEBV4-99275, gammaEBV4-99399, gammaEBV4-99402, gammaEBV4-99405, gammaEBV4-99422, gammaEBV4-99423, gammaEBV4-99440, gammaEBV4-99441, gammaEBV4-99535

Row 35: gene = BKRF4

gene: BKRF4

sample: CAEBV1, CAEBV11, CAEBV14, CAEBV16, CAEBV18, CAEBV19, CAEBV2, CAEBV20, CAEBV21, CAEBV5, CAEBV6, CAEBV8, CAEBV9

pos: gammaEBV4-100012, gammaEBV4-100015, gammaEBV4-100017, gammaEBV4-100032, gammaEBV4-100470, gammaEBV4-100929, gammaEBV4-101078, gammaEBV4-101090, gammaEBV4-101143, gammaEBV4-101244, gammaEBV4-97303, gammaEBV4-97310, gammaEBV4-97457, gammaEBV4-97485, gammaEBV4-97584, gammaEBV4-97632, gammaEBV4-98321, gammaEBV4-98485, gammaEBV4-98607, gammaEBV4-98649, gammaEBV4-98857, gammaEBV4-99257, gammaEBV4-99261, gammaEBV4-99275, gammaEBV4-99399, gammaEBV4-99402, gammaEBV4-99405, gammaEBV4-99422, gammaEBV4-99423, gammaEBV4-99440, gammaEBV4-99441, gammaEBV4-99535

Row 36: gene = BLLF1

gene: BLLF1

sample: CAEBV1, CAEBV11, CAEBV13, CAEBV15, CAEBV18, CAEBV19, CAEBV2, CAEBV20, CAEBV21, CAEBV5, CAEBV6, CAEBV8, CAEBV9

pos: gammaEBV4-75292, gammaEBV4-75357, gammaEBV4-75429, gammaEBV4-75555, gammaEBV4-75694, gammaEBV4-75699, gammaEBV4-76014, gammaEBV4-76449, gammaEBV4-76489, gammaEBV4-76525, gammaEBV4-76598, gammaEBV4-76943, gammaEBV4-77179, gammaEBV4-77304, gammaEBV4-77373, gammaEBV4-77401, gammaEBV4-77404, gammaEBV4-77419, gammaEBV4-77613, gammaEBV4-77647, gammaEBV4-77672, gammaEBV4-77826, gammaEBV4-78055, gammaEBV4-78268, gammaEBV4-78767, gammaEBV4-78969, gammaEBV4-79898, gammaEBV4-80010, gammaEBV4-80158, gammaEBV4-80180, gammaEBV4-80333, gammaEBV4-80380, gammaEBV4-80419, gammaEBV4-80444, gammaEBV4-81096, gammaEBV4-81378, gammaEBV4-81601, gammaEBV4-81671, gammaEBV4-81722, gammaEBV4-81794, gammaEBV4-81818

Row 37: gene = BLLF2

gene: BLLF2

sample: CAEBV1, CAEBV11, CAEBV18, CAEBV19, CAEBV2, CAEBV20, CAEBV21, CAEBV5, CAEBV6, CAEBV8, CAEBV9

pos: gammaEBV4-75292, gammaEBV4-75357, gammaEBV4-75429, gammaEBV4-75555, gammaEBV4-75694, gammaEBV4-75699, gammaEBV4-76014, gammaEBV4-76449, gammaEBV4-76489, gammaEBV4-76525, gammaEBV4-76598, gammaEBV4-76943, gammaEBV4-77179, gammaEBV4-77304, gammaEBV4-77373, gammaEBV4-77401, gammaEBV4-77404, gammaEBV4-77419, gammaEBV4-77613, gammaEBV4-77647, gammaEBV4-77672, gammaEBV4-77826, gammaEBV4-78055, gammaEBV4-78268, gammaEBV4-78767, gammaEBV4-78969

Row 38: gene = BLLF3

gene: BLLF3

sample: CAEBV1, CAEBV11, CAEBV13, CAEBV18, CAEBV19, CAEBV2, CAEBV20, CAEBV21, CAEBV5, CAEBV6, CAEBV8, CAEBV9

pos: gammaEBV4-73366, gammaEBV4-73378, gammaEBV4-73577, gammaEBV4-73713, gammaEBV4-73903, gammaEBV4-73989, gammaEBV4-74183, gammaEBV4-74250, gammaEBV4-74856, gammaEBV4-74882, gammaEBV4-75292, gammaEBV4-75357, gammaEBV4-75429, gammaEBV4-75555, gammaEBV4-75694, gammaEBV4-75699, gammaEBV4-76014, gammaEBV4-76449, gammaEBV4-76489, gammaEBV4-76525, gammaEBV4-76598, gammaEBV4-76943, gammaEBV4-77179, gammaEBV4-77304, gammaEBV4-77373, gammaEBV4-77401, gammaEBV4-77404, gammaEBV4-77419, gammaEBV4-77613, gammaEBV4-77647, gammaEBV4-77672, gammaEBV4-77826, gammaEBV4-78055

Row 39: gene = BLRF1

gene: BLRF1

sample: CAEBV1, CAEBV11, CAEBV18, CAEBV19, CAEBV2, CAEBV20, CAEBV21, CAEBV5, CAEBV6, CAEBV8, CAEBV9

pos: gammaEBV4-74856, gammaEBV4-74882, gammaEBV4-75292, gammaEBV4-75357, gammaEBV4-75429, gammaEBV4-75555, gammaEBV4-75694, gammaEBV4-75699, gammaEBV4-76014, gammaEBV4-76449, gammaEBV4-76489, gammaEBV4-76525, gammaEBV4-76598, gammaEBV4-76943, gammaEBV4-77179, gammaEBV4-77304, gammaEBV4-77373, gammaEBV4-77401, gammaEBV4-77404, gammaEBV4-77419, gammaEBV4-77613, gammaEBV4-77647, gammaEBV4-77672, gammaEBV4-77826, gammaEBV4-78055, gammaEBV4-78268

Row 40: gene = BMRF1

gene: BMRF1

sample: CAEBV1, CAEBV19, CAEBV2, CAEBV20, CAEBV5, CAEBV6, CAEBV7, CAEBV8, CAEBV9

pos: gammaEBV4-65685, gammaEBV4-65718, gammaEBV4-65785, gammaEBV4-65950, gammaEBV4-66061, gammaEBV4-66069, gammaEBV4-66363, gammaEBV4-66405, gammaEBV4-66612, gammaEBV4-66809, gammaEBV4-67279, gammaEBV4-67485, gammaEBV4-67505, gammaEBV4-67749, gammaEBV4-68057, gammaEBV4-68261, gammaEBV4-68279, gammaEBV4-68497, gammaEBV4-68583, gammaEBV4-68589, gammaEBV4-68612, gammaEBV4-68646, gammaEBV4-68657, gammaEBV4-68729, gammaEBV4-68748, gammaEBV4-69148, gammaEBV4-69192, gammaEBV4-69276, gammaEBV4-69611, gammaEBV4-69904, gammaEBV4-69950, gammaEBV4-70330, gammaEBV4-70538

Row 41: gene = BMRF2

gene: BMRF2

sample: CAEBV1, CAEBV11, CAEBV13, CAEBV19, CAEBV2, CAEBV20, CAEBV21, CAEBV5, CAEBV6, CAEBV7, CAEBV9

pos: gammaEBV4-67279, gammaEBV4-67485, gammaEBV4-67505, gammaEBV4-67749, gammaEBV4-68057, gammaEBV4-68261, gammaEBV4-68279, gammaEBV4-68497, gammaEBV4-68583, gammaEBV4-68589, gammaEBV4-68612, gammaEBV4-68646, gammaEBV4-68657, gammaEBV4-68729, gammaEBV4-68748, gammaEBV4-69148, gammaEBV4-69192, gammaEBV4-69276, gammaEBV4-69611, gammaEBV4-69904, gammaEBV4-69950, gammaEBV4-70330, gammaEBV4-70538, gammaEBV4-71139, gammaEBV4-71229, gammaEBV4-71292, gammaEBV4-71344, gammaEBV4-71546, gammaEBV4-71618, gammaEBV4-71671

Row 42: gene = BNRF1

gene: BNRF1

sample: CAEBV11, CAEBV13, CAEBV17, CAEBV18, CAEBV19, CAEBV2, CAEBV20, CAEBV21, CAEBV3, CAEBV5, CAEBV8, CAEBV9

pos: gammaEBV4-2403, gammaEBV4-2768, gammaEBV4-2769, gammaEBV4-2958, gammaEBV4-3452, gammaEBV4-3640, gammaEBV4-3720, gammaEBV4-3754, gammaEBV4-3758, gammaEBV4-3775, gammaEBV4-4082, gammaEBV4-4944, gammaEBV4-5080, gammaEBV4-5182, gammaEBV4-5406, gammaEBV4-5665, gammaEBV4-5739, gammaEBV4-5751, gammaEBV4-5758, gammaEBV4-5762, gammaEBV4-5793, gammaEBV4-5796, gammaEBV4-5798, gammaEBV4-5799, gammaEBV4-5802, gammaEBV4-5803, gammaEBV4-5804, gammaEBV4-5805, gammaEBV4-5807, gammaEBV4-5808, gammaEBV4-5809, gammaEBV4-5810, gammaEBV4-7019, gammaEBV4-7035, gammaEBV4-7210, gammaEBV4-7253, gammaEBV4-7691

Row 43: gene = BOLF1

gene: BOLF1

sample: CAEBV11, CAEBV13, CAEBV14, CAEBV17, CAEBV18, CAEBV19, CAEBV2, CAEBV20, CAEBV21, CAEBV23, CAEBV5, CAEBV6, CAEBV8, CAEBV9

pos: gammaEBV4-57429, gammaEBV4-57632, gammaEBV4-57708, gammaEBV4-57740, gammaEBV4-57806, gammaEBV4-57842, gammaEBV4-57869, gammaEBV4-57870, gammaEBV4-57879, gammaEBV4-57880, gammaEBV4-57896, gammaEBV4-57897, gammaEBV4-58047, gammaEBV4-58301, gammaEBV4-58407, gammaEBV4-58634, gammaEBV4-58692, gammaEBV4-58785, gammaEBV4-58867, gammaEBV4-59115, gammaEBV4-59448, gammaEBV4-59672, gammaEBV4-59799, gammaEBV4-59923, gammaEBV4-59992, gammaEBV4-60095, gammaEBV4-60374, gammaEBV4-60398, gammaEBV4-61987, gammaEBV4-62009, gammaEBV4-62182, gammaEBV4-62247, gammaEBV4-62391, gammaEBV4-62449, gammaEBV4-62499, gammaEBV4-62677, gammaEBV4-62690, gammaEBV4-62774, gammaEBV4-62778, gammaEBV4-62916, gammaEBV4-63356, gammaEBV4-63580, gammaEBV4-63692, gammaEBV4-64008, gammaEBV4-64199, gammaEBV4-64224, gammaEBV4-64324, gammaEBV4-64571, gammaEBV4-64795

Row 44: gene = BORF2

gene: BORF2

sample: CAEBV1, CAEBV11, CAEBV13, CAEBV19, CAEBV2, CAEBV20, CAEBV21, CAEBV5, CAEBV6, CAEBV7, CAEBV8, CAEBV9

pos: gammaEBV4-62182, gammaEBV4-62247, gammaEBV4-62391, gammaEBV4-62449, gammaEBV4-62499, gammaEBV4-62677, gammaEBV4-62690, gammaEBV4-62774, gammaEBV4-62778, gammaEBV4-62916, gammaEBV4-63356, gammaEBV4-63580, gammaEBV4-63692, gammaEBV4-64008, gammaEBV4-64199, gammaEBV4-64224, gammaEBV4-64324, gammaEBV4-64571, gammaEBV4-64795, gammaEBV4-64976, gammaEBV4-65127, gammaEBV4-65205, gammaEBV4-65433, gammaEBV4-65450, gammaEBV4-65685, gammaEBV4-65718, gammaEBV4-65785, gammaEBV4-65950, gammaEBV4-66061, gammaEBV4-66069, gammaEBV4-66363, gammaEBV4-66405, gammaEBV4-66612,

gammaEBV4-66809, gammaEBV4-67279, gammaEBV4-67485, gammaEBV4-67505, gammaEBV4-67749, gammaEBV4-68057, gammaEBV4-68261, gammaEBV4-68279, gammaEBV4-68497, gammaEBV4-68583, gammaEBV4-68589

Row 45: gene = BPLF1

gene: BPLF1

sample: CAEBV1, CAEBV10, CAEBV11, CAEBV13, CAEBV14, CAEBV17, CAEBV18, CAEBV19, CAEBV2, CAEBV20, CAEBV21, CAEBV23, CAEBV5, CAEBV6, CAEBV7, CAEBV8, CAEBV9

pos: gammaEBV4-47908, gammaEBV4-47955, gammaEBV4-48227, gammaEBV4-48251, gammaEBV4-48335, gammaEBV4-48519, gammaEBV4-48797, gammaEBV4-48983, gammaEBV4-49102, gammaEBV4-49719, gammaEBV4-50133, gammaEBV4-50242, gammaEBV4-51166, gammaEBV4-51500, gammaEBV4-51571, gammaEBV4-52091, gammaEBV4-52205, gammaEBV4-52211, gammaEBV4-52227, gammaEBV4-52259, gammaEBV4-52372, gammaEBV4-52814, gammaEBV4-52837, gammaEBV4-52952, gammaEBV4-53724, gammaEBV4-53902, gammaEBV4-54149, gammaEBV4-54243, gammaEBV4-54255, gammaEBV4-54491, gammaEBV4-54802, gammaEBV4-54921, gammaEBV4-55000, gammaEBV4-55730, gammaEBV4-55780, gammaEBV4-55793, gammaEBV4-55811, gammaEBV4-55869, gammaEBV4-55887, gammaEBV4-55914, gammaEBV4-56237, gammaEBV4-56566, gammaEBV4-56866, gammaEBV4-57429, gammaEBV4-57632, gammaEBV4-57708, gammaEBV4-57740, gammaEBV4-57806, gammaEBV4-57842, gammaEBV4-57869, gammaEBV4-57870, gammaEBV4-57879, gammaEBV4-57880, gammaEBV4-57896, gammaEBV4-57897, gammaEBV4-58047, gammaEBV4-58301, gammaEBV4-58407, gammaEBV4-58634, gammaEBV4-58692, gammaEBV4-58785, gammaEBV4-58867, gammaEBV4-59115, gammaEBV4-59448, gammaEBV4-59672, gammaEBV4-59799, gammaEBV4-59923, gammaEBV4-59992, gammaEBV4-60095, gammaEBV4-60374, gammaEBV4-60398

Row 46: gene = BRLF1

gene: BRLF1

sample: CAEBV1, CAEBV10, CAEBV11, CAEBV13, CAEBV14, CAEBV16, CAEBV17, CAEBV18, CAEBV19, CAEBV2, CAEBV20, CAEBV21, CAEBV23, CAEBV24, CAEBV3, CAEBV5, CAEBV6, CAEBV7, CAEBV8, CAEBV9

pos: gammaEBV4-87870, gammaEBV4-87967, gammaEBV4-88158, gammaEBV4-88365, gammaEBV4-88383, gammaEBV4-88385, gammaEBV4-88388, gammaEBV4-88390, gammaEBV4-88393, gammaEBV4-88397, gammaEBV4-88400, gammaEBV4-88401, gammaEBV4-88402, gammaEBV4-88405, gammaEBV4-88409, gammaEBV4-88410, gammaEBV4-88411, gammaEBV4-88412, gammaEBV4-88413, gammaEBV4-88414, gammaEBV4-88415, gammaEBV4-88416, gammaEBV4-88417, gammaEBV4-88419, gammaEBV4-88420, gammaEBV4-88421, gammaEBV4-88423, gammaEBV4-88424, gammaEBV4-88425, gammaEBV4-88426, gammaEBV4-88427, gammaEBV4-88429, gammaEBV4-88431, gammaEBV4-88432, gammaEBV4-88433, gammaEBV4-88435, gammaEBV4-88436, gammaEBV4-88437, gammaEBV4-88438, gammaEBV4-88439, gammaEBV4-88440, gammaEBV4-88442, gammaEBV4-88444, gammaEBV4-88445, gammaEBV4-88446, gammaEBV4-88447, gammaEBV4-88448, gammaEBV4-88449, gammaEBV4-88450, gammaEBV4-88451, gammaEBV4-88452, gammaEBV4-88453, gammaEBV4-88454, gammaEBV4-88455, gammaEBV4-88456, gammaEBV4-88457, gammaEBV4-88458, gammaEBV4-88459, gammaEBV4-88462, gammaEBV4-88463, gammaEBV4-88464, gammaEBV4-88465, gammaEBV4-88466, gammaEBV4-88467, gammaEBV4-88469, gammaEBV4-88470, gammaEBV4-88471, gammaEBV4-88472, gammaEBV4-88474, gammaEBV4-88476, gammaEBV4-88478, gammaEBV4-88483, gammaEBV4-88486, gammaEBV4-88495, gammaEBV4-88497, gammaEBV4-88498, gammaEBV4-88499, gammaEBV4-88500, gammaEBV4-88501, gammaEBV4-88502, gammaEBV4-88503, gammaEBV4-88504, gammaEBV4-88506, gammaEBV4-88507, gammaEBV4-88508, gammaEBV4-88511, gammaEBV4-88512, gammaEBV4-88514, gammaEBV4-88516, gammaEBV4-88527, gammaEBV4-88528, gammaEBV4-88529, gammaEBV4-88597, gammaEBV4-88834, gammaEBV4-89231, gammaEBV4-89456, gammaEBV4-90140, gammaEBV4-90282, gammaEBV4-90294, gammaEBV4-90298, gammaEBV4-90302, gammaEBV4-90304, gammaEBV4-90308, gammaEBV4-90311, gammaEBV4-90312, gammaEBV4-90313, gammaEBV4-90315, gammaEBV4-90317, gammaEBV4-90319, gammaEBV4-90320, gammaEBV4-90321, gammaEBV4-90322, gammaEBV4-90324, gammaEBV4-90332, gammaEBV4-90339, gammaEBV4-90340, gammaEBV4-90341, gammaEBV4-90342, gammaEBV4-90343, gammaEBV4-90347, gammaEBV4-90348, gammaEBV4-90349, gammaEBV4-90350, gammaEBV4-90351, gammaEBV4-90352, gammaEBV4-90353, gammaEBV4-90354, gammaEBV4-90355, gammaEBV4-90356, gammaEBV4-90357, gammaEBV4-90358, gammaEBV4-90359, gammaEBV4-90361, gammaEBV4-90370, gammaEBV4-90371, gammaEBV4-90372, gammaEBV4-90373, gammaEBV4-90376, gammaEBV4-90378, gammaEBV4-91150, gammaEBV4-91422, gammaEBV4-91471, gammaEBV4-91480, gammaEBV4-91705, gammaEBV4-93050, gammaEBV4-93159, gammaEBV4-93490, gammaEBV4-94085, gammaEBV4-94177, gammaEBV4-94212, gammaEBV4-94589, gammaEBV4-95186, gammaEBV4-95199, gammaEBV4-95240, gammaEBV4-95258, gammaEBV4-95261, gammaEBV4-95588, gammaEBV4-95590, gammaEBV4-95666

Row 47: gene = BRRF1

gene: BRRF1

sample: CAEBV11, CAEBV13, CAEBV14, CAEBV16, CAEBV19, CAEBV2, CAEBV20, CAEBV3, CAEBV5, CAEBV6, CAEBV8, CAEBV9

pos: gammaEBV4-91150, gammaEBV4-91422, gammaEBV4-91471, gammaEBV4-91480, gammaEBV4-91705, gammaEBV4-93050, gammaEBV4-93159, gammaEBV4-93490, gammaEBV4-94085, gammaEBV4-94177, gammaEBV4-94212, gammaEBV4-94589, gammaEBV4-95186, gammaEBV4-95199, gammaEBV4-95240, gammaEBV4-95258, gammaEBV4-95261, gammaEBV4-95588, gammaEBV4-95590, gammaEBV4-95666

Row 48: gene = BRRF2

gene: BRRF2

sample: CAEBV1, CAEBV10, CAEBV11, CAEBV13, CAEBV14, CAEBV15, CAEBV16, CAEBV17, CAEBV18, CAEBV19, CAEBV2, CAEBV20, CAEBV21, CAEBV3, CAEBV5, CAEBV6, CAEBV7, CAEBV8, CAEBV9

pos: gammaEBV4-93050, gammaEBV4-93159, gammaEBV4-93490, gammaEBV4-94085, gammaEBV4-94177, gammaEBV4-94212, gammaEBV4-94589, gammaEBV4-95186, gammaEBV4-95199, gammaEBV4-95240, gammaEBV4-95258, gammaEBV4-95261, gammaEBV4-95588, gammaEBV4-95590, gammaEBV4-95666, gammaEBV4-95906, gammaEBV4-95942, gammaEBV4-95961, gammaEBV4-95963, gammaEBV4-95969, gammaEBV4-95974, gammaEBV4-95983, gammaEBV4-95984, gammaEBV4-95999, gammaEBV4-96000, gammaEBV4-96003, gammaEBV4-96004, gammaEBV4-96006, gammaEBV4-96008, gammaEBV4-96010, gammaEBV4-96024, gammaEBV4-96029, gammaEBV4-96038, gammaEBV4-96055, gammaEBV4-96064, gammaEBV4-96065, gammaEBV4-96070, gammaEBV4-96073, gammaEBV4-96078, gammaEBV4-96096, gammaEBV4-96114, gammaEBV4-96117, gammaEBV4-96118, gammaEBV4-96123, gammaEBV4-96127, gammaEBV4-96128, gammaEBV4-96142, gammaEBV4-96181, gammaEBV4-96193, gammaEBV4-96197, gammaEBV4-96199, gammaEBV4-96203, gammaEBV4-96205, gammaEBV4-96207, gammaEBV4-96208, gammaEBV4-96240, gammaEBV4-96254, gammaEBV4-96259, gammaEBV4-96266, gammaEBV4-96276, gammaEBV4-96279, gammaEBV4-96280, gammaEBV4-96281, gammaEBV4-96289, gammaEBV4-96296, gammaEBV4-96298, gammaEBV4-96302, gammaEBV4-96306, gammaEBV4-96310, gammaEBV4-96313, gammaEBV4-96314, gammaEBV4-96315, gammaEBV4-96316, gammaEBV4-96319, gammaEBV4-96328, gammaEBV4-96333, gammaEBV4-96334, gammaEBV4-96337, gammaEBV4-96346, gammaEBV4-96349, gammaEBV4-96357, gammaEBV4-96364, gammaEBV4-96365, gammaEBV4-96367, gammaEBV4-96370, gammaEBV4-96373, gammaEBV4-96378, gammaEBV4-96379, gammaEBV4-96380, gammaEBV4-96382, gammaEBV4-96387, gammaEBV4-96388, gammaEBV4-96394, gammaEBV4-96396, gammaEBV4-96399, gammaEBV4-96400, gammaEBV4-96403, gammaEBV4-96405, gammaEBV4-96406, gammaEBV4-96410, gammaEBV4-96414, gammaEBV4-96415, gammaEBV4-96416, gammaEBV4-96417, gammaEBV4-96419, gammaEBV4-96422, gammaEBV4-96424, gammaEBV4-96425, gammaEBV4-96430, gammaEBV4-96436, gammaEBV4-96440, gammaEBV4-96442, gammaEBV4-96445, gammaEBV4-96446, gammaEBV4-96459, gammaEBV4-96462, gammaEBV4-96464, gammaEBV4-96466, gammaEBV4-96467, gammaEBV4-96468, gammaEBV4-96470, gammaEBV4-96472, gammaEBV4-96473, gammaEBV4-96481, gammaEBV4-96505, gammaEBV4-96521, gammaEBV4-96522, gammaEBV4-96542, gammaEBV4-96546, gammaEBV4-96554, gammaEBV4-96565, gammaEBV4-96566, gammaEBV4-96569, gammaEBV4-96575, gammaEBV4-96582, gammaEBV4-96586, gammaEBV4-96587, gammaEBV4-96588, gammaEBV4-96591, gammaEBV4-96592, gammaEBV4-96594, gammaEBV4-96595, gammaEBV4-96596, gammaEBV4-96598, gammaEBV4-96599, gammaEBV4-96601, gammaEBV4-96604, gammaEBV4-96607, gammaEBV4-96618, gammaEBV4-96622, gammaEBV4-96633, gammaEBV4-96634, gammaEBV4-96636, gammaEBV4-96637, gammaEBV4-96638, gammaEBV4-96640, gammaEBV4-96653, gammaEBV4-96675, gammaEBV4-96677, gammaEBV4-96685, gammaEBV4-96701, gammaEBV4-96703, gammaEBV4-96714, gammaEBV4-96719, gammaEBV4-96722, gammaEBV4-96796, gammaEBV4-97303, gammaEBV4-97310, gammaEBV4-97457, gammaEBV4-97485, gammaEBV4-97584

Row 49: gene = BSLF1

gene: BSLF1

sample: CAEBV1, CAEBV11, CAEBV13, CAEBV18, CAEBV19, CAEBV2, CAEBV20, CAEBV21, CAEBV5, CAEBV6, CAEBV9

pos: gammaEBV4-70330, gammaEBV4-70538, gammaEBV4-71139, gammaEBV4-71229, gammaEBV4-71292, gammaEBV4-71344, gammaEBV4-71546, gammaEBV4-71618, gammaEBV4-71671, gammaEBV4-72053, gammaEBV4-72289, gammaEBV4-72560, gammaEBV4-72580, gammaEBV4-72690, gammaEBV4-72723, gammaEBV4-72979, gammaEBV4-73029, gammaEBV4-73305, gammaEBV4-73366, gammaEBV4-73378, gammaEBV4-73577, gammaEBV4-73713, gammaEBV4-73903, gammaEBV4-73989, gammaEBV4-74183, gammaEBV4-74250, gammaEBV4-74856, gammaEBV4-74882, gammaEBV4-75292, gammaEBV4-75357, gammaEBV4-75429, gammaEBV4-75555, gammaEBV4-75694, gammaEBV4-75699, gammaEBV4-76014, gammaEBV4-76449, gammaEBV4-76489, gammaEBV4-76525

Row 50: gene = BSLF2/BMLF1

gene: BSLF2/BMLF1

sample: CAEBV11, CAEBV13, CAEBV19, CAEBV2, CAEBV20, CAEBV21, CAEBV5, CAEBV6, CAEBV7, CAEBV9

pos: gammaEBV4-68497, gammaEBV4-68583, gammaEBV4-68589, gammaEBV4-68612, gammaEBV4-68646, gammaEBV4-68657, gammaEBV4-68729, gammaEBV4-68748, gammaEBV4-69148, gammaEBV4-69192, gammaEBV4-69276, gammaEBV4-69611, gammaEBV4-69904, gammaEBV4-69950, gammaEBV4-70330, gammaEBV4-70538, gammaEBV4-71139, gammaEBV4-71229, gammaEBV4-71292, gammaEBV4-71344, gammaEBV4-71546, gammaEBV4-71618, gammaEBV4-71671, gammaEBV4-72053, gammaEBV4-72289, gammaEBV4-72560, gammaEBV4-72580, gammaEBV4-72690, gammaEBV4-72723, gammaEBV4-72979, gammaEBV4-73029, gammaEBV4-73305, gammaEBV4-73366, gammaEBV4-73378, gammaEBV4-73577, gammaEBV4-73713, gammaEBV4-73903, gammaEBV4-73989

Row 51: gene = BSRF1

gene: BSRF1

sample: CAEBV1, CAEBV11, CAEBV13, CAEBV18, CAEBV19, CAEBV2, CAEBV20, CAEBV21, CAEBV5, CAEBV6, CAEBV8, CAEBV9

pos: gammaEBV4-72690, gammaEBV4-72723, gammaEBV4-72979, gammaEBV4-73029, gammaEBV4-73305, gammaEBV4-73366, gammaEBV4-73378, gammaEBV4-73577, gammaEBV4-73713, gammaEBV4-73903, gammaEBV4-73989, gammaEBV4-74183, gammaEBV4-74250, gammaEBV4-74856, gammaEBV4-74882, gammaEBV4-75292, gammaEBV4-75357, gammaEBV4-75429, gammaEBV4-75555, gammaEBV4-75694, gammaEBV4-75699, gammaEBV4-76014, gammaEBV4-76449, gammaEBV4-76489, gammaEBV4-76525, gammaEBV4-76598, gammaEBV4-76943, gammaEBV4-77179

Row 52: gene = BVRF1

gene: BVRF1

sample: CAEBV10, CAEBV11, CAEBV15, CAEBV18, CAEBV19, CAEBV2, CAEBV20, CAEBV5, CAEBV6, CAEBV9

pos: gammaEBV4-131300, gammaEBV4-131367, gammaEBV4-131675, gammaEBV4-132155, gammaEBV4-132194, gammaEBV4-132223, gammaEBV4-132224, gammaEBV4-132232, gammaEBV4-132437, gammaEBV4-133088, gammaEBV4-133974, gammaEBV4-134311, gammaEBV4-134348, gammaEBV4-134460, gammaEBV4-134537, gammaEBV4-134765, gammaEBV4-134834, gammaEBV4-135676, gammaEBV4-135687, gammaEBV4-135692, gammaEBV4-135852, gammaEBV4-135865, gammaEBV4-136075, gammaEBV4-136560, gammaEBV4-136578, gammaEBV4-136653, gammaEBV4-136754

Row 53: gene = BVRF2

gene: BVRF2

sample: CAEBV11, CAEBV15, CAEBV18, CAEBV19, CAEBV2, CAEBV20, CAEBV4, CAEBV5, CAEBV6, CAEBV8, CAEBV9

pos: gammaEBV4-133974, gammaEBV4-134311, gammaEBV4-134348, gammaEBV4-134460, gammaEBV4-134537, gammaEBV4-134765, gammaEBV4-134834, gammaEBV4-135676, gammaEBV4-135687, gammaEBV4-135692, gammaEBV4-135852, gammaEBV4-135865, gammaEBV4-136075, gammaEBV4-136560, gammaEBV4-136578, gammaEBV4-136653, gammaEBV4-136754, gammaEBV4-136896, gammaEBV4-136927, gammaEBV4-137017, gammaEBV4-137032, gammaEBV4-137120, gammaEBV4-137135, gammaEBV4-137142, gammaEBV4-137148, gammaEBV4-137151, gammaEBV4-137170, gammaEBV4-137227, gammaEBV4-137268, gammaEBV4-138016, gammaEBV4-138281, gammaEBV4-138618, gammaEBV4-138625, gammaEBV4-138872, gammaEBV4-138960, gammaEBV4-139071, gammaEBV4-139128, gammaEBV4-139154, gammaEBV4-139166, gammaEBV4-139196, gammaEBV4-139198, gammaEBV4-139202, gammaEBV4-139204, gammaEBV4-139207, gammaEBV4-139209, gammaEBV4-139214, gammaEBV4-139216, gammaEBV4-139438

Row 54: gene = BXLF1

gene: BXLF1

sample: CAEBV1, CAEBV10, CAEBV11, CAEBV14, CAEBV15, CAEBV18, CAEBV19, CAEBV2, CAEBV20-1, CAEBV20-2, CAEBV5, CAEBV6, CAEBV8, CAEBV9

pos: gammaEBV4-128770, gammaEBV4-128837, gammaEBV4-129213, gammaEBV4-129395, gammaEBV4-130293, gammaEBV4-130302, gammaEBV4-130543, gammaEBV4-130568, gammaEBV4-130569, gammaEBV4-130696, gammaEBV4-130723, gammaEBV4-130930, gammaEBV4-131300, gammaEBV4-131367, gammaEBV4-131675, gammaEBV4-132155, gammaEBV4-132194, gammaEBV4-132223, gammaEBV4-132224, gammaEBV4-132232, gammaEBV4-132437, gammaEBV4-133088, gammaEBV4-133974, gammaEBV4-134311, gammaEBV4-134348, gammaEBV4-134460, gammaEBV4-134537

Row 55: gene = BXLF2

gene: BXLF2

sample: CAEBV1, CAEBV10, CAEBV11, CAEBV14, CAEBV15, CAEBV18, CAEBV19, CAEBV2, CAEBV20, CAEBV5, CAEBV6, CAEBV8, CAEBV9

pos: gammaEBV4-127266, gammaEBV4-127347, gammaEBV4-127645, gammaEBV4-127663, gammaEBV4-127669, gammaEBV4-127827, gammaEBV4-128010, gammaEBV4-128770, gammaEBV4-128837, gammaEBV4-129213, gammaEBV4-129395, gammaEBV4-130293, gammaEBV4-130302, gammaEBV4-130543, gammaEBV4-130568, gammaEBV4-130569, gammaEBV4-130696, gammaEBV4-130723, gammaEBV4-130930, gammaEBV4-131300, gammaEBV4-131367, gammaEBV4-131675, gammaEBV4-132155, gammaEBV4-132194, gammaEBV4-132223, gammaEBV4-132224, gammaEBV4-132232, gammaEBV4-132437

Row 56: gene = BZLF1

gene: BZLF1

sample: CAEBV1, CAEBV10, CAEBV11, CAEBV13, CAEBV16, CAEBV17, CAEBV18, CAEBV19, CAEBV2, CAEBV20, CAEBV21, CAEBV23, CAEBV24, CAEBV3, CAEBV5, CAEBV6, CAEBV7, CAEBV8, CAEBV9

pos: gammaEBV4-87870, gammaEBV4-87967, gammaEBV4-88158, gammaEBV4-88365, gammaEBV4-88383, gammaEBV4-88385, gammaEBV4-88388, gammaEBV4-88390, gammaEBV4-88393, gammaEBV4-88397, gammaEBV4-88400, gammaEBV4-88401, gammaEBV4-88402, gammaEBV4-88405, gammaEBV4-88409, gammaEBV4-88410, gammaEBV4-88411, gammaEBV4-88412, gammaEBV4-88413, gammaEBV4-88414, gammaEBV4-88415, gammaEBV4-88416, gammaEBV4-88417, gammaEBV4-88419, gammaEBV4-88420, gammaEBV4-88421, gammaEBV4-88423, gammaEBV4-88424, gammaEBV4-88425, gammaEBV4-88426, gammaEBV4-88427, gammaEBV4-88429, gammaEBV4-88431, gammaEBV4-88432, gammaEBV4-88433, gammaEBV4-88435, gammaEBV4-88436, gammaEBV4-88437, gammaEBV4-88438, gammaEBV4-88439, gammaEBV4-88440, gammaEBV4-88442, gammaEBV4-88444, gammaEBV4-88445,

gammaEBV4-88446, gammaEBV4-88447, gammaEBV4-88448, gammaEBV4-88449, gammaEBV4-88450, gammaEBV4-88451, gammaEBV4-88452, gammaEBV4-88453, gammaEBV4-88454, gammaEBV4-88455, gammaEBV4-88456, gammaEBV4-88457, gammaEBV4-88458, gammaEBV4-88459, gammaEBV4-88462, gammaEBV4-88463, gammaEBV4-88464, gammaEBV4-88465, gammaEBV4-88466, gammaEBV4-88467, gammaEBV4-88469, gammaEBV4-88470, gammaEBV4-88471, gammaEBV4-88472, gammaEBV4-88474, gammaEBV4-88476, gammaEBV4-88478, gammaEBV4-88483, gammaEBV4-88486, gammaEBV4-88495, gammaEBV4-88497, gammaEBV4-88498, gammaEBV4-88499, gammaEBV4-88500, gammaEBV4-88501, gammaEBV4-88502, gammaEBV4-88503, gammaEBV4-88504, gammaEBV4-88506, gammaEBV4-88507, gammaEBV4-88508, gammaEBV4-88511, gammaEBV4-88512, gammaEBV4-88514, gammaEBV4-88516, gammaEBV4-88527, gammaEBV4-88528, gammaEBV4-88529, gammaEBV4-88597, gammaEBV4-88834, gammaEBV4-89231, gammaEBV4-89456, gammaEBV4-90140, gammaEBV4-90282, gammaEBV4-90294, gammaEBV4-90298, gammaEBV4-90302, gammaEBV4-90304, gammaEBV4-90308, gammaEBV4-90311, gammaEBV4-90312, gammaEBV4-90313, gammaEBV4-90315, gammaEBV4-90317, gammaEBV4-90319, gammaEBV4-90320, gammaEBV4-90321, gammaEBV4-90322, gammaEBV4-90324, gammaEBV4-90332, gammaEBV4-90339, gammaEBV4-90340, gammaEBV4-90341, gammaEBV4-90342, gammaEBV4-90343, gammaEBV4-90347, gammaEBV4-90348, gammaEBV4-90349, gammaEBV4-90350, gammaEBV4-90351, gammaEBV4-90352, gammaEBV4-90353, gammaEBV4-90354, gammaEBV4-90355, gammaEBV4-90356, gammaEBV4-90357, gammaEBV4-90358, gammaEBV4-90359, gammaEBV4-90361, gammaEBV4-90370, gammaEBV4-90371, gammaEBV4-90372, gammaEBV4-90373, gammaEBV4-90376, gammaEBV4-90378, gammaEBV4-91150, gammaEBV4-91422, gammaEBV4-91471, gammaEBV4-91480, gammaEBV4-91705

Row 57: gene = EBNA-1

gene: EBNA-1

sample: CAEBV1, CAEBV10, CAEBV11, CAEBV13, CAEBV14, CAEBV15, CAEBV16, CAEBV17, CAEBV18, CAEBV19, CAEBV2, CAEBV20, CAEBV21, CAEBV23, CAEBV24, CAEBV3, CAEBV5, CAEBV6, CAEBV7, CAEBV8, CAEBV9

pos: gammaEBV4-48227, gammaEBV4-48251, gammaEBV4-48335, gammaEBV4-48519, gammaEBV4-48797, gammaEBV4-48983, gammaEBV4-49102, gammaEBV4-49719, gammaEBV4-50133, gammaEBV4-50242, gammaEBV4-51166, gammaEBV4-51500, gammaEBV4-51571, gammaEBV4-52091, gammaEBV4-52205, gammaEBV4-52211, gammaEBV4-52227, gammaEBV4-52259, gammaEBV4-52372, gammaEBV4-52814, gammaEBV4-52837, gammaEBV4-52952, gammaEBV4-53724, gammaEBV4-53902, gammaEBV4-54149, gammaEBV4-54243, gammaEBV4-54255, gammaEBV4-54491, gammaEBV4-54802, gammaEBV4-54921, gammaEBV4-55000, gammaEBV4-55730, gammaEBV4-55780, gammaEBV4-55793, gammaEBV4-55811, gammaEBV4-55869, gammaEBV4-55887, gammaEBV4-55914, gammaEBV4-56237, gammaEBV4-56566, gammaEBV4-56866, gammaEBV4-57429, gammaEBV4-57632, gammaEBV4-57708, gammaEBV4-57740, gammaEBV4-57806, gammaEBV4-57842, gammaEBV4-57869, gammaEBV4-57870, gammaEBV4-57879, gammaEBV4-57880, gammaEBV4-57896, gammaEBV4-57897, gammaEBV4-58047, gammaEBV4-58301, gammaEBV4-58407, gammaEBV4-58634, gammaEBV4-58692, gammaEBV4-58785, gammaEBV4-58867, gammaEBV4-59115, gammaEBV4-59448, gammaEBV4-59672, gammaEBV4-59799, gammaEBV4-59923, gammaEBV4-59992, gammaEBV4-60095, gammaEBV4-60374, gammaEBV4-60398, gammaEBV4-61987, gammaEBV4-62009, gammaEBV4-62182, gammaEBV4-62247, gammaEBV4-62391, gammaEBV4-62449, gammaEBV4-62499, gammaEBV4-62677, gammaEBV4-62690, gammaEBV4-62774, gammaEBV4-62778, gammaEBV4-62916, gammaEBV4-63356, gammaEBV4-63580, gammaEBV4-63692, gammaEBV4-64008, gammaEBV4-64199, gammaEBV4-64224, gammaEBV4-64324, gammaEBV4-64571, gammaEBV4-64795, gammaEBV4-64976, gammaEBV4-65127, gammaEBV4-65205, gammaEBV4-65433, gammaEBV4-65450, gammaEBV4-65685, gammaEBV4-65718, gammaEBV4-65785, gammaEBV4-65950, gammaEBV4-66061, gammaEBV4-66069, gammaEBV4-66363, gammaEBV4-66405, gammaEBV4-66612, gammaEBV4-66809, gammaEBV4-67279, gammaEBV4-67485, gammaEBV4-67505, gammaEBV4-67749, gammaEBV4-68057, gammaEBV4-68261, gammaEBV4-68279, gammaEBV4-68497, gammaEBV4-68583, gammaEBV4-68589, gammaEBV4-68612, gammaEBV4-68646, gammaEBV4-68657, gammaEBV4-68729, gammaEBV4-68748, gammaEBV4-69148, gammaEBV4-69192, gammaEBV4-69276, gammaEBV4-69611, gammaEBV4-69904, gammaEBV4-69950, gammaEBV4-70330, gammaEBV4-70538, gammaEBV4-71139, gammaEBV4-71229, gammaEBV4-71292, gammaEBV4-71344, gammaEBV4-71546, gammaEBV4-71618, gammaEBV4-71671, gammaEBV4-72053, gammaEBV4-72289, gammaEBV4-72560, gammaEBV4-72580, gammaEBV4-72690, gammaEBV4-72723, gammaEBV4-72979, gammaEBV4-73029, gammaEBV4-73305, gammaEBV4-73366, gammaEBV4-73378, gammaEBV4-73577, gammaEBV4-73713, gammaEBV4-73903, gammaEBV4-73989, gammaEBV4-74183, gammaEBV4-74250, gammaEBV4-74856, gammaEBV4-74882, gammaEBV4-75292, gammaEBV4-75357, gammaEBV4-75429, gammaEBV4-75555, gammaEBV4-75694, gammaEBV4-75699, gammaEBV4-76014, gammaEBV4-76449, gammaEBV4-76489, gammaEBV4-76525, gammaEBV4-76598, gammaEBV4-76943, gammaEBV4-77179, gammaEBV4-77304, gammaEBV4-77373, gammaEBV4-77401, gammaEBV4-77404, gammaEBV4-77419, gammaEBV4-77613, gammaEBV4-77647, gammaEBV4-77672, gammaEBV4-77826, gammaEBV4-78055, gammaEBV4-78268, gammaEBV4-78767, gammaEBV4-78969, gammaEBV4-79898, gammaEBV4-80010, gammaEBV4-80158, gammaEBV4-80180, gammaEBV4-80333, gammaEBV4-80380, gammaEBV4-80419, gammaEBV4-80444, gammaEBV4-81096, gammaEBV4-81378, gammaEBV4-81601, gammaEBV4-81671, gammaEBV4-81722, gammaEBV4-81794, gammaEBV4-81818, gammaEBV4-81950, gammaEBV4-81952, gammaEBV4-81957, gammaEBV4-81959, gammaEBV4-82027, gammaEBV4-82041, gammaEBV4-82056, gammaEBV4-82082, gammaEBV4-82089, gammaEBV4-82233, gammaEBV4-82234, gammaEBV4-82250, gammaEBV4-82253, gammaEBV4-82254, gammaEBV4-82259, gammaEBV4-82274, gammaEBV4-82347, gammaEBV4-82356, gammaEBV4-82358, gammaEBV4-82363, gammaEBV4-82366, gammaEBV4-82414, gammaEBV4-82418, gammaEBV4-82557, gammaEBV4-82685, gammaEBV4-82902, gammaEBV4-82911, gammaEBV4-83123, gammaEBV4-83632, gammaEBV4-84936, gammaEBV4-85018, gammaEBV4-85048, gammaEBV4-85131, gammaEBV4-85235, gammaEBV4-85666, gammaEBV4-87597, gammaEBV4-87818, gammaEBV4-87870, gammaEBV4-87967, gammaEBV4-88158, gammaEBV4-88365, gammaEBV4-88383, gammaEBV4-88385, gammaEBV4-88388, gammaEBV4-88390, gammaEBV4-88393, gammaEBV4-88397, gammaEBV4-88400, gammaEBV4-88401, gammaEBV4-88402, gammaEBV4-88405, gammaEBV4-88409, gammaEBV4-88410, gammaEBV4-88411, gammaEBV4-88412, gammaEBV4-88413, gammaEBV4-88414, gammaEBV4-88415, gammaEBV4-88416, gammaEBV4-88417, gammaEBV4-88419, gammaEBV4-88420, gammaEBV4-88421, gammaEBV4-88423, gammaEBV4-88424, gammaEBV4-88425, gammaEBV4-88426, gammaEBV4-88427, gammaEBV4-88429, gammaEBV4-88431, gammaEBV4-88432, gammaEBV4-88433, gammaEBV4-88435, gammaEBV4-88436, gammaEBV4-88437, gammaEBV4-88438, gammaEBV4-88439, gammaEBV4-88440, gammaEBV4-88442, gammaEBV4-88444, gammaEBV4-88445, gammaEBV4-88446, gammaEBV4-88447, gammaEBV4-88448, gammaEBV4-88449, gammaEBV4-88450, gammaEBV4-88451, gammaEBV4-88452, gammaEBV4-88453, gammaEBV4-88454, gammaEBV4-88455, gammaEBV4-88456, gammaEBV4-88457, gammaEBV4-88458, gammaEBV4-88459, gammaEBV4-88462, gammaEBV4-88463, gammaEBV4-88464, gammaEBV4-88465, gammaEBV4-88466, gammaEBV4-88467, gammaEBV4-88469, gammaEBV4-88470, gammaEBV4-88471, gammaEBV4-88472, gammaEBV4-88474, gammaEBV4-88476, gammaEBV4-88478, gammaEBV4-88483, gammaEBV4-88486, gammaEBV4-88495, gammaEBV4-88497, gammaEBV4-88498, gammaEBV4-88499, gammaEBV4-88500, gammaEBV4-88501, gammaEBV4-88502, gammaEBV4-88503, gammaEBV4-88504, gammaEBV4-88506, gammaEBV4-88507, gammaEBV4-88508, gammaEBV4-88511, gammaEBV4-88512, gammaEBV4-88514, gammaEBV4-88516, gammaEBV4-88527, gammaEBV4-88528, gammaEBV4-88529, gammaEBV4-88597, gammaEBV4-88834, gammaEBV4-89231, gammaEBV4-89456, gammaEBV4-90140, gammaEBV4-90282, gammaEBV4-90294, gammaEBV4-90298, gammaEBV4-90302, gammaEBV4-90304, gammaEBV4-90308, gammaEBV4-90311, gammaEBV4-90312, gammaEBV4-90313, gammaEBV4-90315, gammaEBV4-90317, gammaEBV4-90319, gammaEBV4-90320, gammaEBV4-90321, gammaEBV4-90322, gammaEBV4-90324, gammaEBV4-90332, gammaEBV4-90339, gammaEBV4-90340, gammaEBV4-90341, gammaEBV4-90342, gammaEBV4-90343, gammaEBV4-90347, gammaEBV4-90348, gammaEBV4-90349, gammaEBV4-90350, gammaEBV4-90351, gammaEBV4-90352, gammaEBV4-90353, gammaEBV4-90354, gammaEBV4-90355, gammaEBV4-90356, gammaEBV4-90357, gammaEBV4-90358, gammaEBV4-90359, gammaEBV4-90361, gammaEBV4-90370, gammaEBV4-90371, gammaEBV4-90372, gammaEBV4-90373, gammaEBV4-90376, gammaEBV4-90378, gammaEBV4-91150, gammaEBV4-91422, gammaEBV4-91471, gammaEBV4-91480, gammaEBV4-91705, gammaEBV4-93050, gammaEBV4-93159, gammaEBV4-93490, gammaEBV4-94085, gammaEBV4-94177, gammaEBV4-94212, gammaEBV4-94589, gammaEBV4-95186, gammaEBV4-95199, gammaEBV4-95240, gammaEBV4-95258, gammaEBV4-95261, gammaEBV4-95588, gammaEBV4-95590, gammaEBV4-95666, gammaEBV4-95906, gammaEBV4-95942, gammaEBV4-95961, gammaEBV4-95963, gammaEBV4-95969, gammaEBV4-95974, gammaEBV4-95983, gammaEBV4-95984, gammaEBV4-95999, gammaEBV4-96000, gammaEBV4-96003, gammaEBV4-96004, gammaEBV4-96006, gammaEBV4-96008, gammaEBV4-96010, gammaEBV4-96024, gammaEBV4-96029, gammaEBV4-96038, gammaEBV4-96055, gammaEBV4-96064, gammaEBV4-96065, gammaEBV4-96070, gammaEBV4-96073, gammaEBV4-96078, gammaEBV4-96096, gammaEBV4-96114, gammaEBV4-96117, gammaEBV4-96118, gammaEBV4-96123, gammaEBV4-96127, gammaEBV4-96128, gammaEBV4-96142, gammaEBV4-96181, gammaEBV4-96193, gammaEBV4-96197, gammaEBV4-96199, gammaEBV4-96203, gammaEBV4-96205, gammaEBV4-96207, gammaEBV4-96208, gammaEBV4-96240, gammaEBV4-96254, gammaEBV4-96259, gammaEBV4-96266, gammaEBV4-96276, gammaEBV4-96279, gammaEBV4-96280, gammaEBV4-96281, gammaEBV4-96289, gammaEBV4-96296, gammaEBV4-96298, gammaEBV4-96302, gammaEBV4-96306, gammaEBV4-96310, gammaEBV4-96313, gammaEBV4-96314, gammaEBV4-96315, gammaEBV4-96316, gammaEBV4-96319, gammaEBV4-96328, gammaEBV4-96333, gammaEBV4-96334, gammaEBV4-96337, gammaEBV4-96346, gammaEBV4-96349, gammaEBV4-96357, gammaEBV4-96364, gammaEBV4-96365, gammaEBV4-96367, gammaEBV4-96370, gammaEBV4-96373, gammaEBV4-96378, gammaEBV4-96379, gammaEBV4-96380, gammaEBV4-96382, gammaEBV4-96387, gammaEBV4-96388, gammaEBV4-96394, gammaEBV4-96396, gammaEBV4-96399, gammaEBV4-96400, gammaEBV4-96403, gammaEBV4-96405, gammaEBV4-96406, gammaEBV4-96410, gammaEBV4-96414, gammaEBV4-96415, gammaEBV4-96416, gammaEBV4-96417, gammaEBV4-96419, gammaEBV4-96422, gammaEBV4-96424, gammaEBV4-96425, gammaEBV4-96430, gammaEBV4-96436, gammaEBV4-96440, gammaEBV4-96442, gammaEBV4-96445, gammaEBV4-96446, gammaEBV4-96459, gammaEBV4-96462, gammaEBV4-96464, gammaEBV4-96466, gammaEBV4-96467, gammaEBV4-96468, gammaEBV4-96470, gammaEBV4-96472, gammaEBV4-96473, gammaEBV4-96481, gammaEBV4-96505, gammaEBV4-96521, gammaEBV4-96522, gammaEBV4-96542, gammaEBV4-96546, gammaEBV4-96554, gammaEBV4-96565, gammaEBV4-96566, gammaEBV4-96569, gammaEBV4-96575, gammaEBV4-96582, gammaEBV4-96586, gammaEBV4-96587, gammaEBV4-96588, gammaEBV4-96591, gammaEBV4-96592, gammaEBV4-96594, gammaEBV4-96595, gammaEBV4-96596, gammaEBV4-96598, gammaEBV4-96599, gammaEBV4-96601, gammaEBV4-96604, gammaEBV4-96607, gammaEBV4-96618, gammaEBV4-96622, gammaEBV4-96633, gammaEBV4-96634, gammaEBV4-96636, gammaEBV4-96637, gammaEBV4-96638, gammaEBV4-96640, gammaEBV4-96653, gammaEBV4-96675, gammaEBV4-96677, gammaEBV4-96685, gammaEBV4-96701, gammaEBV4-96703, gammaEBV4-96714, gammaEBV4-96719, gammaEBV4-96722, gammaEBV4-96796, gammaEBV4-97303, gammaEBV4-97310, gammaEBV4-97457, gammaEBV4-97485, gammaEBV4-97584, gammaEBV4-97632, gammaEBV4-98321, gammaEBV4-98485, gammaEBV4-98607, gammaEBV4-98649, gammaEBV4-98857, gammaEBV4-99257, gammaEBV4-99261, gammaEBV4-99275, gammaEBV4-99399, gammaEBV4-99402, gammaEBV4-99405, gammaEBV4-99422, gammaEBV4-99423, gammaEBV4-99440, gammaEBV4-99441, gammaEBV4-99535

Row 58: gene = EBNA-3A

gene: EBNA-3A

sample: CAEBV1, CAEBV10, CAEBV11, CAEBV12, CAEBV13, CAEBV14, CAEBV15, CAEBV16, CAEBV17, CAEBV18, CAEBV19, CAEBV2, CAEBV20, CAEBV21, CAEBV22, CAEBV23, CAEBV24, CAEBV3, CAEBV5, CAEBV6, CAEBV7, CAEBV8, CAEBV9

pos: gammaEBV4-10539, gammaEBV4-10920, gammaEBV4-11339, gammaEBV4-11955, gammaEBV4-13361, gammaEBV4-16098, gammaEBV4-19491, gammaEBV4-22574, gammaEBV4-26126, gammaEBV4-34536, gammaEBV4-35458, gammaEBV4-35769, gammaEBV4-36014, gammaEBV4-36209, gammaEBV4-36221, gammaEBV4-36265, gammaEBV4-36307, gammaEBV4-36315, gammaEBV4-36322, gammaEBV4-36355, gammaEBV4-36356, gammaEBV4-36360, gammaEBV4-36368, gammaEBV4-36389, gammaEBV4-36390, gammaEBV4-36391, gammaEBV4-36393, gammaEBV4-36394, gammaEBV4-36397, gammaEBV4-36399, gammaEBV4-36401, gammaEBV4-36402, gammaEBV4-36403, gammaEBV4-36405, gammaEBV4-36406, gammaEBV4-36408, gammaEBV4-36410, gammaEBV4-36411, gammaEBV4-36414, gammaEBV4-36415, gammaEBV4-36417, gammaEBV4-36420, gammaEBV4-36421, gammaEBV4-36423, gammaEBV4-36424, gammaEBV4-36425, gammaEBV4-36429, gammaEBV4-36432, gammaEBV4-36433, gammaEBV4-36435, gammaEBV4-36436, gammaEBV4-36437, gammaEBV4-36438, gammaEBV4-36439, gammaEBV4-36441, gammaEBV4-36442, gammaEBV4-36445, gammaEBV4-36446, gammaEBV4-36447, gammaEBV4-36448, gammaEBV4-36449, gammaEBV4-36450, gammaEBV4-36451, gammaEBV4-36453, gammaEBV4-36454, gammaEBV4-36455, gammaEBV4-36457, gammaEBV4-36458, gammaEBV4-36459, gammaEBV4-36460, gammaEBV4-36462, gammaEBV4-36463, gammaEBV4-36464, gammaEBV4-36465, gammaEBV4-36466, gammaEBV4-36468, gammaEBV4-36469, gammaEBV4-36470, gammaEBV4-36472, gammaEBV4-36475, gammaEBV4-36476, gammaEBV4-36477, gammaEBV4-36478, gammaEBV4-36479, gammaEBV4-36481, gammaEBV4-36482, gammaEBV4-36483, gammaEBV4-36486, gammaEBV4-36487, gammaEBV4-36489, gammaEBV4-36490, gammaEBV4-36491, gammaEBV4-36492, gammaEBV4-36495, gammaEBV4-36497, gammaEBV4-36498, gammaEBV4-36499, gammaEBV4-36501, gammaEBV4-36502, gammaEBV4-36507, gammaEBV4-36521, gammaEBV4-36528, gammaEBV4-36543, gammaEBV4-36558, gammaEBV4-36564, gammaEBV4-36710, gammaEBV4-36712, gammaEBV4-36745, gammaEBV4-36781, gammaEBV4-36801, gammaEBV4-36816, gammaEBV4-36834, gammaEBV4-36855, gammaEBV4-36856, gammaEBV4-36860, gammaEBV4-36865, gammaEBV4-36869, gammaEBV4-36882, gammaEBV4-36885, gammaEBV4-36897, gammaEBV4-36924, gammaEBV4-36940, gammaEBV4-36942, gammaEBV4-36946, gammaEBV4-36947, gammaEBV4-36961, gammaEBV4-36965, gammaEBV4-36993, gammaEBV4-37009, gammaEBV4-37019, gammaEBV4-37033, gammaEBV4-37044, gammaEBV4-37087, gammaEBV4-37113, gammaEBV4-37116, gammaEBV4-37130, gammaEBV4-37270, gammaEBV4-37490, gammaEBV4-37678, gammaEBV4-37924, gammaEBV4-37979, gammaEBV4-38989, gammaEBV4-39482, gammaEBV4-39873, gammaEBV4-39899, gammaEBV4-39903, gammaEBV4-39908, gammaEBV4-39915, gammaEBV4-39925, gammaEBV4-40194, gammaEBV4-40213, gammaEBV4-40219, gammaEBV4-40220, gammaEBV4-40224, gammaEBV4-40225, gammaEBV4-40263, gammaEBV4-40282, gammaEBV4-41499, gammaEBV4-41553, gammaEBV4-41648, gammaEBV4-41794, gammaEBV4-41821, gammaEBV4-43009, gammaEBV4-43140, gammaEBV4-43141, gammaEBV4-43142, gammaEBV4-43145, gammaEBV4-43146, gammaEBV4-43373, gammaEBV4-43465, gammaEBV4-43477, gammaEBV4-43509, gammaEBV4-43624, gammaEBV4-43684, gammaEBV4-43981, gammaEBV4-44109, gammaEBV4-44155, gammaEBV4-44242, gammaEBV4-44658, gammaEBV4-45678, gammaEBV4-45790, gammaEBV4-45805, gammaEBV4-45860, gammaEBV4-46042, gammaEBV4-46062, gammaEBV4-46257, gammaEBV4-46472, gammaEBV4-46706, gammaEBV4-46912, gammaEBV4-47220, gammaEBV4-47236, gammaEBV4-47240, gammaEBV4-47908, gammaEBV4-47955, gammaEBV4-48227, gammaEBV4-48251, gammaEBV4-48335, gammaEBV4-48519, gammaEBV4-48797, gammaEBV4-48983, gammaEBV4-49102, gammaEBV4-49719, gammaEBV4-50133, gammaEBV4-50242, gammaEBV4-51166, gammaEBV4-51500, gammaEBV4-51571, gammaEBV4-52091, gammaEBV4-52205, gammaEBV4-52211, gammaEBV4-52227, gammaEBV4-52259, gammaEBV4-52372, gammaEBV4-52814, gammaEBV4-52837, gammaEBV4-52952, gammaEBV4-53724, gammaEBV4-53902, gammaEBV4-54149, gammaEBV4-54243, gammaEBV4-54255, gammaEBV4-54491, gammaEBV4-54802, gammaEBV4-54921, gammaEBV4-55000, gammaEBV4-55730, gammaEBV4-55780, gammaEBV4-55793, gammaEBV4-55811, gammaEBV4-55869, gammaEBV4-55887, gammaEBV4-55914, gammaEBV4-56237, gammaEBV4-56566, gammaEBV4-56866, gammaEBV4-57429, gammaEBV4-57632, gammaEBV4-57708, gammaEBV4-57740, gammaEBV4-57806, gammaEBV4-57842, gammaEBV4-57869, gammaEBV4-57870, gammaEBV4-57879, gammaEBV4-57880, gammaEBV4-57896, gammaEBV4-57897, gammaEBV4-58047, gammaEBV4-58301, gammaEBV4-58407, gammaEBV4-58634, gammaEBV4-58692, gammaEBV4-58785, gammaEBV4-58867, gammaEBV4-59115, gammaEBV4-59448, gammaEBV4-59672, gammaEBV4-59799, gammaEBV4-59923, gammaEBV4-59992, gammaEBV4-60095, gammaEBV4-60374, gammaEBV4-60398, gammaEBV4-61987, gammaEBV4-62009, gammaEBV4-62182, gammaEBV4-62247, gammaEBV4-62391, gammaEBV4-62449, gammaEBV4-62499, gammaEBV4-62677, gammaEBV4-62690, gammaEBV4-62774, gammaEBV4-62778, gammaEBV4-62916, gammaEBV4-63356, gammaEBV4-63580, gammaEBV4-63692, gammaEBV4-64008, gammaEBV4-64199, gammaEBV4-64224, gammaEBV4-64324, gammaEBV4-64571, gammaEBV4-64795, gammaEBV4-64976, gammaEBV4-65127, gammaEBV4-65205, gammaEBV4-65433, gammaEBV4-65450, gammaEBV4-65685, gammaEBV4-65718, gammaEBV4-65785, gammaEBV4-65950, gammaEBV4-66061, gammaEBV4-66069, gammaEBV4-66363, gammaEBV4-66405, gammaEBV4-66612, gammaEBV4-66809, gammaEBV4-67279, gammaEBV4-67485, gammaEBV4-67505, gammaEBV4-67749, gammaEBV4-68057, gammaEBV4-68261, gammaEBV4-68279, gammaEBV4-68497, gammaEBV4-68583, gammaEBV4-68589, gammaEBV4-68612, gammaEBV4-68646, gammaEBV4-68657, gammaEBV4-68729, gammaEBV4-68748, gammaEBV4-69148, gammaEBV4-69192, gammaEBV4-69276, gammaEBV4-69611, gammaEBV4-69904, gammaEBV4-69950, gammaEBV4-70330, gammaEBV4-70538, gammaEBV4-71139, gammaEBV4-71229, gammaEBV4-71292, gammaEBV4-71344, gammaEBV4-71546, gammaEBV4-71618, gammaEBV4-71671, gammaEBV4-72053, gammaEBV4-72289, gammaEBV4-72560, gammaEBV4-72580, gammaEBV4-72690, gammaEBV4-72723, gammaEBV4-72979, gammaEBV4-73029, gammaEBV4-73305, gammaEBV4-73366, gammaEBV4-73378, gammaEBV4-73577, gammaEBV4-73713, gammaEBV4-73903, gammaEBV4-73989, gammaEBV4-74183, gammaEBV4-74250, gammaEBV4-74856, gammaEBV4-74882, gammaEBV4-75292, gammaEBV4-75357, gammaEBV4-75429, gammaEBV4-75555, gammaEBV4-75694, gammaEBV4-75699, gammaEBV4-76014, gammaEBV4-76449, gammaEBV4-76489, gammaEBV4-76525, gammaEBV4-76598, gammaEBV4-76943, gammaEBV4-77179, gammaEBV4-77304, gammaEBV4-77373, gammaEBV4-77401, gammaEBV4-77404, gammaEBV4-77419, gammaEBV4-77613, gammaEBV4-77647, gammaEBV4-77672, gammaEBV4-77826, gammaEBV4-78055, gammaEBV4-78268, gammaEBV4-78767, gammaEBV4-78969, gammaEBV4-79898, gammaEBV4-80010, gammaEBV4-80158, gammaEBV4-80180, gammaEBV4-80333, gammaEBV4-80380, gammaEBV4-80419, gammaEBV4-80444, gammaEBV4-81096, gammaEBV4-81378, gammaEBV4-81601, gammaEBV4-81671, gammaEBV4-81722, gammaEBV4-81794, gammaEBV4-81818, gammaEBV4-81950, gammaEBV4-81952, gammaEBV4-81957, gammaEBV4-81959, gammaEBV4-82027, gammaEBV4-82041, gammaEBV4-82056, gammaEBV4-82082, gammaEBV4-82089, gammaEBV4-82233, gammaEBV4-82234, gammaEBV4-82250, gammaEBV4-82253, gammaEBV4-82254, gammaEBV4-82259, gammaEBV4-82274, gammaEBV4-82347, gammaEBV4-82356, gammaEBV4-82358, gammaEBV4-82363, gammaEBV4-82366, gammaEBV4-82414, gammaEBV4-82418, gammaEBV4-82557, gammaEBV4-82685, gammaEBV4-82902, gammaEBV4-82911, gammaEBV4-83123, gammaEBV4-83632, gammaEBV4-84936, gammaEBV4-9391, gammaEBV4-9426, gammaEBV4-9427, gammaEBV4-9428, gammaEBV4-9430, gammaEBV4-9432, gammaEBV4-9433, gammaEBV4-9434, gammaEBV4-9437, gammaEBV4-9438, gammaEBV4-9439, gammaEBV4-9442, gammaEBV4-9444, gammaEBV4-9446, gammaEBV4-9447, gammaEBV4-9518, gammaEBV4-9679, gammaEBV4-9712

Row 59: gene = EBNA-LP

gene: EBNA-LP

sample: CAEBV1, CAEBV10, CAEBV11, CAEBV12, CAEBV13, CAEBV14, CAEBV15, CAEBV16, CAEBV17, CAEBV18, CAEBV19CAEBV2, CAEBV20, CAEBV21, CAEBV22, CAEBV23, CAEBV24, CAEBV3, CAEBV5, CAEBV6, CAEBV7, CAEBV8, CAEBV9

pos: gammaEBV4-13361, gammaEBV4-16098, gammaEBV4-19491, gammaEBV4-22574, gammaEBV4-26126, gammaEBV4-34536, gammaEBV4-35458, gammaEBV4-35769, gammaEBV4-36014, gammaEBV4-36209, gammaEBV4-36221, gammaEBV4-36265, gammaEBV4-36307, gammaEBV4-36315, gammaEBV4-36322, gammaEBV4-36355, gammaEBV4-36356, gammaEBV4-36360, gammaEBV4-36368, gammaEBV4-36389, gammaEBV4-36390, gammaEBV4-36391, gammaEBV4-36393, gammaEBV4-36394, gammaEBV4-36397, gammaEBV4-36399, gammaEBV4-36401, gammaEBV4-36402, gammaEBV4-36403, gammaEBV4-36405, gammaEBV4-36406, gammaEBV4-36408, gammaEBV4-36410, gammaEBV4-36411, gammaEBV4-36414, gammaEBV4-36415, gammaEBV4-36417, gammaEBV4-36420, gammaEBV4-36421, gammaEBV4-36423, gammaEBV4-36424, gammaEBV4-36425, gammaEBV4-36429, gammaEBV4-36432, gammaEBV4-36433, gammaEBV4-36435, gammaEBV4-36436, gammaEBV4-36437, gammaEBV4-36438, gammaEBV4-36439, gammaEBV4-36441, gammaEBV4-36442, gammaEBV4-36445, gammaEBV4-36446, gammaEBV4-36447, gammaEBV4-36448, gammaEBV4-36449, gammaEBV4-36450, gammaEBV4-36451, gammaEBV4-36453, gammaEBV4-36454, gammaEBV4-36455, gammaEBV4-36457, gammaEBV4-36458, gammaEBV4-36459, gammaEBV4-36460, gammaEBV4-36462, gammaEBV4-36463, gammaEBV4-36464, gammaEBV4-36465, gammaEBV4-36466, gammaEBV4-36468, gammaEBV4-36469, gammaEBV4-36470, gammaEBV4-36472, gammaEBV4-36475, gammaEBV4-36476, gammaEBV4-36477, gammaEBV4-36478, gammaEBV4-36479, gammaEBV4-36481, gammaEBV4-36482, gammaEBV4-36483, gammaEBV4-36486, gammaEBV4-36487, gammaEBV4-36489, gammaEBV4-36490, gammaEBV4-36491, gammaEBV4-36492, gammaEBV4-36495, gammaEBV4-36497, gammaEBV4-36498, gammaEBV4-36499, gammaEBV4-36501, gammaEBV4-36502, gammaEBV4-36507, gammaEBV4-36521, gammaEBV4-36528, gammaEBV4-36543, gammaEBV4-36558, gammaEBV4-36564, gammaEBV4-36710, gammaEBV4-36712, gammaEBV4-36745, gammaEBV4-36781, gammaEBV4-36801, gammaEBV4-36816, gammaEBV4-36834, gammaEBV4-36855, gammaEBV4-36856, gammaEBV4-36860, gammaEBV4-36865, gammaEBV4-36869, gammaEBV4-36882, gammaEBV4-36885, gammaEBV4-36897, gammaEBV4-36924, gammaEBV4-36940, gammaEBV4-36942, gammaEBV4-36946, gammaEBV4-36947, gammaEBV4-36961, gammaEBV4-36965, gammaEBV4-36993, gammaEBV4-37009, gammaEBV4-37019, gammaEBV4-37033, gammaEBV4-37044, gammaEBV4-37087, gammaEBV4-37113, gammaEBV4-37116, gammaEBV4-37130, gammaEBV4-37270, gammaEBV4-37490, gammaEBV4-37678, gammaEBV4-37924, gammaEBV4-37979, gammaEBV4-38989, gammaEBV4-39482

Row 60: gene = LF1

gene: LF1

sample: CAEBV1, CAEBV13, CAEBV17, CAEBV19, CAEBV20, CAEBV22, CAEBV5, CAEBV6, CAEBV8, CAEBV9

pos: gammaEBV4-148630, gammaEBV4-148650, gammaEBV4-148658, gammaEBV4-148892, gammaEBV4-148962, gammaEBV4-148975, gammaEBV4-149020, gammaEBV4-149096, gammaEBV4-149397, gammaEBV4-149494, gammaEBV4-149760, gammaEBV4-150546, gammaEBV4-150551, gammaEBV4-150702, gammaEBV4-150749, gammaEBV4-151271, gammaEBV4-151272, gammaEBV4-152545, gammaEBV4-152557, gammaEBV4-152613, gammaEBV4-153004, gammaEBV4-153077, gammaEBV4-153117, gammaEBV4-153180, gammaEBV4-153617, gammaEBV4-153639

Row 61: gene = LF2

gene: LF2

sample: CAEBV11, CAEBV19, CAEBV20, CAEBV22, CAEBV5, CAEBV6, CAEBV8, CAEBV9

pos: gammaEBV4-147843, gammaEBV4-147959, gammaEBV4-148630, gammaEBV4-148650, gammaEBV4-148658, gammaEBV4-148892, gammaEBV4-148962, gammaEBV4-148975, gammaEBV4-149020, gammaEBV4-149096, gammaEBV4-149397, gammaEBV4-149494, gammaEBV4-149760, gammaEBV4-150546, gammaEBV4-150551, gammaEBV4-150702, gammaEBV4-150749, gammaEBV4-151271, gammaEBV4-151272

Row 62: gene = LMP-1

gene: LMP-1

sample: CAEBV1, CAEBV10, CAEBV11, CAEBV13, CAEBV19, CAEBV2, CAEBV20, CAEBV21, CAEBV5, CAEBV6, CAEBV8, CAEBV9

pos: gammaEBV4-164551, gammaEBV4-164586, gammaEBV4-164676, gammaEBV4-164692, gammaEBV4-164787, gammaEBV4-164982, gammaEBV4-165051, gammaEBV4-165155, gammaEBV4-165639, gammaEBV4-165807, gammaEBV4-166017, gammaEBV4-166116, gammaEBV4-166237, gammaEBV4-166370, gammaEBV4-166656, gammaEBV4-166695, gammaEBV4-166813, gammaEBV4-167362, gammaEBV4-167370, gammaEBV4-167399, gammaEBV4-167404, gammaEBV4-167437, gammaEBV4-167488, gammaEBV4-167506, gammaEBV4-168640, gammaEBV4-169257, gammaEBV4-169350, gammaEBV4-169430, gammaEBV4-169442, gammaEBV4-169460, gammaEBV4-169485, gammaEBV4-169565

Row 63: gene = LMP-2B

gene: LMP-2B

sample: CAEBV1, CAEBV10, CAEBV11, CAEBV12, CAEBV13, CAEBV14, CAEBV15, CAEBV16, CAEBV17, CAEBV18, CAEBV19, CAEBV2, CAEBV20, CAEBV21, CAEBV22, CAEBV23, CAEBV24, CAEBV3, CAEBV4, CAEBV5, CAEBV6, CAEBV7, CAEBV8, CAEBV9

pos: gammaEBV4-100012, gammaEBV4-100015, gammaEBV4-100017, gammaEBV4-100032, gammaEBV4-100470, gammaEBV4-100929, gammaEBV4-101078, gammaEBV4-101090, gammaEBV4-101143, gammaEBV4-101244, gammaEBV4-101896, gammaEBV4-101996, gammaEBV4-102132, gammaEBV4-102279, gammaEBV4-102548, gammaEBV4-102833, gammaEBV4-102972, gammaEBV4-103167, gammaEBV4-103346, gammaEBV4-103649, gammaEBV4-103838, gammaEBV4-104080, gammaEBV4-104243, gammaEBV4-104986, gammaEBV4-104994, gammaEBV4-105325, gammaEBV4-10539, gammaEBV4-105501, gammaEBV4-105542, gammaEBV4-105598, gammaEBV4-106045, gammaEBV4-106231, gammaEBV4-106388, gammaEBV4-106430, gammaEBV4-107019, gammaEBV4-107473, gammaEBV4-107738, gammaEBV4-108135, gammaEBV4-108164, gammaEBV4-108165, gammaEBV4-108219, gammaEBV4-108226, gammaEBV4-108228, gammaEBV4-109037, gammaEBV4-109074, gammaEBV4-10920, gammaEBV4-110375, gammaEBV4-110393, gammaEBV4-110468, gammaEBV4-110538, gammaEBV4-110756, gammaEBV4-110937, gammaEBV4-111238, gammaEBV4-112229, gammaEBV4-112312, gammaEBV4-112741, gammaEBV4-113146, gammaEBV4-11339, gammaEBV4-113587, gammaEBV4-113631, gammaEBV4-113886, gammaEBV4-113897, gammaEBV4-114033, gammaEBV4-114134, gammaEBV4-114178, gammaEBV4-114596, gammaEBV4-114785, gammaEBV4-115055, gammaEBV4-115222, gammaEBV4-115343, gammaEBV4-115861, gammaEBV4-115891, gammaEBV4-115990, gammaEBV4-116738, gammaEBV4-117372, gammaEBV4-117462, gammaEBV4-117583, gammaEBV4-117880, gammaEBV4-117936, gammaEBV4-117941, gammaEBV4-118378, gammaEBV4-118380, gammaEBV4-118437, gammaEBV4-118443, gammaEBV4-118497, gammaEBV4-118554, gammaEBV4-118559, gammaEBV4-119450, gammaEBV4-11955, gammaEBV4-119719, gammaEBV4-119738, gammaEBV4-119764, gammaEBV4-119766, gammaEBV4-120040, gammaEBV4-120837, gammaEBV4-121112, gammaEBV4-121131, gammaEBV4-122449, gammaEBV4-122641, gammaEBV4-123006, gammaEBV4-123073, gammaEBV4-123123, gammaEBV4-123468, gammaEBV4-123550, gammaEBV4-123573, gammaEBV4-123594, gammaEBV4-123722, gammaEBV4-123726, gammaEBV4-123773, gammaEBV4-123786, gammaEBV4-123790, gammaEBV4-123855, gammaEBV4-124007, gammaEBV4-124331, gammaEBV4-124333, gammaEBV4-124335, gammaEBV4-124336, gammaEBV4-124337, gammaEBV4-124338, gammaEBV4-124339, gammaEBV4-124340, gammaEBV4-124342, gammaEBV4-124343, gammaEBV4-124344, gammaEBV4-124345, gammaEBV4-124346, gammaEBV4-124347, gammaEBV4-124348, gammaEBV4-124349, gammaEBV4-124350, gammaEBV4-124351, gammaEBV4-124352, gammaEBV4-124356, gammaEBV4-124357, gammaEBV4-124373, gammaEBV4-124681, gammaEBV4-124988, gammaEBV4-125072, gammaEBV4-125106, gammaEBV4-125211, gammaEBV4-125323, gammaEBV4-125330, gammaEBV4-125786, gammaEBV4-125876, gammaEBV4-125881, gammaEBV4-126135, gammaEBV4-126219, gammaEBV4-126402, gammaEBV4-127266, gammaEBV4-127347, gammaEBV4-127645, gammaEBV4-127663, gammaEBV4-127669, gammaEBV4-127827, gammaEBV4-128010, gammaEBV4-128770, gammaEBV4-128837, gammaEBV4-129213, gammaEBV4-129395, gammaEBV4-130293, gammaEBV4-130302, gammaEBV4-130543, gammaEBV4-130568, gammaEBV4-130569, gammaEBV4-130696, gammaEBV4-130723, gammaEBV4-130930, gammaEBV4-131300, gammaEBV4-131367, gammaEBV4-131675, gammaEBV4-132155, gammaEBV4-132194, gammaEBV4-132223, gammaEBV4-132224, gammaEBV4-132232, gammaEBV4-132437, gammaEBV4-133088, gammaEBV4-13361, gammaEBV4-133974, gammaEBV4-134311, gammaEBV4-134348, gammaEBV4-134460, gammaEBV4-134537, gammaEBV4-134765, gammaEBV4-134834, gammaEBV4-135676, gammaEBV4-135687, gammaEBV4-135692, gammaEBV4-135852, gammaEBV4-135865, gammaEBV4-136075, gammaEBV4-136560, gammaEBV4-136578, gammaEBV4-136653, gammaEBV4-136754, gammaEBV4-136896, gammaEBV4-136927, gammaEBV4-137017, gammaEBV4-137032, gammaEBV4-137120, gammaEBV4-137135, gammaEBV4-137142, gammaEBV4-137148, gammaEBV4-137151, gammaEBV4-137170, gammaEBV4-137227, gammaEBV4-137268, gammaEBV4-138016, gammaEBV4-138281, gammaEBV4-138618, gammaEBV4-138625, gammaEBV4-138872, gammaEBV4-138960, gammaEBV4-139071, gammaEBV4-139128, gammaEBV4-139154, gammaEBV4-139166, gammaEBV4-139196, gammaEBV4-139198, gammaEBV4-139202, gammaEBV4-139204, gammaEBV4-139207, gammaEBV4-139209, gammaEBV4-139214, gammaEBV4-139216, gammaEBV4-139438, gammaEBV4-139502, gammaEBV4-140335, gammaEBV4-140663, gammaEBV4-142143, gammaEBV4-142423, gammaEBV4-144869, gammaEBV4-145085, gammaEBV4-145352, gammaEBV4-145444, gammaEBV4-146172, gammaEBV4-146843, gammaEBV4-147843, gammaEBV4-147959, gammaEBV4-148630, gammaEBV4-148650, gammaEBV4-148658, gammaEBV4-148892, gammaEBV4-148962, gammaEBV4-148975, gammaEBV4-149020, gammaEBV4-149096, gammaEBV4-149397, gammaEBV4-149494, gammaEBV4-149760, gammaEBV4-150546, gammaEBV4-150551, gammaEBV4-150702, gammaEBV4-150749, gammaEBV4-151271, gammaEBV4-151272, gammaEBV4-152545, gammaEBV4-152557, gammaEBV4-152613, gammaEBV4-153004, gammaEBV4-153077, gammaEBV4-153117, gammaEBV4-153180, gammaEBV4-153617, gammaEBV4-153639, gammaEBV4-153725, gammaEBV4-153763, gammaEBV4-153860, gammaEBV4-153864, gammaEBV4-153952, gammaEBV4-154055, gammaEBV4-154299, gammaEBV4-154429, gammaEBV4-154453, gammaEBV4-154654, gammaEBV4-154720, gammaEBV4-154982, gammaEBV4-155166, gammaEBV4-155263, gammaEBV4-155584, gammaEBV4-155832, gammaEBV4-156147, gammaEBV4-156363, gammaEBV4-156375, gammaEBV4-156562, gammaEBV4-156606, gammaEBV4-157058, gammaEBV4-157364, gammaEBV4-157434, gammaEBV4-157853, gammaEBV4-159158, gammaEBV4-159376, gammaEBV4-160788, gammaEBV4-16098, gammaEBV4-161004, gammaEBV4-161324, gammaEBV4-161348, gammaEBV4-161445, gammaEBV4-161533, gammaEBV4-162029, gammaEBV4-162140, gammaEBV4-162204, gammaEBV4-162480, gammaEBV4-162703, gammaEBV4-162758, gammaEBV4-163569, gammaEBV4-163668, gammaEBV4-163804, gammaEBV4-163829, gammaEBV4-164100, gammaEBV4-164161, gammaEBV4-164382, gammaEBV4-164387, gammaEBV4-164551, gammaEBV4-164586, gammaEBV4-164676, gammaEBV4-164692, gammaEBV4-164787, gammaEBV4-164982, gammaEBV4-165051, gammaEBV4-165155, gammaEBV4-165639, gammaEBV4-165807, gammaEBV4-166017, gammaEBV4-166116, gammaEBV4-166237, gammaEBV4-166370, gammaEBV4-166656, gammaEBV4-166695, gammaEBV4-166813, gammaEBV4-167362, gammaEBV4-167370, gammaEBV4-167399, gammaEBV4-167404, gammaEBV4-167437, gammaEBV4-167488, gammaEBV4-167506, gammaEBV4-168640, gammaEBV4-169257, gammaEBV4-169350, gammaEBV4-169430, gammaEBV4-169442, gammaEBV4-169460, gammaEBV4-169485, gammaEBV4-169565, gammaEBV4-171084, gammaEBV4-171244, gammaEBV4-171498, gammaEBV4-171768, gammaEBV4-171773, gammaEBV4-171783, gammaEBV4-187, gammaEBV4-19491, gammaEBV4-22574, gammaEBV4-2403, gammaEBV4-26126, gammaEBV4-2768, gammaEBV4-2769, gammaEBV4-2958, gammaEBV4-336, gammaEBV4-3452, gammaEBV4-34536, gammaEBV4-35458, gammaEBV4-35769, gammaEBV4-36014, gammaEBV4-36209, gammaEBV4-36221, gammaEBV4-36265, gammaEBV4-36307, gammaEBV4-36315, gammaEBV4-36322, gammaEBV4-36355, gammaEBV4-36356, gammaEBV4-36360, gammaEBV4-36368, gammaEBV4-36389, gammaEBV4-36390, gammaEBV4-36391, gammaEBV4-36393, gammaEBV4-36394, gammaEBV4-36397, gammaEBV4-36399, gammaEBV4-3640, gammaEBV4-36401, gammaEBV4-36402, gammaEBV4-36403, gammaEBV4-36405, gammaEBV4-36406, gammaEBV4-36408, gammaEBV4-36410, gammaEBV4-36411, gammaEBV4-36414, gammaEBV4-36415, gammaEBV4-36417, gammaEBV4-36420, gammaEBV4-36421, gammaEBV4-36423, gammaEBV4-36424, gammaEBV4-36425, gammaEBV4-36429, gammaEBV4-36432, gammaEBV4-36433, gammaEBV4-36435, gammaEBV4-36436, gammaEBV4-36437, gammaEBV4-36438, gammaEBV4-36439, gammaEBV4-36441, gammaEBV4-36442, gammaEBV4-36445, gammaEBV4-36446, gammaEBV4-36447, gammaEBV4-36448, gammaEBV4-36449, gammaEBV4-36450, gammaEBV4-36451, gammaEBV4-36453, gammaEBV4-36454, gammaEBV4-36455, gammaEBV4-36457, gammaEBV4-36458, gammaEBV4-36459, gammaEBV4-36460, gammaEBV4-36462, gammaEBV4-36463, gammaEBV4-36464, gammaEBV4-36465, gammaEBV4-36466, gammaEBV4-36468, gammaEBV4-36469, gammaEBV4-36470, gammaEBV4-36472, gammaEBV4-36475, gammaEBV4-36476, gammaEBV4-36477, gammaEBV4-36478, gammaEBV4-36479, gammaEBV4-36481, gammaEBV4-36482, gammaEBV4-36483, gammaEBV4-36486, gammaEBV4-36487, gammaEBV4-36489, gammaEBV4-36490, gammaEBV4-36491, gammaEBV4-36492, gammaEBV4-36495, gammaEBV4-36497, gammaEBV4-36498, gammaEBV4-36499, gammaEBV4-36501, gammaEBV4-36502, gammaEBV4-36507, gammaEBV4-36521, gammaEBV4-36528, gammaEBV4-36543, gammaEBV4-36558, gammaEBV4-36564, gammaEBV4-36710, gammaEBV4-36712, gammaEBV4-36745, gammaEBV4-36781, gammaEBV4-36801, gammaEBV4-36816, gammaEBV4-36834, gammaEBV4-36855, gammaEBV4-36856, gammaEBV4-36860, gammaEBV4-36865, gammaEBV4-36869, gammaEBV4-36882, gammaEBV4-36885, gammaEBV4-36897, gammaEBV4-36924, gammaEBV4-36940, gammaEBV4-36942, gammaEBV4-36946, gammaEBV4-36947, gammaEBV4-36961, gammaEBV4-36965, gammaEBV4-36993, gammaEBV4-37009, gammaEBV4-37019, gammaEBV4-37033, gammaEBV4-37044, gammaEBV4-37087,

gammaEBV4-37113, gammaEBV4-37116, gammaEBV4-37130, gammaEBV4-3720, gammaEBV4-37270, gammaEBV4-37490, gammaEBV4-3754, gammaEBV4-3758, gammaEBV4-37678, gammaEBV4-3775, gammaEBV4-37924, gammaEBV4-37979, gammaEBV4-38989, gammaEBV4-39482, gammaEBV4-39873, gammaEBV4-39899, gammaEBV4-39903, gammaEBV4-39908, gammaEBV4-39915, gammaEBV4-39925, gammaEBV4-40194, gammaEBV4-40213, gammaEBV4-40219, gammaEBV4-40220, gammaEBV4-40224, gammaEBV4-40225, gammaEBV4-40263, gammaEBV4-40282, gammaEBV4-4082, gammaEBV4-413, gammaEBV4-41499, gammaEBV4-41553, gammaEBV4-416, gammaEBV4-41648, gammaEBV4-41794, gammaEBV4-41821, gammaEBV4-43009, gammaEBV4-43140, gammaEBV4-43141, gammaEBV4-43142, gammaEBV4-43145, gammaEBV4-43146, gammaEBV4-43373, gammaEBV4-43465, gammaEBV4-43477, gammaEBV4-43509, gammaEBV4-43624, gammaEBV4-43684, gammaEBV4-43981, gammaEBV4-44109, gammaEBV4-44155, gammaEBV4-44242, gammaEBV4-44658, gammaEBV4-45678, gammaEBV4-45790, gammaEBV4-45805, gammaEBV4-45860, gammaEBV4-46042, gammaEBV4-46062, gammaEBV4-46257, gammaEBV4-46472, gammaEBV4-46706, gammaEBV4-46912, gammaEBV4-47220, gammaEBV4-47236, gammaEBV4-47240, gammaEBV4-47908, gammaEBV4-47955, gammaEBV4-48227, gammaEBV4-48251, gammaEBV4-48335, gammaEBV4-48519, gammaEBV4-48797, gammaEBV4-48983, gammaEBV4-49102, gammaEBV4-4944, gammaEBV4-49719, gammaEBV4-50133, gammaEBV4-50242, gammaEBV4-5080, gammaEBV4-51166, gammaEBV4-51500, gammaEBV4-51571, gammaEBV4-5182, gammaEBV4-52091, gammaEBV4-52205, gammaEBV4-52211, gammaEBV4-52227, gammaEBV4-52259, gammaEBV4-52372, gammaEBV4-52814, gammaEBV4-52837, gammaEBV4-52952, gammaEBV4-53724, gammaEBV4-53902, gammaEBV4-5406, gammaEBV4-54149, gammaEBV4-54243, gammaEBV4-54255, gammaEBV4-54491, gammaEBV4-54802, gammaEBV4-54921, gammaEBV4-55000, gammaEBV4-55730, gammaEBV4-55780, gammaEBV4-55793, gammaEBV4-55811, gammaEBV4-55869, gammaEBV4-55887, gammaEBV4-55914, gammaEBV4-56237, gammaEBV4-56566, gammaEBV4-5665, gammaEBV4-56866, gammaEBV4-5739, gammaEBV4-57429, gammaEBV4-5751, gammaEBV4-5758, gammaEBV4-5762, gammaEBV4-57632, gammaEBV4-57708, gammaEBV4-57740, gammaEBV4-57806, gammaEBV4-57842, gammaEBV4-57869, gammaEBV4-57870, gammaEBV4-57879, gammaEBV4-57880, gammaEBV4-57896, gammaEBV4-57897, gammaEBV4-5793, gammaEBV4-5796, gammaEBV4-5798, gammaEBV4-5799, gammaEBV4-5802, gammaEBV4-5803, gammaEBV4-5804, gammaEBV4-58047, gammaEBV4-5805, gammaEBV4-5807, gammaEBV4-5808, gammaEBV4-5809, gammaEBV4-5810, gammaEBV4-58301, gammaEBV4-58407, gammaEBV4-58634, gammaEBV4-58692, gammaEBV4-58785, gammaEBV4-58867, gammaEBV4-59115, gammaEBV4-59448, gammaEBV4-59672, gammaEBV4-59799, gammaEBV4-59923, gammaEBV4-59992, gammaEBV4-60095, gammaEBV4-60374, gammaEBV4-60398, gammaEBV4-61987, gammaEBV4-62009, gammaEBV4-62182, gammaEBV4-62247, gammaEBV4-62391, gammaEBV4-62449, gammaEBV4-62499, gammaEBV4-62677, gammaEBV4-62690, gammaEBV4-62774, gammaEBV4-62778, gammaEBV4-62916, gammaEBV4-63356, gammaEBV4-63580, gammaEBV4-63692, gammaEBV4-64008, gammaEBV4-64199, gammaEBV4-64224, gammaEBV4-64324, gammaEBV4-64571, gammaEBV4-64795, gammaEBV4-64976, gammaEBV4-65127, gammaEBV4-65205, gammaEBV4-65433, gammaEBV4-65450, gammaEBV4-65685, gammaEBV4-65718, gammaEBV4-65785, gammaEBV4-65950, gammaEBV4-66061, gammaEBV4-66069, gammaEBV4-66363, gammaEBV4-66405, gammaEBV4-66612, gammaEBV4-66809, gammaEBV4-67279, gammaEBV4-67485, gammaEBV4-67505, gammaEBV4-67749, gammaEBV4-68057, gammaEBV4-68261, gammaEBV4-68279, gammaEBV4-68497, gammaEBV4-68583, gammaEBV4-68589, gammaEBV4-68612, gammaEBV4-68646, gammaEBV4-68657, gammaEBV4-68729, gammaEBV4-68748, gammaEBV4-69148, gammaEBV4-69192, gammaEBV4-69276, gammaEBV4-69611, gammaEBV4-69904, gammaEBV4-69950, gammaEBV4-7019, gammaEBV4-70330, gammaEBV4-7035, gammaEBV4-70538, gammaEBV4-71139, gammaEBV4-71229, gammaEBV4-71292, gammaEBV4-71344, gammaEBV4-71546, gammaEBV4-71618, gammaEBV4-71671, gammaEBV4-72053, gammaEBV4-7210, gammaEBV4-72289, gammaEBV4-7253, gammaEBV4-72560, gammaEBV4-72580, gammaEBV4-72690, gammaEBV4-72723, gammaEBV4-72979, gammaEBV4-73029, gammaEBV4-73305, gammaEBV4-73366, gammaEBV4-73378, gammaEBV4-73577, gammaEBV4-73713, gammaEBV4-73903, gammaEBV4-73989, gammaEBV4-74183, gammaEBV4-74250, gammaEBV4-74856, gammaEBV4-74882, gammaEBV4-75292, gammaEBV4-75357, gammaEBV4-75429, gammaEBV4-75555, gammaEBV4-75694, gammaEBV4-75699, gammaEBV4-76014, gammaEBV4-76449, gammaEBV4-76489, gammaEBV4-76525, gammaEBV4-76598, gammaEBV4-7691, gammaEBV4-76943, gammaEBV4-7705, gammaEBV4-77179, gammaEBV4-77304, gammaEBV4-77373, gammaEBV4-77401, gammaEBV4-77404, gammaEBV4-77419, gammaEBV4-77613, gammaEBV4-77647, gammaEBV4-77672, gammaEBV4-77826, gammaEBV4-78055, gammaEBV4-78268, gammaEBV4-78767, gammaEBV4-78969, gammaEBV4-79898, gammaEBV4-80010, gammaEBV4-80158, gammaEBV4-80180, gammaEBV4-80333, gammaEBV4-80380, gammaEBV4-80419, gammaEBV4-80444, gammaEBV4-8048, gammaEBV4-81096, gammaEBV4-81378, gammaEBV4-81601, gammaEBV4-81671, gammaEBV4-81722, gammaEBV4-81794, gammaEBV4-81818, gammaEBV4-81950, gammaEBV4-81952, gammaEBV4-81957, gammaEBV4-81959, gammaEBV4-82027, gammaEBV4-82041, gammaEBV4-82056, gammaEBV4-82082, gammaEBV4-82089, gammaEBV4-82233, gammaEBV4-82234, gammaEBV4-82250, gammaEBV4-82253, gammaEBV4-82254, gammaEBV4-82259, gammaEBV4-82274, gammaEBV4-82347, gammaEBV4-82356, gammaEBV4-82358, gammaEBV4-82363, gammaEBV4-82366, gammaEBV4-82414, gammaEBV4-82418, gammaEBV4-8244, gammaEBV4-82557, gammaEBV4-82685, gammaEBV4-82902, gammaEBV4-82911, gammaEBV4-83123, gammaEBV4-83632, gammaEBV4-84936, gammaEBV4-8500, gammaEBV4-85018, gammaEBV4-85048, gammaEBV4-85131, gammaEBV4-85235, gammaEBV4-8555, gammaEBV4-85666, gammaEBV4-87597, gammaEBV4-87818, gammaEBV4-87870, gammaEBV4-87967, gammaEBV4-88158, gammaEBV4-88365, gammaEBV4-88383, gammaEBV4-88385, gammaEBV4-88388, gammaEBV4-88390, gammaEBV4-88393, gammaEBV4-88397, gammaEBV4-88400, gammaEBV4-88401, gammaEBV4-88402, gammaEBV4-88405, gammaEBV4-88409, gammaEBV4-88410, gammaEBV4-88411, gammaEBV4-88412, gammaEBV4-88413, gammaEBV4-88414, gammaEBV4-88415, gammaEBV4-88416, gammaEBV4-88417, gammaEBV4-88419, gammaEBV4-88420, gammaEBV4-88421, gammaEBV4-88423, gammaEBV4-88424, gammaEBV4-88425, gammaEBV4-88426, gammaEBV4-88427, gammaEBV4-88429, gammaEBV4-88431, gammaEBV4-88432, gammaEBV4-88433, gammaEBV4-88435, gammaEBV4-88436, gammaEBV4-88437, gammaEBV4-88438, gammaEBV4-88439, gammaEBV4-88440, gammaEBV4-88442, gammaEBV4-88444, gammaEBV4-88445, gammaEBV4-88446, gammaEBV4-88447, gammaEBV4-88448, gammaEBV4-88449, gammaEBV4-88450, gammaEBV4-88451, gammaEBV4-88452, gammaEBV4-88453, gammaEBV4-88454, gammaEBV4-88455, gammaEBV4-88456, gammaEBV4-88457, gammaEBV4-88458, gammaEBV4-88459, gammaEBV4-88462, gammaEBV4-88463, gammaEBV4-88464, gammaEBV4-88465, gammaEBV4-88466, gammaEBV4-88467, gammaEBV4-88469, gammaEBV4-88470, gammaEBV4-88471, gammaEBV4-88472, gammaEBV4-88474, gammaEBV4-88476, gammaEBV4-88478, gammaEBV4-88483, gammaEBV4-88486, gammaEBV4-88495, gammaEBV4-88497, gammaEBV4-88498, gammaEBV4-88499, gammaEBV4-88500, gammaEBV4-88501, gammaEBV4-88502, gammaEBV4-88503, gammaEBV4-88504, gammaEBV4-88506, gammaEBV4-88507, gammaEBV4-88508, gammaEBV4-88511, gammaEBV4-88512, gammaEBV4-88514, gammaEBV4-88516, gammaEBV4-88527, gammaEBV4-88528, gammaEBV4-88529, gammaEBV4-88597, gammaEBV4-88834, gammaEBV4-8901, gammaEBV4-89231, gammaEBV4-89456, gammaEBV4-9007, gammaEBV4-90140, gammaEBV4-90282, gammaEBV4-90294, gammaEBV4-90298, gammaEBV4-90302, gammaEBV4-90304, gammaEBV4-90308, gammaEBV4-90311, gammaEBV4-90312, gammaEBV4-90313, gammaEBV4-90315, gammaEBV4-90317, gammaEBV4-90319, gammaEBV4-90320, gammaEBV4-90321, gammaEBV4-90322, gammaEBV4-90324, gammaEBV4-90332, gammaEBV4-90339, gammaEBV4-90340, gammaEBV4-90341, gammaEBV4-90342, gammaEBV4-90343, gammaEBV4-90347, gammaEBV4-90348, gammaEBV4-90349, gammaEBV4-90350, gammaEBV4-90351, gammaEBV4-90352, gammaEBV4-90353, gammaEBV4-90354, gammaEBV4-90355, gammaEBV4-90356, gammaEBV4-90357, gammaEBV4-90358, gammaEBV4-90359, gammaEBV4-90361, gammaEBV4-90370, gammaEBV4-90371, gammaEBV4-90372, gammaEBV4-90373, gammaEBV4-90376, gammaEBV4-90378, gammaEBV4-91150, gammaEBV4-91422, gammaEBV4-91471, gammaEBV4-91480, gammaEBV4-91705, gammaEBV4-93050, gammaEBV4-93159, gammaEBV4-93490, gammaEBV4-9391, gammaEBV4-94085, gammaEBV4-94177, gammaEBV4-94212, gammaEBV4-9426, gammaEBV4-9427, gammaEBV4-9428, gammaEBV4-9430, gammaEBV4-9432, gammaEBV4-9433, gammaEBV4-9434, gammaEBV4-9437, gammaEBV4-9438, gammaEBV4-9439, gammaEBV4-9442, gammaEBV4-9444, gammaEBV4-9446, gammaEBV4-9447, gammaEBV4-94589, gammaEBV4-9518, gammaEBV4-95186, gammaEBV4-95199, gammaEBV4-95240, gammaEBV4-95258, gammaEBV4-95261, gammaEBV4-95588, gammaEBV4-95590, gammaEBV4-95666, gammaEBV4-95906, gammaEBV4-95942, gammaEBV4-95961, gammaEBV4-95963, gammaEBV4-95969, gammaEBV4-95974, gammaEBV4-95983, gammaEBV4-95984, gammaEBV4-95999, gammaEBV4-96000, gammaEBV4-96003, gammaEBV4-96004, gammaEBV4-96006, gammaEBV4-96008, gammaEBV4-96010, gammaEBV4-96024, gammaEBV4-96029, gammaEBV4-96038, gammaEBV4-96055, gammaEBV4-96064, gammaEBV4-96065, gammaEBV4-96070, gammaEBV4-96073, gammaEBV4-96078, gammaEBV4-96096, gammaEBV4-96114, gammaEBV4-96117, gammaEBV4-96118, gammaEBV4-96123, gammaEBV4-96127, gammaEBV4-96128, gammaEBV4-96142, gammaEBV4-96181, gammaEBV4-96193, gammaEBV4-96197, gammaEBV4-96199, gammaEBV4-962, gammaEBV4-96203, gammaEBV4-96205, gammaEBV4-96207, gammaEBV4-96208, gammaEBV4-96240, gammaEBV4-96254, gammaEBV4-96259, gammaEBV4-96266, gammaEBV4-96276, gammaEBV4-96279, gammaEBV4-96280, gammaEBV4-96281, gammaEBV4-96289, gammaEBV4-96296, gammaEBV4-96298, gammaEBV4-96302, gammaEBV4-96306, gammaEBV4-96310, gammaEBV4-96313, gammaEBV4-96314, gammaEBV4-96315, gammaEBV4-96316, gammaEBV4-96319, gammaEBV4-96328, gammaEBV4-96333, gammaEBV4-96334, gammaEBV4-96337, gammaEBV4-96346, gammaEBV4-96349, gammaEBV4-96357, gammaEBV4-96364, gammaEBV4-96365, gammaEBV4-96367, gammaEBV4-96370, gammaEBV4-96373, gammaEBV4-96378, gammaEBV4-96379, gammaEBV4-96380, gammaEBV4-96382, gammaEBV4-96387, gammaEBV4-96388, gammaEBV4-96394, gammaEBV4-96396, gammaEBV4-96399, gammaEBV4-96400, gammaEBV4-96403, gammaEBV4-96405, gammaEBV4-96406, gammaEBV4-96410, gammaEBV4-96414, gammaEBV4-96415, gammaEBV4-96416, gammaEBV4-96417, gammaEBV4-96419, gammaEBV4-96422, gammaEBV4-96424, gammaEBV4-96425, gammaEBV4-96430, gammaEBV4-96436, gammaEBV4-96440, gammaEBV4-96442, gammaEBV4-96445, gammaEBV4-96446, gammaEBV4-96459, gammaEBV4-96462, gammaEBV4-96464, gammaEBV4-96466, gammaEBV4-96467, gammaEBV4-96468, gammaEBV4-96470, gammaEBV4-96472, gammaEBV4-96473, gammaEBV4-96481, gammaEBV4-96505, gammaEBV4-96521, gammaEBV4-96522, gammaEBV4-96542, gammaEBV4-96546, gammaEBV4-96554, gammaEBV4-96565, gammaEBV4-96566, gammaEBV4-96569, gammaEBV4-96575, gammaEBV4-96582, gammaEBV4-96586, gammaEBV4-96587, gammaEBV4-96588, gammaEBV4-96591, gammaEBV4-96592, gammaEBV4-96594, gammaEBV4-96595, gammaEBV4-96596, gammaEBV4-96598, gammaEBV4-96599, gammaEBV4-96601, gammaEBV4-96604, gammaEBV4-96607, gammaEBV4-96618, gammaEBV4-96622, gammaEBV4-96633, gammaEBV4-96634, gammaEBV4-96636, gammaEBV4-96637, gammaEBV4-96638, gammaEBV4-96640, gammaEBV4-96653, gammaEBV4-96675, gammaEBV4-96677, gammaEBV4-96685, gammaEBV4-96701, gammaEBV4-96703, gammaEBV4-96714, gammaEBV4-96719, gammaEBV4-96722, gammaEBV4-9679, gammaEBV4-96796, gammaEBV4-9712, gammaEBV4-97303, gammaEBV4-97310, gammaEBV4-97457, gammaEBV4-97485, gammaEBV4-97584, gammaEBV4-97632, gammaEBV4-98321, gammaEBV4-98485, gammaEBV4-98607, gammaEBV4-98649, gammaEBV4-98857, gammaEBV4-99257, gammaEBV4-99261, gammaEBV4-99275, gammaEBV4-99399, gammaEBV4-99402, gammaEBV4-99405, gammaEBV4-99422, gammaEBV4-99423, gammaEBV4-99440, gammaEBV4-99441, gammaEBV4-99535

Sheet: IM

The following worksheet contains very long list fields. It is rendered row-by-row to preserve complete cell contents without clipping.

Row 2: gene = BALF1

gene: BALF1

sample: IM10, IM12, IM13, IM5

pos: gammaEBV4-162480, gammaEBV4-162703, gammaEBV4-162758, gammaEBV4-163569, gammaEBV4-163668, gammaEBV4-163804, gammaEBV4-163829, gammaEBV4-164100, gammaEBV4-164161, gammaEBV4-164382, gammaEBV4-164387, gammaEBV4-164551, gammaEBV4-164586, gammaEBV4-164676, gammaEBV4-164692, gammaEBV4-164787, gammaEBV4-164982, gammaEBV4-165051, gammaEBV4-165155, gammaEBV4-165639, gammaEBV4-165807, gammaEBV4-166017, gammaEBV4-166116, gammaEBV4-166237, gammaEBV4-166370, gammaEBV4-166656, gammaEBV4-166695, gammaEBV4-166813

Row 3: gene = BALF2

gene: BALF2

sample: IM10, IM12, IM5

pos: gammaEBV4-159158, gammaEBV4-159376, gammaEBV4-160788, gammaEBV4-161004, gammaEBV4-161324, gammaEBV4-161348, gammaEBV4-161445, gammaEBV4-161533, gammaEBV4-162029, gammaEBV4-162140, gammaEBV4-162204, gammaEBV4-162480, gammaEBV4-162703, gammaEBV4-162758, gammaEBV4-163569, gammaEBV4-163668, gammaEBV4-163804, gammaEBV4-163829, gammaEBV4-164100, gammaEBV4-164161, gammaEBV4-164382, gammaEBV4-164387, gammaEBV4-164551, gammaEBV4-164586, gammaEBV4-164676, gammaEBV4-164692, gammaEBV4-164787, gammaEBV4-164982, gammaEBV4-165051, gammaEBV4-165155, gammaEBV4-165639, gammaEBV4-165807, gammaEBV4-166017, gammaEBV4-166116, gammaEBV4-166237

Row 4: gene = BALF3

gene: BALF3

sample: IM12

pos: gammaEBV4-157058, gammaEBV4-157364, gammaEBV4-157434, gammaEBV4-157853, gammaEBV4-159158, gammaEBV4-159376, gammaEBV4-160788, gammaEBV4-161004, gammaEBV4-161324, gammaEBV4-161348, gammaEBV4-161445, gammaEBV4-161533, gammaEBV4-162029, gammaEBV4-162140, gammaEBV4-162204, gammaEBV4-162480, gammaEBV4-162703, gammaEBV4-162758

Row 5: gene = BALF5

gene: BALF5

sample: IM6

pos: gammaEBV4-151271, gammaEBV4-151272, gammaEBV4-152545, gammaEBV4-152557, gammaEBV4-152613, gammaEBV4-153004, gammaEBV4-153077, gammaEBV4-153117, gammaEBV4-153180, gammaEBV4-153617, gammaEBV4-153639, gammaEBV4-153725, gammaEBV4-153763, gammaEBV4-153860, gammaEBV4-153864, gammaEBV4-153952, gammaEBV4-154055, gammaEBV4-154299, gammaEBV4-154429, gammaEBV4-154453, gammaEBV4-154654, gammaEBV4-154720, gammaEBV4-154982, gammaEBV4-155166, gammaEBV4-155263, gammaEBV4-155584, gammaEBV4-155832, gammaEBV4-156147, gammaEBV4-156363, gammaEBV4-156375, gammaEBV4-156562, gammaEBV4-156606, gammaEBV4-157058, gammaEBV4-157364, gammaEBV4-157434, gammaEBV4-157853

Row 6: gene = BARF1

gene: BARF1

sample: IM10, IM12, IM13, IM5

pos: gammaEBV4-163569, gammaEBV4-163668, gammaEBV4-163804, gammaEBV4-163829, gammaEBV4-164100, gammaEBV4-164161, gammaEBV4-164382, gammaEBV4-164387, gammaEBV4-164551, gammaEBV4-164586, gammaEBV4-164676, gammaEBV4-164692, gammaEBV4-164787, gammaEBV4-164982, gammaEBV4-165051, gammaEBV4-165155, gammaEBV4-165639, gammaEBV4-165807, gammaEBV4-166017, gammaEBV4-166116, gammaEBV4-166237, gammaEBV4-166370, gammaEBV4-166656, gammaEBV4-166695, gammaEBV4-166813, gammaEBV4-167362, gammaEBV4-167370, gammaEBV4-167399, gammaEBV4-167404, gammaEBV4-167437, gammaEBV4-167488, gammaEBV4-167506

Row 7: gene = BBLF2/BBLF3

gene: BBLF2/BBLF3

sample: IM12

pos: gammaEBV4-102548, gammaEBV4-102833, gammaEBV4-102972, gammaEBV4-103167, gammaEBV4-103346, gammaEBV4-103649, gammaEBV4-103838, gammaEBV4-104080, gammaEBV4-104243, gammaEBV4-104986, gammaEBV4-104994, gammaEBV4-105325, gammaEBV4-105501, gammaEBV4-105542, gammaEBV4-105598, gammaEBV4-106045, gammaEBV4-106231, gammaEBV4-106388, gammaEBV4-106430, gammaEBV4-107019, gammaEBV4-107473, gammaEBV4-107738, gammaEBV4-108135, gammaEBV4-108164, gammaEBV4-108165, gammaEBV4-108219, gammaEBV4-108226, gammaEBV4-108228

Row 8: gene = BBLF4

gene: BBLF4

sample: IM13, IM7

pos: gammaEBV4-100012, gammaEBV4-100015, gammaEBV4-100017, gammaEBV4-100032, gammaEBV4-100470, gammaEBV4-100929, gammaEBV4-101078, gammaEBV4-101090, gammaEBV4-101143, gammaEBV4-101244, gammaEBV4-101896, gammaEBV4-101996, gammaEBV4-102132, gammaEBV4-102279, gammaEBV4-102548, gammaEBV4-102833, gammaEBV4-102972, gammaEBV4-103167, gammaEBV4-103346, gammaEBV4-103649, gammaEBV4-103838, gammaEBV4-97584, gammaEBV4-97632, gammaEBV4-98321, gammaEBV4-98485, gammaEBV4-98607, gammaEBV4-98649, gammaEBV4-98857, gammaEBV4-99257, gammaEBV4-99261, gammaEBV4-99275, gammaEBV4-99399, gammaEBV4-99402, gammaEBV4-99405, gammaEBV4-99422, gammaEBV4-99423, gammaEBV4-99440, gammaEBV4-99441, gammaEBV4-99535

Row 9: gene = BDLF3

gene: BDLF3

sample: IM7

pos: gammaEBV4-116738, gammaEBV4-117372, gammaEBV4-117462, gammaEBV4-117583, gammaEBV4-117880, gammaEBV4-117936, gammaEBV4-117941, gammaEBV4-118378, gammaEBV4-118380, gammaEBV4-118437, gammaEBV4-118443, gammaEBV4-118497, gammaEBV4-118554, gammaEBV4-118559, gammaEBV4-119450, gammaEBV4-119719, gammaEBV4-119738, gammaEBV4-119764, gammaEBV4-119766, gammaEBV4-120040

Row 10: gene = BFLF1

gene: BFLF1

sample: IM7, IM8

pos: gammaEBV4-43009, gammaEBV4-43140, gammaEBV4-43141, gammaEBV4-43142, gammaEBV4-43145, gammaEBV4-43146, gammaEBV4-43373, gammaEBV4-43465, gammaEBV4-43477, gammaEBV4-43509, gammaEBV4-43624, gammaEBV4-43684, gammaEBV4-43981, gammaEBV4-44109, gammaEBV4-44155, gammaEBV4-44242, gammaEBV4-44658, gammaEBV4-45678, gammaEBV4-45790, gammaEBV4-45805, gammaEBV4-45860, gammaEBV4-46042, gammaEBV4-46062, gammaEBV4-46257, gammaEBV4-46472, gammaEBV4-46706, gammaEBV4-46912, gammaEBV4-47220, gammaEBV4-47236, gammaEBV4-47240, gammaEBV4-47908, gammaEBV4-47955, gammaEBV4-48227

Row 11: gene = BFLF2

gene: BFLF2

sample: IM7, IM8

pos: gammaEBV4-41794, gammaEBV4-41821, gammaEBV4-43009, gammaEBV4-43140, gammaEBV4-43141, gammaEBV4-43142, gammaEBV4-43145, gammaEBV4-43146, gammaEBV4-43373, gammaEBV4-43465, gammaEBV4-43477, gammaEBV4-43509, gammaEBV4-43624, gammaEBV4-43684, gammaEBV4-43981, gammaEBV4-44109, gammaEBV4-44155, gammaEBV4-44242, gammaEBV4-44658, gammaEBV4-45678, gammaEBV4-45790, gammaEBV4-45805, gammaEBV4-45860, gammaEBV4-46042, gammaEBV4-46062, gammaEBV4-46257, gammaEBV4-46472

Row 12: gene = BFRF3

gene: BFRF3

sample: IM12

pos: gammaEBV4-47220, gammaEBV4-47236, gammaEBV4-47240, gammaEBV4-47908, gammaEBV4-47955, gammaEBV4-48227, gammaEBV4-48251, gammaEBV4-48335, gammaEBV4-48519, gammaEBV4-48797, gammaEBV4-48983, gammaEBV4-49102, gammaEBV4-49719, gammaEBV4-50133, gammaEBV4-50242, gammaEBV4-51166, gammaEBV4-51500, gammaEBV4-51571

Row 13: gene = BGLF4

gene: BGLF4

sample: IM10, IM11, IM12

pos: gammaEBV4-108135, gammaEBV4-108164, gammaEBV4-108165, gammaEBV4-108219, gammaEBV4-108226, gammaEBV4-108228, gammaEBV4-109037, gammaEBV4-109074, gammaEBV4-110375, gammaEBV4-110393, gammaEBV4-110468, gammaEBV4-110538, gammaEBV4-110756, gammaEBV4-110937, gammaEBV4-111238, gammaEBV4-112229, gammaEBV4-112312, gammaEBV4-112741, gammaEBV4-113146

Row 14: gene = BGLF5

gene: BGLF5

sample: IM10, IM11, IM12

pos: gammaEBV4-107019, gammaEBV4-107473, gammaEBV4-107738, gammaEBV4-108135, gammaEBV4-108164, gammaEBV4-108165, gammaEBV4-108219, gammaEBV4-108226, gammaEBV4-108228, gammaEBV4-109037, gammaEBV4-109074, gammaEBV4-110375, gammaEBV4-110393, gammaEBV4-110468, gammaEBV4-110538, gammaEBV4-110756, gammaEBV4-110937, gammaEBV4-111238

Row 15: gene = BHLF1

gene: BHLF1

sample: IM1, IM2, IM10, IM11, IM12, IM13, IM14, IM15, IM16, IM17, IM4, IM5, IM6, IM7, IM8, IM9

pos: gammaEBV4-36307, gammaEBV4-36315, gammaEBV4-36322, gammaEBV4-36355, gammaEBV4-36356, gammaEBV4-36360, gammaEBV4-36368, gammaEBV4-36389, gammaEBV4-36390, gammaEBV4-36391, gammaEBV4-36393, gammaEBV4-36394, gammaEBV4-36397, gammaEBV4-36399, gammaEBV4-36401, gammaEBV4-36402, gammaEBV4-36403, gammaEBV4-36405, gammaEBV4-36406, gammaEBV4-36408, gammaEBV4-36410, gammaEBV4-36411, gammaEBV4-36414, gammaEBV4-36415, gammaEBV4-36417, gammaEBV4-36420, gammaEBV4-36421, gammaEBV4-36423, gammaEBV4-36424, gammaEBV4-36425, gammaEBV4-36429, gammaEBV4-36432, gammaEBV4-36433, gammaEBV4-36435, gammaEBV4-36436, gammaEBV4-36437, gammaEBV4-36438, gammaEBV4-36439, gammaEBV4-36441, gammaEBV4-36442, gammaEBV4-36445, gammaEBV4-36446, gammaEBV4-36447, gammaEBV4-36448, gammaEBV4-36449, gammaEBV4-36450, gammaEBV4-36451, gammaEBV4-36453, gammaEBV4-36454, gammaEBV4-36455, gammaEBV4-36457, gammaEBV4-36458, gammaEBV4-36459, gammaEBV4-36460, gammaEBV4-36462, gammaEBV4-36463, gammaEBV4-36464, gammaEBV4-36465, gammaEBV4-36466, gammaEBV4-36468, gammaEBV4-36469, gammaEBV4-36470, gammaEBV4-36472, gammaEBV4-36475, gammaEBV4-36476, gammaEBV4-36477, gammaEBV4-36478, gammaEBV4-36479, gammaEBV4-36481, gammaEBV4-36482, gammaEBV4-36483, gammaEBV4-36486, gammaEBV4-36487, gammaEBV4-36489, gammaEBV4-36490, gammaEBV4-36491, gammaEBV4-36492, gammaEBV4-36495, gammaEBV4-36497, gammaEBV4-36498, gammaEBV4-36499, gammaEBV4-36501, gammaEBV4-36502, gammaEBV4-36507, gammaEBV4-36521, gammaEBV4-36528, gammaEBV4-36543, gammaEBV4-36558, gammaEBV4-36564, gammaEBV4-36710, gammaEBV4-36712, gammaEBV4-36745, gammaEBV4-36781, gammaEBV4-36801, gammaEBV4-36816, gammaEBV4-36834, gammaEBV4-36855, gammaEBV4-36856, gammaEBV4-36860, gammaEBV4-36865, gammaEBV4-36869, gammaEBV4-36882, gammaEBV4-36885, gammaEBV4-36897, gammaEBV4-36924, gammaEBV4-36940, gammaEBV4-36942, gammaEBV4-36946, gammaEBV4-36947, gammaEBV4-36961, gammaEBV4-36965, gammaEBV4-36993, gammaEBV4-37009, gammaEBV4-37019, gammaEBV4-37033, gammaEBV4-37044, gammaEBV4-37087, gammaEBV4-37113, gammaEBV4-37116, gammaEBV4-37130, gammaEBV4-37270, gammaEBV4-37490, gammaEBV4-37678, gammaEBV4-37924, gammaEBV4-37979, gammaEBV4-38989, gammaEBV4-39482, gammaEBV4-39873, gammaEBV4-39899, gammaEBV4-39903, gammaEBV4-39908, gammaEBV4-39915, gammaEBV4-39925, gammaEBV4-40194, gammaEBV4-40213, gammaEBV4-40219, gammaEBV4-40220, gammaEBV4-40224, gammaEBV4-40225, gammaEBV4-40263, gammaEBV4-40282, gammaEBV4-41499, gammaEBV4-41553, gammaEBV4-41648, gammaEBV4-41794, gammaEBV4-41821

Row 16: gene = BLLF1

gene: BLLF1

sample: IM15, IM4, IM5

pos: gammaEBV4-75292, gammaEBV4-75357, gammaEBV4-75429, gammaEBV4-75555, gammaEBV4-75694, gammaEBV4-75699, gammaEBV4-76014, gammaEBV4-76449, gammaEBV4-76489, gammaEBV4-76525, gammaEBV4-76598, gammaEBV4-76943, gammaEBV4-77179, gammaEBV4-77304, gammaEBV4-77373, gammaEBV4-77401, gammaEBV4-77404, gammaEBV4-77419, gammaEBV4-77613, gammaEBV4-77647, gammaEBV4-77672, gammaEBV4-77826, gammaEBV4-78055, gammaEBV4-78268, gammaEBV4-78767, gammaEBV4-78969, gammaEBV4-79898, gammaEBV4-80010, gammaEBV4-80158, gammaEBV4-80180, gammaEBV4-80333, gammaEBV4-80380, gammaEBV4-80419, gammaEBV4-80444, gammaEBV4-81096, gammaEBV4-81378, gammaEBV4-81601, gammaEBV4-81671, gammaEBV4-81722, gammaEBV4-81794, gammaEBV4-81818

Row 17: gene = BLRF1

gene: BLRF1

sample: IM4, IM5

pos: gammaEBV4-74856, gammaEBV4-74882, gammaEBV4-75292, gammaEBV4-75357, gammaEBV4-75429, gammaEBV4-75555, gammaEBV4-75694, gammaEBV4-75699, gammaEBV4-76014, gammaEBV4-76449, gammaEBV4-76489, gammaEBV4-76525, gammaEBV4-76598, gammaEBV4-76943, gammaEBV4-77179, gammaEBV4-77304, gammaEBV4-77373, gammaEBV4-77401, gammaEBV4-77404, gammaEBV4-77419, gammaEBV4-77613, gammaEBV4-77647, gammaEBV4-77672, gammaEBV4-77826, gammaEBV4-78055, gammaEBV4-78268

Row 18: gene = BMRF1

gene: BMRF1

sample: IM12, IM16

pos: gammaEBV4-65685, gammaEBV4-65718, gammaEBV4-65785, gammaEBV4-65950, gammaEBV4-66061, gammaEBV4-66069, gammaEBV4-66363, gammaEBV4-66405, gammaEBV4-66612, gammaEBV4-66809, gammaEBV4-67279, gammaEBV4-67485, gammaEBV4-67505, gammaEBV4-67749, gammaEBV4-68057, gammaEBV4-68261, gammaEBV4-68279, gammaEBV4-68497, gammaEBV4-68583, gammaEBV4-68589, gammaEBV4-68612, gammaEBV4-68646, gammaEBV4-68657, gammaEBV4-68729, gammaEBV4-68748, gammaEBV4-69148, gammaEBV4-69192, gammaEBV4-69276, gammaEBV4-69611, gammaEBV4-69904, gammaEBV4-69950, gammaEBV4-70330, gammaEBV4-70538

Row 19: gene = BNRF1

gene: BNRF1

sample: IM2, IM7

pos: gammaEBV4-2403, gammaEBV4-2768, gammaEBV4-2769, gammaEBV4-2958, gammaEBV4-3452, gammaEBV4-3640, gammaEBV4-3720, gammaEBV4-3754, gammaEBV4-3758, gammaEBV4-3775, gammaEBV4-4082, gammaEBV4-4944, gammaEBV4-5080, gammaEBV4-5182, gammaEBV4-5406, gammaEBV4-5665, gammaEBV4-5739, gammaEBV4-5751, gammaEBV4-5758, gammaEBV4-5762, gammaEBV4-5793, gammaEBV4-5796, gammaEBV4-5798, gammaEBV4-5799, gammaEBV4-5802, gammaEBV4-5803, gammaEBV4-5804, gammaEBV4-5805, gammaEBV4-5807, gammaEBV4-5808, gammaEBV4-5809, gammaEBV4-5810, gammaEBV4-7019, gammaEBV4-7035, gammaEBV4-7210, gammaEBV4-7253, gammaEBV4-7691

Row 20: gene = BOLF1

gene: BOLF1

sample: IM12, IM7

pos: gammaEBV4-57429, gammaEBV4-57632, gammaEBV4-57708, gammaEBV4-57740, gammaEBV4-57806, gammaEBV4-57842, gammaEBV4-57869, gammaEBV4-57870, gammaEBV4-57879, gammaEBV4-57880, gammaEBV4-57896, gammaEBV4-57897, gammaEBV4-58047, gammaEBV4-58301, gammaEBV4-58407, gammaEBV4-58634, gammaEBV4-58692, gammaEBV4-58785, gammaEBV4-58867, gammaEBV4-59115, gammaEBV4-59448, gammaEBV4-59672, gammaEBV4-59799, gammaEBV4-59923, gammaEBV4-59992, gammaEBV4-60095, gammaEBV4-60374, gammaEBV4-60398, gammaEBV4-61987, gammaEBV4-62009, gammaEBV4-62182, gammaEBV4-62247, gammaEBV4-62391, gammaEBV4-62449, gammaEBV4-62499, gammaEBV4-62677, gammaEBV4-62690, gammaEBV4-62774, gammaEBV4-62778, gammaEBV4-62916, gammaEBV4-63356, gammaEBV4-63580, gammaEBV4-63692, gammaEBV4-64008, gammaEBV4-64199, gammaEBV4-64224, gammaEBV4-64324, gammaEBV4-64571, gammaEBV4-64795

Row 21: gene = BPLF1

gene: BPLF1

sample: IM12, IM14

pos: gammaEBV4-47908, gammaEBV4-47955, gammaEBV4-48227, gammaEBV4-48251, gammaEBV4-48335, gammaEBV4-48519, gammaEBV4-48797, gammaEBV4-48983, gammaEBV4-49102, gammaEBV4-49719, gammaEBV4-50133, gammaEBV4-50242, gammaEBV4-51166, gammaEBV4-51500, gammaEBV4-51571, gammaEBV4-52091, gammaEBV4-52205, gammaEBV4-52211, gammaEBV4-52227, gammaEBV4-52259, gammaEBV4-52372, gammaEBV4-52814, gammaEBV4-52837, gammaEBV4-52952, gammaEBV4-53724, gammaEBV4-53902, gammaEBV4-54149, gammaEBV4-54243, gammaEBV4-54255, gammaEBV4-54491, gammaEBV4-54802, gammaEBV4-54921, gammaEBV4-55000, gammaEBV4-55730, gammaEBV4-55780, gammaEBV4-55793, gammaEBV4-55811, gammaEBV4-55869, gammaEBV4-55887, gammaEBV4-55914, gammaEBV4-56237, gammaEBV4-56566, gammaEBV4-56866, gammaEBV4-57429, gammaEBV4-57632, gammaEBV4-57708, gammaEBV4-57740, gammaEBV4-57806, gammaEBV4-57842, gammaEBV4-57869, gammaEBV4-57870, gammaEBV4-57879, gammaEBV4-57880, gammaEBV4-57896, gammaEBV4-57897, gammaEBV4-58047, gammaEBV4-58301, gammaEBV4-58407, gammaEBV4-58634, gammaEBV4-58692, gammaEBV4-58785, gammaEBV4-58867, gammaEBV4-59115, gammaEBV4-59448, gammaEBV4-59672, gammaEBV4-59799, gammaEBV4-59923, gammaEBV4-59992, gammaEBV4-60095, gammaEBV4-60374, gammaEBV4-60398

Row 22: gene = BSLF1

gene: BSLF1

sample: IM11, IM5

pos: gammaEBV4-70330, gammaEBV4-70538, gammaEBV4-71139, gammaEBV4-71229, gammaEBV4-71292, gammaEBV4-71344, gammaEBV4-71546, gammaEBV4-71618, gammaEBV4-71671, gammaEBV4-72053, gammaEBV4-72289, gammaEBV4-72560, gammaEBV4-72580, gammaEBV4-72690, gammaEBV4-72723, gammaEBV4-72979, gammaEBV4-73029, gammaEBV4-73305, gammaEBV4-73366, gammaEBV4-73378, gammaEBV4-73577, gammaEBV4-73713, gammaEBV4-73903, gammaEBV4-73989, gammaEBV4-74183, gammaEBV4-74250, gammaEBV4-74856, gammaEBV4-74882, gammaEBV4-75292, gammaEBV4-75357, gammaEBV4-75429, gammaEBV4-75555, gammaEBV4-75694, gammaEBV4-75699, gammaEBV4-76014, gammaEBV4-76449, gammaEBV4-76489, gammaEBV4-76525

Row 23: gene = BTRF1

gene: BTRF1

sample: IM11

pos: gammaEBV4-125786, gammaEBV4-125876, gammaEBV4-125881, gammaEBV4-126135, gammaEBV4-126219, gammaEBV4-126402, gammaEBV4-127266, gammaEBV4-127347, gammaEBV4-127645, gammaEBV4-127663, gammaEBV4-127669, gammaEBV4-127827, gammaEBV4-128010, gammaEBV4-128770, gammaEBV4-128837, gammaEBV4-129213, gammaEBV4-129395, gammaEBV4-130293, gammaEBV4-130302, gammaEBV4-130543, gammaEBV4-130568, gammaEBV4-130569

Row 24: gene = BZLF1

gene: BZLF1

sample: IM1, IM2, IM10, IM12, IM14, IM15, IM5, IM6, IM7, IM8

pos: gammaEBV4-87870, gammaEBV4-87967, gammaEBV4-88158, gammaEBV4-88365, gammaEBV4-88383, gammaEBV4-88385, gammaEBV4-88388, gammaEBV4-88390, gammaEBV4-88393, gammaEBV4-88397, gammaEBV4-88400, gammaEBV4-88401, gammaEBV4-88402, gammaEBV4-88405, gammaEBV4-88409, gammaEBV4-88410, gammaEBV4-88411, gammaEBV4-88412, gammaEBV4-88413, gammaEBV4-88414, gammaEBV4-88415, gammaEBV4-88416, gammaEBV4-88417, gammaEBV4-88419, gammaEBV4-88420, gammaEBV4-88421, gammaEBV4-88423, gammaEBV4-88424, gammaEBV4-88425, gammaEBV4-88426, gammaEBV4-88427, gammaEBV4-88429, gammaEBV4-88431, gammaEBV4-88432, gammaEBV4-88433, gammaEBV4-88435, gammaEBV4-88436, gammaEBV4-88437, gammaEBV4-88438, gammaEBV4-88439, gammaEBV4-88440, gammaEBV4-88442, gammaEBV4-88444, gammaEBV4-88445, gammaEBV4-88446, gammaEBV4-88447, gammaEBV4-88448, gammaEBV4-88449, gammaEBV4-88450, gammaEBV4-88451, gammaEBV4-88452, gammaEBV4-88453, gammaEBV4-88454, gammaEBV4-88455, gammaEBV4-88456, gammaEBV4-88457, gammaEBV4-88458, gammaEBV4-88459, gammaEBV4-88462, gammaEBV4-88463, gammaEBV4-88464, gammaEBV4-88465, gammaEBV4-88466, gammaEBV4-88467, gammaEBV4-88469, gammaEBV4-88470, gammaEBV4-88471, gammaEBV4-88472, gammaEBV4-88474, gammaEBV4-88476, gammaEBV4-88478, gammaEBV4-88483, gammaEBV4-88486, gammaEBV4-88495, gammaEBV4-88497, gammaEBV4-88498, gammaEBV4-88499, gammaEBV4-88500, gammaEBV4-88501, gammaEBV4-88502, gammaEBV4-88503, gammaEBV4-88504, gammaEBV4-88506, gammaEBV4-88507, gammaEBV4-88508, gammaEBV4-88511, gammaEBV4-88512, gammaEBV4-88514, gammaEBV4-88516, gammaEBV4-88527, gammaEBV4-88528, gammaEBV4-88529, gammaEBV4-88597, gammaEBV4-88834, gammaEBV4-89231, gammaEBV4-89456, gammaEBV4-90140, gammaEBV4-90282, gammaEBV4-90294, gammaEBV4-90298, gammaEBV4-90302, gammaEBV4-90304, gammaEBV4-90308, gammaEBV4-90311, gammaEBV4-90312, gammaEBV4-90313, gammaEBV4-90315, gammaEBV4-90317, gammaEBV4-90319, gammaEBV4-90320, gammaEBV4-90321, gammaEBV4-90322, gammaEBV4-90324, gammaEBV4-90332, gammaEBV4-90339, gammaEBV4-90340, gammaEBV4-90341, gammaEBV4-90342, gammaEBV4-90343, gammaEBV4-90347, gammaEBV4-90348, gammaEBV4-90349, gammaEBV4-90350, gammaEBV4-90351, gammaEBV4-90352, gammaEBV4-90353, gammaEBV4-90354, gammaEBV4-90355, gammaEBV4-90356, gammaEBV4-90357, gammaEBV4-90358, gammaEBV4-90359, gammaEBV4-90361, gammaEBV4-90370, gammaEBV4-90371, gammaEBV4-90372, gammaEBV4-90373, gammaEBV4-90376, gammaEBV4-90378, gammaEBV4-91150, gammaEBV4-91422, gammaEBV4-91471, gammaEBV4-91480, gammaEBV4-91705

Row 25: gene = BZLF2

gene: BZLF2

sample: IM1, IM2, IM10, IM12, IM14, IM15, IM5, IM6, IM7, IM8

pos: gammaEBV4-87597, gammaEBV4-87818, gammaEBV4-87870, gammaEBV4-87967, gammaEBV4-88158, gammaEBV4-88365, gammaEBV4-88383, gammaEBV4-88385, gammaEBV4-88388, gammaEBV4-88390, gammaEBV4-88393, gammaEBV4-88397, gammaEBV4-88400, gammaEBV4-88401, gammaEBV4-88402, gammaEBV4-88405, gammaEBV4-88409, gammaEBV4-88410, gammaEBV4-88411, gammaEBV4-88412, gammaEBV4-88413, gammaEBV4-88414, gammaEBV4-88415, gammaEBV4-88416, gammaEBV4-88417, gammaEBV4-88419, gammaEBV4-88420, gammaEBV4-88421, gammaEBV4-88423, gammaEBV4-88424, gammaEBV4-88425, gammaEBV4-88426, gammaEBV4-88427, gammaEBV4-88429, gammaEBV4-88431, gammaEBV4-88432, gammaEBV4-88433, gammaEBV4-88435, gammaEBV4-88436, gammaEBV4-88437, gammaEBV4-88438, gammaEBV4-88439, gammaEBV4-88440, gammaEBV4-88442, gammaEBV4-88444, gammaEBV4-88445, gammaEBV4-88446, gammaEBV4-88447, gammaEBV4-88448, gammaEBV4-88449, gammaEBV4-88450, gammaEBV4-88451, gammaEBV4-88452, gammaEBV4-88453, gammaEBV4-88454, gammaEBV4-88455, gammaEBV4-88456, gammaEBV4-88457, gammaEBV4-88458, gammaEBV4-88459, gammaEBV4-88462, gammaEBV4-88463, gammaEBV4-88464, gammaEBV4-88465, gammaEBV4-88466, gammaEBV4-88467, gammaEBV4-88469, gammaEBV4-88470, gammaEBV4-88471, gammaEBV4-88472, gammaEBV4-88474, gammaEBV4-88476, gammaEBV4-88478, gammaEBV4-88483, gammaEBV4-88486, gammaEBV4-88495, gammaEBV4-88497, gammaEBV4-88498, gammaEBV4-88499, gammaEBV4-88500, gammaEBV4-88501, gammaEBV4-88502, gammaEBV4-88503, gammaEBV4-88504, gammaEBV4-88506, gammaEBV4-88507, gammaEBV4-88508, gammaEBV4-88511, gammaEBV4-88512, gammaEBV4-88514, gammaEBV4-88516, gammaEBV4-88527, gammaEBV4-88528, gammaEBV4-88529, gammaEBV4-88597, gammaEBV4-88834, gammaEBV4-89231, gammaEBV4-89456, gammaEBV4-90140, gammaEBV4-90282, gammaEBV4-90294, gammaEBV4-90298, gammaEBV4-90302, gammaEBV4-90304, gammaEBV4-90308, gammaEBV4-90311, gammaEBV4-90312, gammaEBV4-90313, gammaEBV4-90315, gammaEBV4-90317, gammaEBV4-90319, gammaEBV4-90320, gammaEBV4-90321, gammaEBV4-90322, gammaEBV4-90324, gammaEBV4-90332, gammaEBV4-90339, gammaEBV4-90340, gammaEBV4-90341, gammaEBV4-90342, gammaEBV4-90343, gammaEBV4-90347, gammaEBV4-90348, gammaEBV4-90349, gammaEBV4-90350, gammaEBV4-90351, gammaEBV4-90352, gammaEBV4-90353, gammaEBV4-90354, gammaEBV4-90355, gammaEBV4-90356, gammaEBV4-90357, gammaEBV4-90358, gammaEBV4-90359, gammaEBV4-90361, gammaEBV4-90370, gammaEBV4-90371, gammaEBV4-90372, gammaEBV4-90373, gammaEBV4-90376, gammaEBV4-90378, gammaEBV4-91150, gammaEBV4-91422, gammaEBV4-91471, gammaEBV4-91480, gammaEBV4-91705

Row 26: gene = BaRF1

gene: BaRF1

sample: IM12, IM16

pos: gammaEBV4-64795, gammaEBV4-64976, gammaEBV4-65127, gammaEBV4-65205, gammaEBV4-65433, gammaEBV4-65450, gammaEBV4-65685, gammaEBV4-65718, gammaEBV4-65785, gammaEBV4-65950, gammaEBV4-66061, gammaEBV4-66069, gammaEBV4-66363, gammaEBV4-66405, gammaEBV4-66612, gammaEBV4-66809, gammaEBV4-67279, gammaEBV4-67485, gammaEBV4-67505, gammaEBV4-67749, gammaEBV4-68057, gammaEBV4-68261, gammaEBV4-68279, gammaEBV4-68497, gammaEBV4-68583, gammaEBV4-68589, gammaEBV4-68612, gammaEBV4-68646, gammaEBV4-68657, gammaEBV4-68729, gammaEBV4-68748, gammaEBV4-69148, gammaEBV4-69192, gammaEBV4-69276

Row 27: gene = BcLF1

gene: BcLF1

sample: IM11, IM12, IM13, IM15, IM5, IM6, IM7, IM9

pos: gammaEBV4-119450, gammaEBV4-119719, gammaEBV4-119738, gammaEBV4-119764, gammaEBV4-119766, gammaEBV4-120040, gammaEBV4-120837, gammaEBV4-121112, gammaEBV4-121131, gammaEBV4-122449, gammaEBV4-122641, gammaEBV4-123006, gammaEBV4-123073, gammaEBV4-123123, gammaEBV4-123468, gammaEBV4-123550, gammaEBV4-123573, gammaEBV4-123594, gammaEBV4-123722, gammaEBV4-123726, gammaEBV4-123773, gammaEBV4-123786, gammaEBV4-123790, gammaEBV4-123855, gammaEBV4-124007, gammaEBV4-124331, gammaEBV4-124333, gammaEBV4-124335, gammaEBV4-124336, gammaEBV4-124337, gammaEBV4-124338, gammaEBV4-124339, gammaEBV4-124340, gammaEBV4-124342, gammaEBV4-124343, gammaEBV4-124344, gammaEBV4-124345, gammaEBV4-124346, gammaEBV4-124347, gammaEBV4-124348, gammaEBV4-124349, gammaEBV4-124350, gammaEBV4-124351, gammaEBV4-124352, gammaEBV4-124356, gammaEBV4-124357, gammaEBV4-124373, gammaEBV4-124681, gammaEBV4-124988, gammaEBV4-125072, gammaEBV4-125106, gammaEBV4-125211, gammaEBV4-125323, gammaEBV4-125330, gammaEBV4-125786, gammaEBV4-125876, gammaEBV4-125881, gammaEBV4-126135, gammaEBV4-126219, gammaEBV4-126402

Row 28: gene = EBNA-1

gene: EBNA-1

sample: IM1, IM2, IM10, IM11, IM12, IM13, IM14, IM15, IM16, IM4, IM5, IM6, IM7, IM8

pos: gammaEBV4-48227, gammaEBV4-48251, gammaEBV4-48335, gammaEBV4-48519, gammaEBV4-48797, gammaEBV4-48983, gammaEBV4-49102, gammaEBV4-49719, gammaEBV4-50133, gammaEBV4-50242, gammaEBV4-51166, gammaEBV4-51500, gammaEBV4-51571, gammaEBV4-52091, gammaEBV4-52205, gammaEBV4-52211, gammaEBV4-52227, gammaEBV4-52259, gammaEBV4-52372, gammaEBV4-52814, gammaEBV4-52837, gammaEBV4-52952, gammaEBV4-53724, gammaEBV4-53902, gammaEBV4-54149, gammaEBV4-54243, gammaEBV4-54255, gammaEBV4-54491, gammaEBV4-54802, gammaEBV4-54921, gammaEBV4-55000, gammaEBV4-55730, gammaEBV4-55780, gammaEBV4-55793, gammaEBV4-55811, gammaEBV4-55869, gammaEBV4-55887, gammaEBV4-55914, gammaEBV4-56237, gammaEBV4-56566, gammaEBV4-56866, gammaEBV4-57429, gammaEBV4-57632, gammaEBV4-57708, gammaEBV4-57740, gammaEBV4-57806, gammaEBV4-57842, gammaEBV4-57869, gammaEBV4-57870, gammaEBV4-57879, gammaEBV4-57880, gammaEBV4-57896, gammaEBV4-57897, gammaEBV4-58047, gammaEBV4-58301, gammaEBV4-58407, gammaEBV4-58634, gammaEBV4-58692, gammaEBV4-58785, gammaEBV4-58867, gammaEBV4-59115, gammaEBV4-59448, gammaEBV4-59672, gammaEBV4-59799, gammaEBV4-59923, gammaEBV4-59992, gammaEBV4-60095, gammaEBV4-60374, gammaEBV4-60398, gammaEBV4-61987, gammaEBV4-62009, gammaEBV4-62182, gammaEBV4-62247, gammaEBV4-62391, gammaEBV4-62449, gammaEBV4-62499, gammaEBV4-62677, gammaEBV4-62690, gammaEBV4-62774, gammaEBV4-62778, gammaEBV4-62916, gammaEBV4-63356, gammaEBV4-63580, gammaEBV4-63692, gammaEBV4-64008, gammaEBV4-64199, gammaEBV4-64224, gammaEBV4-64324, gammaEBV4-64571, gammaEBV4-64795, gammaEBV4-64976, gammaEBV4-65127, gammaEBV4-65205, gammaEBV4-65433, gammaEBV4-65450, gammaEBV4-65685, gammaEBV4-65718, gammaEBV4-65785, gammaEBV4-65950, gammaEBV4-66061, gammaEBV4-66069, gammaEBV4-66363, gammaEBV4-66405, gammaEBV4-66612, gammaEBV4-66809, gammaEBV4-67279, gammaEBV4-67485, gammaEBV4-67505, gammaEBV4-67749, gammaEBV4-68057, gammaEBV4-68261, gammaEBV4-68279, gammaEBV4-68497, gammaEBV4-68583, gammaEBV4-68589, gammaEBV4-68612, gammaEBV4-68646, gammaEBV4-68657, gammaEBV4-68729, gammaEBV4-68748, gammaEBV4-69148, gammaEBV4-69192, gammaEBV4-69276, gammaEBV4-69611, gammaEBV4-69904, gammaEBV4-69950, gammaEBV4-70330, gammaEBV4-70538, gammaEBV4-71139, gammaEBV4-71229, gammaEBV4-71292, gammaEBV4-71344, gammaEBV4-71546, gammaEBV4-71618, gammaEBV4-71671, gammaEBV4-72053, gammaEBV4-72289, gammaEBV4-72560, gammaEBV4-72580, gammaEBV4-72690, gammaEBV4-72723, gammaEBV4-72979, gammaEBV4-73029, gammaEBV4-73305, gammaEBV4-73366, gammaEBV4-73378, gammaEBV4-73577, gammaEBV4-73713, gammaEBV4-73903, gammaEBV4-73989, gammaEBV4-74183, gammaEBV4-74250, gammaEBV4-74856, gammaEBV4-74882, gammaEBV4-75292, gammaEBV4-75357, gammaEBV4-75429, gammaEBV4-75555, gammaEBV4-75694, gammaEBV4-75699, gammaEBV4-76014, gammaEBV4-76449, gammaEBV4-76489, gammaEBV4-76525, gammaEBV4-76598, gammaEBV4-76943, gammaEBV4-77179, gammaEBV4-77304, gammaEBV4-77373, gammaEBV4-77401, gammaEBV4-77404, gammaEBV4-77419, gammaEBV4-77613, gammaEBV4-77647, gammaEBV4-77672, gammaEBV4-77826, gammaEBV4-78055, gammaEBV4-78268, gammaEBV4-78767, gammaEBV4-78969, gammaEBV4-79898, gammaEBV4-80010, gammaEBV4-80158, gammaEBV4-80180, gammaEBV4-80333, gammaEBV4-80380, gammaEBV4-80419, gammaEBV4-80444, gammaEBV4-81096, gammaEBV4-81378, gammaEBV4-81601, gammaEBV4-81671, gammaEBV4-81722, gammaEBV4-81794, gammaEBV4-81818, gammaEBV4-81950, gammaEBV4-81952, gammaEBV4-81957, gammaEBV4-81959, gammaEBV4-82027, gammaEBV4-82041, gammaEBV4-82056, gammaEBV4-82082, gammaEBV4-82089, gammaEBV4-82233, gammaEBV4-82234, gammaEBV4-82250, gammaEBV4-82253, gammaEBV4-82254, gammaEBV4-82259, gammaEBV4-82274, gammaEBV4-82347, gammaEBV4-82356, gammaEBV4-82358, gammaEBV4-82363, gammaEBV4-82366, gammaEBV4-82414, gammaEBV4-82418, gammaEBV4-82557, gammaEBV4-82685, gammaEBV4-82902, gammaEBV4-82911, gammaEBV4-83123, gammaEBV4-83632, gammaEBV4-84936, gammaEBV4-85018, gammaEBV4-85048, gammaEBV4-85131, gammaEBV4-85235, gammaEBV4-85666, gammaEBV4-87597, gammaEBV4-87818, gammaEBV4-87870, gammaEBV4-87967, gammaEBV4-88158, gammaEBV4-88365, gammaEBV4-88383, gammaEBV4-88385, gammaEBV4-88388, gammaEBV4-88390, gammaEBV4-88393, gammaEBV4-88397, gammaEBV4-88400, gammaEBV4-88401, gammaEBV4-88402, gammaEBV4-88405, gammaEBV4-88409, gammaEBV4-88410, gammaEBV4-88411, gammaEBV4-88412, gammaEBV4-88413, gammaEBV4-88414, gammaEBV4-88415, gammaEBV4-88416, gammaEBV4-88417, gammaEBV4-88419, gammaEBV4-88420, gammaEBV4-88421, gammaEBV4-88423, gammaEBV4-88424, gammaEBV4-88425, gammaEBV4-88426, gammaEBV4-88427, gammaEBV4-88429, gammaEBV4-88431, gammaEBV4-88432, gammaEBV4-88433, gammaEBV4-88435, gammaEBV4-88436, gammaEBV4-88437, gammaEBV4-88438, gammaEBV4-88439, gammaEBV4-88440, gammaEBV4-88442, gammaEBV4-88444, gammaEBV4-88445, gammaEBV4-88446, gammaEBV4-88447, gammaEBV4-88448, gammaEBV4-88449, gammaEBV4-88450, gammaEBV4-88451, gammaEBV4-88452, gammaEBV4-88453, gammaEBV4-88454, gammaEBV4-88455, gammaEBV4-88456, gammaEBV4-88457, gammaEBV4-88458, gammaEBV4-88459, gammaEBV4-88462, gammaEBV4-88463, gammaEBV4-88464, gammaEBV4-88465, gammaEBV4-88466, gammaEBV4-88467, gammaEBV4-88469, gammaEBV4-88470, gammaEBV4-88471, gammaEBV4-88472, gammaEBV4-88474, gammaEBV4-88476, gammaEBV4-88478, gammaEBV4-88483, gammaEBV4-88486, gammaEBV4-88495, gammaEBV4-88497, gammaEBV4-88498, gammaEBV4-88499, gammaEBV4-88500, gammaEBV4-88501, gammaEBV4-88502, gammaEBV4-88503, gammaEBV4-88504, gammaEBV4-88506, gammaEBV4-88507, gammaEBV4-88508, gammaEBV4-88511, gammaEBV4-88512, gammaEBV4-88514, gammaEBV4-88516, gammaEBV4-88527, gammaEBV4-88528, gammaEBV4-88529, gammaEBV4-88597, gammaEBV4-88834, gammaEBV4-89231, gammaEBV4-89456, gammaEBV4-90140, gammaEBV4-90282, gammaEBV4-90294, gammaEBV4-90298, gammaEBV4-90302, gammaEBV4-90304, gammaEBV4-90308, gammaEBV4-90311, gammaEBV4-90312, gammaEBV4-90313, gammaEBV4-90315, gammaEBV4-90317, gammaEBV4-90319, gammaEBV4-90320, gammaEBV4-90321, gammaEBV4-90322, gammaEBV4-90324, gammaEBV4-90332, gammaEBV4-90339, gammaEBV4-90340, gammaEBV4-90341, gammaEBV4-90342, gammaEBV4-90343, gammaEBV4-90347, gammaEBV4-90348, gammaEBV4-90349, gammaEBV4-90350, gammaEBV4-90351, gammaEBV4-90352, gammaEBV4-90353, gammaEBV4-90354, gammaEBV4-90355, gammaEBV4-90356, gammaEBV4-90357, gammaEBV4-90358, gammaEBV4-90359, gammaEBV4-90361, gammaEBV4-90370, gammaEBV4-90371, gammaEBV4-90372, gammaEBV4-90373, gammaEBV4-90376, gammaEBV4-90378, gammaEBV4-91150, gammaEBV4-91422, gammaEBV4-91471, gammaEBV4-91480, gammaEBV4-91705, gammaEBV4-93050, gammaEBV4-93159, gammaEBV4-93490, gammaEBV4-94085, gammaEBV4-94177, gammaEBV4-94212, gammaEBV4-94589, gammaEBV4-95186, gammaEBV4-95199, gammaEBV4-95240, gammaEBV4-95258, gammaEBV4-95261, gammaEBV4-95588, gammaEBV4-95590, gammaEBV4-95666, gammaEBV4-95906, gammaEBV4-95942, gammaEBV4-95961, gammaEBV4-95963, gammaEBV4-95969, gammaEBV4-95974, gammaEBV4-95983, gammaEBV4-95984, gammaEBV4-95999, gammaEBV4-96000, gammaEBV4-96003, gammaEBV4-96004, gammaEBV4-96006, gammaEBV4-96008, gammaEBV4-96010, gammaEBV4-96024, gammaEBV4-96029, gammaEBV4-96038, gammaEBV4-96055, gammaEBV4-96064, gammaEBV4-96065, gammaEBV4-96070, gammaEBV4-96073, gammaEBV4-96078, gammaEBV4-96096, gammaEBV4-96114, gammaEBV4-96117, gammaEBV4-96118, gammaEBV4-96123, gammaEBV4-96127, gammaEBV4-96128, gammaEBV4-96142, gammaEBV4-96181, gammaEBV4-96193, gammaEBV4-96197, gammaEBV4-96199, gammaEBV4-96203, gammaEBV4-96205, gammaEBV4-96207, gammaEBV4-96208, gammaEBV4-96240, gammaEBV4-96254, gammaEBV4-96259, gammaEBV4-96266, gammaEBV4-96276, gammaEBV4-96279, gammaEBV4-96280, gammaEBV4-96281, gammaEBV4-96289, gammaEBV4-96296, gammaEBV4-96298, gammaEBV4-96302, gammaEBV4-96306, gammaEBV4-96310, gammaEBV4-96313, gammaEBV4-96314, gammaEBV4-96315, gammaEBV4-96316, gammaEBV4-96319, gammaEBV4-96328, gammaEBV4-96333, gammaEBV4-96334, gammaEBV4-96337, gammaEBV4-96346, gammaEBV4-96349, gammaEBV4-96357, gammaEBV4-96364, gammaEBV4-96365, gammaEBV4-96367, gammaEBV4-96370, gammaEBV4-96373, gammaEBV4-96378, gammaEBV4-96379, gammaEBV4-96380, gammaEBV4-96382, gammaEBV4-96387, gammaEBV4-96388, gammaEBV4-96394, gammaEBV4-96396, gammaEBV4-96399, gammaEBV4-96400, gammaEBV4-96403, gammaEBV4-96405, gammaEBV4-96406, gammaEBV4-96410, gammaEBV4-96414, gammaEBV4-96415, gammaEBV4-96416, gammaEBV4-96417, gammaEBV4-96419, gammaEBV4-96422, gammaEBV4-96424, gammaEBV4-96425, gammaEBV4-96430, gammaEBV4-96436, gammaEBV4-96440, gammaEBV4-96442, gammaEBV4-96445, gammaEBV4-96446, gammaEBV4-96459, gammaEBV4-96462, gammaEBV4-96464, gammaEBV4-96466, gammaEBV4-96467, gammaEBV4-96468, gammaEBV4-96470, gammaEBV4-96472, gammaEBV4-96473, gammaEBV4-96481, gammaEBV4-96505, gammaEBV4-96521, gammaEBV4-96522, gammaEBV4-96542, gammaEBV4-96546, gammaEBV4-96554, gammaEBV4-96565, gammaEBV4-96566, gammaEBV4-96569, gammaEBV4-96575, gammaEBV4-96582, gammaEBV4-96586, gammaEBV4-96587, gammaEBV4-96588, gammaEBV4-96591, gammaEBV4-96592, gammaEBV4-96594, gammaEBV4-96595, gammaEBV4-96596, gammaEBV4-96598, gammaEBV4-96599, gammaEBV4-96601, gammaEBV4-96604, gammaEBV4-96607, gammaEBV4-96618, gammaEBV4-96622, gammaEBV4-96633, gammaEBV4-96634, gammaEBV4-96636, gammaEBV4-96637, gammaEBV4-96638, gammaEBV4-96640, gammaEBV4-96653, gammaEBV4-96675, gammaEBV4-96677, gammaEBV4-96685, gammaEBV4-96701, gammaEBV4-96703, gammaEBV4-96714, gammaEBV4-96719, gammaEBV4-96722, gammaEBV4-96796, gammaEBV4-97303, gammaEBV4-97310, gammaEBV4-97457, gammaEBV4-97485, gammaEBV4-97584, gammaEBV4-97632, gammaEBV4-98321, gammaEBV4-98485, gammaEBV4-98607, gammaEBV4-98649, gammaEBV4-98857, gammaEBV4-99257, gammaEBV4-99261, gammaEBV4-99275, gammaEBV4-99399, gammaEBV4-99402, gammaEBV4-99405, gammaEBV4-99422, gammaEBV4-99423, gammaEBV4-99440, gammaEBV4-99441, gammaEBV4-99535

Row 29: gene = EBNA-3A

gene: EBNA-3A

sample: IM1, IM2, IM10, IM11, IM12, IM13, IM14, IM15, IM16, IM17, IM4, IM5, IM6, IM7, IM8, IM9

pos: gammaEBV4-10539, gammaEBV4-10920, gammaEBV4-11339, gammaEBV4-11955, gammaEBV4-13361, gammaEBV4-16098, gammaEBV4-19491, gammaEBV4-22574, gammaEBV4-26126, gammaEBV4-34536, gammaEBV4-35458, gammaEBV4-35769, gammaEBV4-36014, gammaEBV4-36209, gammaEBV4-36221, gammaEBV4-36265, gammaEBV4-36307, gammaEBV4-36315, gammaEBV4-36322, gammaEBV4-36355, gammaEBV4-36356, gammaEBV4-36360, gammaEBV4-36368, gammaEBV4-36389, gammaEBV4-36390, gammaEBV4-36391, gammaEBV4-36393, gammaEBV4-36394, gammaEBV4-36397, gammaEBV4-36399, gammaEBV4-36401, gammaEBV4-36402, gammaEBV4-36403, gammaEBV4-36405, gammaEBV4-36406, gammaEBV4-36408, gammaEBV4-36410, gammaEBV4-36411, gammaEBV4-36414, gammaEBV4-36415, gammaEBV4-36417, gammaEBV4-36420, gammaEBV4-36421, gammaEBV4-36423, gammaEBV4-36424, gammaEBV4-36425, gammaEBV4-36429, gammaEBV4-36432, gammaEBV4-36433, gammaEBV4-36435, gammaEBV4-36436, gammaEBV4-36437, gammaEBV4-36438, gammaEBV4-36439, gammaEBV4-36441, gammaEBV4-36442, gammaEBV4-36445, gammaEBV4-36446, gammaEBV4-36447, gammaEBV4-36448, gammaEBV4-36449, gammaEBV4-36450, gammaEBV4-36451, gammaEBV4-36453, gammaEBV4-36454, gammaEBV4-36455, gammaEBV4-36457, gammaEBV4-36458, gammaEBV4-36459, gammaEBV4-36460, gammaEBV4-36462, gammaEBV4-36463, gammaEBV4-36464, gammaEBV4-36465, gammaEBV4-36466, gammaEBV4-36468, gammaEBV4-36469, gammaEBV4-36470, gammaEBV4-36472, gammaEBV4-36475, gammaEBV4-36476, gammaEBV4-36477, gammaEBV4-36478, gammaEBV4-36479, gammaEBV4-36481, gammaEBV4-36482, gammaEBV4-36483, gammaEBV4-36486, gammaEBV4-36487, gammaEBV4-36489, gammaEBV4-36490, gammaEBV4-36491, gammaEBV4-36492, gammaEBV4-36495, gammaEBV4-36497, gammaEBV4-36498, gammaEBV4-36499, gammaEBV4-36501, gammaEBV4-36502, gammaEBV4-36507, gammaEBV4-36521, gammaEBV4-36528, gammaEBV4-36543, gammaEBV4-36558, gammaEBV4-36564, gammaEBV4-36710, gammaEBV4-36712, gammaEBV4-36745, gammaEBV4-36781, gammaEBV4-36801,

gammaEBV4-36816, gammaEBV4-36834, gammaEBV4-36855, gammaEBV4-36856, gammaEBV4-36860, gammaEBV4-36865, gammaEBV4-36869, gammaEBV4-36882, gammaEBV4-36885, gammaEBV4-36897, gammaEBV4-36924, gammaEBV4-36940, gammaEBV4-36942, gammaEBV4-36946, gammaEBV4-36947, gammaEBV4-36961, gammaEBV4-36965, gammaEBV4-36993, gammaEBV4-37009, gammaEBV4-37019, gammaEBV4-37033, gammaEBV4-37044, gammaEBV4-37087, gammaEBV4-37113, gammaEBV4-37116, gammaEBV4-37130, gammaEBV4-37270, gammaEBV4-37490, gammaEBV4-37678, gammaEBV4-37924, gammaEBV4-37979, gammaEBV4-38989, gammaEBV4-39482, gammaEBV4-39873, gammaEBV4-39899, gammaEBV4-39903, gammaEBV4-39908, gammaEBV4-39915, gammaEBV4-39925, gammaEBV4-40194, gammaEBV4-40213, gammaEBV4-40219, gammaEBV4-40220, gammaEBV4-40224, gammaEBV4-40225, gammaEBV4-40263, gammaEBV4-40282, gammaEBV4-41499, gammaEBV4-41553, gammaEBV4-41648, gammaEBV4-41794, gammaEBV4-41821, gammaEBV4-43009, gammaEBV4-43140, gammaEBV4-43141, gammaEBV4-43142, gammaEBV4-43145, gammaEBV4-43146, gammaEBV4-43373, gammaEBV4-43465, gammaEBV4-43477, gammaEBV4-43509, gammaEBV4-43624, gammaEBV4-43684, gammaEBV4-43981, gammaEBV4-44109, gammaEBV4-44155, gammaEBV4-44242, gammaEBV4-44658, gammaEBV4-45678, gammaEBV4-45790, gammaEBV4-45805, gammaEBV4-45860, gammaEBV4-46042, gammaEBV4-46062, gammaEBV4-46257, gammaEBV4-46472, gammaEBV4-46706, gammaEBV4-46912, gammaEBV4-47220, gammaEBV4-47236, gammaEBV4-47240, gammaEBV4-47908, gammaEBV4-47955, gammaEBV4-48227, gammaEBV4-48251, gammaEBV4-48335, gammaEBV4-48519, gammaEBV4-48797, gammaEBV4-48983, gammaEBV4-49102, gammaEBV4-49719, gammaEBV4-50133, gammaEBV4-50242, gammaEBV4-51166, gammaEBV4-51500, gammaEBV4-51571, gammaEBV4-52091, gammaEBV4-52205, gammaEBV4-52211, gammaEBV4-52227, gammaEBV4-52259, gammaEBV4-52372, gammaEBV4-52814, gammaEBV4-52837, gammaEBV4-52952, gammaEBV4-53724, gammaEBV4-53902, gammaEBV4-54149, gammaEBV4-54243, gammaEBV4-54255, gammaEBV4-54491, gammaEBV4-54802, gammaEBV4-54921, gammaEBV4-55000, gammaEBV4-55730, gammaEBV4-55780, gammaEBV4-55793, gammaEBV4-55811, gammaEBV4-55869, gammaEBV4-55887, gammaEBV4-55914, gammaEBV4-56237, gammaEBV4-56566, gammaEBV4-56866, gammaEBV4-57429, gammaEBV4-57632, gammaEBV4-57708, gammaEBV4-57740, gammaEBV4-57806, gammaEBV4-57842, gammaEBV4-57869, gammaEBV4-57870, gammaEBV4-57879, gammaEBV4-57880, gammaEBV4-57896, gammaEBV4-57897, gammaEBV4-58047, gammaEBV4-58301, gammaEBV4-58407, gammaEBV4-58634, gammaEBV4-58692, gammaEBV4-58785, gammaEBV4-58867, gammaEBV4-59115, gammaEBV4-59448, gammaEBV4-59672, gammaEBV4-59799, gammaEBV4-59923, gammaEBV4-59992, gammaEBV4-60095, gammaEBV4-60374, gammaEBV4-60398, gammaEBV4-61987, gammaEBV4-62009, gammaEBV4-62182, gammaEBV4-62247, gammaEBV4-62391, gammaEBV4-62449, gammaEBV4-62499, gammaEBV4-62677, gammaEBV4-62690, gammaEBV4-62774, gammaEBV4-62778, gammaEBV4-62916, gammaEBV4-63356, gammaEBV4-63580, gammaEBV4-63692, gammaEBV4-64008, gammaEBV4-64199, gammaEBV4-64224, gammaEBV4-64324, gammaEBV4-64571, gammaEBV4-64795, gammaEBV4-64976, gammaEBV4-65127, gammaEBV4-65205, gammaEBV4-65433, gammaEBV4-65450, gammaEBV4-65685, gammaEBV4-65718, gammaEBV4-65785, gammaEBV4-65950, gammaEBV4-66061, gammaEBV4-66069, gammaEBV4-66363, gammaEBV4-66405, gammaEBV4-66612, gammaEBV4-66809, gammaEBV4-67279, gammaEBV4-67485, gammaEBV4-67505, gammaEBV4-67749, gammaEBV4-68057, gammaEBV4-68261, gammaEBV4-68279, gammaEBV4-68497, gammaEBV4-68583, gammaEBV4-68589, gammaEBV4-68612, gammaEBV4-68646, gammaEBV4-68657, gammaEBV4-68729, gammaEBV4-68748, gammaEBV4-69148, gammaEBV4-69192, gammaEBV4-69276, gammaEBV4-69611, gammaEBV4-69904, gammaEBV4-69950, gammaEBV4-70330, gammaEBV4-70538, gammaEBV4-71139, gammaEBV4-71229, gammaEBV4-71292, gammaEBV4-71344, gammaEBV4-71546, gammaEBV4-71618, gammaEBV4-71671, gammaEBV4-72053, gammaEBV4-72289, gammaEBV4-72560, gammaEBV4-72580, gammaEBV4-72690, gammaEBV4-72723, gammaEBV4-72979, gammaEBV4-73029, gammaEBV4-73305, gammaEBV4-73366, gammaEBV4-73378, gammaEBV4-73577, gammaEBV4-73713, gammaEBV4-73903, gammaEBV4-73989, gammaEBV4-74183, gammaEBV4-74250, gammaEBV4-74856, gammaEBV4-74882, gammaEBV4-75292, gammaEBV4-75357, gammaEBV4-75429, gammaEBV4-75555, gammaEBV4-75694, gammaEBV4-75699, gammaEBV4-76014, gammaEBV4-76449, gammaEBV4-76489, gammaEBV4-76525, gammaEBV4-76598, gammaEBV4-76943, gammaEBV4-77179, gammaEBV4-77304, gammaEBV4-77373, gammaEBV4-77401, gammaEBV4-77404, gammaEBV4-77419, gammaEBV4-77613, gammaEBV4-77647, gammaEBV4-77672, gammaEBV4-77826, gammaEBV4-78055, gammaEBV4-78268, gammaEBV4-78767, gammaEBV4-78969, gammaEBV4-79898, gammaEBV4-80010, gammaEBV4-80158, gammaEBV4-80180, gammaEBV4-80333, gammaEBV4-80380, gammaEBV4-80419, gammaEBV4-80444, gammaEBV4-81096, gammaEBV4-81378, gammaEBV4-81601, gammaEBV4-81671, gammaEBV4-81722, gammaEBV4-81794, gammaEBV4-81818, gammaEBV4-81950, gammaEBV4-81952, gammaEBV4-81957, gammaEBV4-81959, gammaEBV4-82027, gammaEBV4-82041, gammaEBV4-82056, gammaEBV4-82082, gammaEBV4-82089, gammaEBV4-82233, gammaEBV4-82234, gammaEBV4-82250, gammaEBV4-82253, gammaEBV4-82254, gammaEBV4-82259, gammaEBV4-82274, gammaEBV4-82347, gammaEBV4-82356, gammaEBV4-82358, gammaEBV4-82363, gammaEBV4-82366, gammaEBV4-82414, gammaEBV4-82418, gammaEBV4-82557, gammaEBV4-82685, gammaEBV4-82902, gammaEBV4-82911, gammaEBV4-83123, gammaEBV4-83632, gammaEBV4-84936, gammaEBV4-9391, gammaEBV4-9426, gammaEBV4-9427, gammaEBV4-9428, gammaEBV4-9430, gammaEBV4-9432, gammaEBV4-9433, gammaEBV4-9434, gammaEBV4-9437, gammaEBV4-9438, gammaEBV4-9439, gammaEBV4-9442, gammaEBV4-9444, gammaEBV4-9446, gammaEBV4-9447, gammaEBV4-9518, gammaEBV4-9679, gammaEBV4-9712

Row 30: gene = EBNA-LP

gene: EBNA-LP

sample: IM1, IM2, IM10, IM11, IM12, IM13, IM14, IM15, IM16, IM17, IM4, IM5, IM6, IM7, IM8, IM9

pos: gammaEBV4-13361, gammaEBV4-16098, gammaEBV4-19491, gammaEBV4-22574, gammaEBV4-26126, gammaEBV4-34536, gammaEBV4-35458, gammaEBV4-35769, gammaEBV4-36014, gammaEBV4-36209, gammaEBV4-36221, gammaEBV4-36265, gammaEBV4-36307, gammaEBV4-36315, gammaEBV4-36322, gammaEBV4-36355, gammaEBV4-36356, gammaEBV4-36360, gammaEBV4-36368, gammaEBV4-36389, gammaEBV4-36390, gammaEBV4-36391, gammaEBV4-36393, gammaEBV4-36394, gammaEBV4-36397, gammaEBV4-36399, gammaEBV4-36401, gammaEBV4-36402, gammaEBV4-36403, gammaEBV4-36405, gammaEBV4-36406, gammaEBV4-36408, gammaEBV4-36410, gammaEBV4-36411, gammaEBV4-36414, gammaEBV4-36415, gammaEBV4-36417, gammaEBV4-36420, gammaEBV4-36421, gammaEBV4-36423, gammaEBV4-36424, gammaEBV4-36425, gammaEBV4-36429, gammaEBV4-36432, gammaEBV4-36433, gammaEBV4-36435, gammaEBV4-36436, gammaEBV4-36437, gammaEBV4-36438, gammaEBV4-36439, gammaEBV4-36441, gammaEBV4-36442, gammaEBV4-36445, gammaEBV4-36446, gammaEBV4-36447, gammaEBV4-36448, gammaEBV4-36449, gammaEBV4-36450, gammaEBV4-36451, gammaEBV4-36453, gammaEBV4-36454, gammaEBV4-36455, gammaEBV4-36457, gammaEBV4-36458, gammaEBV4-36459, gammaEBV4-36460, gammaEBV4-36462, gammaEBV4-36463, gammaEBV4-36464, gammaEBV4-36465, gammaEBV4-36466, gammaEBV4-36468, gammaEBV4-36469, gammaEBV4-36470, gammaEBV4-36472, gammaEBV4-36475, gammaEBV4-36476, gammaEBV4-36477, gammaEBV4-36478, gammaEBV4-36479, gammaEBV4-36481, gammaEBV4-36482, gammaEBV4-36483, gammaEBV4-36486, gammaEBV4-36487, gammaEBV4-36489, gammaEBV4-36490, gammaEBV4-36491, gammaEBV4-36492, gammaEBV4-36495, gammaEBV4-36497, gammaEBV4-36498, gammaEBV4-36499, gammaEBV4-36501, gammaEBV4-36502, gammaEBV4-36507, gammaEBV4-36521, gammaEBV4-36528, gammaEBV4-36543, gammaEBV4-36558, gammaEBV4-36564, gammaEBV4-36710, gammaEBV4-36712, gammaEBV4-36745, gammaEBV4-36781, gammaEBV4-36801, gammaEBV4-36816, gammaEBV4-36834, gammaEBV4-36855, gammaEBV4-36856, gammaEBV4-36860, gammaEBV4-36865, gammaEBV4-36869, gammaEBV4-36882, gammaEBV4-36885, gammaEBV4-36897, gammaEBV4-36924, gammaEBV4-36940, gammaEBV4-36942, gammaEBV4-36946, gammaEBV4-36947, gammaEBV4-36961, gammaEBV4-36965, gammaEBV4-36993, gammaEBV4-37009, gammaEBV4-37019, gammaEBV4-37033, gammaEBV4-37044, gammaEBV4-37087, gammaEBV4-37113, gammaEBV4-37116, gammaEBV4-37130, gammaEBV4-37270, gammaEBV4-37490, gammaEBV4-37678, gammaEBV4-37924, gammaEBV4-37979, gammaEBV4-38989, gammaEBV4-39482

Row 31: gene = LF2

gene: LF2

sample: IM16

pos: gammaEBV4-147843, gammaEBV4-147959, gammaEBV4-148630, gammaEBV4-148650, gammaEBV4-148658, gammaEBV4-148892, gammaEBV4-148962, gammaEBV4-148975, gammaEBV4-149020, gammaEBV4-149096, gammaEBV4-149397, gammaEBV4-149494, gammaEBV4-149760, gammaEBV4-150546, gammaEBV4-150551, gammaEBV4-150702, gammaEBV4-150749, gammaEBV4-151271, gammaEBV4-151272

Row 32: gene = LF3

gene: LF3

sample: IM12

pos: gammaEBV4-138618, gammaEBV4-138625, gammaEBV4-138872, gammaEBV4-138960, gammaEBV4-139071, gammaEBV4-139128, gammaEBV4-139154, gammaEBV4-139166, gammaEBV4-139196, gammaEBV4-139198, gammaEBV4-139202, gammaEBV4-139204, gammaEBV4-139207, gammaEBV4-139209, gammaEBV4-139214, gammaEBV4-139216, gammaEBV4-139438, gammaEBV4-139502, gammaEBV4-140335, gammaEBV4-140663, gammaEBV4-142143, gammaEBV4-142423, gammaEBV4-144869, gammaEBV4-145085

Row 33: gene = LMP-1

gene: LMP-1

sample: IM12, IM13, IM5

pos: gammaEBV4-164551, gammaEBV4-164586, gammaEBV4-164676, gammaEBV4-164692, gammaEBV4-164787, gammaEBV4-164982, gammaEBV4-165051, gammaEBV4-165155, gammaEBV4-165639, gammaEBV4-165807, gammaEBV4-166017, gammaEBV4-166116, gammaEBV4-166237, gammaEBV4-166370, gammaEBV4-166656, gammaEBV4-166695, gammaEBV4-166813, gammaEBV4-167362, gammaEBV4-167370, gammaEBV4-167399, gammaEBV4-167404, gammaEBV4-167437, gammaEBV4-167488, gammaEBV4-167506, gammaEBV4-168640, gammaEBV4-169257, gammaEBV4-169350, gammaEBV4-169430, gammaEBV4-169442, gammaEBV4-169460, gammaEBV4-169485, gammaEBV4-169565

Row 34: gene = LMP-2A

gene: LMP-2A

sample: IM10, IM12, IM13, IM5

pos: gammaEBV4-164100, gammaEBV4-164161, gammaEBV4-164382, gammaEBV4-164387, gammaEBV4-164551, gammaEBV4-164586, gammaEBV4-164676, gammaEBV4-164692, gammaEBV4-164787, gammaEBV4-164982, gammaEBV4-165051, gammaEBV4-165155, gammaEBV4-165639, gammaEBV4-165807, gammaEBV4-166017, gammaEBV4-166116, gammaEBV4-166237, gammaEBV4-166370, gammaEBV4-166656, gammaEBV4-166695, gammaEBV4-166813, gammaEBV4-167362, gammaEBV4-167370, gammaEBV4-167399, gammaEBV4-167404, gammaEBV4-167437, gammaEBV4-167488, gammaEBV4-167506, gammaEBV4-168640, gammaEBV4-169257, gammaEBV4-169350, gammaEBV4-169430, gammaEBV4-169442, gammaEBV4-169460, gammaEBV4-169485, gammaEBV4-169565, gammaEBV4-171084, gammaEBV4-171244, gammaEBV4-171498, gammaEBV4-171768, gammaEBV4-171773, gammaEBV4-171783

Row 35: gene = LMP-2B

gene: LMP-2B

sample: IM1, IM2, IM10, IM11, IM12, IM13, IM14, IM15, IM16, IM17, IM4, IM5, IM6, IM7, IM8, IM9

pos: gammaEBV4-100012, gammaEBV4-100015, gammaEBV4-100017, gammaEBV4-100032, gammaEBV4-100470, gammaEBV4-100929, gammaEBV4-101078, gammaEBV4-101090, gammaEBV4-101143, gammaEBV4-101244, gammaEBV4-101896, gammaEBV4-101996, gammaEBV4-102132, gammaEBV4-102279, gammaEBV4-102548, gammaEBV4-102833, gammaEBV4-102972, gammaEBV4-103167, gammaEBV4-103346, gammaEBV4-103649, gammaEBV4-103838, gammaEBV4-104080, gammaEBV4-104243, gammaEBV4-104986, gammaEBV4-104994, gammaEBV4-105325, gammaEBV4-10539, gammaEBV4-105501, gammaEBV4-105542, gammaEBV4-105598, gammaEBV4-106045, gammaEBV4-106231, gammaEBV4-106388, gammaEBV4-106430, gammaEBV4-107019, gammaEBV4-107473, gammaEBV4-107738, gammaEBV4-108135, gammaEBV4-108164, gammaEBV4-108165, gammaEBV4-108219, gammaEBV4-108226, gammaEBV4-108228, gammaEBV4-109037, gammaEBV4-109074, gammaEBV4-10920, gammaEBV4-110375, gammaEBV4-110393, gammaEBV4-110468, gammaEBV4-110538, gammaEBV4-110756, gammaEBV4-110937, gammaEBV4-111238, gammaEBV4-112229, gammaEBV4-112312, gammaEBV4-112741, gammaEBV4-113146, gammaEBV4-11339, gammaEBV4-113587, gammaEBV4-113631, gammaEBV4-113886, gammaEBV4-113897, gammaEBV4-114033, gammaEBV4-114134, gammaEBV4-114178, gammaEBV4-114596, gammaEBV4-114785, gammaEBV4-115055, gammaEBV4-115222, gammaEBV4-115343, gammaEBV4-115861, gammaEBV4-115891, gammaEBV4-115990, gammaEBV4-116738, gammaEBV4-117372, gammaEBV4-117462, gammaEBV4-117583, gammaEBV4-117880, gammaEBV4-117936, gammaEBV4-117941, gammaEBV4-118378, gammaEBV4-118380, gammaEBV4-118437, gammaEBV4-118443, gammaEBV4-118497, gammaEBV4-118554, gammaEBV4-118559, gammaEBV4-119450, gammaEBV4-11955, gammaEBV4-119719, gammaEBV4-119738, gammaEBV4-119764, gammaEBV4-119766, gammaEBV4-120040, gammaEBV4-120837, gammaEBV4-121112, gammaEBV4-121131, gammaEBV4-122449, gammaEBV4-122641, gammaEBV4-123006, gammaEBV4-123073, gammaEBV4-123123, gammaEBV4-123468, gammaEBV4-123550, gammaEBV4-123573, gammaEBV4-123594, gammaEBV4-123722, gammaEBV4-123726, gammaEBV4-123773, gammaEBV4-123786, gammaEBV4-123790, gammaEBV4-123855, gammaEBV4-124007, gammaEBV4-124331, gammaEBV4-124333, gammaEBV4-124335, gammaEBV4-124336, gammaEBV4-124337, gammaEBV4-124338, gammaEBV4-124339, gammaEBV4-124340, gammaEBV4-124342, gammaEBV4-124343, gammaEBV4-124344, gammaEBV4-124345, gammaEBV4-124346, gammaEBV4-124347, gammaEBV4-124348, gammaEBV4-124349, gammaEBV4-124350, gammaEBV4-124351, gammaEBV4-124352, gammaEBV4-124356, gammaEBV4-124357, gammaEBV4-124373, gammaEBV4-124681, gammaEBV4-124988, gammaEBV4-125072, gammaEBV4-125106, gammaEBV4-125211, gammaEBV4-125323, gammaEBV4-125330, gammaEBV4-125786, gammaEBV4-125876, gammaEBV4-125881, gammaEBV4-126135, gammaEBV4-126219, gammaEBV4-126402, gammaEBV4-127266, gammaEBV4-127347, gammaEBV4-127645, gammaEBV4-127663, gammaEBV4-127669, gammaEBV4-127827, gammaEBV4-128010, gammaEBV4-128770, gammaEBV4-128837, gammaEBV4-129213, gammaEBV4-129395, gammaEBV4-130293, gammaEBV4-130302, gammaEBV4-130543, gammaEBV4-130568, gammaEBV4-130569, gammaEBV4-130696, gammaEBV4-130723, gammaEBV4-130930, gammaEBV4-131300, gammaEBV4-131367, gammaEBV4-131675, gammaEBV4-132155, gammaEBV4-132194, gammaEBV4-132223, gammaEBV4-132224, gammaEBV4-132232, gammaEBV4-132437, gammaEBV4-133088, gammaEBV4-13361, gammaEBV4-133974, gammaEBV4-134311, gammaEBV4-134348, gammaEBV4-134460, gammaEBV4-134537, gammaEBV4-134765, gammaEBV4-134834, gammaEBV4-135676, gammaEBV4-135687, gammaEBV4-135692, gammaEBV4-135852, gammaEBV4-135865, gammaEBV4-136075, gammaEBV4-136560, gammaEBV4-136578, gammaEBV4-136653, gammaEBV4-136754, gammaEBV4-136896, gammaEBV4-136927, gammaEBV4-137017, gammaEBV4-137032, gammaEBV4-137120, gammaEBV4-137135, gammaEBV4-137142, gammaEBV4-137148, gammaEBV4-137151, gammaEBV4-137170, gammaEBV4-137227, gammaEBV4-137268, gammaEBV4-138016, gammaEBV4-138281, gammaEBV4-138618, gammaEBV4-138625, gammaEBV4-138872, gammaEBV4-138960, gammaEBV4-139071, gammaEBV4-139128, gammaEBV4-139154, gammaEBV4-139166, gammaEBV4-139196, gammaEBV4-139198, gammaEBV4-139202, gammaEBV4-139204, gammaEBV4-139207, gammaEBV4-139209, gammaEBV4-139214, gammaEBV4-139216, gammaEBV4-139438, gammaEBV4-139502, gammaEBV4-140335, gammaEBV4-140663, gammaEBV4-142143, gammaEBV4-142423, gammaEBV4-144869, gammaEBV4-145085, gammaEBV4-145352, gammaEBV4-145444, gammaEBV4-146172, gammaEBV4-146843, gammaEBV4-147843, gammaEBV4-147959, gammaEBV4-148630, gammaEBV4-148650, gammaEBV4-148658, gammaEBV4-148892, gammaEBV4-148962, gammaEBV4-148975, gammaEBV4-149020, gammaEBV4-149096, gammaEBV4-149397, gammaEBV4-149494, gammaEBV4-149760, gammaEBV4-150546, gammaEBV4-150551, gammaEBV4-150702, gammaEBV4-150749, gammaEBV4-151271, gammaEBV4-151272, gammaEBV4-152545, gammaEBV4-152557, gammaEBV4-152613, gammaEBV4-153004, gammaEBV4-153077, gammaEBV4-153117, gammaEBV4-153180, gammaEBV4-153617, gammaEBV4-153639, gammaEBV4-153725, gammaEBV4-153763, gammaEBV4-153860, gammaEBV4-153864, gammaEBV4-153952, gammaEBV4-154055, gammaEBV4-154299, gammaEBV4-154429, gammaEBV4-154453, gammaEBV4-154654, gammaEBV4-154720, gammaEBV4-154982, gammaEBV4-155166, gammaEBV4-155263, gammaEBV4-155584, gammaEBV4-155832, gammaEBV4-156147, gammaEBV4-156363, gammaEBV4-156375, gammaEBV4-156562, gammaEBV4-156606, gammaEBV4-157058, gammaEBV4-157364, gammaEBV4-157434, gammaEBV4-157853, gammaEBV4-159158, gammaEBV4-159376, gammaEBV4-160788, gammaEBV4-16098, gammaEBV4-161004, gammaEBV4-161324, gammaEBV4-161348, gammaEBV4-161445, gammaEBV4-161533, gammaEBV4-162029, gammaEBV4-162140, gammaEBV4-162204, gammaEBV4-162480, gammaEBV4-162703, gammaEBV4-162758, gammaEBV4-163569, gammaEBV4-163668, gammaEBV4-163804, gammaEBV4-163829, gammaEBV4-164100, gammaEBV4-164161, gammaEBV4-164382, gammaEBV4-164387, gammaEBV4-164551, gammaEBV4-164586, gammaEBV4-164676, gammaEBV4-164692, gammaEBV4-164787, gammaEBV4-164982, gammaEBV4-165051, gammaEBV4-165155, gammaEBV4-165639, gammaEBV4-165807, gammaEBV4-166017, gammaEBV4-166116, gammaEBV4-166237, gammaEBV4-166370, gammaEBV4-166656, gammaEBV4-166695, gammaEBV4-166813, gammaEBV4-167362, gammaEBV4-167370, gammaEBV4-167399, gammaEBV4-167404, gammaEBV4-167437, gammaEBV4-167488, gammaEBV4-167506, gammaEBV4-168640, gammaEBV4-169257, gammaEBV4-169350, gammaEBV4-169430, gammaEBV4-169442, gammaEBV4-169460, gammaEBV4-169485, gammaEBV4-169565, gammaEBV4-171084, gammaEBV4-171244, gammaEBV4-171498, gammaEBV4-171768, gammaEBV4-171773, gammaEBV4-171783, gammaEBV4-187, gammaEBV4-19491, gammaEBV4-22574, gammaEBV4-2403, gammaEBV4-26126, gammaEBV4-2768, gammaEBV4-2769, gammaEBV4-2958, gammaEBV4-336, gammaEBV4-3452, gammaEBV4-34536, gammaEBV4-35458, gammaEBV4-35769, gammaEBV4-36014, gammaEBV4-36209, gammaEBV4-36221, gammaEBV4-36265, gammaEBV4-36307, gammaEBV4-36315, gammaEBV4-36322, gammaEBV4-36355, gammaEBV4-36356, gammaEBV4-36360, gammaEBV4-36368, gammaEBV4-36389, gammaEBV4-36390, gammaEBV4-36391, gammaEBV4-36393, gammaEBV4-36394, gammaEBV4-36397, gammaEBV4-36399, gammaEBV4-3640, gammaEBV4-36401, gammaEBV4-36402, gammaEBV4-36403, gammaEBV4-36405, gammaEBV4-36406, gammaEBV4-36408, gammaEBV4-36410, gammaEBV4-36411, gammaEBV4-36414, gammaEBV4-36415, gammaEBV4-36417, gammaEBV4-36420, gammaEBV4-36421, gammaEBV4-36423, gammaEBV4-36424, gammaEBV4-36425, gammaEBV4-36429, gammaEBV4-36432, gammaEBV4-36433, gammaEBV4-36435, gammaEBV4-36436, gammaEBV4-36437, gammaEBV4-36438, gammaEBV4-36439, gammaEBV4-36441, gammaEBV4-36442, gammaEBV4-36445, gammaEBV4-36446, gammaEBV4-36447, gammaEBV4-36448, gammaEBV4-36449, gammaEBV4-36450, gammaEBV4-36451, gammaEBV4-36453, gammaEBV4-36454, gammaEBV4-36455, gammaEBV4-36457, gammaEBV4-36458, gammaEBV4-36459, gammaEBV4-36460, gammaEBV4-36462, gammaEBV4-36463, gammaEBV4-36464, gammaEBV4-36465, gammaEBV4-36466, gammaEBV4-36468, gammaEBV4-36469, gammaEBV4-36470, gammaEBV4-36472, gammaEBV4-36475, gammaEBV4-36476, gammaEBV4-36477, gammaEBV4-36478, gammaEBV4-36479, gammaEBV4-36481, gammaEBV4-36482, gammaEBV4-36483, gammaEBV4-36486, gammaEBV4-36487, gammaEBV4-36489, gammaEBV4-36490, gammaEBV4-36491, gammaEBV4-36492, gammaEBV4-36495, gammaEBV4-36497, gammaEBV4-36498, gammaEBV4-36499, gammaEBV4-36501, gammaEBV4-36502, gammaEBV4-36507, gammaEBV4-36521, gammaEBV4-36528, gammaEBV4-36543, gammaEBV4-36558, gammaEBV4-36564, gammaEBV4-36710, gammaEBV4-36712, gammaEBV4-36745, gammaEBV4-36781, gammaEBV4-36801, gammaEBV4-36816, gammaEBV4-36834, gammaEBV4-36855, gammaEBV4-36856, gammaEBV4-36860, gammaEBV4-36865, gammaEBV4-36869, gammaEBV4-36882, gammaEBV4-36885, gammaEBV4-36897, gammaEBV4-36924, gammaEBV4-36940, gammaEBV4-36942, gammaEBV4-36946, gammaEBV4-36947, gammaEBV4-36961, gammaEBV4-36965, gammaEBV4-36993, gammaEBV4-37009, gammaEBV4-37019, gammaEBV4-37033, gammaEBV4-37044, gammaEBV4-37087, gammaEBV4-37113, gammaEBV4-37116, gammaEBV4-37130, gammaEBV4-3720, gammaEBV4-37270, gammaEBV4-37490, gammaEBV4-3754, gammaEBV4-3758, gammaEBV4-37678, gammaEBV4-3775, gammaEBV4-37924, gammaEBV4-37979, gammaEBV4-38989, gammaEBV4-39482, gammaEBV4-39873, gammaEBV4-39899, gammaEBV4-39903, gammaEBV4-39908, gammaEBV4-39915, gammaEBV4-39925, gammaEBV4-40194, gammaEBV4-40213, gammaEBV4-40219, gammaEBV4-40220, gammaEBV4-40224, gammaEBV4-40225, gammaEBV4-40263, gammaEBV4-40282, gammaEBV4-4082, gammaEBV4-413, gammaEBV4-41499, gammaEBV4-41553, gammaEBV4-416, gammaEBV4-41648, gammaEBV4-41794, gammaEBV4-41821, gammaEBV4-43009, gammaEBV4-43140, gammaEBV4-43141, gammaEBV4-43142, gammaEBV4-43145, gammaEBV4-43146, gammaEBV4-43373, gammaEBV4-43465, gammaEBV4-43477, gammaEBV4-43509, gammaEBV4-43624, gammaEBV4-43684, gammaEBV4-43981, gammaEBV4-44109, gammaEBV4-44155, gammaEBV4-44242, gammaEBV4-44658, gammaEBV4-45678, gammaEBV4-45790, gammaEBV4-45805, gammaEBV4-45860,

gammaEBV4-46042, gammaEBV4-46062, gammaEBV4-46257, gammaEBV4-46472, gammaEBV4-46706, gammaEBV4-46912, gammaEBV4-47220, gammaEBV4-47236, gammaEBV4-47240, gammaEBV4-47908, gammaEBV4-47955, gammaEBV4-48227, gammaEBV4-48251, gammaEBV4-48335, gammaEBV4-48519, gammaEBV4-48797, gammaEBV4-48983, gammaEBV4-49102, gammaEBV4-4944, gammaEBV4-49719, gammaEBV4-50133, gammaEBV4-50242, gammaEBV4-5080, gammaEBV4-51166, gammaEBV4-51500, gammaEBV4-51571, gammaEBV4-5182, gammaEBV4-52091, gammaEBV4-52205, gammaEBV4-52211, gammaEBV4-52227, gammaEBV4-52259, gammaEBV4-52372, gammaEBV4-52814, gammaEBV4-52837, gammaEBV4-52952, gammaEBV4-53724, gammaEBV4-53902, gammaEBV4-5406, gammaEBV4-54149, gammaEBV4-54243, gammaEBV4-54255, gammaEBV4-54491, gammaEBV4-54802, gammaEBV4-54921, gammaEBV4-55000, gammaEBV4-55730, gammaEBV4-55780, gammaEBV4-55793, gammaEBV4-55811, gammaEBV4-55869, gammaEBV4-55887, gammaEBV4-55914, gammaEBV4-56237, gammaEBV4-56566, gammaEBV4-5665, gammaEBV4-56866, gammaEBV4-5739, gammaEBV4-57429, gammaEBV4-5751, gammaEBV4-5758, gammaEBV4-5762, gammaEBV4-57632, gammaEBV4-57708, gammaEBV4-57740, gammaEBV4-57806, gammaEBV4-57842, gammaEBV4-57869, gammaEBV4-57870, gammaEBV4-57879, gammaEBV4-57880, gammaEBV4-57896, gammaEBV4-57897, gammaEBV4-5793, gammaEBV4-5796, gammaEBV4-5798, gammaEBV4-5799, gammaEBV4-5802, gammaEBV4-5803, gammaEBV4-5804, gammaEBV4-58047, gammaEBV4-5805, gammaEBV4-5807, gammaEBV4-5808, gammaEBV4-5809, gammaEBV4-5810, gammaEBV4-58301, gammaEBV4-58407, gammaEBV4-58634, gammaEBV4-58692, gammaEBV4-58785, gammaEBV4-58867, gammaEBV4-59115, gammaEBV4-59448, gammaEBV4-59672, gammaEBV4-59799, gammaEBV4-59923, gammaEBV4-59992, gammaEBV4-60095, gammaEBV4-60374, gammaEBV4-60398, gammaEBV4-61987, gammaEBV4-62009, gammaEBV4-62182, gammaEBV4-62247, gammaEBV4-62391, gammaEBV4-62449, gammaEBV4-62499, gammaEBV4-62677, gammaEBV4-62690, gammaEBV4-62774, gammaEBV4-62778, gammaEBV4-62916, gammaEBV4-63356, gammaEBV4-63580, gammaEBV4-63692, gammaEBV4-64008, gammaEBV4-64199, gammaEBV4-64224, gammaEBV4-64324, gammaEBV4-64571, gammaEBV4-64795, gammaEBV4-64976, gammaEBV4-65127, gammaEBV4-65205, gammaEBV4-65433, gammaEBV4-65450, gammaEBV4-65685, gammaEBV4-65718, gammaEBV4-65785, gammaEBV4-65950, gammaEBV4-66061, gammaEBV4-66069, gammaEBV4-66363, gammaEBV4-66405, gammaEBV4-66612, gammaEBV4-66809, gammaEBV4-67279, gammaEBV4-67485, gammaEBV4-67505, gammaEBV4-67749, gammaEBV4-68057, gammaEBV4-68261, gammaEBV4-68279, gammaEBV4-68497, gammaEBV4-68583, gammaEBV4-68589, gammaEBV4-68612, gammaEBV4-68646, gammaEBV4-68657, gammaEBV4-68729, gammaEBV4-68748, gammaEBV4-69148, gammaEBV4-69192, gammaEBV4-69276, gammaEBV4-69611, gammaEBV4-69904, gammaEBV4-69950, gammaEBV4-7019, gammaEBV4-70330, gammaEBV4-7035, gammaEBV4-70538, gammaEBV4-71139, gammaEBV4-71229, gammaEBV4-71292, gammaEBV4-71344, gammaEBV4-71546, gammaEBV4-71618, gammaEBV4-71671, gammaEBV4-72053, gammaEBV4-7210, gammaEBV4-72289, gammaEBV4-7253, gammaEBV4-72560, gammaEBV4-72580, gammaEBV4-72690, gammaEBV4-72723, gammaEBV4-72979, gammaEBV4-73029, gammaEBV4-73305, gammaEBV4-73366, gammaEBV4-73378, gammaEBV4-73577, gammaEBV4-73713, gammaEBV4-73903, gammaEBV4-73989, gammaEBV4-74183, gammaEBV4-74250, gammaEBV4-74856, gammaEBV4-74882, gammaEBV4-75292, gammaEBV4-75357, gammaEBV4-75429, gammaEBV4-75555, gammaEBV4-75694, gammaEBV4-75699, gammaEBV4-76014, gammaEBV4-76449, gammaEBV4-76489, gammaEBV4-76525, gammaEBV4-76598, gammaEBV4-7691, gammaEBV4-76943, gammaEBV4-7705, gammaEBV4-77179, gammaEBV4-77304, gammaEBV4-77373, gammaEBV4-77401, gammaEBV4-77404, gammaEBV4-77419, gammaEBV4-77613, gammaEBV4-77647, gammaEBV4-77672, gammaEBV4-77826, gammaEBV4-78055, gammaEBV4-78268, gammaEBV4-78767, gammaEBV4-78969, gammaEBV4-79898, gammaEBV4-80010, gammaEBV4-80158, gammaEBV4-80180, gammaEBV4-80333, gammaEBV4-80380, gammaEBV4-80419, gammaEBV4-80444, gammaEBV4-8048, gammaEBV4-81096, gammaEBV4-81378, gammaEBV4-81601, gammaEBV4-81671, gammaEBV4-81722, gammaEBV4-81794, gammaEBV4-81818, gammaEBV4-81950, gammaEBV4-81952, gammaEBV4-81957, gammaEBV4-81959, gammaEBV4-82027, gammaEBV4-82041, gammaEBV4-82056, gammaEBV4-82082, gammaEBV4-82089, gammaEBV4-82233, gammaEBV4-82234, gammaEBV4-82250, gammaEBV4-82253, gammaEBV4-82254, gammaEBV4-82259, gammaEBV4-82274, gammaEBV4-82347, gammaEBV4-82356, gammaEBV4-82358, gammaEBV4-82363, gammaEBV4-82366, gammaEBV4-82414, gammaEBV4-82418, gammaEBV4-8244, gammaEBV4-82557, gammaEBV4-82685, gammaEBV4-82902, gammaEBV4-82911, gammaEBV4-83123, gammaEBV4-83632, gammaEBV4-84936, gammaEBV4-8500, gammaEBV4-85018, gammaEBV4-85048, gammaEBV4-85131, gammaEBV4-85235, gammaEBV4-8555, gammaEBV4-85666, gammaEBV4-87597, gammaEBV4-87818, gammaEBV4-87870, gammaEBV4-87967, gammaEBV4-88158, gammaEBV4-88365, gammaEBV4-88383, gammaEBV4-88385, gammaEBV4-88388, gammaEBV4-88390, gammaEBV4-88393, gammaEBV4-88397, gammaEBV4-88400, gammaEBV4-88401, gammaEBV4-88402, gammaEBV4-88405, gammaEBV4-88409, gammaEBV4-88410, gammaEBV4-88411, gammaEBV4-88412, gammaEBV4-88413, gammaEBV4-88414, gammaEBV4-88415, gammaEBV4-88416, gammaEBV4-88417, gammaEBV4-88419, gammaEBV4-88420, gammaEBV4-88421, gammaEBV4-88423, gammaEBV4-88424, gammaEBV4-88425, gammaEBV4-88426, gammaEBV4-88427, gammaEBV4-88429, gammaEBV4-88431, gammaEBV4-88432, gammaEBV4-88433, gammaEBV4-88435, gammaEBV4-88436, gammaEBV4-88437, gammaEBV4-88438, gammaEBV4-88439, gammaEBV4-88440, gammaEBV4-88442, gammaEBV4-88444, gammaEBV4-88445, gammaEBV4-88446, gammaEBV4-88447, gammaEBV4-88448, gammaEBV4-88449, gammaEBV4-88450, gammaEBV4-88451, gammaEBV4-88452, gammaEBV4-88453, gammaEBV4-88454, gammaEBV4-88455, gammaEBV4-88456, gammaEBV4-88457, gammaEBV4-88458, gammaEBV4-88459, gammaEBV4-88462, gammaEBV4-88463, gammaEBV4-88464, gammaEBV4-88465, gammaEBV4-88466, gammaEBV4-88467, gammaEBV4-88469, gammaEBV4-88470, gammaEBV4-88471, gammaEBV4-88472, gammaEBV4-88474, gammaEBV4-88476, gammaEBV4-88478, gammaEBV4-88483, gammaEBV4-88486, gammaEBV4-88495, gammaEBV4-88497, gammaEBV4-88498, gammaEBV4-88499, gammaEBV4-88500, gammaEBV4-88501, gammaEBV4-88502, gammaEBV4-88503, gammaEBV4-88504, gammaEBV4-88506, gammaEBV4-88507, gammaEBV4-88508, gammaEBV4-88511, gammaEBV4-88512, gammaEBV4-88514, gammaEBV4-88516, gammaEBV4-88527, gammaEBV4-88528, gammaEBV4-88529, gammaEBV4-88597, gammaEBV4-88834, gammaEBV4-8901, gammaEBV4-89231, gammaEBV4-89456, gammaEBV4-9007, gammaEBV4-90140, gammaEBV4-90282, gammaEBV4-90294, gammaEBV4-90298, gammaEBV4-90302, gammaEBV4-90304, gammaEBV4-90308, gammaEBV4-90311, gammaEBV4-90312, gammaEBV4-90313, gammaEBV4-90315, gammaEBV4-90317, gammaEBV4-90319, gammaEBV4-90320, gammaEBV4-90321, gammaEBV4-90322, gammaEBV4-90324, gammaEBV4-90332, gammaEBV4-90339, gammaEBV4-90340, gammaEBV4-90341, gammaEBV4-90342, gammaEBV4-90343, gammaEBV4-90347, gammaEBV4-90348, gammaEBV4-90349, gammaEBV4-90350, gammaEBV4-90351, gammaEBV4-90352, gammaEBV4-90353, gammaEBV4-90354, gammaEBV4-90355, gammaEBV4-90356, gammaEBV4-90357, gammaEBV4-90358, gammaEBV4-90359, gammaEBV4-90361, gammaEBV4-90370, gammaEBV4-90371, gammaEBV4-90372, gammaEBV4-90373, gammaEBV4-90376, gammaEBV4-90378, gammaEBV4-91150, gammaEBV4-91422, gammaEBV4-91471, gammaEBV4-91480, gammaEBV4-91705, gammaEBV4-93050, gammaEBV4-93159, gammaEBV4-93490, gammaEBV4-9391, gammaEBV4-94085, gammaEBV4-94177, gammaEBV4-94212, gammaEBV4-9426, gammaEBV4-9427, gammaEBV4-9428, gammaEBV4-9430, gammaEBV4-9432, gammaEBV4-9433, gammaEBV4-9434, gammaEBV4-9437, gammaEBV4-9438, gammaEBV4-9439, gammaEBV4-9442, gammaEBV4-9444, gammaEBV4-9446, gammaEBV4-9447, gammaEBV4-94589, gammaEBV4-9518, gammaEBV4-95186, gammaEBV4-95199, gammaEBV4-95240, gammaEBV4-95258, gammaEBV4-95261, gammaEBV4-95588, gammaEBV4-95590, gammaEBV4-95666, gammaEBV4-95906, gammaEBV4-95942, gammaEBV4-95961, gammaEBV4-95963, gammaEBV4-95969, gammaEBV4-95974, gammaEBV4-95983, gammaEBV4-95984, gammaEBV4-95999, gammaEBV4-96000, gammaEBV4-96003, gammaEBV4-96004, gammaEBV4-96006, gammaEBV4-96008, gammaEBV4-96010, gammaEBV4-96024, gammaEBV4-96029, gammaEBV4-96038, gammaEBV4-96055, gammaEBV4-96064, gammaEBV4-96065, gammaEBV4-96070, gammaEBV4-96073, gammaEBV4-96078, gammaEBV4-96096, gammaEBV4-96114, gammaEBV4-96117, gammaEBV4-96118, gammaEBV4-96123, gammaEBV4-96127, gammaEBV4-96128, gammaEBV4-96142, gammaEBV4-96181, gammaEBV4-96193, gammaEBV4-96197, gammaEBV4-96199, gammaEBV4-962, gammaEBV4-96203, gammaEBV4-96205, gammaEBV4-96207, gammaEBV4-96208, gammaEBV4-96240, gammaEBV4-96254, gammaEBV4-96259, gammaEBV4-96266, gammaEBV4-96276, gammaEBV4-96279, gammaEBV4-96280, gammaEBV4-96281, gammaEBV4-96289, gammaEBV4-96296, gammaEBV4-96298, gammaEBV4-96302, gammaEBV4-96306, gammaEBV4-96310, gammaEBV4-96313, gammaEBV4-96314, gammaEBV4-96315, gammaEBV4-96316, gammaEBV4-96319, gammaEBV4-96328, gammaEBV4-96333, gammaEBV4-96334, gammaEBV4-96337, gammaEBV4-96346, gammaEBV4-96349, gammaEBV4-96357, gammaEBV4-96364, gammaEBV4-96365, gammaEBV4-96367, gammaEBV4-96370, gammaEBV4-96373, gammaEBV4-96378, gammaEBV4-96379, gammaEBV4-96380, gammaEBV4-96382, gammaEBV4-96387, gammaEBV4-96388, gammaEBV4-96394, gammaEBV4-96396, gammaEBV4-96399, gammaEBV4-96400, gammaEBV4-96403, gammaEBV4-96405, gammaEBV4-96406, gammaEBV4-96410, gammaEBV4-96414, gammaEBV4-96415, gammaEBV4-96416, gammaEBV4-96417, gammaEBV4-96419, gammaEBV4-96422, gammaEBV4-96424, gammaEBV4-96425, gammaEBV4-96430, gammaEBV4-96436, gammaEBV4-96440, gammaEBV4-96442, gammaEBV4-96445, gammaEBV4-96446, gammaEBV4-96459, gammaEBV4-96462, gammaEBV4-96464, gammaEBV4-96466, gammaEBV4-96467, gammaEBV4-96468, gammaEBV4-96470, gammaEBV4-96472, gammaEBV4-96473, gammaEBV4-96481, gammaEBV4-96505, gammaEBV4-96521, gammaEBV4-96522, gammaEBV4-96542, gammaEBV4-96546, gammaEBV4-96554, gammaEBV4-96565, gammaEBV4-96566, gammaEBV4-96569, gammaEBV4-96575, gammaEBV4-96582, gammaEBV4-96586, gammaEBV4-96587, gammaEBV4-96588, gammaEBV4-96591, gammaEBV4-96592, gammaEBV4-96594, gammaEBV4-96595, gammaEBV4-96596, gammaEBV4-96598, gammaEBV4-96599, gammaEBV4-96601, gammaEBV4-96604, gammaEBV4-96607, gammaEBV4-96618, gammaEBV4-96622, gammaEBV4-96633, gammaEBV4-96634, gammaEBV4-96636, gammaEBV4-96637, gammaEBV4-96638, gammaEBV4-96640, gammaEBV4-96653, gammaEBV4-96675, gammaEBV4-96677, gammaEBV4-96685, gammaEBV4-96701, gammaEBV4-96703, gammaEBV4-96714, gammaEBV4-96719, gammaEBV4-96722, gammaEBV4-9679, gammaEBV4-96796, gammaEBV4-9712, gammaEBV4-97303, gammaEBV4-97310, gammaEBV4-97457, gammaEBV4-97485, gammaEBV4-97584, gammaEBV4-97632, gammaEBV4-98321, gammaEBV4-98485, gammaEBV4-98607, gammaEBV4-98649, gammaEBV4-98857, gammaEBV4-99257, gammaEBV4-99261, gammaEBV4-99275, gammaEBV4-99399, gammaEBV4-99402, gammaEBV4-99405, gammaEBV4-99422, gammaEBV4-99423, gammaEBV4-99440, gammaEBV4-99441, gammaEBV4-99535

Supplemental Table 5

Sheet: CAEBV

gene	band	chr
LINC00486	p22.3	chr2-33141316; ; chr2-33141317
SETD3	q32.2	chr14-99887286; chr14-99887286; chr14-99887288; chr14-99887288; chr14-99887285; chr14-99887285; chr14-99887315; chr14-99887315; chr14-99887283; chr14-99887283; chr14-99887283; chr14-99887283; chr14-99887285; chr14-99887285; chr14-99887288; chr14-99887288; chr14-99887317; chr14-99887317; chr14-99887283; chr14-99887283; chr14-99887281; chr14-99887281; chr14-99887285; chr14-99887285; chr14-99887286; chr14-99887286; chr14-99887315; chr14-99887315; chr14-99887287; chr14-99887287; chr14-99887285; chr14-99887285
SYPL1	q22.3	chr7-105741914; chr7-105741914; chr7-105741915; chr7-105741915; chr7-105741917; chr7-105741917; chr7-105741915; chr7-105741915; chr7-105741916; chr7-105741916; chr7-105741888; chr7-105741888; chr7-105741914; chr7-105741914; chr7-105741914; chr7-105741914; chr7-105741915; chr7-105741915; chr7-105741915; chr7-105741915; chr7-105741915; chr7-105741915; chr7-105741915; chr7-105741915; chr7-105741915; chr7-105741915; chr7-105741916; chr7-105741916; chr7-105741916; chr7-105741916; chr7-105741916; chr7-105741916; chr7-105741917; chr7-105741917; chr7-105741914; chr7-105741914; chr7-105741915; chr7-105741915; chr7-105741914; chr7-105741914; chr7-105741915; chr7-105741915; chr7-105741915; chr7-105741915; chr7-105741915; chr7-105741915; chr7-105741915; chr7-105741915; chr7-105741915; chr7-105741915; chr7-105741915; chr7-105741915; chr7-105741915; chr7-105741915; chr7-105741915; chr7-105741915; chr7-105741916; chr7-105741916; chr7-105741915; chr7-105741915; chr7-105741915; chr7-105741915; chr7-105741914; chr7-105741914; chr7-105741915; chr7-105741915; chr7-105741914; chr7-105741914; chr7-105741915; chr7-105741915
TMEM216	q12.2	chr11-61158723; chr11-61158723; chr11-61158720; chr11-61158720; chr11-61158724; chr11-61158724; chr11-61158721; chr11-61158721; chr11-61158723; chr11-61158723; chr11-61158720; chr11-61158720; chr11-61158723; chr11-61158723; chr11-61158723; chr11-61158723; chr11-61158723; chr11-61158723; chr11-61158728; chr11-61158728; chr11-61158757; chr11-61158757; chr11-61158720; chr11-61158720; chr11-61158723; chr11-61158723; chr11-61158720; chr11-61158720; chr11-61158726; chr11-61158726; chr11-61158721; chr11-61158721
ACSL3	q36.1	chr2-223731462; chr2-223731462; chr2-223731495; chr2-223731495; chr2-223731466; chr2-223731466; chr2-223731495; chr2-223731495; chr2-223731497; chr2-223731497; chr2-223731463; chr2-223731463; chr2-223731463; chr2-223731463; chr2-223731462; chr2-223731462; chr2-223731463; chr2-223731463; chr2-223731463; chr2-223731463
FER1L6-AS2	q24.13	chr8-125184243; chr8-125184243; chr8-125184245; chr8-125184245; chr8-125184243; chr8-125184243; chr8-125184243; chr8-125184243; chr8-125184275; chr8-125184275; chr8-125184244; chr8-125184244; chr8-125184246; chr8-125184246; chr8-125184245; chr8-125184245; chr8-125184243; chr8-125184243; chr8-125184244; chr8-125184244; chr8-125184246; chr8-125184246; chr8-125184243; chr8-125184243
NGB	q24.3	chr14-77736168; chr14-77736168; chr14-77736169; chr14-77736169; chr14-77736168; chr14-77736168; chr14-77736168; chr14-77736168; chr14-77736166; chr14-77736166; chr14-77736167; chr14-77736167
MAGEB10	p21.3	chrX-27827589; chrX-27827589; chrX-27827589; chrX-27827589; chrX-27827646; chrX-27827646; chrX-27827601; chrX-27827601; chrX-27827601; chrX-27827601; chrX-27827601; chrX-27827601
MEGF8	q13.2	chr19-42874992; chr19-42874992; chr19-42874993; chr19-42874993; chr19-42874994; chr19-42874994; chr19-42874994; chr19-42874994
PPP1R15B	q32.1	chr1-204381175; chr1-204381175; chr1-204381173; chr1-204381173; chr1-204381175; chr1-204381175; chr1-204381173; chr1-204381173
TJP1	q13.1	chr15-30241253; chr15-30241253; chr15-30241816; chr15-30241816; chr15-30242059; chr15-30242059; chr15-30242051; chr15-30242051
CAMTA1	p36.31	chr1-7395201; chr1-7395201; chr1-7395201; chr1-7395201; chr1-7195715; chr1-7195715
SKIDA1	p12.31	chr10-21811198; chr10-21811198; chr10-21811197; chr10-21811197; chr10-21811229; chr10-21811229
MARCH10	q23.2	chr17-60870860; chr17-60870860; chr17-60870360; chr17-60870360
AGBL1	q25.3	chr15-87348238; chr15-87348238; chr15-86967742; chr15-86967742
DIS3L2	q37.1	chr2-233112353; chr2-233112353; chr2-233097518; chr2-233097518
DLGAP1	p11.31	chr18-3898310; chr18-3898310; chr18-3832478; chr18-3832478
DOCK1	q26.2	chr10-129136147; chr10-129136147; chr10-128928014; chr10-128928014
DPP4	q24.2	chr2-162920027; chr2-162920027; chr2-162873671; chr2-162873671
GALNTL6	q34.1	chr4-173489339; chr4-173489339; chr4-173623283; chr4-173623283
GLB1L3	q25	chr11-134178321; chr11-134178321; chr11-134178108; chr11-134178108
GRID2	q22.1, q22.2	chr4-94243004; chr4-94243004; chr4-94630652; chr4-94630652
LOC101928509	q34.1	chr4-175142914; chr4-175142914; chr4-175021722; chr4-175021722
MPPED1	q13.2	chr22-43895736; chr22-43895736; chr22-43895736; chr22-43895736; chr22-43891954; chr22-43891954; chr22-43895535; chr22-43895535
NHS	p22.13	chrX-17755543; chrX-17755569
PI4KA	q11.21	chr22-21172977; chr22-21172977; chr22-21111416; chr22-21111416
PPP2R2C	p16.1	chr4-6513658; chr4-6513658; chr4-6513664; chr4-6513664
PRKCA	q24.2	chr17-64603840; chr17-64603840; chr17-64795041; chr17-64795041; chr17-64794958; chr17-64794958
PRLH	q37.3	chr2-238474029; chr2-238474029
RAE1	q13.31	chr20-55925919; chr20-55925919; chr20-55925906; chr20-55925906; chr20-55925905; chr20-55925905
RNF213	q25.3	chr17-78289571; chr17-78289571; chr17-78289582; chr17-78289582; chr17-78287622; chr17-78287622; chr17-78289571; chr17-78289571
RNF38	p13.2	chr9-36347116; chr9-36347116; chr9-36347116; chr9-36347116
RTEL1	q13.33	chr20-62325013; chr20-62325013; chr20-62324869; chr20-62324869; chr20-62324811; chr20-62324811; chr20-62324892; chr20-62324892; chr20-62324873; chr20-62324873
RTEL1-TNFRSF6B	q13.33	chr20-62325013; chr20-62325013; chr20-62324869; chr20-62324869; chr20-62324811; chr20-62324811; chr20-62324892; chr20-62324892; chr20-62324873; chr20-62324873

gene	band	chr
RTF1	q15.1	chr15-41718913; chr15-41718913; chr15-41718883; chr15-41718883
SCML1	p22.13	chrX-17755543; chrX-17755543; chrX-17755569; chrX-17755569; chrX-17755569; chrX-17755569
TSNARE1	q24.3	chr8-143447308; chr8-143447308; chr8-143371839; chr8-143371839
AADACL2-AS1	q25.1	chr3-151636699; chr3-151636699
ACAN	q26.1	chr15-89408603; chr15-89408603
ACOXL	q13	chr2-111524230; chr2-111524230
ADAMTS12	p13.3, p13.2	chr5-33787812; chr5-33787812
ADAMTS5	q21.3	chr21-28295639; chr21-28295639
ADARB1	q22.3	chr21-46584471; chr21-46584471
ADGRE3	p13.12	chr19-14769521; chr19-14769521
ADGRL3	q13.1	chr4-62884559; chr4-62884559
AFF1	q22.1, q21.3	chr4-87981993; chr4-87981993
AGBL4	p33	chr1-50146013; chr1-50146013
AGMO	p21.2	chr7-15567819; chr7-15567819
AHRR	p15.33	chr5-310065; chr5-310065
AIG1	q24.2	chr6-143451988; chr6-143451988
AKAP13	q25.3	chr15-86185779; chr15-86185779
AKNA	q32	chr9-117150681; chr9-117150681; chr9-117150681; chr9-117150681
ANKRD30B	p11.21	chr18-14785909; chr18-14785909
ANKRD30BL	q21.2	chr2-133003300; chr2-133003300
ANKRD30BP3	q11.21	chr10-45676013; chr10-45676013
ANKS1B	q23.1	chr12-100231692; chr12-100231692
ANXA6	q33.1	chr5-150486759; chr5-150486759
APBB2	p14	chr4-41218429; chr4-41218429
API5	p12	chr11-43344884; chr11-43344884
ARSG	q24.2	chr17-66256335; chr17-66256335
ASCC2	q12.2	chr22-30210072; chr22-30210072
ASPH	q12.3	chr8-62615487; chr8-62615487
ASTN2	q33.1	chr9-119611582; chr9-119611582
ATG16L2	q13.4	chr11-72527991; chr11-72527991; chr11-72527991; chr11-72527991; chr11-72527991; chr11-72527991; chr11-72527991; chr11-72527991
ATG9B	q36.1	chr7-150721143; chr7-150721143; chr7-150721143; chr7-150721143
ATP6V0A4	q34	chr7-138440817; chr7-138440817
ATXN1	p22.3	chr6-16439573; chr6-16439573
BAGE2	p11.1	chr21-11085453; chr21-11085453
BCAR1	q23.1	chr16-75270272; chr16-75270272
BCL11A	p16.1	chr2-60694271; chr2-60694271
BIRC6	p22.3	chr2-32676082; chr2-32676082
BMP8B	p34.2	chr1-40243351; chr1-40243351
BRAF	q34	chr7-140486675; chr7-140486675
C8orf37-AS1	q22.1	chr8-96382284; chr8-96382284
CACNA1C	p13.33	chr12-2775992; chr12-2775992
CACNA1C-AS2	p13.33	chr12-2775992
CADPS2	q31.32	chr7-122187874; chr7-122187874
CAPN9	q42.2	chr1-230894871; chr1-230894871
CCDC151	p13.2	chr19-11533386; chr19-11533386
CCDC42	p13.1	chr17-8639093; chr17-8639093
CCNJ	q24.1	chr10-97807659; chr10-97807659
CCNJL	q33.3	chr5-159740899; chr5-159740899; chr5-159740899; chr5-159740899

gene	band	chr
CDC14B	q22.32, q22.33	chr9-99344563; chr9-99344563
CELF3	q21.3	chr1-151689308; chr1-151689308
CENPB	p13	chr20-3764858; chr20-3764858
CEP63	q22.2	chr3-134252817; chr3-134252817
CERCAM	q34.11	chr9-131182093; chr9-131182093; chr9-131182093; chr9-131182093
CES5A	q12.2	chr16-55912443; chr16-55912443
CHCHD3	q33, q32.3	chr7-132488173; chr7-132488173
CHMP1B2P	q21.1	chrX-79556466; chrX-79556466
CHRM2	q33	chr7-136577861; chr7-136577861
CHST15	q26.13	chr10-125832151; chr10-125832151
CILP2	p13.11	chr19-19653177; chr19-19653177
CMSS1	q12.1	chr3-99691213; chr3-99691213
CNBD1	q21.3	chr8-88238530; chr8-88238530
CNOT4	q33	chr7-135189252; chr7-135189252
CNTNAP3P2	q12	chr9-65581793; chr9-65581793
CNTNAP4	q23.1	chr16-76488107; chr16-76488107
COBL	p12.1	chr7-51329227; chr7-51329227
COG5	q22.3	chr7-107036995; chr7-107036995
COQ8A	q42.13	chr1-227165799; chr1-227165799
COX1	--	chrM-5894; chrM-5894
COX2	--	chrM-5894; chrM-5894
COX5BP1	q21.22	chr4-82843461; chr4-82843461
COX6B1	q13.12	chr19-36148648; chr19-36148648
COX7A2L	p21	chr2-42571029; chr2-42571029
CPEB3	q23.32	chr10-94041011; chr10-94041011
CRB1	q31.3	chr1-197323985; chr1-197323985; chr1-197323985; chr1-197323985
CRYGB	q34	chr2-209009127; chr2-209009127
CSMD1	p23.2	chr8-3683062; chr8-3683062
CSMD3	q23.3	chr8-113623568; chr8-113623568
CTDSPL	p22.2	chr3-37903171; chr3-37903171
CTNNA2	p12	chr2-79889194; chr2-79889194
CTNNA3	q21.3	chr10-68706554; chr10-68706554
CTNND2	p15.2	chr5-11345809; chr5-11345809
CUX2	q24.12, q24.11	chr12-111499143; chr12-111499143
CYLD	q12.1	chr16-50810040; chr16-50810040
CYTB	--	chrM-16246
DAB1	p32.2	chr1-57637208; chr1-57637208
DBET	q35.2	chr4-190988822; chr4-190988822
DDX11L5	p24.3	chr9-11165; chr9-11165
DDX46	q31.1	chr5-134132862; chr5-134132862
DEFB105A	p23.1	chr8-7680925; chr8-7680925; chr8-7680925
DEFB106A	p23.1	chr8-7680925; chr8-7680925
DGKA	q13.2	chr12-56323329; chr12-56323329
DGKG	q27.3, q27.2	chr3-186072993; chr3-186072993
DIAPH2	q21.33	chrX-96315249; chrX-96315249
DLGAP1-AS3	p11.31	chr18-3898310
DNAH11	p15.3	chr7-21819643; chr7-21819643
DNAH2	p13.1	chr17-7680734; chr17-7680734

gene	band	chr
DNAL1	q24.3	chr14-74171188
DNMT3A	p23.3	chr2-25508191; chr2-25508191
DNMT3B	q11.21	chr20-31372438; chr20-31372438
DOCK4	q31.1	chr7-111695380; chr7-111695380
DOCK8	p24.3	chr9-399990; chr9-399990
DPH1	p13.3	chr17-1946163; chr17-1946163
DPYS	q22.3	chr8-105474073; chr8-105474073
DSE	q22.1	chr6-116602263; chr6-116602263
DUS3L	p13.3	chr19-5787193; chr19-5787193
DUSP15	q11.21	chr20-30437460; chr20-30437460
DUX4L10	q26.3	chr10-135479389; chr10-135479389
DUX4L8	q35.2	chr4-190988822; chr4-190988822
EBF1	q33.3	chr5-158267698; chr5-158267698
EIF4BP4	p22.1	chr3-42381948; chr3-42381948
ENOX1	q14.11	chr13-43867451; chr13-43867451
ENTPD1-AS1	q24.1	chr10-97807659; chr10-97807659
EPB41L4A	q22.1, q22.2	chr5-111512323; chr5-111512323
EPHA6	q11.2	chr3-97371782; chr3-97371782
EPS15L1	p13.11	chr19-16512286; chr19-16512286; chr19-16512286
ERC2	p14.3	chr3-55548986; chr3-55548986
ERHP2	p12.1	chr6-53881231; chr6-53881231; chr6-53881231
ESPNL	q37.3	chr2-239035345; chr2-239035345; chr2-239035345; chr2-239035345
ETS1	q24.3	chr11-128352786; chr11-128352786
EXOC4	q33	chr7-133036945; chr7-133036945
EYA4	q23.2	chr6-133839843; chr6-133839843
FAM226A	q13.2	chrX-72163615; chrX-72163615
FAM227B	q21.2	chr15-49703205; chr15-49703205
FAM83B	p12.1	chr6-54777259; chr6-54777259
FBLN2	p25.1	chr3-13640677; chr3-13640677
FBRSL1	q24.33	chr12-133153704; chr12-133153704
FBXL7	p15.1	chr5-15621561; chr5-15621561
FBXO28	q42.11	chr1-224302946; chr1-224302946
FGD6	q22	chr12-95484125; chr12-95484125
FILIP1L	q12.1	chr3-99691213; chr3-99691213
FLOT1	p21.33	chr6-30704874; chr6-30704874
FOXN3	q32.11, q31.3	chr14-89829007; chr14-89829007; chr14-89828143; chr14-89828143; chr14-89828205; chr14-89828205
FRAS1	q21.21	chr4-79323875; chr4-79323875
FRMD5	q15.3	chr15-44231217; chr15-44231217
FUT9	q16.1	chr6-96571690; chr6-96571690
FZD8	p11.21	chr10-35928241; chr10-35928241
GADL1	p23, p24.1	chr3-30915954; chr3-30915954
GFRAL	p12.1	chr6-55238736; chr6-55238736
GLIS1	p32.3	chr1-54109871; chr1-54109871
GLTSCR2	q13.33	chr19-48253936; chr19-48253936
GOLGA2P10	q25.2	chr15-83142205; chr15-83142205
GOLGA4	p22.2	chr3-37399510; chr3-37399510
GPHN	q23.3	chr14-67590647; chr14-67590647
GRM7	p26.1	chr3-7774394; chr3-7774394

gene	band	chr
GSAP	q11.23	chr7-76983274; chr7-76983274
HECW1	p14.1, p13	chr7-43157031; chr7-43157031
HECW1-IT1	p14.1	chr7-43157031; chr7-43157031
HERPUD1	q13	chr16-56979760
HIP1R	q24.31	chr12-123348654
HIVEP2	q24.2	chr6-143235164; chr6-143235164
HPCAL1	p25.1	chr2-10539643; chr2-10539643
IQCJ-SCHIP1	q25.32, q25.33	chr3-159332927; chr3-159332927
ITGA9-AS1	p22.2	chr3-37903171; chr3-37903171; chr3-37903171
JPH2	q13.12	chr20-42805745; chr20-42805745; chr20-42805745
KALRN	q21.2	chr3-124381663; chr3-124381663
KANK3	p13.2	chr19-8393620; chr19-8393620
KCNH8	p24.3	chr3-19308976; chr3-19308976
KCNIP4	p15.31	chr4-21323370; chr4-21323370
KCNQ1	p15.4, p15.5	chr11-2524101; chr11-2524101
KCNS3	p24.2	chr2-18077140; chr2-18077140
KDM6A	p11.3	chrX-44865215; chrX-44865215
KIAA0922	q31.3	chr4-154423895; chr4-154423895
KIAA1147	q34	chr7-141364338; chr7-141364338
KIAA1549	q34	chr7-138592423; chr7-138592423
KIAA1644	q13.31	chr22-44677281; chr22-44677281
KIF1C	p13.2	chr17-4916659; chr17-4916659
KLF15	q21.3	chr3-126062025; chr3-126062025; chr3-126062026; chr3-126062026
KLHL29	p24.1	chr2-23771985; chr2-23771985
LACE1	q21	chr6-108810890; chr6-108810890
LIMCH1	p13	chr4-41435515; chr4-41435515
LINC00578	q26.32	chr3-177341605; chr3-177341605
LINC00598	q14.11	chr13-40959699; chr13-40959699; chr13-40959699; chr13-40959699
LINC00854	q21.31	chr17-41380228; chr17-41380228
LINC01214	q25.1	chr3-149994447; chr3-149994447
LINC01317	p22.3	chr2-34366749; chr2-34366749
LINC01478	q12.3	chr18-41958094; chr18-41958094
LINC01498	q23.3	chr12-108868954; chr12-108868954
LINC01716	q13.31	chr20-55171640
LINC01847	q33.3	chr5-159207996; chr5-159207996
LINC02085	q12.3	chr3-101661937; chr3-101661937; chr3-101661937; chr3-101661937
LINC02141	q21	chr16-59991509; chr16-59991509
LINGO1	q24.3	chr15-77910949; chr15-77910949
LITAF	p13.13	chr16-11650918; chr16-11650918
LMCD1-AS1	p26.1	chr3-8321796; chr3-8321796
LOC100420587	q12	chr19-29089558; chr19-29089558
LOC100507443	q33.3, q34	chr2-209009127; chr2-209009127
LOC101927286	q26.2	chr15-98061043; chr15-98061043
LOC101927829	p24.3	chr3-20383588; chr3-20383588
LOC101928314	q34.1	chr4-173623283; chr4-173623283
LOC101928961	q21.3	chr10-68706554; chr10-68706554
LOC102723344	q22.2, q22.31	chr15-63731059
LOC105377448	q31.1	chr4-139782212; chr4-139782212

gene	band	chr
LOC153910	q24.2	chr6-142943638; chr6-142943638
LOC339862	p24.3	chr3-18230653; chr3-18230653
LOC653786	p12.2	chr16-22562700; chr16-22562700
LOC728688	p12.2	chr12-20705459; chr12-20705459
LRFN2	p21.1, p21.2	chr6-40456144; chr6-40456144
LRRC20	q22.1	chr10-72137288; chr10-72137288; chr10-72137288; chr10-72137288
LRRTM3	q21.3	chr10-68706554; chr10-68706554
MAPKAP1	q33.3	chr9-128350697; chr9-128350697
MARCKS	q21	chr6-114178680; chr6-114178680; chr6-114178680
MDGA2	q21.3	chr14-47349268; chr14-47349268
MECOM	q26.2	chr3-169253326; chr3-169253326
MED6	q24.2	chr14-71067475; chr14-71067475
MICU2	q12.11	chr13-22084747; chr13-22084747
MIR376A2	q32.31	chr14-101504876; chr14-101504876; chr14-101504882; chr14-101504882
MIR376B	q32.31	chr14-101504876; chr14-101504876; chr14-101504882; chr14-101504882
MIR376C	q32.31	chr14-101504876; chr14-101504876; chr14-101504882; chr14-101504882
MIR4530	q13.2	chr19-39901685; chr19-39901685
MIR4683	p11.21	chr10-35928241
MIR6513	q35	chr2-219145874; chr2-219145874
MIR654	q32.31	chr14-101504876; chr14-101504876; chr14-101504882; chr14-101504882
MIR6723	p36.33	chr1-566443
MLC1	q13.33	chr22-50496744
MLH1	p22.2	chr3-37066023; chr3-37066023
MMP17	q24.33	chr12-132311899; chr12-132311899
MPP6	p15.3	chr7-24688592; chr7-24688592
MYH6	q11.2	chr14-23879459; chr14-23879459
MYLK4	p25.2	chr6-2694730; chr6-2694730
NAAA	q21.1	chr4-76831313
NALCN	q33.1	chr13-101709007; chr13-101709007
NALCN-AS1	q33.1, q32.3	chr13-101709007; chr13-101709007
NBDY	p11.21	chrX-56803314; chrX-56803314
NBPF11	q21.2	chr1-147577209; chr1-147577209
NCOA7	q22.32	chr6-126132867; chr6-126132867
NCOA7-AS1	q22.32	chr6-126132867; chr6-126132867
ND1	--	chrM-5894
ND2	--	chrM-5894
ND3	--	chrM-11998
ND4	--	chrM-11998; chrM-11998
ND4L	--	chrM-11998
ND5	--	chrM-11998; chrM-11998
ND6	--	chrM-16246; chrM-16246
NEGR1	p31.1	chr1-72644686; chr1-72644686
NEK1	q33	chr4-170395128; chr4-170395128
NELL1	p15.1	chr11-20964052; chr11-20964052
NFATC4	q12	chr14-24837629; chr14-24837629; chr14-24837629; chr14-24837629
NKAIN1	p35.2	chr1-31671727; chr1-31671727
NKAIN2	q22.31	chr6-125113992; chr6-125113992
NMT2	p13	chr10-15206897; chr10-15206897

gene	band	chr
NPSR1	p14.3	chr7-34734953; chr7-34734953
NPSR1-AS1	p14.3	chr7-34734953; chr7-34734953
NT5DC1	q22.1	chr6-116566650; chr6-116566650
NTSR1	q13.33	chr20-61371565; chr20-61371565
NUBPL	q12	chr14-32109651; chr14-32109651
OPA1	q29	chr3-193344082; chr3-193344082
OPA1-AS1	q29	chr3-193344082; chr3-193344082
OPCML	q25	chr11-132983754; chr11-132983754
OPN5	p12.3	chr6-47791626; chr6-47791626
OSBPL3	p15.3	chr7-24835674
OTOF	p23.3	chr2-26752147; chr2-26752147
OTUD7A	q13.3	chr15-31822087; chr15-31822087
OVCA2	p13.3	chr17-1946163; chr17-1946163
PACS2	q32.33	chr14-105790958; chr14-105790958
PAPPA2	q25.2	chr1-176718809; chr1-176718809
PARP15	q21.1	chr3-122305957; chr3-122305957
PASD1	q28	chrX-150731330; chrX-150731330
PCDH15	q21.1	chr10-56471858; chr10-56471858
PCNX1	q24.2	chr14-71376752; chr14-71376752
PCOLCE2	q23	chr3-142593741; chr3-142593741
PDCD6	p15.33	chr5-310065; chr5-310065
PDE10A	q27	chr6-166043238; chr6-166043238
PDE3A	p12.2	chr12-20705459; chr12-20705459
PDE4D	q11.2	chr5-59132066; chr5-59132066
PFKFB3	p15.1	chr10-6235717; chr10-6235717
PIK3C2A	p15.1	chr11-17133427; chr11-17133427
PIK3C2G	p12.3	chr12-18652805; chr12-18652805
PKDCC	p21	chr2-42275121; chr2-42275121
PKHD1	p12.3, p12.2	chr6-51917123; chr6-51917123
PLCH2	p36.32	chr1-2402828; chr1-2402828
PNKD	q35	chr2-219145874; chr2-219145874
POLD1	q13.33	chr19-50909801; chr19-50909801
PPARG	p25.2	chr3-12398898; chr3-12398898
PRR20C	q21.1	chr13-57726996; chr13-57726996
PYM1	q13.2	chr12-56323329; chr12-56323329
RAB30	q14.1	chr11-82745085; chr11-82745085
RAB6A	q13.4	chr11-73399667; chr11-73399667
RABL2B	q13.33	chr22-51204555
RERG	p12.3	chr12-15319480; chr12-15319480; chr12-15319479; chr12-15319479
RGS20	q11.23	chr8-54772653; chr8-54772653
RHOJ	q23.2	chr14-63760408
RIF1	q23.3	chr2-152272175; chr2-152272175
RINT1	q22.3	chr7-105199390; chr7-105199390
RNF20	q31.1	chr9-104301923; chr9-104301923
RNF24	p13	chr20-3930817; chr20-3930817
RPL23AP82	q13.33	chr22-51204555; chr22-51204555
RPL7L1P2	p12.1	chr7-51329227; chr7-51329227
RPS2P9	q32.1	chr1-198918906; chr1-198918906

gene	band	chr
RPS3AP37	q21.3	chr10-70432248; chr10-70432248
SCAMP1	q14.1	chr5-77765717; chr5-77765717
SCHIP1	q25.32, q25.33	chr3-159332927; chr3-159332927
SCN7A	q24.3	chr2-167320199; chr2-167320199
SEMA5A	p15.31	chr5-9197422; chr5-9197422
SH3BGRL3	p36.11	chr1-26609365
SHROOM3	q21.1	chr4-77491529; chr4-77491529
SIRPA	p13	chr20-1918623; chr20-1918623
SLC26A7	q21.3	chr8-92270629; chr8-92270629
SLC30A9	p13	chr4-42012516; chr4-42012516; chr4-42012516; chr4-42012516
SLC45A2	p13.2	chr5-33944137
SLC4A10	q24.2	chr2-162772656; chr2-162772656
SLC6A4	q11.2	chr17-28555400; chr17-28555400; chr17-28555478; chr17-28555478
SLC7A10	q13.11	chr19-33706571; chr19-33706571
SMAD4	q21.2	chr18-48565852; chr18-48565852
SMG6	p13.3	chr17-2098215; chr17-2098215
SMPD4P2	q12.11	chr13-19733784; chr13-19733784
SORCS2	p16.1	chr4-7240669; chr4-7240669
SOS2	q21.3	chr14-50605129; chr14-50605129
SOX6	p15.2, p15.1	chr11-16135288; chr11-16135288
SRC	q11.23	chr20-35996922; chr20-35996922
SRPX	p11.4	chrX-38010688; chrX-38010688
SRRT	q22.1	chr7-100472364; chr7-100472364
SSBP4	p13.11	chr19-18540470; chr19-18540470
ST3GAL2	q22.1	chr16-70453296; chr16-70453296
STX7	q23.2	chr6-132834000; chr6-132834000; chr6-132834000; chr6-132834000
SVEP1	q31.3	chr9-113303433; chr9-113303433
TACC2	q26.13	chr10-123817084; chr10-123817084
TAF1B	p25.1	chr2-10062677; chr2-10062677
TARID	q23.2	chr6-133839843; chr6-133839843
TAS1R2	p36.13	chr1-19184602; chr1-19184602
TBCD	q25.3	chr17-80832063; chr17-80832063
TCTEX1D1	p31.3	chr1-67241521; chr1-67241521
TEAD1	p15.2, p15.3	chr11-12874838; chr11-12874838
TENM2	q34	chr5-167578905; chr5-167578905
TERF2	q22.1	chr16-69399505; chr16-69399505
TESC	q24.22	chr12-117509333; chr12-117509333
TET1	q21.3	chr10-70432248; chr10-70432248
TGOLN2	p11.2	chr2-85554907; chr2-85554907
THRA	q21.1	chr17-38244658; chr17-38244658
TMBIM1	q35	chr2-219145874; chr2-219145874
TMED3	q25.1	chr15-79609310; chr15-79609310
TMEM132C	q24.32	chr12-128940912; chr12-128940912
TMEM40	p25.2	chr3-12810416; chr3-12810416
TOX2	q13.12	chr20-42676497; chr20-42676497
TOX3	q12.1	chr16-52581215; chr16-52581215; chr16-52581215; chr16-52581215
TP73-AS1	p36.32	chr1-3663191; chr1-3663191; chr1-3663191
TPRXL	p25.1	chr3-13988694; chr3-13988694

gene	band	chr
TPTE2P3	q14.3	chr13-53097754; chr13-53097754
TRAK1	p22.1	chr3-42212205; chr3-42212205
TRAK2	q33.1	chr2-202293466; chr2-202293466
TRDN	q22.31	chr6-123859628; chr6-123859628
TRIM2	q31.3	chr4-154165435; chr4-154165435
TRIP6	q22.1	chr7-100472364
TRNA	--	chrM-5894; chrM-5894
TRNAI18	q13.2	chr19-39901685
TRNAI22	p22.2	chr6-26744215
TRNC	--	chrM-5894; chrM-5894
TRND	--	chrM-5894; chrM-5894
TRNE	--	chrM-16246; chrM-16246
TRNG	--	chrM-11998
TRNH	--	chrM-11998; chrM-11998
TRNI	--	chrM-5894
TRNL2	--	chrM-11998; chrM-11998
TRNM	--	chrM-5894
TRNN	--	chrM-5894; chrM-5894
TRNP	--	chrM-16246; chrM-16246
TRNQ	--	chrM-5894; chrM-5894
TRNR	--	chrM-11998
TRNS1	--	chrM-5894
TRNS2	--	chrM-11998; chrM-11998
TRNT	--	chrM-16246; chrM-16246
TRNW	--	chrM-5894; chrM-5894
TRNY	--	chrM-5894; chrM-5894
TSPYL1	q22.1	chr6-116602263; chr6-116602263
TXNDC12	p32.3	chr1-52483960
UBASH3A	q22.3	chr21-43835071; chr21-43835071
UBR4	p36.13	chr1-19479019; chr1-19479019; chr1-19479024; chr1-19479024
UBXN11	p36.11	chr1-26609365; chr1-26609365
UNC13A	p13.11	chr19-17799854; chr19-17799854
UNC5D	p12	chr8-35215629; chr8-35215629
UNC93B1	q13.2	chr11-67771332; chr11-67771332; chr11-67771332
UNQ6494	q22.2	chr9-92296299; chr9-92296299
USP40	q37.1	chr2-234421284; chr2-234421284
UTRN	q24.2	chr6-144941821; chr6-144941821
VAMP8	p11.2	chr2-85806719; chr2-85806719
VPS37B	q24.31	chr12-123348654
VPS8	q27.2	chr3-184751065; chr3-184751065
WDFY4	q11.22, q11.23	chr10-50167648; chr10-50167648
WDR72	q21.3	chr15-54028541; chr15-54028541
XKR6	p23.1	chr8-10886783; chr8-10886783
ZFHX3	q22.2, q22.3	chr16-72820963; chr16-72820963
ZFP36	q13.2	chr19-39901685
ZFYVE9	p32.3	chr1-52713344; chr1-52713344
ZMIZ1	q22.3	chr10-80883324; chr10-80883324
ZNF423	q12.1	chr16-49796215; chr16-49796215

gene	band	chr
ZNF692	q44	chr1-249150127; chr1-249150127
ZNF815P	p22.1	chr7-5881326; chr7-5881326
AAK1	p13.3	chr2-69832275; chr2-69832275

Sheet: IM

gene	band	chr
LINC00486	p22.3	chr2-33141316; chr2-33141317
ACSL3	q36.1	chr2-223731462; chr2-223731462; chr2-223731495; chr2-223731495; chr2-223731466; chr2-223731466; chr2-223731495; chr2-223731495; chr2-223731497; chr2-223731497; chr2-223731463; chr2-223731463; chr2-223731463; chr2-223731463; chr2-223731462; chr2-223731462; chr2-223731463; chr2-223731463; chr2-223731463; chr2-223731463
ACSS1	p11.21	chr20-24997155; chr20-24997155
AFG3L1P	q24.3	chr16-90053778; chr16-90053778; chr16-90053789; chr16-90053789
ANAPC5	q24.31	chr12-121790526; chr12-121790526; chr12-121790526; chr12-121790526
CD99P1	p22.33	chrY-2492245; chrY-2492245
CDH11	q21	chr16-65122305; chr16-65122305
CDYL	p25.1	chr6-4780631; chr6-4780631
CNKSR2	p22.12	chrX-21594150; chrX-21594150
DAP	p15.2	chr5-10702132; chr5-10702132
DPP4	q24.2	chr2-162920027; chr2-162920027; chr2-162873671; chr2-162873671
ESR1	q25.1	chr6-152243365; chr6-152243365
FCMR	q32.1	chr1-207080433; chr1-207080433
FOXN3	q32.11, q31.3	chr14-89829007; chr14-89829007; chr14-89828143; chr14-89828143; chr14-89828205; chr14-89828205
GLB1L3	q25	chr11-134178321; chr11-134178321; chr11-134178108; chr11-134178108
GRID2	q22.1, q22.2	chr4-94243004; chr4-94243004; chr4-94630652; chr4-94630652
HERPUD2	p14.2	chr7-35726419; chr7-35726419
HSPG2	p36.12	chr1-22248784; chr1-22248784
HUWE1	p11.22	chrX-53630372; chrX-53630372
INTS11	p36.33	chr1-1251227; chr1-1251227
KLF12	q22.1	chr13-74642804; chr13-74642804
LOC101928663	p22.2	chr6-25256905; chr6-25256905
LOC643201	q35.2	chr5-175625603; chr5-175625603
LRRC7	p31.1	chr1-70050573; chr1-70050573
MAML2	q21	chr11-96074679; chr11-96074679
MIR1260B	q21	chr11-96074679; chr11-96074679; chr11-96074679; chr11-96074679
MPPED1	q13.2	chr22-43895736; chr22-43895736; chr22-43895736; chr22-43895736; chr22-43891954; chr22-43891954; chr22-43895535; chr22-43895535
POU2F1	q24.2, q24.1	chr1-167326586; chr1-167326586
PRKCA	q24.2	chr17-64603840; chr17-64603840; chr17-64795041; chr17-64795041; chr17-64794958; chr17-64794958
RERG	p12.3	chr12-15319480; chr12-15319480; chr12-15319479; chr12-15319479
RIC1	p24.1	chr9-5684305; chr9-5684305
RPL13AP8	q32.1	chr1-207080433; chr1-207080433
RTEL1	q13.33	chr20-62325013; chr20-62325013; chr20-62324869; chr20-62324869; chr20-62324811; chr20-62324811; chr20-62324892; chr20-62324892; chr20-62324873; chr20-62324873
RTEL1-TNFRSF6B	q13.33	chr20-62325013; chr20-62325013; chr20-62324869; chr20-62324869; chr20-62324811; chr20-62324811; chr20-62324892; chr20-62324892; chr20-62324873; chr20-62324873
SLC25A15	q14.11	chr13-41387255
SUN1	p22.3	chr7-902240; chr7-902240
SYPL1	q22.3	chr7-105741914; chr7-105741914; chr7-105741915; chr7-105741915; chr7-105741917; chr7-105741917; chr7-105741915; chr7-105741915; chr7-105741916; chr7-105741916; chr7-105741888; chr7-105741888; chr7-105741914; chr7-105741914; chr7-105741914; chr7-105741914; chr7-105741915; chr7-105741915; chr7-105741915; chr7-105741915; chr7-105741915; chr7-105741915; chr7-105741915; chr7-105741915; chr7-105741915; chr7-105741915; chr7-105741916; chr7-105741916; chr7-105741916; chr7-105741916; chr7-105741916; chr7-105741916; chr7-105741917; chr7-105741917; chr7-105741914; chr7-105741914; chr7-105741915; chr7-105741915; chr7-105741914; chr7-105741914; chr7-105741915; chr7-105741915; chr7-105741915; chr7-105741915; chr7-105741915; chr7-105741915; chr7-105741915; chr7-105741915; chr7-105741915; chr7-105741915; chr7-105741915; chr7-105741915; chr7-105741915; chr7-105741915; chr7-105741915; chr7-105741915; chr7-105741915; chr7-105741915; chr7-105741916; chr7-105741916; chr7-105741915; chr7-105741915; chr7-105741915; chr7-105741915; chr7-105741914; chr7-105741914; chr7-105741915; chr7-105741915; chr7-105741914; chr7-105741914; chr7-105741915; chr7-105741915
THSD7B	q22.1	chr2-137860974; chr2-137860974
TPTE2P5	q14.11	chr13-41387255; chr13-41387255
MAN2B2	p16.1	chr4-6580984; chr4-6580984

Table S6

gene	band	sample_list	number	pos_list
SYPL1	q22.3	CAEBV11;CAEBV14;CAEBV15;CAEBV18;CAEBV19;CAEBV2;CAEBV20;CAEBV23;CAEBV5;CAEBV6;CAEBV7;CAEBV8;CAEBV9;IM2;IM8	15	chr7-105741914;chr7-105741914;chr7-105741915;chr7-105741915;chr7-105741917;chr7-105741917;chr7-105741915;chr7-105741915;chr7-105741916;chr7-105741916;chr7-105741888;chr7-105741888;chr7-105741914;chr7-105741914;chr7-105741914;chr7-105741914;chr7-105741915;chr7-105741915;chr7-105741915;chr7-105741915;chr7-105741915;chr7-105741915;chr7-105741915;chr7-105741915;chr7-105741915;chr7-105741915;chr7-105741916;chr7-105741916;chr7-105741916;chr7-105741916;chr7-105741916;chr7-105741916;chr7-105741917;chr7-105741917;chr7-105741914;chr7-105741914;chr7-105741915;chr7-105741915;chr7-105741914;chr7-105741914;chr7-105741915;chr7-105741915;chr7-105741915;chr7-105741915;chr7-105741915;chr7-105741915;chr7-105741915;chr7-105741915;chr7-105741915;chr7-105741915;chr7-105741915;chr7-105741915;chr7-105741915;chr7-105741915;chr7-105741915;chr7-105741915;chr7-105741916;chr7-105741916;chr7-105741915;chr7-105741915;chr7-105741915;chr7-105741915;chr7-105741914;chr7-105741914;chr7-105741915;chr7-105741915;chr7-105741914;chr7-105741914;chr7-105741915;chr7-105741915
SETD3	q32.2	CAEBV1;CAEBV10;CAEBV13;CAEBV19;CAEBV21;CAEBV24;CAEBV3;CAEBV5;CAEBV6;CAEBV9	10	chr14-99887286;chr14-99887286;chr14-99887288;chr14-99887288;chr14-99887285;chr14-99887285;chr14-99887315;chr14-99887315;chr14-99887283;chr14-99887283;chr14-99887283;chr14-99887283;chr14-99887285;chr14-99887285;chr14-99887288;chr14-99887288;chr14-99887317;chr14-99887317;chr14-99887283;chr14-99887283;chr14-99887281;chr14-99887281;chr14-99887285;chr14-99887285;chr14-99887286;chr14-99887286;chr14-99887315;chr14-99887315;chr14-99887287;chr14-99887287;chr14-99887285;chr14-99887285
TMEM216	q12.2	CAEBV1;CAEBV16;CAEBV19;CAEBV21;CAEBV23;CAEBV3;CAEBV5;CAEBV8	8	chr11-61158723;chr11-61158723;chr11-61158720;chr11-61158720;chr11-61158724;chr11-61158724;chr11-61158721;chr11-61158721;chr11-61158723;chr11-61158723;chr11-61158720;chr11-61158720;chr11-61158723;chr11-61158723;chr11-61158723;chr11-61158723;chr11-61158723;chr11-61158723;chr11-61158728;chr11-61158728;chr11-61158757;chr11-61158757;chr11-61158720;chr11-61158720;chr11-61158723;chr11-61158723;chr11-61158720;chr11-61158720;chr11-61158726;chr11-61158726;chr11-61158721;chr11-61158721
ACSL3	q36.1	CAEBV18;CAEBV19;CAEBV2;CAEBV20;CAEBV6;CAEBV8;IM9	7	chr2-223731462;chr2-223731462;chr2-223731495;chr2-223731495;chr2-223731466;chr2-223731466;chr2-223731495;chr2-223731495;chr2-223731497;chr2-223731497;chr2-223731463;chr2-223731463;chr2-223731463;chr2-223731463;chr2-223731462;chr2-223731462;chr2-223731463;chr2-223731463;chr2-223731463;chr2-223731463
FER1L6-AS2	q24.13	CAEBV13;CAEBV18;CAEBV19;CAEBV20;CAEBV5	5	chr8-125184243;chr8-125184243;chr8-125184245;chr8-125184245;chr8-125184243;chr8-125184243;chr8-125184243;chr8-125184243;chr8-125184275;chr8-125184275;chr8-125184244;chr8-125184244;chr8-125184246;chr8-125184246;chr8-125184245;chr8-125184245;chr8-125184243;chr8-125184243;chr8-125184244;chr8-125184244;chr8-125184246;chr8-125184246;chr8-125184243;chr8-125184243
NGB	q24.3	CAEBV20;CAEBV21;CAEBV6;CAEBV8	4	chr14-77736168;chr14-77736168;chr14-77736169;chr14-77736169;chr14-77736168;chr14-77736168;chr14-77736168;chr14-77736168;chr14-77736166;chr14-77736166;chr14-77736167;chr14-77736167
MAGEB10	p21.3	CAEBV2;CAEBV3;CAEBV5;CAEBV9	4	chrX-27827589;chrX-27827589;chrX-27827589;chrX-27827589;chrX-27827646;chrX-27827646;chrX-27827601;chrX-27827601;chrX-27827601;chrX-27827601;chrX-27827601;chrX-27827601
RTEL1	q13.33	CAEBV17;IM10;IM11;IM12	4	chr20-62325013;chr20-62325013;chr20-62324869;chr20-62324869;chr20-62324811;chr20-62324811;chr20-62324892;chr20-62324892;chr20-62324873;chr20-62324873
MEGF8	q13.2	CAEBV13;CAEBV15;CAEBV19	3	chr19-42874992;chr19-42874992;chr19-42874993;chr19-42874993;chr19-42874994;chr19-42874994;chr19-42874994;chr19-42874994
PPP1R15B	q32.1	CAEBV19;CAEBV2;CAEBV20	3	chr1-204381175;chr1-204381175;chr1-204381173;chr1-204381173;chr1-204381175;chr1-204381175;chr1-204381173;chr1-204381173
SKIDA1	p12.31	CAEBV18;CAEBV19;CAEBV2	3	chr10-21811198;chr10-21811198;chr10-21811197;chr10-21811197;chr10-21811229;chr10-21811229
RNF213	q25.3	CAEBV2;CAEBV21;IM1	3	chr17-78289571;chr17-78289571;chr17-78289582;chr17-78289582;chr17-78287622;chr17-78287622;chr17-78289571;chr17-78289571
RTEL1-TNFRSF6B	q13.33	CAEBV17;IM10;IM12	3	chr20-62325013;chr20-62325013;chr20-62324869;chr20-62324869;chr20-62324811;chr20-62324811;chr20-62324892;chr20-62324892;chr20-62324873;chr20-62324873
CAMTA1	p36.31	CAEBV23;CAEBV6	2	chr1-7395201;chr1-7395201;chr1-7395201;chr1-7395201;chr1-7195715;chr1-7195715
AGBL1	q25.3	CAEBV13;CAEBV6	2	chr15-87348238;chr15-87348238;chr15-86967742;chr15-86967742
DIS3L2	q37.1	CAEBV16;CAEBV20	2	chr2-233112353;chr2-233112353;chr2-233097518;chr2-233097518
DLGAP1	p11.31	CAEBV2;CAEBV9	2	chr18-3898310;chr18-3898310;chr18-3832478;chr18-3832478
DOCK1	q26.2	CAEBV6;CAEBV9	2	chr10-129136147;chr10-129136147;chr10-128928014;chr10-128928014
AFG3L1P	q24.3	IM1;IM4	2	chr16-90053778;chr16-90053778;chr16-90053789;chr16-90053789
DPP4	q24.2	CAEBV6;IM12	2	chr2-162920027;chr2-162920027;chr2-162873671;chr2-162873671
GALNTL6	q34.1	CAEBV11;CAEBV20	2	chr4-173489339;chr4-173489339;chr4-173623283;chr4-173623283
GLB1L3	q25	CAEBV15;IM8	2	chr11-134178321;chr11-134178321;chr11-134178108;chr11-134178108
GRID2	q22.1,q22.2	CAEBV19;IM6	2	chr4-94243004;chr4-94243004;chr4-94630652;chr4-94630652
NHS	p22.13	CAEBV16;CAEBV19	2	chrX-17755543;chrX-17755569
PI4KA	q11.21	CAEBV11;CAEBV5	2	chr22-21172977;chr22-21172977;chr22-21111416;chr22-21111416
PRKCA	q24.2	CAEBV14;IM7	2	chr17-64603840;chr17-64603840;chr17-64795041;chr17-64795041;chr17-64794958;chr17-64794958
RAE1	q13.31	CAEBV19;CAEBV2	2	chr20-55925919;chr20-55925919;chr20-55925906;chr20-55925906;chr20-55925905;chr20-55925905
RTF1	q15.1	CAEBV2;CAEBV9	2	chr15-41718913;chr15-41718913;chr15-41718883;chr15-41718883
SCML1	p22.13	CAEBV16;CAEBV19	2	chrX-17755543;chrX-17755543;chrX-17755569;chrX-17755569;chrX-17755569;chrX-17755569

Table S7

Sheet: Sheet1

gene	chr	start	end	Mean methylation difference	adj.pval1	adj.pval2
DNMT3A	chr2	25517074	25517428	0.380092	1.59e-13	7.68e-26
EBF1	chr5	158524861	158524897	0.351973	1.59e-13	3.38e-16
OSBPL3	chr7	24840598	24840628	0.342929	1.59e-13	3.04e-26
CADPS2	chr7	122526696	122526729	0.33296	1.59e-13	1.43e-31
EPS15L1	chr19	16483496	16483640	0.328868	1.59e-13	7.55e-27
ACOXL	chr2	111605292	111605438	0.320024	1.59e-13	2.28e-27
GLIS1	chr1	54040524	54040624	0.317027	1.59e-13	9.41e-08
FLOT1	chr6	30704560	30704666	0.310141	1.59e-13	2.12e-26
CACNA1C	chr12	2496371	2496576	0.307462	1.59e-13	1.06e-27
DIAPH2	chrX	96726426	96726548	0.307224	1.59e-13	7e-27
DNAL1	chr14	74162510	74162528	0.280929	1.59e-13	3.9e-19
AHRR	chr5	317098	317243	0.280739	1.59e-13	3.31e-14
TBCD	chr17	80733199	80733240	0.271615	1.59e-13	8.31e-12
ZNF423	chr16	49892314	49892528	0.267286	1.59e-13	1.53e-25
SHROOM3	chr4	77610829	77610900	0.253813	1.59e-13	9.82e-43
ADARB1	chr21	46644438	46644482	0.253442	1.59e-13	7.61e-17
MICU2	chr13	22113671	22113726	0.252201	1.59e-13	2.02e-13
ASCC2	chr22	30221762	30221867	0.251781	1.59e-13	4.72e-19
ZMIZ1	chr10	81015063	81015242	0.247514	1.59e-13	2.21e-14
CAMTA1	chr1	7128816	7128915	0.244002	1.59e-13	2.06e-14
DGKA	chr12	56329577	56329904	0.239101	1.72e-13	2.35e-58
SETD3	chr14	99878737	99878823	0.238542	2.33e-13	5.75e-11
FOXN3	chr14	89977113	89977212	0.236445	1.59e-13	4.58e-10
LINC00598	chr13	41019943	41020009	0.23606	1.59e-13	1.23e-10
PACS2	chr14	105830649	105830667	0.232179	1.59e-13	4.49e-13
UBR4	chr1	19400963	19401154	0.231027	1.59e-13	3.76e-08
GLTSCR2	chr19	48256059	48256364	0.228423	4.24e-13	1.37e-10
UBASH3A	chr21	43834421	43834453	0.220188	1.96e-13	3.06e-09
RNF213	chr17	78235671	78235741	0.218546	1.59e-13	2.34e-14
KLHL29	chr2	23755784	23755813	0.2134	1.59e-13	8.27e-10
PDE10A	chr6	166074314	166074359	0.21282	1.59e-13	4.79e-22
TEAD1	chr11	12696648	12696739	0.211746	1.59e-13	1.38e-43
FBRSL1	chr12	133120415	133120543	0.209271	1.59e-13	2.51e-11
TESC	chr12	117489398	117489555	0.20796	1.59e-13	3.87e-10
EPHA6	chr3	96533392	96533414	0.206907	1.59e-13	7.31e-13
KIAA0922	chr4	154407998	154408181	0.206502	1.59e-13	5.77e-21
PRKCA-AS1, PRKCA	chr17	64395278	64395363	0.205254	1.59e-13	3.36e-13
ATG16L2	chr11	72533468	72533840	0.204686	1.59e-13	2.01e-38
PYM1	chr12	56322186	56322798	0.201993	1.59e-13	6.66e-30
OTUD7A	chr15	31775983	31776019	0.200033	1.59e-13	7.57e-22
PDE3A	chr12	20523088	20523122	0.196432	1.59e-13	8.38e-11

gene	chr	start	end	Mean methylation difference	adj.pval1	adj.pval2
TOX3	chr16	52580608	52580651	0.192406	1.59e-13	1.34e-08
CD52	chr1	26645487	26645803	0.189587	1.59e-13	3.84e-24
ESPNL	chr2	239017495	239017527	0.183084	2.04e-08	0.000113665
KIF1C	chr17	4902599	4902690	0.182097	6.48e-08	5.55e-05
NTSR1	chr20	61370897	61371090	0.180151	1.59e-13	7.29e-11
ASTN2	chr9	120177458	120177485	0.179736	1.59e-13	9.35e-14
DLGAP1	chr18	4455524	4455637	0.176544	1.59e-13	1.57e-11
ARSG	chr17	66346654	66346743	0.176463	1.59e-13	2.07e-11
CTNND2	chr5	11904340	11904455	0.175454	1.59e-13	1.08e-92
PFKFB3	chr10	6278321	6278477	0.174619	1.59e-13	4.77e-32
ZFHX3	chr16	73082221	73082238	0.173323	1.59e-13	2.66e-13
HRAT5	chr5	270587	270723	0.169834	1.59e-13	3.63e-13
BCL11A	chr2	60778025	60778047	0.169406	1.59e-13	2.23e-24
DUS3L	chr19	5785511	5785526	0.16632	4.09e-11	2.55e-08
PCOLCE2	chr3	142607502	142607527	0.165753	1.59e-13	9.69e-14
DNAH11	chr7	21582833	21582886	0.165355	1.59e-13	5.67e-18
IQCJ-SCHIP1, SCHIP1	chr3	159482791	159482811	0.164717	1.59e-13	1.42e-12
HIC1	chr17	1961617	1962291	0.164686	0	5.29e-89
KCNIP4	chr4	21950327	21950530	0.162584	1.59e-13	3.7e-71
THRA	chr17	38218104	38218182	0.159517	9.42e-13	1.9e-07
TP73-AS1	chr1	3663327	3663559	0.158984	6.17e-13	1.45e-07
MEGF8	chr19	42828061	42828109	0.158646	1.59e-13	1.18e-21
TMEM132C	chr12	128751903	128751934	0.158629	1.59e-13	5.68e-10
LIMCH1	chr4	41362770	41362817	0.157658	1.59e-13	8.84e-38
SLC7A10	chr19	33717739	33717930	0.155782	1.59e-13	1.09e-37
NALCN	chr13	102068704	102068731	0.153633	1.59e-13	1.08e-19
PKDCC	chr2	42275525	42275554	0.153451	1.28e-10	8.62e-10
ACSL3	chr2	223797596	223797642	0.150178	1.19e-07	1e-07
PDE4D	chr5	59189119	59189174	0.149784	1.59e-13	8.41e-12
FBLN2	chr3	13590610	13590695	0.149365	1.59e-13	3.42e-34
TJP1	chr15	30261119	30261167	0.147675	1.59e-13	6.95e-12
ADAMTS5	chr21	28338082	28338255	0.146867	1.59e-13	5.39e-27
SRC	chr20	36012804	36012865	0.146695	1.59e-13	1.3e-23
CNBD1	chr8	87978823	87978892	0.144874	1.54e-08	9.82e-05
MDGA2	chr14	48143866	48143950	0.143544	1.59e-13	1.97e-14
CTNNA2	chr2	79739773	79740169	0.143279	0	4.04e-61
ANXA6	chr5	150521374	150521474	0.14325	2.69e-11	1.33e-07
ADGRL3	chr4	62066008	62066123	0.143185	1.59e-13	2.32e-21
KANK3	chr19	8407974	8408033	0.142507	1.59e-13	2.7e-29
NHS	chrX	17394200	17394315	0.141841	4.24e-13	2.81e-08
SMURF2	chr17	62659218	62659313	0.141219	3.14e-13	9.47e-09
NELL1	chr11	20691037	20691097	0.141177	1.59e-13	6.68e-17
HIVEP2	chr6	143227067	143227131	0.13911	1.24e-11	2.07e-07
CILP2	chr19	19651064	19651274	0.137177	1.59e-13	4.24e-14
BCAR1	chr16	75285483	75285647	0.134951	1.59e-13	8.79e-34
RNF24	chr20	3996044	3996115	0.134508	1.59e-13	1.11e-37
PPP2R2C	chr4	6565480	6565517	0.133512	1.59e-13	4.84e-18
CCDC42	chr17	8648500	8648607	0.133294	1.59e-13	4.03e-14

gene	chr	start	end	Mean methylation difference	adj.pval1	adj.pval2
TRAK1	chr3	42249193	42249235	0.133127	6.7e-09	3.48e-06
BRAF	chr7	140624327	140624544	0.132905	1.6e-13	2.22e-24
ZNF692	chr1	249146342	249146811	0.132588	0.000158791	0.000255918
ADAMTS12	chr5	33892369	33892458	0.132274	1.59e-13	7.17e-10
FBXL7	chr5	15500270	15500474	0.128344	1.59e-13	4.31e-24
UNC5D	chr8	35093289	35093519	0.126851	1.59e-13	2.32e-29
TET1	chr10	70320146	70320179	0.125583	2.78e-13	2.48e-09
LRFN2	chr6	40555633	40555656	0.125335	2.07e-13	4.61e-10
SORCS2	chr4	7704960	7705120	0.123112	1.59e-13	2.21e-08
SLC6A4	chr17	28562498	28562537	0.122983	1.78e-12	4.17e-06
SEMA5A	chr5	9545294	9545888	0.122554	0	2.16e-49
TOX2	chr20	42544663	42544729	0.121958	1.59e-13	1.1e-14
DAB1	chr1	57889667	57889688	0.121825	1.59e-13	2.15e-19
CSMD3	chr8	114445797	114445864	0.121624	1.59e-13	1.77e-13
ENOX1	chr13	44360024	44360085	0.121438	1.59e-13	2.63e-15
CTDSPL	chr3	37903490	37903683	0.119272	1.65e-13	6.78e-76
ANKS1B	chr12	99289123	99289162	0.118144	1.59e-13	1.33e-10
CCNJL	chr5	159738931	159738938	0.117953	5.51e-09	3.83e-06
CCDC151	chr19	11534134	11534759	0.117316	2.35e-08	2.19e-05
KIAA1644	chr22	44710627	44710712	0.114652	5.8e-12	2.46e-10
COBL	chr7	51384229	51384557	0.114541	0	2.97e-95
CUX2	chr12	111472587	111472667	0.114136	1.59e-13	2.95e-31
RGS20	chr8	54789845	54789941	0.113915	1.59e-13	3.46e-15
PPARG	chr3	12330227	12330274	0.112723	1.94e-12	6.78e-08
KIAA1549	chr7	138665774	138665845	0.112197	1.59e-13	6.23e-28
TGOLN2	chr2	85553616	85553814	0.112115	1.59e-13	4.75e-53
SRRT	chr7	100482578	100482892	0.111519	1.59e-13	2.48e-11
UNC13A	chr19	17799183	17799236	0.111066	1.59e-13	1.79e-16
GALNTL6, LOC441052	chr4	172734394	172735021	0.110367	0	4.37e-59
GRM7	chr3	6902940	6903743	0.110333	0	8.1e-54
MECOM	chr3	168864347	168864384	0.110189	4.03e-10	2.18e-07
SRPX	chrX	38079695	38079856	0.109669	1.59e-13	7.84e-09
MYLK4	chr6	2746223	2746361	0.108889	2.98e-06	0.000168815
TMED3	chr15	79670267	79670280	0.10867	0.001456457	0.011471438
PLCH2	chr1	2427207	2427406	0.108467	5.12e-13	5.66e-08
DUSP15	chr20	30457786	30457929	0.10843	1.59e-13	5.94e-08
FAM226A	chrX	72162803	72163055	0.108331	2.4e-05	0.003119885
XKR6	chr8	11057979	11058060	0.10641	1.59e-13	1.75e-10
FZD8	chr10	35929807	35930010	0.105189	0	7.38e-90
DGKG	chr3	185912063	185912228	0.10497	1.59e-13	1.37e-14
DOCK1	chr10	128593739	128594084	0.104533	0	2.42e-96
KCNH8	chr3	19189773	19189946	0.10389	1.59e-13	7.75e-36
AKAP13	chr15	85944297	85944389	0.103524	4.22e-11	3.23e-07
CSMD1	chr8	4851656	4852077	0.10346	0	8.67e-57
NKAIN2	chr6	124124391	124124801	0.103116	0	2.49e-137
CPEB3	chr10	93999408	93999529	0.103039	1.59e-13	5.93e-19
GPHN	chr14	66975853	66975919	0.102593	1.59e-13	1.48e-14
DPYS	chr8	105479142	105479340	0.102553	1.59e-13	4.82e-12

gene	chr	start	end	Mean methylation difference	adj.pval1	adj.pval2
JPH2	chr20	42744649	42744728	0.102069	1.59e-13	5.04e-12
GSAP, LOC101927243	chr7	77044861	77044899	0.10156	3.95e-11	4.86e-10
MCF2L	chr13	113623456	113623510	0.10148	1.59e-13	2.97e-31
NFATC4	chr14	24836139	24836322	0.101288	4.24e-13	8.44e-17
HECW1	chr7	43153142	43153340	0.100958	1.59e-13	4.65e-12
DUX4L10	chr10	135478615	135478714	0.10029	1.59e-13	1.17e-12
BAGE2	chr21	11092338	11092434	-0.100735	8.3e-08	4.99e-06
AGMO	chr7	15587222	15587235	-0.100806	2.25e-07	0.000185226
LINC01716	chr20	55166674	55166791	-0.103064	4.71e-06	0.000559922
PAPPA2	chr1	176569759	176569798	-0.103127	4.06e-06	0.000571139
GRID2	chr4	93597422	93597627	-0.103707	1.96e-12	1.81e-07
CHST15	chr10	125852598	125852914	-0.104528	2.81e-13	1.36e-20
WDR72	chr15	54051700	54051771	-0.105198	2.81e-13	6.7e-09
TRDN	chr6	123680940	123681092	-0.105443	4.55e-06	0.000220667
NUBPL	chr14	32082809	32082958	-0.106187	0.011456693	0.043899266
EPB41L4A-AS2	chr5	111755300	111755413	-0.106441	2.23e-12	1.14e-10
LRRC20	chr10	72115119	72115259	-0.106497	5.12e-13	1.87e-10
AGBL1	chr15	86962440	86962621	-0.106553	3.61e-07	0.000351186
DOCK4	chr7	111648916	111648973	-0.107113	1.7e-10	7.03e-07
TPRXL	chr3	14101915	14102007	-0.109222	0.011456693	0.034141011
TAS1R2	chr1	19187410	19187768	-0.110081	3.17e-08	3.17e-06
ST3GAL2	chr16	70466865	70467149	-0.110627	1.67e-13	9.12e-08
GOLGA2P10	chr15	83141464	83141530	-0.111354	7.45e-05	0.000875231
CERCAM	chr9	131196620	131196699	-0.116367	0.00068491	0.013050591
CNTNAP4	chr16	76355456	76355482	-0.119148	1.72e-06	0.001163361
LMCD1	chr3	8544445	8544589	-0.119567	2.64e-07	0.000168204
LINC02141	chr16	60032119	60032160	-0.119944	1.48e-06	6.45e-06
ERC2	chr3	55589873	55589903	-0.120591	6.84e-13	4.17e-12
PKHD1	chr6	51739868	51739912	-0.121642	6.14e-09	8.2e-06
OTOF	chr2	26780373	26780974	-0.122666	4.47e-11	6.2e-06
CRB1	chr1	197438123	197438223	-0.124158	2.62e-05	0.002478701
MLC1	chr22	50499350	50499796	-0.124406	4.24e-13	1.16e-10
CHMP1B2P	chrX	79575074	79575163	-0.124557	5.12e-06	0.000131473
DNMT3B	chr20	31351309	31351387	-0.125633	1.39e-07	0.000101847
ANKRD30BL	chr2	132919458	132919793	-0.12653	1.59e-13	9.42e-25
AADACL2-AS1	chr3	151525415	151525465	-0.126741	8.68e-10	5.05e-06
NALCN-AS1	chr13	101628351	101628374	-0.127076	3.37e-07	1.19e-05
UTRN	chr6	144816417	144816517	-0.129614	1.71e-08	4.8e-05
TENM2	chr5	167285669	167285725	-0.130477	6.67e-11	4.55e-07
KIAA1147	chr7	141397267	141397308	-0.132027	4.12e-05	0.005238574
TSNARE1	chr8	143425756	143425854	-0.132871	4.24e-13	8.44e-13
ATP6V0A4	chr7	138452770	138452789	-0.13361	1.5e-08	3.97e-06
CAPN9	chr1	230912977	230913120	-0.135307	6.8e-08	4.05e-06
LOC100420587	chr19	29218140	29218326	-0.135494	5.22e-13	1.32e-12
DPH1	chr17	1932885	1933006	-0.137253	3.28e-05	0.009132142
LOC653786	chr16	22564926	22564964	-0.137292	1.48e-05	0.0003049
CELF3	chr1	151676487	151676507	-0.13829	9.34e-06	0.003470931
KALRN	chr3	124258881	124259062	-0.139921	3.27e-06	0.000382771

gene	chr	start	end	Mean methylation difference	adj.pval1	adj.pval2
LOC105377448	chr4	139775729	139775776	-0.140981	3.97e-13	2.66e-12
TRIM2	chr4	154144643	154144921	-0.143064	1.59e-13	1.11e-12
CTNNA3	chr10	68276701	68277201	-0.144291	7.34e-10	1.05e-06
UNQ6494	chr9	92255727	92255866	-0.144963	1.12e-10	5.23e-08
RAE1	chr20	55949956	55950134	-0.147899	8.14e-08	2.56e-05
RAB6A	chr11	73403419	73403518	-0.149519	3.62e-07	0.000906901
FUT9	chr6	96464015	96464064	-0.149762	2.81e-13	3.43e-14
KDM6A	chrX	44733023	44733055	-0.152596	2.81e-13	6.94e-24
PI4KA	chr22	21088386	21088525	-0.153055	2.81e-13	2.76e-10
VPS37B	chr12	123350735	123350807	-0.156554	1.87e-12	2.7e-06
LINC00486	chr2	33094380	33094556	-0.159185	1.96e-06	0.001178885
DBET	chr4	190987969	190988128	-0.159732	2.81e-13	1.3e-08
PIK3C2G	chr12	18754773	18755094	-0.166717	1.21e-12	1.74e-07
UNC93B1	chr11	67759091	67759288	-0.167791	2.81e-13	3.65e-44
DNAH2	chr17	7695394	7695574	-0.172181	5.49e-07	0.000856771
DOCK8	chr9	446270	446323	-0.174673	6.84e-13	9.29e-12
AGBL4, BEND5	chr1	49242459	49242511	-0.175469	2.81e-13	7.18e-12
NKAIN1	chr1	31654354	31654529	-0.178434	2.81e-13	3.15e-36
FRAS1	chr4	79027193	79027334	-0.17871	2.81e-13	5.64e-11
MYH6	chr14	23876727	23876824	-0.179407	0.000187195	0.012519537
LOC105378311, PCDH15	chr10	56349518	56349578	-0.191987	2.71e-08	3.51e-05
WDFY4	chr10	49932231	49932255	-0.19352	1.12e-12	4.53e-09
CNTNAP3P2	chr9	65602854	65602872	-0.194626	1.92e-07	0.000394382
CES5A	chr16	55880741	55880807	-0.195336	5.64e-08	1.4e-05
ETS1	chr11	128419199	128419345	-0.195919	2.81e-13	6.83e-12
NBDY	chrX	56804659	56806046	-0.197	5.22e-13	7.77e-30
MARCKS	chr6	114180822	114180888	-0.197561	2.81e-13	2.19e-12
GLB1L3	chr11	134156971	134157017	-0.19912	3.26e-06	0.000120791
ANKRD30B	chr18	14748116	14748220	-0.202712	2.81e-13	5.21e-16
ZFYVE9	chr1	52691974	52692100	-0.221153	1.12e-06	0.000139537
SLC12A9	chr7	100463326	100463392	-0.230818	1.59e-13	1.83e-11
KCNQ1OT1, KCNQ1	chr11	2720417	2721384	-0.282302	0	1.22e-146
DIS3L2	chr2	233186258	233186598	-0.286873	2.81e-13	1.16e-15

Sheet: RTGs

gene	chr	start	end	Mean methylation difference	adj.pval1	adj.pval2
CAMTA1	chr1	7128816	7128915	0.244002	1.59e-13	2.06e-14
SETD3	chr14	99878737	99878823	0.238542	2.33e-13	5.75e-11
RNF213	chr17	78235671	78235741	0.218546	1.59e-13	2.34e-14
PRKCA	chr17	64395278	64395363	0.205254	1.59e-13	3.36e-13
DLGAP1	chr18	4455524	4455637	0.176544	1.59e-13	1.57e-11
MEGF8	chr19	42828061	42828109	0.158646	1.59e-13	1.18e-21
NHS	chrX	17394200	17394315	0.141841	4.24e-13	2.81e-08
GALNTL6, LOC441052	chr4	172734394	172735021	0.110367	0	4.37e-59
DOCK1	chr10	128593739	128594084	0.104533	0	2.42e-96
GRID2	chr4	93597422	93597627	-0.103707	1.96e-12	1.81e-07
AGBL1	chr15	86962440	86962621	-0.106553	3.61e-07	0.000351186
RAE1	chr20	55949956	55950134	-0.147899	8.14e-08	2.56e-05
PI4KA	chr22	21088386	21088525	-0.153055	2.81e-13	2.76e-10
GLB1L3	chr11	134156971	134157017	-0.19912	3.26e-06	0.000120791

Table S8

Sheet: Sheet1

Row 2: gene = BBLF4

gene: BBLF4

sample: CAEBV1, CAEBV11, CAEBV14, CAEBV16, CAEBV18, CAEBV19, CAEBV2, CAEBV20, CAEBV21, CAEBV5, CAEBV6, CAEBV8, CAEBV9, IM13, IM7

pos: gammaEBV4-100012, gammaEBV4-100015, gammaEBV4-100017, gammaEBV4-100032, gammaEBV4-100470, gammaEBV4-100929, gammaEBV4-101078, gammaEBV4-101090, gammaEBV4-101143, gammaEBV4-101244, gammaEBV4-101896, gammaEBV4-101996, gammaEBV4-102132, gammaEBV4-102279, gammaEBV4-102548, gammaEBV4-102833, gammaEBV4-102972, gammaEBV4-103167, gammaEBV4-103346, gammaEBV4-103649, gammaEBV4-103838, gammaEBV4-97584, gammaEBV4-97632, gammaEBV4-98321, gammaEBV4-98485, gammaEBV4-98607, gammaEBV4-98649, gammaEBV4-98857, gammaEBV4-99257, gammaEBV4-99261, gammaEBV4-99275, gammaEBV4-99399, gammaEBV4-99402, gammaEBV4-99405, gammaEBV4-99422, gammaEBV4-99423, gammaEBV4-99440, gammaEBV4-99441, gammaEBV4-99535

Row 3: gene = LMP-2B

gene: LMP-2B

sample: CAEBV1, CAEBV10, CAEBV11, CAEBV12, CAEBV13, CAEBV14, CAEBV15, CAEBV16, CAEBV17, CAEBV18, CAEBV19, CAEBV2, CAEBV20, CAEBV21, CAEBV22, CAEBV23, CAEBV24, CAEBV3, CAEBV3-2, CAEBV4, CAEBV5, CAEBV6, CAEBV7, CAEBV8, CAEBV9, IM1, IM2, IM10, IM11, IM12, IM13, IM14, IM15, IM16, IM17, IM4, IM5, IM6, IM7, IM8, IM9

pos: gammaEBV4-100012, gammaEBV4-100015, gammaEBV4-100017, gammaEBV4-100032, gammaEBV4-100470, gammaEBV4-100929, gammaEBV4-101078, gammaEBV4-101090, gammaEBV4-101143, gammaEBV4-101244, gammaEBV4-101896, gammaEBV4-101996, gammaEBV4-102132, gammaEBV4-102279, gammaEBV4-102548, gammaEBV4-102833, gammaEBV4-102972, gammaEBV4-103167, gammaEBV4-103346, gammaEBV4-103649, gammaEBV4-103838, gammaEBV4-104080, gammaEBV4-104243, gammaEBV4-104986, gammaEBV4-104994, gammaEBV4-105325, gammaEBV4-10539, gammaEBV4-105501, gammaEBV4-105542, gammaEBV4-105598, gammaEBV4-106045, gammaEBV4-106231, gammaEBV4-106388, gammaEBV4-106430, gammaEBV4-107019, gammaEBV4-107473, gammaEBV4-107738, gammaEBV4-108135, gammaEBV4-108164, gammaEBV4-108165, gammaEBV4-108219, gammaEBV4-108226, gammaEBV4-108228, gammaEBV4-109037, gammaEBV4-109074, gammaEBV4-10920, gammaEBV4-110375, gammaEBV4-110393, gammaEBV4-110468, gammaEBV4-110538, gammaEBV4-110756, gammaEBV4-110937, gammaEBV4-111238, gammaEBV4-112229, gammaEBV4-112312, gammaEBV4-112741, gammaEBV4-113146, gammaEBV4-11339, gammaEBV4-113587, gammaEBV4-113631, gammaEBV4-113886, gammaEBV4-113897, gammaEBV4-114033, gammaEBV4-114134, gammaEBV4-114178, gammaEBV4-114596, gammaEBV4-114785, gammaEBV4-115055, gammaEBV4-115222, gammaEBV4-115343, gammaEBV4-115861, gammaEBV4-115891, gammaEBV4-115990, gammaEBV4-116738, gammaEBV4-117372, gammaEBV4-117462, gammaEBV4-117583, gammaEBV4-117880, gammaEBV4-117936, gammaEBV4-117941, gammaEBV4-118378, gammaEBV4-118380, gammaEBV4-118437, gammaEBV4-118443, gammaEBV4-118497, gammaEBV4-118554, gammaEBV4-118559, gammaEBV4-119450, gammaEBV4-11955, gammaEBV4-119719, gammaEBV4-119738, gammaEBV4-119764, gammaEBV4-119766, gammaEBV4-120040, gammaEBV4-120837, gammaEBV4-121112, gammaEBV4-121131, gammaEBV4-122449, gammaEBV4-122641, gammaEBV4-123006, gammaEBV4-123073, gammaEBV4-123123, gammaEBV4-123468, gammaEBV4-123550, gammaEBV4-123573, gammaEBV4-123594, gammaEBV4-123722, gammaEBV4-123726, gammaEBV4-123773, gammaEBV4-123786, gammaEBV4-123790, gammaEBV4-123855, gammaEBV4-124007, gammaEBV4-124331, gammaEBV4-124333, gammaEBV4-124335, gammaEBV4-124336, gammaEBV4-124337, gammaEBV4-124338, gammaEBV4-124339, gammaEBV4-124340, gammaEBV4-124342, gammaEBV4-124343, gammaEBV4-124344, gammaEBV4-124345, gammaEBV4-124346, gammaEBV4-124347, gammaEBV4-124348, gammaEBV4-124349, gammaEBV4-124350, gammaEBV4-124351, gammaEBV4-124352, gammaEBV4-124356, gammaEBV4-124357, gammaEBV4-124373, gammaEBV4-124681, gammaEBV4-124988, gammaEBV4-125072, gammaEBV4-125106, gammaEBV4-125211, gammaEBV4-125323, gammaEBV4-125330, gammaEBV4-125786, gammaEBV4-125876, gammaEBV4-125881, gammaEBV4-126135, gammaEBV4-126219, gammaEBV4-126402, gammaEBV4-127266, gammaEBV4-127347, gammaEBV4-127645, gammaEBV4-127663, gammaEBV4-127669, gammaEBV4-127827, gammaEBV4-128010, gammaEBV4-128770, gammaEBV4-128837, gammaEBV4-129213, gammaEBV4-129395, gammaEBV4-130293, gammaEBV4-130302, gammaEBV4-130543, gammaEBV4-130568, gammaEBV4-130569, gammaEBV4-130696, gammaEBV4-130723, gammaEBV4-130930, gammaEBV4-131300, gammaEBV4-131367, gammaEBV4-131675, gammaEBV4-132155, gammaEBV4-132194, gammaEBV4-132223, gammaEBV4-132224, gammaEBV4-132232, gammaEBV4-132437, gammaEBV4-133088, gammaEBV4-13361, gammaEBV4-133974, gammaEBV4-134311, gammaEBV4-134348, gammaEBV4-134460, gammaEBV4-134537, gammaEBV4-134765, gammaEBV4-134834, gammaEBV4-135676, gammaEBV4-135687, gammaEBV4-135692, gammaEBV4-135852, gammaEBV4-135865, gammaEBV4-136075, gammaEBV4-136560, gammaEBV4-136578, gammaEBV4-136653, gammaEBV4-136754, gammaEBV4-136896, gammaEBV4-136927, gammaEBV4-137017, gammaEBV4-137032, gammaEBV4-137120, gammaEBV4-137135, gammaEBV4-137142, gammaEBV4-137148, gammaEBV4-137151, gammaEBV4-137170, gammaEBV4-137227, gammaEBV4-137268, gammaEBV4-138016, gammaEBV4-138281, gammaEBV4-138618, gammaEBV4-138625, gammaEBV4-138872, gammaEBV4-138960, gammaEBV4-139071, gammaEBV4-139128, gammaEBV4-139154, gammaEBV4-139166, gammaEBV4-139196, gammaEBV4-139198, gammaEBV4-139202, gammaEBV4-139204, gammaEBV4-139207, gammaEBV4-139209, gammaEBV4-139214, gammaEBV4-139216, gammaEBV4-139438, gammaEBV4-139502, gammaEBV4-140335, gammaEBV4-140663, gammaEBV4-142143, gammaEBV4-142423, gammaEBV4-144869, gammaEBV4-145085, gammaEBV4-145352, gammaEBV4-145444, gammaEBV4-146172, gammaEBV4-146843, gammaEBV4-147843, gammaEBV4-147959, gammaEBV4-148630, gammaEBV4-148650, gammaEBV4-148658, gammaEBV4-148892, gammaEBV4-148962, gammaEBV4-148975, gammaEBV4-149020, gammaEBV4-149096, gammaEBV4-149397, gammaEBV4-149494, gammaEBV4-149760, gammaEBV4-150546, gammaEBV4-150551, gammaEBV4-150702, gammaEBV4-150749, gammaEBV4-151271, gammaEBV4-151272, gammaEBV4-152545, gammaEBV4-152557, gammaEBV4-152613, gammaEBV4-153004, gammaEBV4-153077, gammaEBV4-153117, gammaEBV4-153180, gammaEBV4-153617, gammaEBV4-153639, gammaEBV4-153725, gammaEBV4-153763, gammaEBV4-153860, gammaEBV4-153864, gammaEBV4-153952, gammaEBV4-154055, gammaEBV4-154299, gammaEBV4-154429, gammaEBV4-154453, gammaEBV4-154654, gammaEBV4-154720, gammaEBV4-154982, gammaEBV4-155166, gammaEBV4-155263, gammaEBV4-155584, gammaEBV4-155832, gammaEBV4-156147, gammaEBV4-156363, gammaEBV4-156375, gammaEBV4-156562, gammaEBV4-156606, gammaEBV4-157058, gammaEBV4-157364, gammaEBV4-157434, gammaEBV4-157853, gammaEBV4-159158, gammaEBV4-159376, gammaEBV4-160788, gammaEBV4-16098, gammaEBV4-161004, gammaEBV4-161324, gammaEBV4-161348, gammaEBV4-161445, gammaEBV4-161533, gammaEBV4-162029, gammaEBV4-162140, gammaEBV4-162204, gammaEBV4-162480, gammaEBV4-162703, gammaEBV4-162758, gammaEBV4-163569, gammaEBV4-163668, gammaEBV4-163804, gammaEBV4-163829, gammaEBV4-164100, gammaEBV4-164161, gammaEBV4-164382, gammaEBV4-164387, gammaEBV4-164551, gammaEBV4-164586, gammaEBV4-164676, gammaEBV4-164692, gammaEBV4-164787, gammaEBV4-164982, gammaEBV4-165051, gammaEBV4-165155, gammaEBV4-165639, gammaEBV4-165807, gammaEBV4-166017, gammaEBV4-166116, gammaEBV4-166237, gammaEBV4-166370, gammaEBV4-166656, gammaEBV4-166695, gammaEBV4-166813, gammaEBV4-167362, gammaEBV4-167370, gammaEBV4-167399, gammaEBV4-167404, gammaEBV4-167437, gammaEBV4-167488, gammaEBV4-167506, gammaEBV4-168640, gammaEBV4-169257, gammaEBV4-169350, gammaEBV4-169430, gammaEBV4-169442, gammaEBV4-169460, gammaEBV4-169485, gammaEBV4-169565, gammaEBV4-171084, gammaEBV4-171244, gammaEBV4-171498, gammaEBV4-171768, gammaEBV4-171773, gammaEBV4-171783, gammaEBV4-187, gammaEBV4-19491, gammaEBV4-22574, gammaEBV4-2403, gammaEBV4-26126, gammaEBV4-2768, gammaEBV4-2769, gammaEBV4-2958, gammaEBV4-336, gammaEBV4-3452, gammaEBV4-34536, gammaEBV4-35458, gammaEBV4-35769, gammaEBV4-36014, gammaEBV4-36209, gammaEBV4-36221, gammaEBV4-36265, gammaEBV4-36307, gammaEBV4-36315, gammaEBV4-36322, gammaEBV4-36355, gammaEBV4-36356, gammaEBV4-36360, gammaEBV4-36368, gammaEBV4-36389, gammaEBV4-36390, gammaEBV4-36391, gammaEBV4-36393, gammaEBV4-36394, gammaEBV4-36397, gammaEBV4-36399, gammaEBV4-3640, gammaEBV4-36401, gammaEBV4-36402, gammaEBV4-36403, gammaEBV4-36405, gammaEBV4-36406, gammaEBV4-36408, gammaEBV4-36410, gammaEBV4-36411, gammaEBV4-36414, gammaEBV4-36415, gammaEBV4-36417, gammaEBV4-36420, gammaEBV4-36421, gammaEBV4-36423, gammaEBV4-36424, gammaEBV4-36425, gammaEBV4-36429, gammaEBV4-36432, gammaEBV4-36433, gammaEBV4-36435, gammaEBV4-36436, gammaEBV4-36437, gammaEBV4-36438, gammaEBV4-36439, gammaEBV4-36441, gammaEBV4-36442, gammaEBV4-36445, gammaEBV4-36446, gammaEBV4-36447, gammaEBV4-36448, gammaEBV4-36449, gammaEBV4-36450, gammaEBV4-36451, gammaEBV4-36453, gammaEBV4-36454, gammaEBV4-36455, gammaEBV4-36457, gammaEBV4-36458, gammaEBV4-36459, gammaEBV4-36460, gammaEBV4-36462, gammaEBV4-36463, gammaEBV4-36464, gammaEBV4-36465, gammaEBV4-36466, gammaEBV4-36468, gammaEBV4-36469, gammaEBV4-36470, gammaEBV4-36472, gammaEBV4-36475, gammaEBV4-36476, gammaEBV4-36477, gammaEBV4-36478, gammaEBV4-36479, gammaEBV4-36481, gammaEBV4-36482, gammaEBV4-36483, gammaEBV4-36486, gammaEBV4-36487, gammaEBV4-36489, gammaEBV4-36490, gammaEBV4-36491, gammaEBV4-36492, gammaEBV4-36495, gammaEBV4-36497, gammaEBV4-36498, gammaEBV4-36499, gammaEBV4-36501, gammaEBV4-36502, gammaEBV4-36507, gammaEBV4-36521, gammaEBV4-36528, gammaEBV4-36543, gammaEBV4-36558, gammaEBV4-36564, gammaEBV4-36710, gammaEBV4-36712, gammaEBV4-36745, gammaEBV4-36781, gammaEBV4-36801, gammaEBV4-36816,

gammaEBV4-36834, gammaEBV4-36855, gammaEBV4-36856, gammaEBV4-36860, gammaEBV4-36865, gammaEBV4-36869, gammaEBV4-36882, gammaEBV4-36885, gammaEBV4-36897, gammaEBV4-36924, gammaEBV4-36940, gammaEBV4-36942, gammaEBV4-36946, gammaEBV4-36947, gammaEBV4-36961, gammaEBV4-36965, gammaEBV4-36993, gammaEBV4-37009, gammaEBV4-37019, gammaEBV4-37033, gammaEBV4-37044, gammaEBV4-37087, gammaEBV4-37113, gammaEBV4-37116, gammaEBV4-37130, gammaEBV4-3720, gammaEBV4-37270, gammaEBV4-37490, gammaEBV4-3754, gammaEBV4-3758, gammaEBV4-37678, gammaEBV4-3775, gammaEBV4-37924, gammaEBV4-37979, gammaEBV4-38989, gammaEBV4-39482, gammaEBV4-39873, gammaEBV4-39899, gammaEBV4-39903, gammaEBV4-39908, gammaEBV4-39915, gammaEBV4-39925, gammaEBV4-40194, gammaEBV4-40213, gammaEBV4-40219, gammaEBV4-40220, gammaEBV4-40224, gammaEBV4-40225, gammaEBV4-40263, gammaEBV4-40282, gammaEBV4-4082, gammaEBV4-413, gammaEBV4-41499, gammaEBV4-41553, gammaEBV4-416, gammaEBV4-41648, gammaEBV4-41794, gammaEBV4-41821, gammaEBV4-43009, gammaEBV4-43140, gammaEBV4-43141, gammaEBV4-43142, gammaEBV4-43145, gammaEBV4-43146, gammaEBV4-43373, gammaEBV4-43465, gammaEBV4-43477, gammaEBV4-43509, gammaEBV4-43624, gammaEBV4-43684, gammaEBV4-43981, gammaEBV4-44109, gammaEBV4-44155, gammaEBV4-44242, gammaEBV4-44658, gammaEBV4-45678, gammaEBV4-45790, gammaEBV4-45805, gammaEBV4-45860, gammaEBV4-46042, gammaEBV4-46062, gammaEBV4-46257, gammaEBV4-46472, gammaEBV4-46706, gammaEBV4-46912, gammaEBV4-47220, gammaEBV4-47236, gammaEBV4-47240, gammaEBV4-47908, gammaEBV4-47955, gammaEBV4-48227, gammaEBV4-48251, gammaEBV4-48335, gammaEBV4-48519, gammaEBV4-48797, gammaEBV4-48983, gammaEBV4-49102, gammaEBV4-4944, gammaEBV4-49719, gammaEBV4-50133, gammaEBV4-50242, gammaEBV4-5080, gammaEBV4-51166, gammaEBV4-51500, gammaEBV4-51571, gammaEBV4-5182, gammaEBV4-52091, gammaEBV4-52205, gammaEBV4-52211, gammaEBV4-52227, gammaEBV4-52259, gammaEBV4-52372, gammaEBV4-52814, gammaEBV4-52837, gammaEBV4-52952, gammaEBV4-53724, gammaEBV4-53902, gammaEBV4-5406, gammaEBV4-54149, gammaEBV4-54243, gammaEBV4-54255, gammaEBV4-54491, gammaEBV4-54802, gammaEBV4-54921, gammaEBV4-55000, gammaEBV4-55730, gammaEBV4-55780, gammaEBV4-55793, gammaEBV4-55811, gammaEBV4-55869, gammaEBV4-55887, gammaEBV4-55914, gammaEBV4-56237, gammaEBV4-56566, gammaEBV4-5665, gammaEBV4-56866, gammaEBV4-5739, gammaEBV4-57429, gammaEBV4-5751, gammaEBV4-5758, gammaEBV4-5762, gammaEBV4-57632, gammaEBV4-57708, gammaEBV4-57740, gammaEBV4-57806, gammaEBV4-57842, gammaEBV4-57869, gammaEBV4-57870, gammaEBV4-57879, gammaEBV4-57880, gammaEBV4-57896, gammaEBV4-57897, gammaEBV4-5793, gammaEBV4-5796, gammaEBV4-5798, gammaEBV4-5799, gammaEBV4-5802, gammaEBV4-5803, gammaEBV4-5804, gammaEBV4-58047, gammaEBV4-5805, gammaEBV4-5807, gammaEBV4-5808, gammaEBV4-5809, gammaEBV4-5810, gammaEBV4-58301, gammaEBV4-58407, gammaEBV4-58634, gammaEBV4-58692, gammaEBV4-58785, gammaEBV4-58867, gammaEBV4-59115, gammaEBV4-59448, gammaEBV4-59672, gammaEBV4-59799, gammaEBV4-59923, gammaEBV4-59992, gammaEBV4-60095, gammaEBV4-60374, gammaEBV4-60398, gammaEBV4-61987, gammaEBV4-62009, gammaEBV4-62182, gammaEBV4-62247, gammaEBV4-62391, gammaEBV4-62449, gammaEBV4-62499, gammaEBV4-62677, gammaEBV4-62690, gammaEBV4-62774, gammaEBV4-62778, gammaEBV4-62916, gammaEBV4-63356, gammaEBV4-63580, gammaEBV4-63692, gammaEBV4-64008, gammaEBV4-64199, gammaEBV4-64224, gammaEBV4-64324, gammaEBV4-64571, gammaEBV4-64795, gammaEBV4-64976, gammaEBV4-65127, gammaEBV4-65205, gammaEBV4-65433, gammaEBV4-65450, gammaEBV4-65685, gammaEBV4-65718, gammaEBV4-65785, gammaEBV4-65950, gammaEBV4-66061, gammaEBV4-66069, gammaEBV4-66363, gammaEBV4-66405, gammaEBV4-66612, gammaEBV4-66809, gammaEBV4-67279, gammaEBV4-67485, gammaEBV4-67505, gammaEBV4-67749, gammaEBV4-68057, gammaEBV4-68261, gammaEBV4-68279, gammaEBV4-68497, gammaEBV4-68583, gammaEBV4-68589, gammaEBV4-68612, gammaEBV4-68646, gammaEBV4-68657, gammaEBV4-68729, gammaEBV4-68748, gammaEBV4-69148, gammaEBV4-69192, gammaEBV4-69276, gammaEBV4-69611, gammaEBV4-69904, gammaEBV4-69950, gammaEBV4-7019, gammaEBV4-70330, gammaEBV4-7035, gammaEBV4-70538, gammaEBV4-71139, gammaEBV4-71229, gammaEBV4-71292, gammaEBV4-71344, gammaEBV4-71546, gammaEBV4-71618, gammaEBV4-71671, gammaEBV4-72053, gammaEBV4-7210, gammaEBV4-72289, gammaEBV4-7253, gammaEBV4-72560, gammaEBV4-72580, gammaEBV4-72690, gammaEBV4-72723, gammaEBV4-72979, gammaEBV4-73029, gammaEBV4-73305, gammaEBV4-73366, gammaEBV4-73378, gammaEBV4-73577, gammaEBV4-73713, gammaEBV4-73903, gammaEBV4-73989, gammaEBV4-74183, gammaEBV4-74250, gammaEBV4-74856, gammaEBV4-74882, gammaEBV4-75292, gammaEBV4-75357, gammaEBV4-75429, gammaEBV4-75555, gammaEBV4-75694, gammaEBV4-75699, gammaEBV4-76014, gammaEBV4-76449, gammaEBV4-76489, gammaEBV4-76525, gammaEBV4-76598, gammaEBV4-7691, gammaEBV4-76943, gammaEBV4-7705, gammaEBV4-77179, gammaEBV4-77304, gammaEBV4-77373, gammaEBV4-77401, gammaEBV4-77404, gammaEBV4-77419, gammaEBV4-77613, gammaEBV4-77647, gammaEBV4-77672, gammaEBV4-77826, gammaEBV4-78055, gammaEBV4-78268, gammaEBV4-78767, gammaEBV4-78969, gammaEBV4-79898, gammaEBV4-80010, gammaEBV4-80158, gammaEBV4-80180, gammaEBV4-80333, gammaEBV4-80380, gammaEBV4-80419, gammaEBV4-80444, gammaEBV4-8048, gammaEBV4-81096, gammaEBV4-81378, gammaEBV4-81601, gammaEBV4-81671, gammaEBV4-81722, gammaEBV4-81794, gammaEBV4-81818, gammaEBV4-81950, gammaEBV4-81952, gammaEBV4-81957, gammaEBV4-81959, gammaEBV4-82027, gammaEBV4-82041, gammaEBV4-82056, gammaEBV4-82082, gammaEBV4-82089, gammaEBV4-82233, gammaEBV4-82234, gammaEBV4-82250, gammaEBV4-82253, gammaEBV4-82254, gammaEBV4-82259, gammaEBV4-82274, gammaEBV4-82347, gammaEBV4-82356, gammaEBV4-82358, gammaEBV4-82363, gammaEBV4-82366, gammaEBV4-82414, gammaEBV4-82418, gammaEBV4-8244, gammaEBV4-82557, gammaEBV4-82685, gammaEBV4-82902, gammaEBV4-82911, gammaEBV4-83123, gammaEBV4-83632, gammaEBV4-84936, gammaEBV4-8500, gammaEBV4-85018, gammaEBV4-85048, gammaEBV4-85131, gammaEBV4-85235, gammaEBV4-8555, gammaEBV4-85666, gammaEBV4-87597, gammaEBV4-87818, gammaEBV4-87870, gammaEBV4-87967, gammaEBV4-88158, gammaEBV4-88365, gammaEBV4-88383, gammaEBV4-88385, gammaEBV4-88388, gammaEBV4-88390, gammaEBV4-88393, gammaEBV4-88397, gammaEBV4-88400, gammaEBV4-88401, gammaEBV4-88402, gammaEBV4-88405, gammaEBV4-88409, gammaEBV4-88410, gammaEBV4-88411, gammaEBV4-88412, gammaEBV4-88413, gammaEBV4-88414, gammaEBV4-88415, gammaEBV4-88416, gammaEBV4-88417, gammaEBV4-88419, gammaEBV4-88420, gammaEBV4-88421, gammaEBV4-88423, gammaEBV4-88424, gammaEBV4-88425, gammaEBV4-88426, gammaEBV4-88427, gammaEBV4-88429, gammaEBV4-88431, gammaEBV4-88432, gammaEBV4-88433, gammaEBV4-88435, gammaEBV4-88436, gammaEBV4-88437, gammaEBV4-88438, gammaEBV4-88439, gammaEBV4-88440, gammaEBV4-88442, gammaEBV4-88444, gammaEBV4-88445, gammaEBV4-88446, gammaEBV4-88447, gammaEBV4-88448, gammaEBV4-88449, gammaEBV4-88450, gammaEBV4-88451, gammaEBV4-88452, gammaEBV4-88453, gammaEBV4-88454, gammaEBV4-88455, gammaEBV4-88456, gammaEBV4-88457, gammaEBV4-88458, gammaEBV4-88459, gammaEBV4-88462, gammaEBV4-88463, gammaEBV4-88464, gammaEBV4-88465, gammaEBV4-88466, gammaEBV4-88467, gammaEBV4-88469, gammaEBV4-88470, gammaEBV4-88471, gammaEBV4-88472, gammaEBV4-88474, gammaEBV4-88476, gammaEBV4-88478, gammaEBV4-88483, gammaEBV4-88486, gammaEBV4-88495, gammaEBV4-88497, gammaEBV4-88498, gammaEBV4-88499, gammaEBV4-88500, gammaEBV4-88501, gammaEBV4-88502, gammaEBV4-88503, gammaEBV4-88504, gammaEBV4-88506, gammaEBV4-88507, gammaEBV4-88508, gammaEBV4-88511, gammaEBV4-88512, gammaEBV4-88514, gammaEBV4-88516, gammaEBV4-88527, gammaEBV4-88528, gammaEBV4-88529, gammaEBV4-88597, gammaEBV4-88834, gammaEBV4-8901, gammaEBV4-89231, gammaEBV4-89456, gammaEBV4-9007, gammaEBV4-90140, gammaEBV4-90282, gammaEBV4-90294, gammaEBV4-90298, gammaEBV4-90302, gammaEBV4-90304, gammaEBV4-90308, gammaEBV4-90311, gammaEBV4-90312, gammaEBV4-90313, gammaEBV4-90315, gammaEBV4-90317, gammaEBV4-90319, gammaEBV4-90320, gammaEBV4-90321, gammaEBV4-90322, gammaEBV4-90324, gammaEBV4-90332, gammaEBV4-90339, gammaEBV4-90340, gammaEBV4-90341, gammaEBV4-90342, gammaEBV4-90343, gammaEBV4-90347, gammaEBV4-90348, gammaEBV4-90349, gammaEBV4-90350, gammaEBV4-90351, gammaEBV4-90352, gammaEBV4-90353, gammaEBV4-90354, gammaEBV4-90355, gammaEBV4-90356, gammaEBV4-90357, gammaEBV4-90358, gammaEBV4-90359, gammaEBV4-90361, gammaEBV4-90370, gammaEBV4-90371, gammaEBV4-90372, gammaEBV4-90373, gammaEBV4-90376, gammaEBV4-90378, gammaEBV4-91150, gammaEBV4-91422, gammaEBV4-91471, gammaEBV4-91480, gammaEBV4-91705, gammaEBV4-93050, gammaEBV4-93159, gammaEBV4-93490, gammaEBV4-9391, gammaEBV4-94085, gammaEBV4-94177, gammaEBV4-94212, gammaEBV4-9426, gammaEBV4-9427, gammaEBV4-9428, gammaEBV4-9430, gammaEBV4-9432, gammaEBV4-9433, gammaEBV4-9434, gammaEBV4-9437, gammaEBV4-9438, gammaEBV4-9439, gammaEBV4-9442, gammaEBV4-9444, gammaEBV4-9446, gammaEBV4-9447, gammaEBV4-94589, gammaEBV4-9518, gammaEBV4-95186, gammaEBV4-95199, gammaEBV4-95240, gammaEBV4-95258, gammaEBV4-95261, gammaEBV4-95588, gammaEBV4-95590, gammaEBV4-95666, gammaEBV4-95906, gammaEBV4-95942, gammaEBV4-95961, gammaEBV4-95963, gammaEBV4-95969, gammaEBV4-95974, gammaEBV4-95983, gammaEBV4-95984, gammaEBV4-95999, gammaEBV4-96000, gammaEBV4-96003, gammaEBV4-96004, gammaEBV4-96006, gammaEBV4-96008, gammaEBV4-96010, gammaEBV4-96024, gammaEBV4-96029, gammaEBV4-96038, gammaEBV4-96055, gammaEBV4-96064, gammaEBV4-96065, gammaEBV4-96070, gammaEBV4-96073, gammaEBV4-96078, gammaEBV4-96096, gammaEBV4-96114, gammaEBV4-96117, gammaEBV4-96118, gammaEBV4-96123, gammaEBV4-96127, gammaEBV4-96128, gammaEBV4-96142, gammaEBV4-96181, gammaEBV4-96193, gammaEBV4-96197, gammaEBV4-96199, gammaEBV4-962, gammaEBV4-96203, gammaEBV4-96205, gammaEBV4-96207, gammaEBV4-96208, gammaEBV4-96240, gammaEBV4-96254, gammaEBV4-96259, gammaEBV4-96266, gammaEBV4-96276, gammaEBV4-96279, gammaEBV4-96280, gammaEBV4-96281, gammaEBV4-96289, gammaEBV4-96296, gammaEBV4-96298, gammaEBV4-96302, gammaEBV4-96306, gammaEBV4-96310, gammaEBV4-96313, gammaEBV4-96314, gammaEBV4-96315, gammaEBV4-96316, gammaEBV4-96319, gammaEBV4-96328, gammaEBV4-96333, gammaEBV4-96334, gammaEBV4-96337, gammaEBV4-96346, gammaEBV4-96349, gammaEBV4-96357, gammaEBV4-96364, gammaEBV4-96365, gammaEBV4-96367, gammaEBV4-96370, gammaEBV4-96373, gammaEBV4-96378, gammaEBV4-96379, gammaEBV4-96380, gammaEBV4-96382, gammaEBV4-96387, gammaEBV4-96388, gammaEBV4-96394, gammaEBV4-96396, gammaEBV4-96399, gammaEBV4-96400, gammaEBV4-96403, gammaEBV4-96405, gammaEBV4-96406, gammaEBV4-96410, gammaEBV4-96414, gammaEBV4-96415, gammaEBV4-96416, gammaEBV4-96417, gammaEBV4-96419, gammaEBV4-96422, gammaEBV4-96424, gammaEBV4-96425, gammaEBV4-96430, gammaEBV4-96436, gammaEBV4-96440, gammaEBV4-96442, gammaEBV4-96445, gammaEBV4-96446, gammaEBV4-96459, gammaEBV4-96462, gammaEBV4-96464, gammaEBV4-96466, gammaEBV4-96467, gammaEBV4-96468, gammaEBV4-96470, gammaEBV4-96472, gammaEBV4-96473, gammaEBV4-96481, gammaEBV4-96505, gammaEBV4-96521, gammaEBV4-96522, gammaEBV4-96542, gammaEBV4-96546, gammaEBV4-96554, gammaEBV4-96565, gammaEBV4-96566, gammaEBV4-96569, gammaEBV4-96575, gammaEBV4-96582, gammaEBV4-96586, gammaEBV4-96587, gammaEBV4-96588, gammaEBV4-96591, gammaEBV4-96592, gammaEBV4-96594, gammaEBV4-96595, gammaEBV4-96596, gammaEBV4-96598, gammaEBV4-96599, gammaEBV4-96601, gammaEBV4-96604, gammaEBV4-96607, gammaEBV4-96618, gammaEBV4-96622, gammaEBV4-96633, gammaEBV4-96634, gammaEBV4-96636, gammaEBV4-96637, gammaEBV4-96638, gammaEBV4-96640, gammaEBV4-96653, gammaEBV4-96675, gammaEBV4-96677, gammaEBV4-96685, gammaEBV4-96701, gammaEBV4-96703, gammaEBV4-96714, gammaEBV4-96719, gammaEBV4-96722, gammaEBV4-9679, gammaEBV4-96796, gammaEBV4-9712, gammaEBV4-97303, gammaEBV4-97310, gammaEBV4-97457, gammaEBV4-97485, gammaEBV4-97584, gammaEBV4-97632, gammaEBV4-98321, gammaEBV4-98485, gammaEBV4-98607, gammaEBV4-98649, gammaEBV4-98857, gammaEBV4-99257, gammaEBV4-99261, gammaEBV4-99275, gammaEBV4-99399, gammaEBV4-99402, gammaEBV4-99405, gammaEBV4-99422, gammaEBV4-99423, gammaEBV4-99440, gammaEBV4-99441, gammaEBV4-99535

Row 4: gene = BKRF4

gene: BKRF4

sample: CAEBV1, CAEBV11, CAEBV14, CAEBV16, CAEBV18, CAEBV19, CAEBV2, CAEBV20, CAEBV21, CAEBV5, CAEBV6, CAEBV8, CAEBV9, IM13, IM7

pos: gammaEBV4-100012, gammaEBV4-100015, gammaEBV4-100017, gammaEBV4-100032, gammaEBV4-100470, gammaEBV4-100929, gammaEBV4-101078, gammaEBV4-101090, gammaEBV4-101143, gammaEBV4-101244, gammaEBV4-97303, gammaEBV4-97310, gammaEBV4-97457, gammaEBV4-97485, gammaEBV4-97584, gammaEBV4-97632, gammaEBV4-98321, gammaEBV4-98485, gammaEBV4-98607, gammaEBV4-98649, gammaEBV4-98857, gammaEBV4-99257, gammaEBV4-99261, gammaEBV4-99275, gammaEBV4-99399, gammaEBV4-99402, gammaEBV4-99405, gammaEBV4-99422, gammaEBV4-99423, gammaEBV4-99440, gammaEBV4-99441, gammaEBV4-99535

Row 5: gene = BBRF2

gene: BBRF2

sample: CAEBV11, CAEBV14, CAEBV18, CAEBV19, CAEBV2, CAEBV20, CAEBV21, CAEBV5, CAEBV6, CAEBV9, IM12

pos: gammaEBV4-101896, gammaEBV4-101996, gammaEBV4-102132, gammaEBV4-102279, gammaEBV4-102548, gammaEBV4-102833, gammaEBV4-102972, gammaEBV4-103167, gammaEBV4-103346, gammaEBV4-103649, gammaEBV4-103838, gammaEBV4-104080, gammaEBV4-104243, gammaEBV4-104986, gammaEBV4-104994, gammaEBV4-105325, gammaEBV4-105501, gammaEBV4-105542, gammaEBV4-105598, gammaEBV4-106045, gammaEBV4-106231, gammaEBV4-106388, gammaEBV4-106430

Row 6: gene = BBLF2/BBLF3

gene: BBLF2/BBLF3

sample: CAEBV11, CAEBV14, CAEBV18, CAEBV19, CAEBV2, CAEBV20, CAEBV21, CAEBV5, CAEBV6, CAEBV7, CAEBV9, IM12

pos: gammaEBV4-102548, gammaEBV4-102833, gammaEBV4-102972, gammaEBV4-103167, gammaEBV4-103346, gammaEBV4-103649, gammaEBV4-103838, gammaEBV4-104080, gammaEBV4-104243, gammaEBV4-104986, gammaEBV4-104994, gammaEBV4-105325, gammaEBV4-105501, gammaEBV4-105542, gammaEBV4-105598, gammaEBV4-106045, gammaEBV4-106231, gammaEBV4-106388, gammaEBV4-106430, gammaEBV4-107019, gammaEBV4-107473, gammaEBV4-107738, gammaEBV4-108135, gammaEBV4-108164, gammaEBV4-108165, gammaEBV4-108219, gammaEBV4-108226, gammaEBV4-108228

Row 7: gene = EBNA-3A

gene: EBNA-3A

sample: CAEBV1, CAEBV10, CAEBV11, CAEBV12, CAEBV13, CAEBV14, CAEBV15, CAEBV16, CAEBV17, CAEBV18, CAEBV19, CAEBV2, CAEBV20, CAEBV21, CAEBV22, CAEBV23, CAEBV24, CAEBV3, CAEBV5, CAEBV6, CAEBV7, CAEBV8, CAEBV9, IM1, IM2, IM10, IM11, IM12, IM13, IM14, IM15, IM16, IM17, IM4, IM5, IM6, IM7, IM8, IM9

pos: gammaEBV4-10539, gammaEBV4-10920, gammaEBV4-11339, gammaEBV4-11955, gammaEBV4-13361, gammaEBV4-16098, gammaEBV4-19491, gammaEBV4-22574, gammaEBV4-26126, gammaEBV4-34536, gammaEBV4-35458, gammaEBV4-35769, gammaEBV4-36014, gammaEBV4-36209, gammaEBV4-36221, gammaEBV4-36265, gammaEBV4-36307, gammaEBV4-36315, gammaEBV4-36322, gammaEBV4-36355, gammaEBV4-36356, gammaEBV4-36360, gammaEBV4-36368, gammaEBV4-36389, gammaEBV4-36390, gammaEBV4-36391, gammaEBV4-36393, gammaEBV4-36394, gammaEBV4-36397, gammaEBV4-36399, gammaEBV4-36401, gammaEBV4-36402, gammaEBV4-36403, gammaEBV4-36405, gammaEBV4-36406, gammaEBV4-36408, gammaEBV4-36410, gammaEBV4-36411, gammaEBV4-36414, gammaEBV4-36415, gammaEBV4-36417, gammaEBV4-36420, gammaEBV4-36421, gammaEBV4-36423, gammaEBV4-36424, gammaEBV4-36425, gammaEBV4-36429, gammaEBV4-36432, gammaEBV4-36433, gammaEBV4-36435, gammaEBV4-36436, gammaEBV4-36437, gammaEBV4-36438, gammaEBV4-36439, gammaEBV4-36441, gammaEBV4-36442, gammaEBV4-36445, gammaEBV4-36446, gammaEBV4-36447, gammaEBV4-36448, gammaEBV4-36449, gammaEBV4-36450, gammaEBV4-36451, gammaEBV4-36453, gammaEBV4-36454, gammaEBV4-36455, gammaEBV4-36457, gammaEBV4-36458, gammaEBV4-36459, gammaEBV4-36460, gammaEBV4-36462, gammaEBV4-36463, gammaEBV4-36464, gammaEBV4-36465, gammaEBV4-36466, gammaEBV4-36468, gammaEBV4-36469, gammaEBV4-36470, gammaEBV4-36472, gammaEBV4-36475, gammaEBV4-36476, gammaEBV4-36477, gammaEBV4-36478, gammaEBV4-36479, gammaEBV4-36481, gammaEBV4-36482, gammaEBV4-36483, gammaEBV4-36486, gammaEBV4-36487, gammaEBV4-36489, gammaEBV4-36490, gammaEBV4-36491, gammaEBV4-36492, gammaEBV4-36495, gammaEBV4-36497, gammaEBV4-36498, gammaEBV4-36499, gammaEBV4-36501, gammaEBV4-36502, gammaEBV4-36507, gammaEBV4-36521, gammaEBV4-36528, gammaEBV4-36543, gammaEBV4-36558, gammaEBV4-36564, gammaEBV4-36710, gammaEBV4-36712, gammaEBV4-36745, gammaEBV4-36781, gammaEBV4-36801, gammaEBV4-36816, gammaEBV4-36834, gammaEBV4-36855, gammaEBV4-36856, gammaEBV4-36860, gammaEBV4-36865, gammaEBV4-36869, gammaEBV4-36882, gammaEBV4-36885, gammaEBV4-36897, gammaEBV4-36924, gammaEBV4-36940, gammaEBV4-36942, gammaEBV4-36946, gammaEBV4-36947, gammaEBV4-36961, gammaEBV4-36965, gammaEBV4-36993, gammaEBV4-37009, gammaEBV4-37019, gammaEBV4-37033, gammaEBV4-37044, gammaEBV4-37087, gammaEBV4-37113, gammaEBV4-37116, gammaEBV4-37130, gammaEBV4-37270, gammaEBV4-37490, gammaEBV4-37678, gammaEBV4-37924, gammaEBV4-37979, gammaEBV4-38989, gammaEBV4-39482, gammaEBV4-39873, gammaEBV4-39899, gammaEBV4-39903, gammaEBV4-39908, gammaEBV4-39915, gammaEBV4-39925, gammaEBV4-40194, gammaEBV4-40213, gammaEBV4-40219, gammaEBV4-40220, gammaEBV4-40224, gammaEBV4-40225, gammaEBV4-40263, gammaEBV4-40282, gammaEBV4-41499, gammaEBV4-41553, gammaEBV4-41648, gammaEBV4-41794, gammaEBV4-41821, gammaEBV4-43009, gammaEBV4-43140, gammaEBV4-43141, gammaEBV4-43142, gammaEBV4-43145, gammaEBV4-43146, gammaEBV4-43373, gammaEBV4-43465, gammaEBV4-43477, gammaEBV4-43509, gammaEBV4-43624, gammaEBV4-43684, gammaEBV4-43981, gammaEBV4-44109, gammaEBV4-44155, gammaEBV4-44242, gammaEBV4-44658, gammaEBV4-45678, gammaEBV4-45790, gammaEBV4-45805, gammaEBV4-45860, gammaEBV4-46042, gammaEBV4-46062, gammaEBV4-46257, gammaEBV4-46472, gammaEBV4-46706, gammaEBV4-46912, gammaEBV4-47220, gammaEBV4-47236, gammaEBV4-47240, gammaEBV4-47908, gammaEBV4-47955, gammaEBV4-48227, gammaEBV4-48251, gammaEBV4-48335, gammaEBV4-48519, gammaEBV4-48797, gammaEBV4-48983, gammaEBV4-49102, gammaEBV4-49719, gammaEBV4-50133, gammaEBV4-50242, gammaEBV4-51166, gammaEBV4-51500, gammaEBV4-51571, gammaEBV4-52091, gammaEBV4-52205, gammaEBV4-52211, gammaEBV4-52227, gammaEBV4-52259, gammaEBV4-52372, gammaEBV4-52814, gammaEBV4-52837, gammaEBV4-52952, gammaEBV4-53724, gammaEBV4-53902, gammaEBV4-54149, gammaEBV4-54243, gammaEBV4-54255, gammaEBV4-54491, gammaEBV4-54802, gammaEBV4-54921, gammaEBV4-55000, gammaEBV4-55730, gammaEBV4-55780, gammaEBV4-55793, gammaEBV4-55811, gammaEBV4-55869, gammaEBV4-55887, gammaEBV4-55914, gammaEBV4-56237, gammaEBV4-56566, gammaEBV4-56866, gammaEBV4-57429, gammaEBV4-57632, gammaEBV4-57708, gammaEBV4-57740, gammaEBV4-57806, gammaEBV4-57842, gammaEBV4-57869, gammaEBV4-57870, gammaEBV4-57879, gammaEBV4-57880, gammaEBV4-57896, gammaEBV4-57897, gammaEBV4-58047, gammaEBV4-58301, gammaEBV4-58407, gammaEBV4-58634, gammaEBV4-58692, gammaEBV4-58785, gammaEBV4-58867, gammaEBV4-59115, gammaEBV4-59448, gammaEBV4-59672, gammaEBV4-59799, gammaEBV4-59923, gammaEBV4-59992, gammaEBV4-60095, gammaEBV4-60374, gammaEBV4-60398, gammaEBV4-61987, gammaEBV4-62009, gammaEBV4-62182, gammaEBV4-62247, gammaEBV4-62391, gammaEBV4-62449, gammaEBV4-62499, gammaEBV4-62677, gammaEBV4-62690, gammaEBV4-62774, gammaEBV4-62778, gammaEBV4-62916, gammaEBV4-63356, gammaEBV4-63580, gammaEBV4-63692, gammaEBV4-64008, gammaEBV4-64199, gammaEBV4-64224, gammaEBV4-64324, gammaEBV4-64571, gammaEBV4-64795, gammaEBV4-64976, gammaEBV4-65127, gammaEBV4-65205, gammaEBV4-65433, gammaEBV4-65450, gammaEBV4-65685, gammaEBV4-65718, gammaEBV4-65785, gammaEBV4-65950, gammaEBV4-66061, gammaEBV4-66069, gammaEBV4-66363, gammaEBV4-66405, gammaEBV4-66612, gammaEBV4-66809, gammaEBV4-67279, gammaEBV4-67485, gammaEBV4-67505, gammaEBV4-67749, gammaEBV4-68057, gammaEBV4-68261, gammaEBV4-68279, gammaEBV4-68497, gammaEBV4-68583, gammaEBV4-68589, gammaEBV4-68612, gammaEBV4-68646, gammaEBV4-68657, gammaEBV4-68729, gammaEBV4-68748, gammaEBV4-69148, gammaEBV4-69192, gammaEBV4-69276, gammaEBV4-69611, gammaEBV4-69904, gammaEBV4-69950, gammaEBV4-70330, gammaEBV4-70538, gammaEBV4-71139, gammaEBV4-71229, gammaEBV4-71292, gammaEBV4-71344, gammaEBV4-71546, gammaEBV4-71618, gammaEBV4-71671, gammaEBV4-72053, gammaEBV4-72289, gammaEBV4-72560, gammaEBV4-72580, gammaEBV4-72690, gammaEBV4-72723, gammaEBV4-72979, gammaEBV4-73029, gammaEBV4-73305, gammaEBV4-73366, gammaEBV4-73378, gammaEBV4-73577, gammaEBV4-73713, gammaEBV4-73903, gammaEBV4-73989, gammaEBV4-74183, gammaEBV4-74250, gammaEBV4-74856, gammaEBV4-74882, gammaEBV4-75292, gammaEBV4-75357, gammaEBV4-75429, gammaEBV4-75555, gammaEBV4-75694, gammaEBV4-75699, gammaEBV4-76014, gammaEBV4-76449, gammaEBV4-76489, gammaEBV4-76525, gammaEBV4-76598, gammaEBV4-76943, gammaEBV4-77179, gammaEBV4-77304, gammaEBV4-77373, gammaEBV4-77401, gammaEBV4-77404, gammaEBV4-77419, gammaEBV4-77613, gammaEBV4-77647, gammaEBV4-77672, gammaEBV4-77826, gammaEBV4-78055, gammaEBV4-78268, gammaEBV4-78767, gammaEBV4-78969, gammaEBV4-79898, gammaEBV4-80010, gammaEBV4-80158, gammaEBV4-80180, gammaEBV4-80333, gammaEBV4-80380, gammaEBV4-80419, gammaEBV4-80444, gammaEBV4-81096, gammaEBV4-81378, gammaEBV4-81601, gammaEBV4-81671, gammaEBV4-81722, gammaEBV4-81794, gammaEBV4-81818, gammaEBV4-81950, gammaEBV4-81952, gammaEBV4-81957, gammaEBV4-81959, gammaEBV4-82027, gammaEBV4-82041, gammaEBV4-82056, gammaEBV4-82082, gammaEBV4-82089, gammaEBV4-82233, gammaEBV4-82234, gammaEBV4-82250, gammaEBV4-82253, gammaEBV4-82254, gammaEBV4-82259, gammaEBV4-82274, gammaEBV4-82347, gammaEBV4-82356, gammaEBV4-82358, gammaEBV4-82363, gammaEBV4-82366, gammaEBV4-82414, gammaEBV4-82418, gammaEBV4-82557, gammaEBV4-82685, gammaEBV4-82902, gammaEBV4-82911, gammaEBV4-83123, gammaEBV4-83632, gammaEBV4-84936, gammaEBV4-9391, gammaEBV4-9426, gammaEBV4-9427, gammaEBV4-9428, gammaEBV4-9430, gammaEBV4-9432, gammaEBV4-9433, gammaEBV4-9434, gammaEBV4-9437, gammaEBV4-9438, gammaEBV4-9439, gammaEBV4-9442, gammaEBV4-9444, gammaEBV4-9446, gammaEBV4-9447, gammaEBV4-9518, gammaEBV4-9679, gammaEBV4-9712

Row 8: gene = BCRF1

gene: BCRF1

sample: CAEBV10, CAEBV11, CAEBV12, CAEBV13, CAEBV17, CAEBV18, CAEBV19, CAEBV2, CAEBV20, CAEBV23, CAEBV5, CAEBV6, CAEBV8, IM12

pos: gammaEBV4-10539, gammaEBV4-10920, gammaEBV4-11339, gammaEBV4-11955, gammaEBV4-7691, gammaEBV4-7705, gammaEBV4-8048, gammaEBV4-8244, gammaEBV4-8500, gammaEBV4-8555, gammaEBV4-8901, gammaEBV4-9007, gammaEBV4-9391, gammaEBV4-9426, gammaEBV4-9427, gammaEBV4-9428, gammaEBV4-9430, gammaEBV4-9432, gammaEBV4-9433, gammaEBV4-9434, gammaEBV4-9437, gammaEBV4-9438, gammaEBV4-9439, gammaEBV4-9442, gammaEBV4-9444, gammaEBV4-9446, gammaEBV4-9447, gammaEBV4-9518, gammaEBV4-9679, gammaEBV4-9712

Row 9: gene = BGLF5

gene: BGLF5

sample: CAEBV11, CAEBV2, CAEBV20, CAEBV21, CAEBV6, CAEBV7, CAEBV9, IM10, IM11, IM12

pos: gammaEBV4-107019, gammaEBV4-107473, gammaEBV4-107738, gammaEBV4-108135, gammaEBV4-108164, gammaEBV4-108165, gammaEBV4-108219, gammaEBV4-108226, gammaEBV4-108228, gammaEBV4-109037, gammaEBV4-109074, gammaEBV4-110375, gammaEBV4-110393, gammaEBV4-110468, gammaEBV4-110538, gammaEBV4-110756, gammaEBV4-110937, gammaEBV4-111238

Row 10: gene = BGLF4

gene: BGLF4

sample: CAEBV11, CAEBV14, CAEBV2, CAEBV20, CAEBV5, CAEBV6, CAEBV7, CAEBV9, IM10, IM11, IM12

pos: gammaEBV4-108135, gammaEBV4-108164, gammaEBV4-108165, gammaEBV4-108219, gammaEBV4-108226, gammaEBV4-108228, gammaEBV4-109037, gammaEBV4-109074, gammaEBV4-110375, gammaEBV4-110393, gammaEBV4-110468, gammaEBV4-110538, gammaEBV4-110756, gammaEBV4-110937, gammaEBV4-111238, gammaEBV4-112229, gammaEBV4-112312, gammaEBV4-112741, gammaEBV4-113146

Row 11: gene = BGLF3.5

gene: BGLF3.5

sample: CAEBV11, CAEBV14, CAEBV2, CAEBV20, CAEBV5, CAEBV6, CAEBV7, CAEBV9, IM11, IM12

pos: gammaEBV4-110375, gammaEBV4-110393, gammaEBV4-110468, gammaEBV4-110538, gammaEBV4-110756, gammaEBV4-110937, gammaEBV4-111238, gammaEBV4-112229, gammaEBV4-112312, gammaEBV4-112741, gammaEBV4-113146, gammaEBV4-113587, gammaEBV4-113631

Row 12: gene = BGLF3

gene: BGLF3

sample: CAEBV11, CAEBV14, CAEBV18, CAEBV19, CAEBV2, CAEBV20, CAEBV5, CAEBV6, CAEBV7, CAEBV9, IM11, IM12

pos: gammaEBV4-110375, gammaEBV4-110393, gammaEBV4-110468, gammaEBV4-110538, gammaEBV4-110756, gammaEBV4-110937, gammaEBV4-111238, gammaEBV4-112229, gammaEBV4-112312, gammaEBV4-112741, gammaEBV4-113146, gammaEBV4-113587, gammaEBV4-113631, gammaEBV4-113886, gammaEBV4-113897, gammaEBV4-114033, gammaEBV4-114134, gammaEBV4-114178, gammaEBV4-114596

Row 13: gene = BGRF1/BDRF1

gene: BGRF1/BDRF1

sample: CAEBV11, CAEBV14, CAEBV18, CAEBV19, CAEBV2, CAEBV20, CAEBV5, CAEBV6, CAEBV7, CAEBV9, IM11, IM7

pos: gammaEBV4-110756, gammaEBV4-110937, gammaEBV4-111238, gammaEBV4-112229, gammaEBV4-112312, gammaEBV4-112741, gammaEBV4-113146, gammaEBV4-113587, gammaEBV4-113631, gammaEBV4-113886, gammaEBV4-113897, gammaEBV4-114033, gammaEBV4-114134, gammaEBV4-114178, gammaEBV4-114596, gammaEBV4-114785, gammaEBV4-115055, gammaEBV4-115222, gammaEBV4-115343, gammaEBV4-115861, gammaEBV4-115891, gammaEBV4-115990, gammaEBV4-116738, gammaEBV4-117372, gammaEBV4-117462, gammaEBV4-117583, gammaEBV4-117880, gammaEBV4-117936, gammaEBV4-117941, gammaEBV4-118378, gammaEBV4-118380, gammaEBV4-118437, gammaEBV4-118443, gammaEBV4-118497, gammaEBV4-118554, gammaEBV4-118559, gammaEBV4-119450, gammaEBV4-119719, gammaEBV4-119738, gammaEBV4-119764, gammaEBV4-119766, gammaEBV4-120040

Row 14: gene = BcLF1

gene: BcLF1

sample: CAEBV1, CAEBV10, CAEBV11, CAEBV12, CAEBV13, CAEBV14, CAEBV17, CAEBV18, CAEBV19, CAEBV2, CAEBV20, CAEBV21, CAEBV22, CAEBV23, CAEBV3, CAEBV5, CAEBV6, CAEBV7, CAEBV8, CAEBV9, IM11, IM12, IM13, IM15, IM5, IM6, IM7, IM9

pos: gammaEBV4-119450, gammaEBV4-119719, gammaEBV4-119738, gammaEBV4-119764, gammaEBV4-119766, gammaEBV4-120040, gammaEBV4-120837, gammaEBV4-121112, gammaEBV4-121131, gammaEBV4-122449, gammaEBV4-122641, gammaEBV4-123006, gammaEBV4-123073, gammaEBV4-123123, gammaEBV4-123468, gammaEBV4-123550, gammaEBV4-123573, gammaEBV4-123594, gammaEBV4-123722, gammaEBV4-123726, gammaEBV4-123773, gammaEBV4-123786, gammaEBV4-123790, gammaEBV4-123855, gammaEBV4-124007, gammaEBV4-124331, gammaEBV4-124333, gammaEBV4-124335, gammaEBV4-124336, gammaEBV4-124337, gammaEBV4-124338, gammaEBV4-124339, gammaEBV4-124340, gammaEBV4-124342, gammaEBV4-124343, gammaEBV4-124344, gammaEBV4-124345, gammaEBV4-124346, gammaEBV4-124347, gammaEBV4-124348, gammaEBV4-124349, gammaEBV4-124350, gammaEBV4-124351, gammaEBV4-124352, gammaEBV4-124356, gammaEBV4-124357, gammaEBV4-124373, gammaEBV4-124681, gammaEBV4-124988, gammaEBV4-125072, gammaEBV4-125106, gammaEBV4-125211, gammaEBV4-125323, gammaEBV4-125330, gammaEBV4-125786, gammaEBV4-125876, gammaEBV4-125881, gammaEBV4-126135, gammaEBV4-126219, gammaEBV4-126402

Row 15: gene = BcRF1

gene: BcRF1

sample: CAEBV1, CAEBV10, CAEBV11, CAEBV12, CAEBV13, CAEBV14, CAEBV17, CAEBV18, CAEBV19, CAEBV2, CAEBV20, CAEBV21, CAEBV22, CAEBV23, CAEBV3, CAEBV5, CAEBV6, CAEBV7, CAEBV8, CAEBV9, IM11, IM12, IM13, IM15, IM5, IM6, IM7, IM9

pos: gammaEBV4-123468, gammaEBV4-123550, gammaEBV4-123573, gammaEBV4-123594, gammaEBV4-123722, gammaEBV4-123726, gammaEBV4-123773, gammaEBV4-123786, gammaEBV4-123790, gammaEBV4-123855, gammaEBV4-124007, gammaEBV4-124331, gammaEBV4-124333, gammaEBV4-124335, gammaEBV4-124336, gammaEBV4-124337, gammaEBV4-124338, gammaEBV4-124339, gammaEBV4-124340, gammaEBV4-124342, gammaEBV4-124343, gammaEBV4-124344, gammaEBV4-124345, gammaEBV4-124346, gammaEBV4-124347, gammaEBV4-124348, gammaEBV4-124349, gammaEBV4-124350, gammaEBV4-124351, gammaEBV4-124352, gammaEBV4-124356, gammaEBV4-124357, gammaEBV4-124373, gammaEBV4-124681, gammaEBV4-124988, gammaEBV4-125072, gammaEBV4-125106, gammaEBV4-125211, gammaEBV4-125323, gammaEBV4-125330, gammaEBV4-125786, gammaEBV4-125876, gammaEBV4-125881, gammaEBV4-126135,

gammaEBV4-126219, gammaEBV4-126402, gammaEBV4-127266, gammaEBV4-127347, gammaEBV4-127645, gammaEBV4-127663, gammaEBV4-127669, gammaEBV4-127827, gammaEBV4-128010, gammaEBV4-128770, gammaEBV4-128837, gammaEBV4-129213, gammaEBV4-129395

Row 16: gene = BTRF1

gene: BTRF1

sample: CAEBV1, CAEBV11, CAEBV13, CAEBV14, CAEBV18, CAEBV19, CAEBV2, CAEBV20, CAEBV5, CAEBV6, CAEBV8, CAEBV9, IM11

pos: gammaEBV4-125786, gammaEBV4-125876, gammaEBV4-125881, gammaEBV4-126135, gammaEBV4-126219, gammaEBV4-126402, gammaEBV4-127266, gammaEBV4-127347, gammaEBV4-127645, gammaEBV4-127663, gammaEBV4-127669, gammaEBV4-127827, gammaEBV4-128010, gammaEBV4-128770, gammaEBV4-128837, gammaEBV4-129213, gammaEBV4-129395, gammaEBV4-130293, gammaEBV4-130302, gammaEBV4-130543, gammaEBV4-130568, gammaEBV4-130569

Row 17: gene = BXLF1

gene: BXLF1

sample: CAEBV1, CAEBV10, CAEBV11, CAEBV14, CAEBV15, CAEBV18, CAEBV19, CAEBV2, CAEBV20, CAEBV5, CAEBV6, CAEBV8, CAEBV9

pos: gammaEBV4-128770, gammaEBV4-128837, gammaEBV4-129213, gammaEBV4-129395, gammaEBV4-130293, gammaEBV4-130302, gammaEBV4-130543, gammaEBV4-130568, gammaEBV4-130569, gammaEBV4-130696, gammaEBV4-130723, gammaEBV4-130930, gammaEBV4-131300, gammaEBV4-131367, gammaEBV4-131675, gammaEBV4-132155, gammaEBV4-132194, gammaEBV4-132223, gammaEBV4-132224, gammaEBV4-132232, gammaEBV4-132437, gammaEBV4-133088, gammaEBV4-133974, gammaEBV4-134311, gammaEBV4-134348, gammaEBV4-134460, gammaEBV4-134537

Row 18: gene = BXRF1

gene: BXRF1

sample: CAEBV1, CAEBV10, CAEBV15, CAEBV18, CAEBV19, CAEBV2, CAEBV20, CAEBV5, CAEBV6, CAEBV9

pos: gammaEBV4-130569, gammaEBV4-130696, gammaEBV4-130723, gammaEBV4-130930, gammaEBV4-131300, gammaEBV4-131367, gammaEBV4-131675, gammaEBV4-132155, gammaEBV4-132194, gammaEBV4-132223, gammaEBV4-132224, gammaEBV4-132232, gammaEBV4-132437, gammaEBV4-133088, gammaEBV4-133974, gammaEBV4-134311, gammaEBV4-134348, gammaEBV4-134460, gammaEBV4-134537, gammaEBV4-134765, gammaEBV4-134834

Row 19: gene = BVLF1

gene: BVLF1

sample: CAEBV11, CAEBV18, CAEBV19, CAEBV2, CAEBV20, CAEBV4, CAEBV5, CAEBV6, CAEBV9

pos: gammaEBV4-133088, gammaEBV4-133974, gammaEBV4-134311, gammaEBV4-134348, gammaEBV4-134460, gammaEBV4-134537, gammaEBV4-134765, gammaEBV4-134834, gammaEBV4-135676, gammaEBV4-135687, gammaEBV4-135692, gammaEBV4-135852, gammaEBV4-135865, gammaEBV4-136075, gammaEBV4-136560, gammaEBV4-136578, gammaEBV4-136653, gammaEBV4-136754, gammaEBV4-136896, gammaEBV4-136927, gammaEBV4-137017, gammaEBV4-137032, gammaEBV4-137120, gammaEBV4-137135, gammaEBV4-137142, gammaEBV4-137148, gammaEBV4-137151, gammaEBV4-137170, gammaEBV4-137227, gammaEBV4-137268

Row 20: gene = EBNA-LP

gene: EBNA-LP

sample: CAEBV1, CAEBV10, CAEBV11, CAEBV12, CAEBV13, CAEBV14, CAEBV15, CAEBV16, CAEBV17, CAEBV18, CAEBV19, CAEBV2, CAEBV20, CAEBV21, CAEBV22, CAEBV23, CAEBV24, CAEBV3, CAEBV5, CAEBV6, CAEBV7, CAEBV8, CAEBV9, IM1, IM2, IM10, IM11, IM12, IM13, IM14, IM15, IM16, IM17, IM4, IM5, IM6, IM7, IM8, IM9

pos: gammaEBV4-13361, gammaEBV4-16098, gammaEBV4-19491, gammaEBV4-22574, gammaEBV4-26126, gammaEBV4-34536, gammaEBV4-35458, gammaEBV4-35769, gammaEBV4-36014, gammaEBV4-36209, gammaEBV4-36221, gammaEBV4-36265, gammaEBV4-36307, gammaEBV4-36315, gammaEBV4-36322, gammaEBV4-36355, gammaEBV4-36356, gammaEBV4-36360, gammaEBV4-36368, gammaEBV4-36389, gammaEBV4-36390, gammaEBV4-36391, gammaEBV4-36393, gammaEBV4-36394, gammaEBV4-36397, gammaEBV4-36399, gammaEBV4-36401, gammaEBV4-36402, gammaEBV4-36403, gammaEBV4-36405, gammaEBV4-36406, gammaEBV4-36408, gammaEBV4-36410, gammaEBV4-36411, gammaEBV4-36414, gammaEBV4-36415, gammaEBV4-36417, gammaEBV4-36420, gammaEBV4-36421, gammaEBV4-36423, gammaEBV4-36424, gammaEBV4-36425, gammaEBV4-36429, gammaEBV4-36432, gammaEBV4-36433, gammaEBV4-36435, gammaEBV4-36436, gammaEBV4-36437, gammaEBV4-36438, gammaEBV4-36439, gammaEBV4-36441, gammaEBV4-36442, gammaEBV4-36445, gammaEBV4-36446, gammaEBV4-36447, gammaEBV4-36448, gammaEBV4-36449, gammaEBV4-36450, gammaEBV4-36451, gammaEBV4-36453, gammaEBV4-36454, gammaEBV4-36455, gammaEBV4-36457, gammaEBV4-36458, gammaEBV4-36459, gammaEBV4-36460, gammaEBV4-36462, gammaEBV4-36463, gammaEBV4-36464, gammaEBV4-36465, gammaEBV4-36466, gammaEBV4-36468, gammaEBV4-36469, gammaEBV4-36470, gammaEBV4-36472, gammaEBV4-36475, gammaEBV4-36476, gammaEBV4-36477, gammaEBV4-36478, gammaEBV4-36479, gammaEBV4-36481, gammaEBV4-36482, gammaEBV4-36483, gammaEBV4-36486, gammaEBV4-36487, gammaEBV4-36489, gammaEBV4-36490, gammaEBV4-36491, gammaEBV4-36492, gammaEBV4-36495, gammaEBV4-36497, gammaEBV4-36498, gammaEBV4-36499, gammaEBV4-36501, gammaEBV4-36502, gammaEBV4-36507, gammaEBV4-36521, gammaEBV4-36528, gammaEBV4-36543, gammaEBV4-36558, gammaEBV4-36564, gammaEBV4-36710, gammaEBV4-36712, gammaEBV4-36745, gammaEBV4-36781, gammaEBV4-36801, gammaEBV4-36816, gammaEBV4-36834, gammaEBV4-36855, gammaEBV4-36856, gammaEBV4-36860, gammaEBV4-36865, gammaEBV4-36869, gammaEBV4-36882, gammaEBV4-36885, gammaEBV4-36897, gammaEBV4-36924, gammaEBV4-36940, gammaEBV4-36942, gammaEBV4-36946, gammaEBV4-36947, gammaEBV4-36961, gammaEBV4-36965, gammaEBV4-36993, gammaEBV4-37009, gammaEBV4-37019, gammaEBV4-37033, gammaEBV4-37044, gammaEBV4-37087, gammaEBV4-37113, gammaEBV4-37116, gammaEBV4-37130, gammaEBV4-37270, gammaEBV4-37490, gammaEBV4-37678, gammaEBV4-37924, gammaEBV4-37979, gammaEBV4-38989, gammaEBV4-39482

Row 21: gene = BVRF2

gene: BVRF2

sample: CAEBV11, CAEBV15, CAEBV18, CAEBV19, CAEBV2, CAEBV20, CAEBV4, CAEBV5, CAEBV6, CAEBV8, CAEBV9, IM12

pos: gammaEBV4-133974, gammaEBV4-134311, gammaEBV4-134348, gammaEBV4-134460, gammaEBV4-134537, gammaEBV4-134765, gammaEBV4-134834, gammaEBV4-135676, gammaEBV4-135687, gammaEBV4-135692, gammaEBV4-135852, gammaEBV4-135865, gammaEBV4-136075, gammaEBV4-136560, gammaEBV4-136578, gammaEBV4-136653, gammaEBV4-136754, gammaEBV4-136896, gammaEBV4-136927, gammaEBV4-137017, gammaEBV4-137032, gammaEBV4-137120, gammaEBV4-137135, gammaEBV4-137142, gammaEBV4-137148, gammaEBV4-137151, gammaEBV4-137170, gammaEBV4-137227, gammaEBV4-137268, gammaEBV4-138016, gammaEBV4-138281, gammaEBV4-138618, gammaEBV4-138625, gammaEBV4-138872, gammaEBV4-138960, gammaEBV4-139071, gammaEBV4-139128, gammaEBV4-139154, gammaEBV4-139166, gammaEBV4-139196, gammaEBV4-139198, gammaEBV4-139202, gammaEBV4-139204, gammaEBV4-139207, gammaEBV4-139209, gammaEBV4-139214, gammaEBV4-139216, gammaEBV4-139438

Row 22: gene = BdRF1

gene: BdRF1

sample: CAEBV11, CAEBV15, CAEBV18, CAEBV19, CAEBV2, CAEBV20, CAEBV4, CAEBV5, CAEBV6, CAEBV8, CAEBV9, IM12

pos: gammaEBV4-134460, gammaEBV4-134537, gammaEBV4-134765, gammaEBV4-134834, gammaEBV4-135676, gammaEBV4-135687, gammaEBV4-135692, gammaEBV4-135852, gammaEBV4-135865, gammaEBV4-136075, gammaEBV4-136560, gammaEBV4-136578, gammaEBV4-136653, gammaEBV4-136754, gammaEBV4-136896, gammaEBV4-136927, gammaEBV4-137017, gammaEBV4-137032, gammaEBV4-137120, gammaEBV4-137135, gammaEBV4-137142, gammaEBV4-137148, gammaEBV4-137151, gammaEBV4-137170, gammaEBV4-137227, gammaEBV4-137268, gammaEBV4-138016, gammaEBV4-138281, gammaEBV4-138618, gammaEBV4-138625, gammaEBV4-138872, gammaEBV4-138960, gammaEBV4-139071, gammaEBV4-139128, gammaEBV4-139154, gammaEBV4-139166, gammaEBV4-139196, gammaEBV4-139198, gammaEBV4-139202, gammaEBV4-139204, gammaEBV4-139207, gammaEBV4-139209, gammaEBV4-139214, gammaEBV4-139216, gammaEBV4-139438

Row 23: gene = LF2

gene: LF2

sample: CAEBV11, CAEBV19, CAEBV20, CAEBV22, CAEBV5, CAEBV6, CAEBV8, CAEBV9, IM16

pos: gammaEBV4-147843, gammaEBV4-147959, gammaEBV4-148630, gammaEBV4-148650, gammaEBV4-148658, gammaEBV4-148892, gammaEBV4-148962, gammaEBV4-148975, gammaEBV4-149020, gammaEBV4-149096, gammaEBV4-149397, gammaEBV4-149494, gammaEBV4-149760, gammaEBV4-150546, gammaEBV4-150551, gammaEBV4-150702, gammaEBV4-150749, gammaEBV4-151271, gammaEBV4-151272

Row 24: gene = LF1

gene: LF1

sample: CAEBV1, CAEBV13, CAEBV17, CAEBV19, CAEBV20, CAEBV22, CAEBV5, CAEBV6, CAEBV8, CAEBV9, IM16

pos: gammaEBV4-148630, gammaEBV4-148650, gammaEBV4-148658, gammaEBV4-148892, gammaEBV4-148962, gammaEBV4-148975, gammaEBV4-149020, gammaEBV4-149096, gammaEBV4-149397, gammaEBV4-149494, gammaEBV4-149760, gammaEBV4-150546, gammaEBV4-150551, gammaEBV4-150702, gammaEBV4-150749, gammaEBV4-151271, gammaEBV4-151272, gammaEBV4-152545, gammaEBV4-152557, gammaEBV4-152613, gammaEBV4-153004, gammaEBV4-153077, gammaEBV4-153117, gammaEBV4-153180, gammaEBV4-153617, gammaEBV4-153639

Row 25: gene = BILF1

gene: BILF1

sample: CAEBV1, CAEBV11, CAEBV13, CAEBV17, CAEBV18, CAEBV19, CAEBV22, CAEBV5, CAEBV6, CAEBV8, CAEBV9, IM6

pos: gammaEBV4-149760, gammaEBV4-150546, gammaEBV4-150551, gammaEBV4-150702, gammaEBV4-150749, gammaEBV4-151271, gammaEBV4-151272, gammaEBV4-152545, gammaEBV4-152557, gammaEBV4-152613, gammaEBV4-153004, gammaEBV4-153077, gammaEBV4-153117, gammaEBV4-153180, gammaEBV4-153617, gammaEBV4-153639, gammaEBV4-153725, gammaEBV4-153763, gammaEBV4-153860, gammaEBV4-153864, gammaEBV4-153952, gammaEBV4-154055, gammaEBV4-154299, gammaEBV4-154429, gammaEBV4-154453

Row 26: gene = BALF5

gene: BALF5

sample: CAEBV1, CAEBV11, CAEBV13, CAEBV14, CAEBV17, CAEBV18, CAEBV19, CAEBV2, CAEBV20, CAEBV3, CAEBV5, CAEBV6, CAEBV8, CAEBV9, IM6

pos: gammaEBV4-151271, gammaEBV4-151272, gammaEBV4-152545, gammaEBV4-152557, gammaEBV4-152613, gammaEBV4-153004, gammaEBV4-153077, gammaEBV4-153117, gammaEBV4-153180, gammaEBV4-153617, gammaEBV4-153639, gammaEBV4-153725, gammaEBV4-153763, gammaEBV4-153860, gammaEBV4-153864, gammaEBV4-153952, gammaEBV4-154055, gammaEBV4-154299, gammaEBV4-154429, gammaEBV4-154453, gammaEBV4-154654, gammaEBV4-154720, gammaEBV4-154982, gammaEBV4-155166, gammaEBV4-155263, gammaEBV4-155584, gammaEBV4-155832, gammaEBV4-156147, gammaEBV4-156363, gammaEBV4-156375, gammaEBV4-156562, gammaEBV4-156606, gammaEBV4-157058, gammaEBV4-157364, gammaEBV4-157434, gammaEBV4-157853

Row 27: gene = A73

gene: A73

sample: CAEBV1, CAEBV11, CAEBV13, CAEBV14, CAEBV18, CAEBV19, CAEBV2, CAEBV20, CAEBV21, CAEBV3, CAEBV5, CAEBV6, CAEBV7, CAEBV8, CAEBV9, IM12, IM6

pos: gammaEBV4-153617, gammaEBV4-153639, gammaEBV4-153725, gammaEBV4-153763, gammaEBV4-153860, gammaEBV4-153864, gammaEBV4-153952, gammaEBV4-154055, gammaEBV4-154299, gammaEBV4-154429, gammaEBV4-154453, gammaEBV4-154654, gammaEBV4-154720, gammaEBV4-154982, gammaEBV4-155166, gammaEBV4-155263, gammaEBV4-155584, gammaEBV4-155832, gammaEBV4-156147, gammaEBV4-156363, gammaEBV4-156375, gammaEBV4-156562, gammaEBV4-156606, gammaEBV4-157058, gammaEBV4-157364, gammaEBV4-157434, gammaEBV4-157853, gammaEBV4-159158, gammaEBV4-159376, gammaEBV4-160788, gammaEBV4-161004, gammaEBV4-161324, gammaEBV4-161348, gammaEBV4-161445, gammaEBV4-161533, gammaEBV4-162029, gammaEBV4-162140, gammaEBV4-162204, gammaEBV4-162480

Row 28: gene = BALF2

gene: BALF2

sample: CAEBV1, CAEBV19, CAEBV2, CAEBV20, CAEBV21, CAEBV5, CAEBV6, CAEBV7, CAEBV8, CAEBV9, IM10, IM12, IM5

pos: gammaEBV4-159158, gammaEBV4-159376, gammaEBV4-160788, gammaEBV4-161004, gammaEBV4-161324, gammaEBV4-161348, gammaEBV4-161445, gammaEBV4-161533, gammaEBV4-162029, gammaEBV4-162140, gammaEBV4-162204, gammaEBV4-162480, gammaEBV4-162703, gammaEBV4-162758, gammaEBV4-163569, gammaEBV4-163668, gammaEBV4-163804, gammaEBV4-163829, gammaEBV4-164100, gammaEBV4-164161, gammaEBV4-164382, gammaEBV4-164387, gammaEBV4-164551, gammaEBV4-164586, gammaEBV4-164676, gammaEBV4-164692, gammaEBV4-164787, gammaEBV4-164982, gammaEBV4-165051, gammaEBV4-165155, gammaEBV4-165639, gammaEBV4-165807, gammaEBV4-166017, gammaEBV4-166116, gammaEBV4-166237

Row 29: gene = BALF1

gene: BALF1

sample: CAEBV1, CAEBV10, CAEBV11, CAEBV19, CAEBV2, CAEBV20, CAEBV21, CAEBV5, CAEBV6, CAEBV8, CAEBV9, IM10, IM12, IM13, IM5

pos: gammaEBV4-162480, gammaEBV4-162703, gammaEBV4-162758, gammaEBV4-163569, gammaEBV4-163668, gammaEBV4-163804, gammaEBV4-163829, gammaEBV4-164100, gammaEBV4-164161, gammaEBV4-164382, gammaEBV4-164387, gammaEBV4-164551, gammaEBV4-164586, gammaEBV4-164676, gammaEBV4-164692, gammaEBV4-164787, gammaEBV4-164982, gammaEBV4-165051, gammaEBV4-165155, gammaEBV4-165639, gammaEBV4-165807, gammaEBV4-166017,

gammaEBV4-166116, gammaEBV4-166237, gammaEBV4-166370, gammaEBV4-166656, gammaEBV4-166695, gammaEBV4-166813

Row 30: gene = BARF1

gene: BARF1

sample: CAEBV1, CAEBV10, CAEBV11, CAEBV13, CAEBV19, CAEBV2, CAEBV20, CAEBV21, CAEBV5, CAEBV6, CAEBV8, CAEBV9, IM10, IM12, IM13, IM5

pos: gammaEBV4-163569, gammaEBV4-163668, gammaEBV4-163804, gammaEBV4-163829, gammaEBV4-164100, gammaEBV4-164161, gammaEBV4-164382, gammaEBV4-164387, gammaEBV4-164551, gammaEBV4-164586, gammaEBV4-164676, gammaEBV4-164692, gammaEBV4-164787, gammaEBV4-164982, gammaEBV4-165051, gammaEBV4-165155, gammaEBV4-165639, gammaEBV4-165807, gammaEBV4-166017, gammaEBV4-166116, gammaEBV4-166237, gammaEBV4-166370, gammaEBV4-166656, gammaEBV4-166695, gammaEBV4-166813, gammaEBV4-167362, gammaEBV4-167370, gammaEBV4-167399, gammaEBV4-167404, gammaEBV4-167437, gammaEBV4-167488, gammaEBV4-167506

Row 31: gene = LMP-2A

gene: LMP-2A

sample: CAEBV1, CAEBV10, CAEBV11, CAEBV13, CAEBV16-2, CAEBV19, CAEBV2, CAEBV20, CAEBV21, CAEBV5, CAEBV6, CAEBV8, CAEBV9, IM10, IM12, IM13, IM5

pos: gammaEBV4-164100, gammaEBV4-164161, gammaEBV4-164382, gammaEBV4-164387, gammaEBV4-164551, gammaEBV4-164586, gammaEBV4-164676, gammaEBV4-164692, gammaEBV4-164787, gammaEBV4-164982, gammaEBV4-165051, gammaEBV4-165155, gammaEBV4-165639, gammaEBV4-165807, gammaEBV4-166017, gammaEBV4-166116, gammaEBV4-166237, gammaEBV4-166370, gammaEBV4-166656, gammaEBV4-166695, gammaEBV4-166813, gammaEBV4-167362, gammaEBV4-167370, gammaEBV4-167399, gammaEBV4-167404, gammaEBV4-167437, gammaEBV4-167488, gammaEBV4-167506, gammaEBV4-168640, gammaEBV4-169257, gammaEBV4-169350, gammaEBV4-169430, gammaEBV4-169442, gammaEBV4-169460, gammaEBV4-169485, gammaEBV4-169565, gammaEBV4-171084, gammaEBV4-171244, gammaEBV4-171498, gammaEBV4-171768, gammaEBV4-171773, gammaEBV4-171783

Row 32: gene = BNRF1

gene: BNRF1

sample: CAEBV11, CAEBV13, CAEBV17, CAEBV18, CAEBV19, CAEBV2, CAEBV20, CAEBV21, CAEBV3, CAEBV5, CAEBV8, CAEBV9, IM2, IM7

pos: gammaEBV4-2403, gammaEBV4-2768, gammaEBV4-2769, gammaEBV4-2958, gammaEBV4-3452, gammaEBV4-3640, gammaEBV4-3720, gammaEBV4-3754, gammaEBV4-3758, gammaEBV4-3775, gammaEBV4-4082, gammaEBV4-4944, gammaEBV4-5080, gammaEBV4-5182, gammaEBV4-5406, gammaEBV4-5665, gammaEBV4-5739, gammaEBV4-5751, gammaEBV4-5758, gammaEBV4-5762, gammaEBV4-5793, gammaEBV4-5796, gammaEBV4-5798, gammaEBV4-5799, gammaEBV4-5802, gammaEBV4-5803, gammaEBV4-5804, gammaEBV4-5805, gammaEBV4-5807, gammaEBV4-5808, gammaEBV4-5809, gammaEBV4-5810, gammaEBV4-7019, gammaEBV4-7035, gammaEBV4-7210, gammaEBV4-7253, gammaEBV4-7691

Row 33: gene = BFLF1

gene: BFLF1

sample: CAEBV1, CAEBV11, CAEBV13, CAEBV19, CAEBV2, CAEBV21, CAEBV3, CAEBV5, CAEBV6, CAEBV7, IM7, IM8

pos: gammaEBV4-43009, gammaEBV4-43140, gammaEBV4-43141, gammaEBV4-43142, gammaEBV4-43145, gammaEBV4-43146, gammaEBV4-43373, gammaEBV4-43465, gammaEBV4-43477, gammaEBV4-43509, gammaEBV4-43624, gammaEBV4-43684, gammaEBV4-43981, gammaEBV4-44109, gammaEBV4-44155, gammaEBV4-44242, gammaEBV4-44658, gammaEBV4-45678, gammaEBV4-45790, gammaEBV4-45805, gammaEBV4-45860, gammaEBV4-46042, gammaEBV4-46062, gammaEBV4-46257, gammaEBV4-46472, gammaEBV4-46706, gammaEBV4-46912, gammaEBV4-47220, gammaEBV4-47236, gammaEBV4-47240, gammaEBV4-47908, gammaEBV4-47955, gammaEBV4-48227

Row 34: gene = BFRF1A

gene: BFRF1A

sample: CAEBV10, CAEBV11, CAEBV13, CAEBV19, CAEBV2, CAEBV21, CAEBV5, CAEBV6, CAEBV7, IM7

pos: gammaEBV4-44242, gammaEBV4-44658, gammaEBV4-45678, gammaEBV4-45790, gammaEBV4-45805, gammaEBV4-45860, gammaEBV4-46042, gammaEBV4-46062, gammaEBV4-46257, gammaEBV4-46472, gammaEBV4-46706, gammaEBV4-46912, gammaEBV4-47220, gammaEBV4-47236, gammaEBV4-47240, gammaEBV4-47908, gammaEBV4-47955, gammaEBV4-48227, gammaEBV4-48251, gammaEBV4-48335, gammaEBV4-48519

Row 35: gene = BFRF1

gene: BFRF1

sample: CAEBV10, CAEBV11, CAEBV13, CAEBV14, CAEBV19, CAEBV2, CAEBV20, CAEBV21, CAEBV5, CAEBV6, CAEBV7, IM7

pos: gammaEBV4-44658, gammaEBV4-45678, gammaEBV4-45790, gammaEBV4-45805, gammaEBV4-45860, gammaEBV4-46042, gammaEBV4-46062, gammaEBV4-46257, gammaEBV4-46472, gammaEBV4-46706, gammaEBV4-46912, gammaEBV4-47220, gammaEBV4-47236, gammaEBV4-47240, gammaEBV4-47908, gammaEBV4-47955, gammaEBV4-48227, gammaEBV4-48251, gammaEBV4-48335, gammaEBV4-48519, gammaEBV4-48797, gammaEBV4-48983, gammaEBV4-49102

Row 36: gene = BFRF2

gene: BFRF2

sample: CAEBV10, CAEBV11, CAEBV13, CAEBV14, CAEBV19, CAEBV2, CAEBV20, CAEBV21, CAEBV5, CAEBV6, CAEBV7, IM12, IM7

pos: gammaEBV4-45678, gammaEBV4-45790, gammaEBV4-45805, gammaEBV4-45860, gammaEBV4-46042, gammaEBV4-46062, gammaEBV4-46257, gammaEBV4-46472, gammaEBV4-46706, gammaEBV4-46912, gammaEBV4-47220, gammaEBV4-47236, gammaEBV4-47240, gammaEBV4-47908, gammaEBV4-47955, gammaEBV4-48227, gammaEBV4-48251, gammaEBV4-48335, gammaEBV4-48519, gammaEBV4-48797, gammaEBV4-48983, gammaEBV4-49102, gammaEBV4-49719, gammaEBV4-50133, gammaEBV4-50242, gammaEBV4-51166

Row 37: gene = BFRF3

gene: BFRF3

sample: CAEBV10, CAEBV13, CAEBV14, CAEBV19, CAEBV2, CAEBV20, CAEBV5, CAEBV6, IM12

pos: gammaEBV4-47220, gammaEBV4-47236, gammaEBV4-47240, gammaEBV4-47908, gammaEBV4-47955, gammaEBV4-48227, gammaEBV4-48251, gammaEBV4-48335, gammaEBV4-48519, gammaEBV4-48797, gammaEBV4-48983, gammaEBV4-49102, gammaEBV4-49719, gammaEBV4-50133, gammaEBV4-50242, gammaEBV4-51166, gammaEBV4-51500, gammaEBV4-51571

Row 38: gene = BPLF1

gene: BPLF1

sample: CAEBV1, CAEBV10, CAEBV11, CAEBV13, CAEBV14, CAEBV17, CAEBV18, CAEBV19, CAEBV2, CAEBV20, CAEBV21, CAEBV23, CAEBV5, CAEBV6, CAEBV7, CAEBV8, CAEBV9, IM12, IM14

pos: gammaEBV4-47908, gammaEBV4-47955, gammaEBV4-48227, gammaEBV4-48251, gammaEBV4-48335, gammaEBV4-48519, gammaEBV4-48797, gammaEBV4-48983, gammaEBV4-49102, gammaEBV4-49719, gammaEBV4-50133, gammaEBV4-50242, gammaEBV4-51166, gammaEBV4-51500, gammaEBV4-51571, gammaEBV4-52091, gammaEBV4-52205, gammaEBV4-52211, gammaEBV4-52227, gammaEBV4-52259, gammaEBV4-52372, gammaEBV4-52814, gammaEBV4-52837, gammaEBV4-52952, gammaEBV4-53724, gammaEBV4-53902, gammaEBV4-54149, gammaEBV4-54243, gammaEBV4-54255, gammaEBV4-54491, gammaEBV4-54802, gammaEBV4-54921, gammaEBV4-55000, gammaEBV4-55730, gammaEBV4-55780, gammaEBV4-55793, gammaEBV4-55811, gammaEBV4-55869, gammaEBV4-55887, gammaEBV4-55914, gammaEBV4-56237, gammaEBV4-56566, gammaEBV4-56866, gammaEBV4-57429, gammaEBV4-57632, gammaEBV4-57708, gammaEBV4-57740, gammaEBV4-57806, gammaEBV4-57842, gammaEBV4-57869, gammaEBV4-57870, gammaEBV4-57879, gammaEBV4-57880, gammaEBV4-57896, gammaEBV4-57897, gammaEBV4-58047, gammaEBV4-58301, gammaEBV4-58407, gammaEBV4-58634, gammaEBV4-58692, gammaEBV4-58785, gammaEBV4-58867, gammaEBV4-59115, gammaEBV4-59448, gammaEBV4-59672, gammaEBV4-59799, gammaEBV4-59923, gammaEBV4-59992, gammaEBV4-60095, gammaEBV4-60374, gammaEBV4-60398

Row 39: gene = EBNA-1

gene: EBNA-1

sample: CAEBV1, CAEBV10, CAEBV11, CAEBV13, CAEBV14, CAEBV15, CAEBV16, CAEBV17, CAEBV18, CAEBV19, CAEBV2, CAEBV20, CAEBV21, CAEBV23, CAEBV24, CAEBV3, CAEBV5, CAEBV6, CAEBV7, CAEBV8, CAEBV9, IM1, IM2, IM10, IM11, IM12, IM13, IM14, IM15, IM16, IM4, IM5, IM6, IM7, IM8

pos: gammaEBV4-48227, gammaEBV4-48251, gammaEBV4-48335, gammaEBV4-48519, gammaEBV4-48797, gammaEBV4-48983, gammaEBV4-49102, gammaEBV4-49719, gammaEBV4-50133, gammaEBV4-50242, gammaEBV4-51166, gammaEBV4-51500, gammaEBV4-51571, gammaEBV4-52091, gammaEBV4-52205, gammaEBV4-52211, gammaEBV4-52227, gammaEBV4-52259, gammaEBV4-52372, gammaEBV4-52814, gammaEBV4-52837, gammaEBV4-52952, gammaEBV4-53724, gammaEBV4-53902, gammaEBV4-54149, gammaEBV4-54243, gammaEBV4-54255, gammaEBV4-54491, gammaEBV4-54802, gammaEBV4-54921, gammaEBV4-55000, gammaEBV4-55730, gammaEBV4-55780, gammaEBV4-55793, gammaEBV4-55811, gammaEBV4-55869, gammaEBV4-55887, gammaEBV4-55914, gammaEBV4-56237, gammaEBV4-56566, gammaEBV4-56866, gammaEBV4-57429, gammaEBV4-57632, gammaEBV4-57708, gammaEBV4-57740, gammaEBV4-57806, gammaEBV4-57842, gammaEBV4-57869, gammaEBV4-57870, gammaEBV4-57879, gammaEBV4-57880, gammaEBV4-57896, gammaEBV4-57897, gammaEBV4-58047, gammaEBV4-58301, gammaEBV4-58407, gammaEBV4-58634, gammaEBV4-58692, gammaEBV4-58785, gammaEBV4-58867, gammaEBV4-59115, gammaEBV4-59448, gammaEBV4-59672, gammaEBV4-59799, gammaEBV4-59923, gammaEBV4-59992, gammaEBV4-60095, gammaEBV4-60374, gammaEBV4-60398, gammaEBV4-61987, gammaEBV4-62009, gammaEBV4-62182, gammaEBV4-62247, gammaEBV4-62391, gammaEBV4-62449, gammaEBV4-62499, gammaEBV4-62677, gammaEBV4-62690, gammaEBV4-62774, gammaEBV4-62778, gammaEBV4-62916, gammaEBV4-63356, gammaEBV4-63580, gammaEBV4-63692, gammaEBV4-64008, gammaEBV4-64199, gammaEBV4-64224, gammaEBV4-64324, gammaEBV4-64571, gammaEBV4-64795, gammaEBV4-64976, gammaEBV4-65127, gammaEBV4-65205, gammaEBV4-65433, gammaEBV4-65450, gammaEBV4-65685, gammaEBV4-65718, gammaEBV4-65785, gammaEBV4-65950, gammaEBV4-66061, gammaEBV4-66069, gammaEBV4-66363, gammaEBV4-66405, gammaEBV4-66612, gammaEBV4-66809, gammaEBV4-67279, gammaEBV4-67485, gammaEBV4-67505, gammaEBV4-67749, gammaEBV4-68057, gammaEBV4-68261, gammaEBV4-68279, gammaEBV4-68497, gammaEBV4-68583, gammaEBV4-68589, gammaEBV4-68612, gammaEBV4-68646, gammaEBV4-68657, gammaEBV4-68729, gammaEBV4-68748, gammaEBV4-69148, gammaEBV4-69192, gammaEBV4-69276, gammaEBV4-69611, gammaEBV4-69904, gammaEBV4-69950, gammaEBV4-70330, gammaEBV4-70538, gammaEBV4-71139, gammaEBV4-71229, gammaEBV4-71292, gammaEBV4-71344, gammaEBV4-71546, gammaEBV4-71618, gammaEBV4-71671, gammaEBV4-72053, gammaEBV4-72289, gammaEBV4-72560, gammaEBV4-72580, gammaEBV4-72690, gammaEBV4-72723, gammaEBV4-72979, gammaEBV4-73029, gammaEBV4-73305, gammaEBV4-73366, gammaEBV4-73378, gammaEBV4-73577, gammaEBV4-73713, gammaEBV4-73903, gammaEBV4-73989, gammaEBV4-74183, gammaEBV4-74250, gammaEBV4-74856, gammaEBV4-74882, gammaEBV4-75292, gammaEBV4-75357, gammaEBV4-75429, gammaEBV4-75555, gammaEBV4-75694, gammaEBV4-75699, gammaEBV4-76014, gammaEBV4-76449, gammaEBV4-76489, gammaEBV4-76525, gammaEBV4-76598, gammaEBV4-76943, gammaEBV4-77179, gammaEBV4-77304, gammaEBV4-77373, gammaEBV4-77401, gammaEBV4-77404, gammaEBV4-77419, gammaEBV4-77613, gammaEBV4-77647, gammaEBV4-77672, gammaEBV4-77826, gammaEBV4-78055, gammaEBV4-78268, gammaEBV4-78767, gammaEBV4-78969, gammaEBV4-79898, gammaEBV4-80010, gammaEBV4-80158, gammaEBV4-80180, gammaEBV4-80333, gammaEBV4-80380, gammaEBV4-80419, gammaEBV4-80444, gammaEBV4-81096, gammaEBV4-81378, gammaEBV4-81601, gammaEBV4-81671, gammaEBV4-81722, gammaEBV4-81794, gammaEBV4-81818, gammaEBV4-81950, gammaEBV4-81952, gammaEBV4-81957, gammaEBV4-81959, gammaEBV4-82027, gammaEBV4-82041, gammaEBV4-82056, gammaEBV4-82082, gammaEBV4-82089, gammaEBV4-82233, gammaEBV4-82234, gammaEBV4-82250, gammaEBV4-82253, gammaEBV4-82254, gammaEBV4-82259, gammaEBV4-82274, gammaEBV4-82347, gammaEBV4-82356, gammaEBV4-82358, gammaEBV4-82363, gammaEBV4-82366, gammaEBV4-82414, gammaEBV4-82418, gammaEBV4-82557, gammaEBV4-82685, gammaEBV4-82902, gammaEBV4-82911, gammaEBV4-83123, gammaEBV4-83632, gammaEBV4-84936, gammaEBV4-85018, gammaEBV4-85048, gammaEBV4-85131, gammaEBV4-85235, gammaEBV4-85666, gammaEBV4-87597, gammaEBV4-87818, gammaEBV4-87870, gammaEBV4-87967, gammaEBV4-88158, gammaEBV4-88365, gammaEBV4-88383, gammaEBV4-88385, gammaEBV4-88388, gammaEBV4-88390, gammaEBV4-88393, gammaEBV4-88397, gammaEBV4-88400, gammaEBV4-88401, gammaEBV4-88402, gammaEBV4-88405, gammaEBV4-88409, gammaEBV4-88410, gammaEBV4-88411, gammaEBV4-88412, gammaEBV4-88413, gammaEBV4-88414, gammaEBV4-88415, gammaEBV4-88416, gammaEBV4-88417, gammaEBV4-88419, gammaEBV4-88420, gammaEBV4-88421, gammaEBV4-88423, gammaEBV4-88424, gammaEBV4-88425, gammaEBV4-88426, gammaEBV4-88427, gammaEBV4-88429, gammaEBV4-88431, gammaEBV4-88432, gammaEBV4-88433, gammaEBV4-88435, gammaEBV4-88436, gammaEBV4-88437, gammaEBV4-88438, gammaEBV4-88439, gammaEBV4-88440, gammaEBV4-88442, gammaEBV4-88444, gammaEBV4-88445, gammaEBV4-88446, gammaEBV4-88447, gammaEBV4-88448, gammaEBV4-88449, gammaEBV4-88450, gammaEBV4-88451, gammaEBV4-88452, gammaEBV4-88453, gammaEBV4-88454, gammaEBV4-88455, gammaEBV4-88456, gammaEBV4-88457, gammaEBV4-88458, gammaEBV4-88459, gammaEBV4-88462, gammaEBV4-88463, gammaEBV4-88464, gammaEBV4-88465, gammaEBV4-88466, gammaEBV4-88467, gammaEBV4-88469, gammaEBV4-88470, gammaEBV4-88471, gammaEBV4-88472, gammaEBV4-88474, gammaEBV4-88476, gammaEBV4-88478, gammaEBV4-88483, gammaEBV4-88486, gammaEBV4-88495, gammaEBV4-88497, gammaEBV4-88498, gammaEBV4-88499, gammaEBV4-88500, gammaEBV4-88501, gammaEBV4-88502, gammaEBV4-88503, gammaEBV4-88504, gammaEBV4-88506, gammaEBV4-88507, gammaEBV4-88508, gammaEBV4-88511, gammaEBV4-88512, gammaEBV4-88514, gammaEBV4-88516, gammaEBV4-88527, gammaEBV4-88528, gammaEBV4-88529, gammaEBV4-88597, gammaEBV4-88834, gammaEBV4-89231, gammaEBV4-89456, gammaEBV4-90140, gammaEBV4-90282, gammaEBV4-90294, gammaEBV4-90298, gammaEBV4-90302, gammaEBV4-90304, gammaEBV4-90308, gammaEBV4-90311, gammaEBV4-90312, gammaEBV4-90313, gammaEBV4-90315, gammaEBV4-90317, gammaEBV4-90319, gammaEBV4-90320, gammaEBV4-90321, gammaEBV4-90322, gammaEBV4-90324, gammaEBV4-90332, gammaEBV4-90339, gammaEBV4-90340, gammaEBV4-90341, gammaEBV4-90342, gammaEBV4-90343, gammaEBV4-90347, gammaEBV4-90348, gammaEBV4-90349, gammaEBV4-90350, gammaEBV4-90351, gammaEBV4-90352, gammaEBV4-90353, gammaEBV4-90354, gammaEBV4-90355, gammaEBV4-90356, gammaEBV4-90357, gammaEBV4-90358, gammaEBV4-90359, gammaEBV4-90361, gammaEBV4-90370, gammaEBV4-90371, gammaEBV4-90372, gammaEBV4-90373, gammaEBV4-90376, gammaEBV4-90378, gammaEBV4-91150, gammaEBV4-91422, gammaEBV4-91471, gammaEBV4-91480, gammaEBV4-91705, gammaEBV4-93050, gammaEBV4-93159, gammaEBV4-93490, gammaEBV4-94085, gammaEBV4-94177, gammaEBV4-94212, gammaEBV4-94589, gammaEBV4-95186, gammaEBV4-95199, gammaEBV4-95240, gammaEBV4-95258, gammaEBV4-95261, gammaEBV4-95588, gammaEBV4-95590, gammaEBV4-95666, gammaEBV4-95906, gammaEBV4-95942, gammaEBV4-95961, gammaEBV4-95963, gammaEBV4-95969, gammaEBV4-95974, gammaEBV4-95983, gammaEBV4-95984, gammaEBV4-95999, gammaEBV4-96000, gammaEBV4-96003, gammaEBV4-96004, gammaEBV4-96006, gammaEBV4-96008, gammaEBV4-96010, gammaEBV4-96024, gammaEBV4-96029, gammaEBV4-96038, gammaEBV4-96055, gammaEBV4-96064, gammaEBV4-96065, gammaEBV4-96070, gammaEBV4-96073, gammaEBV4-96078, gammaEBV4-96096, gammaEBV4-96114, gammaEBV4-96117, gammaEBV4-96118, gammaEBV4-96123, gammaEBV4-96127, gammaEBV4-96128, gammaEBV4-96142, gammaEBV4-96181, gammaEBV4-96193, gammaEBV4-96197, gammaEBV4-96199, gammaEBV4-96203, gammaEBV4-96205, gammaEBV4-96207, gammaEBV4-96208, gammaEBV4-96240, gammaEBV4-96254, gammaEBV4-96259, gammaEBV4-96266, gammaEBV4-96276, gammaEBV4-96279, gammaEBV4-96280, gammaEBV4-96281, gammaEBV4-96289, gammaEBV4-96296, gammaEBV4-96298, gammaEBV4-96302, gammaEBV4-96306, gammaEBV4-96310, gammaEBV4-96313, gammaEBV4-96314, gammaEBV4-96315, gammaEBV4-96316, gammaEBV4-96319, gammaEBV4-96328, gammaEBV4-96333, gammaEBV4-96334, gammaEBV4-96337, gammaEBV4-96346, gammaEBV4-96349, gammaEBV4-96357, gammaEBV4-96364, gammaEBV4-96365, gammaEBV4-96367, gammaEBV4-96370, gammaEBV4-96373, gammaEBV4-96378, gammaEBV4-96379, gammaEBV4-96380, gammaEBV4-96382, gammaEBV4-96387, gammaEBV4-96388, gammaEBV4-96394, gammaEBV4-96396, gammaEBV4-96399, gammaEBV4-96400, gammaEBV4-96403, gammaEBV4-96405, gammaEBV4-96406, gammaEBV4-96410, gammaEBV4-96414, gammaEBV4-96415, gammaEBV4-96416, gammaEBV4-96417, gammaEBV4-96419, gammaEBV4-96422, gammaEBV4-96424, gammaEBV4-96425, gammaEBV4-96430, gammaEBV4-96436, gammaEBV4-96440, gammaEBV4-96442, gammaEBV4-96445, gammaEBV4-96446, gammaEBV4-96459, gammaEBV4-96462, gammaEBV4-96464, gammaEBV4-96466, gammaEBV4-96467, gammaEBV4-96468, gammaEBV4-96470, gammaEBV4-96472, gammaEBV4-96473, gammaEBV4-96481, gammaEBV4-96505, gammaEBV4-96521, gammaEBV4-96522, gammaEBV4-96542, gammaEBV4-96546, gammaEBV4-96554, gammaEBV4-96565, gammaEBV4-96566, gammaEBV4-96569, gammaEBV4-96575, gammaEBV4-96582, gammaEBV4-96586, gammaEBV4-96587, gammaEBV4-96588, gammaEBV4-96591, gammaEBV4-96592, gammaEBV4-96594, gammaEBV4-96595, gammaEBV4-96596, gammaEBV4-96598, gammaEBV4-96599, gammaEBV4-96601, gammaEBV4-96604, gammaEBV4-96607, gammaEBV4-96618, gammaEBV4-96622, gammaEBV4-96633, gammaEBV4-96634,

gammaEBV4-96636, gammaEBV4-96637, gammaEBV4-96638, gammaEBV4-96640, gammaEBV4-96653, gammaEBV4-96675, gammaEBV4-96677, gammaEBV4-96685, gammaEBV4-96701, gammaEBV4-96703, gammaEBV4-96714, gammaEBV4-96719, gammaEBV4-96722, gammaEBV4-96796, gammaEBV4-97303, gammaEBV4-97310, gammaEBV4-97457, gammaEBV4-97485, gammaEBV4-97584, gammaEBV4-97632, gammaEBV4-98321, gammaEBV4-98485, gammaEBV4-98607, gammaEBV4-98649, gammaEBV4-98857, gammaEBV4-99257, gammaEBV4-99261, gammaEBV4-99275, gammaEBV4-99399, gammaEBV4-99402, gammaEBV4-99405, gammaEBV4-99422, gammaEBV4-99423, gammaEBV4-99440, gammaEBV4-99441, gammaEBV4-99535

Row 40: gene = BOLF1

gene: BOLF1

sample: CAEBV11, CAEBV13, CAEBV14, CAEBV17, CAEBV18, CAEBV19, CAEBV2, CAEBV20, CAEBV21, CAEBV23, CAEBV5, CAEBV6, CAEBV8, CAEBV9, IM12, IM7

pos: gammaEBV4-57429, gammaEBV4-57632, gammaEBV4-57708, gammaEBV4-57740, gammaEBV4-57806, gammaEBV4-57842, gammaEBV4-57869, gammaEBV4-57870, gammaEBV4-57879, gammaEBV4-57880, gammaEBV4-57896, gammaEBV4-57897, gammaEBV4-58047, gammaEBV4-58301, gammaEBV4-58407, gammaEBV4-58634, gammaEBV4-58692, gammaEBV4-58785, gammaEBV4-58867, gammaEBV4-59115, gammaEBV4-59448, gammaEBV4-59672, gammaEBV4-59799, gammaEBV4-59923, gammaEBV4-59992, gammaEBV4-60095, gammaEBV4-60374, gammaEBV4-60398, gammaEBV4-61987, gammaEBV4-62009, gammaEBV4-62182, gammaEBV4-62247, gammaEBV4-62391, gammaEBV4-62449, gammaEBV4-62499, gammaEBV4-62677, gammaEBV4-62690, gammaEBV4-62774, gammaEBV4-62778, gammaEBV4-62916, gammaEBV4-63356, gammaEBV4-63580, gammaEBV4-63692, gammaEBV4-64008, gammaEBV4-64199, gammaEBV4-64224, gammaEBV4-64324, gammaEBV4-64571, gammaEBV4-64795

Row 41: gene = BORF2

gene: BORF2

sample: CAEBV1, CAEBV11, CAEBV13, CAEBV19, CAEBV2, CAEBV20, CAEBV21, CAEBV5, CAEBV6, CAEBV7, CAEBV8, CAEBV9, IM12, IM7

pos: gammaEBV4-62182, gammaEBV4-62247, gammaEBV4-62391, gammaEBV4-62449, gammaEBV4-62499, gammaEBV4-62677, gammaEBV4-62690, gammaEBV4-62774, gammaEBV4-62778, gammaEBV4-62916, gammaEBV4-63356, gammaEBV4-63580, gammaEBV4-63692, gammaEBV4-64008, gammaEBV4-64199, gammaEBV4-64224, gammaEBV4-64324, gammaEBV4-64571, gammaEBV4-64795, gammaEBV4-64976, gammaEBV4-65127, gammaEBV4-65205, gammaEBV4-65433, gammaEBV4-65450, gammaEBV4-65685, gammaEBV4-65718, gammaEBV4-65785, gammaEBV4-65950, gammaEBV4-66061, gammaEBV4-66069, gammaEBV4-66363, gammaEBV4-66405, gammaEBV4-66612, gammaEBV4-66809, gammaEBV4-67279, gammaEBV4-67485, gammaEBV4-67505, gammaEBV4-67749, gammaEBV4-68057, gammaEBV4-68261, gammaEBV4-68279, gammaEBV4-68497, gammaEBV4-68583, gammaEBV4-68589

Row 42: gene = BaRF1

gene: BaRF1

sample: CAEBV1, CAEBV11, CAEBV19, CAEBV2, CAEBV20, CAEBV5, CAEBV6, CAEBV7, CAEBV8, CAEBV9, IM12, IM16

pos: gammaEBV4-64795, gammaEBV4-64976, gammaEBV4-65127, gammaEBV4-65205, gammaEBV4-65433, gammaEBV4-65450, gammaEBV4-65685, gammaEBV4-65718, gammaEBV4-65785, gammaEBV4-65950, gammaEBV4-66061, gammaEBV4-66069, gammaEBV4-66363, gammaEBV4-66405, gammaEBV4-66612, gammaEBV4-66809, gammaEBV4-67279, gammaEBV4-67485, gammaEBV4-67505, gammaEBV4-67749, gammaEBV4-68057, gammaEBV4-68261, gammaEBV4-68279, gammaEBV4-68497, gammaEBV4-68583, gammaEBV4-68589, gammaEBV4-68612, gammaEBV4-68646, gammaEBV4-68657, gammaEBV4-68729, gammaEBV4-68748, gammaEBV4-69148, gammaEBV4-69192, gammaEBV4-69276

Row 43: gene = BSLF1

gene: BSLF1

sample: CAEBV1, CAEBV11, CAEBV13, CAEBV18, CAEBV19, CAEBV2, CAEBV20, CAEBV21, CAEBV5, CAEBV6, CAEBV9, IM11, IM5

pos: gammaEBV4-70330, gammaEBV4-70538, gammaEBV4-71139, gammaEBV4-71229, gammaEBV4-71292, gammaEBV4-71344, gammaEBV4-71546, gammaEBV4-71618, gammaEBV4-71671, gammaEBV4-72053, gammaEBV4-72289, gammaEBV4-72560, gammaEBV4-72580, gammaEBV4-72690, gammaEBV4-72723, gammaEBV4-72979, gammaEBV4-73029, gammaEBV4-73305, gammaEBV4-73366, gammaEBV4-73378, gammaEBV4-73577, gammaEBV4-73713, gammaEBV4-73903, gammaEBV4-73989, gammaEBV4-74183, gammaEBV4-74250, gammaEBV4-74856, gammaEBV4-74882, gammaEBV4-75292, gammaEBV4-75357, gammaEBV4-75429, gammaEBV4-75555, gammaEBV4-75694, gammaEBV4-75699, gammaEBV4-76014, gammaEBV4-76449, gammaEBV4-76489, gammaEBV4-76525

Row 44: gene = BLLF3

gene: BLLF3

sample: CAEBV1, CAEBV11, CAEBV13, CAEBV18, CAEBV19, CAEBV2, CAEBV20, CAEBV21, CAEBV5, CAEBV6, CAEBV8, CAEBV9, IM11, IM4, IM5

pos: gammaEBV4-73366, gammaEBV4-73378, gammaEBV4-73577, gammaEBV4-73713, gammaEBV4-73903, gammaEBV4-73989, gammaEBV4-74183, gammaEBV4-74250, gammaEBV4-74856, gammaEBV4-74882, gammaEBV4-75292, gammaEBV4-75357, gammaEBV4-75429, gammaEBV4-75555, gammaEBV4-75694, gammaEBV4-75699, gammaEBV4-76014, gammaEBV4-76449, gammaEBV4-76489, gammaEBV4-76525, gammaEBV4-76598, gammaEBV4-76943, gammaEBV4-77179, gammaEBV4-77304, gammaEBV4-77373, gammaEBV4-77401, gammaEBV4-77404, gammaEBV4-77419, gammaEBV4-77613, gammaEBV4-77647, gammaEBV4-77672, gammaEBV4-77826, gammaEBV4-78055

Row 45: gene = BLLF1

gene: BLLF1

sample: CAEBV1, CAEBV11, CAEBV13, CAEBV15, CAEBV18, CAEBV19, CAEBV2, CAEBV20, CAEBV21, CAEBV5, CAEBV6, CAEBV8, CAEBV9, IM15, IM4, IM5

pos: gammaEBV4-75292, gammaEBV4-75357, gammaEBV4-75429, gammaEBV4-75555, gammaEBV4-75694, gammaEBV4-75699, gammaEBV4-76014, gammaEBV4-76449, gammaEBV4-76489, gammaEBV4-76525, gammaEBV4-76598, gammaEBV4-76943, gammaEBV4-77179, gammaEBV4-77304, gammaEBV4-77373, gammaEBV4-77401, gammaEBV4-77404, gammaEBV4-77419, gammaEBV4-77613, gammaEBV4-77647, gammaEBV4-77672, gammaEBV4-77826, gammaEBV4-78055, gammaEBV4-78268, gammaEBV4-78767, gammaEBV4-78969, gammaEBV4-79898, gammaEBV4-80010, gammaEBV4-80158, gammaEBV4-80180, gammaEBV4-80333, gammaEBV4-80380, gammaEBV4-80419, gammaEBV4-80444, gammaEBV4-81096, gammaEBV4-81378, gammaEBV4-81601, gammaEBV4-81671, gammaEBV4-81722, gammaEBV4-81794, gammaEBV4-81818

Row 46: gene = BRLF1

gene: BRLF1

sample: CAEBV1, CAEBV10, CAEBV11, CAEBV13, CAEBV14, CAEBV16, CAEBV17, CAEBV18, CAEBV19, CAEBV2, CAEBV20, CAEBV21, CAEBV23, CAEBV24, CAEBV3, CAEBV5, CAEBV6, CAEBV7, CAEBV8, CAEBV9, IM1, IM2, IM10, IM12, IM14, IM15, IM5, IM6, IM7, IM8

pos: gammaEBV4-87870, gammaEBV4-87967, gammaEBV4-88158, gammaEBV4-88365, gammaEBV4-88383, gammaEBV4-88385, gammaEBV4-88388, gammaEBV4-88390, gammaEBV4-88393, gammaEBV4-88397, gammaEBV4-88400, gammaEBV4-88401, gammaEBV4-88402, gammaEBV4-88405, gammaEBV4-88409, gammaEBV4-88410, gammaEBV4-88411, gammaEBV4-88412, gammaEBV4-88413, gammaEBV4-88414, gammaEBV4-88415, gammaEBV4-88416, gammaEBV4-88417, gammaEBV4-88419, gammaEBV4-88420, gammaEBV4-88421, gammaEBV4-88423, gammaEBV4-88424, gammaEBV4-88425, gammaEBV4-88426, gammaEBV4-88427, gammaEBV4-88429, gammaEBV4-88431, gammaEBV4-88432, gammaEBV4-88433, gammaEBV4-88435, gammaEBV4-88436, gammaEBV4-88437, gammaEBV4-88438, gammaEBV4-88439, gammaEBV4-88440, gammaEBV4-88442, gammaEBV4-88444, gammaEBV4-88445, gammaEBV4-88446, gammaEBV4-88447, gammaEBV4-88448, gammaEBV4-88449, gammaEBV4-88450, gammaEBV4-88451, gammaEBV4-88452, gammaEBV4-88453, gammaEBV4-88454, gammaEBV4-88455, gammaEBV4-88456, gammaEBV4-88457, gammaEBV4-88458, gammaEBV4-88459, gammaEBV4-88462, gammaEBV4-88463, gammaEBV4-88464, gammaEBV4-88465, gammaEBV4-88466, gammaEBV4-88467, gammaEBV4-88469, gammaEBV4-88470, gammaEBV4-88471, gammaEBV4-88472, gammaEBV4-88474, gammaEBV4-88476, gammaEBV4-88478, gammaEBV4-88483, gammaEBV4-88486, gammaEBV4-88495, gammaEBV4-88497, gammaEBV4-88498, gammaEBV4-88499, gammaEBV4-88500, gammaEBV4-88501, gammaEBV4-88502, gammaEBV4-88503, gammaEBV4-88504, gammaEBV4-88506, gammaEBV4-88507, gammaEBV4-88508, gammaEBV4-88511, gammaEBV4-88512, gammaEBV4-88514, gammaEBV4-88516, gammaEBV4-88527, gammaEBV4-88528, gammaEBV4-88529, gammaEBV4-88597, gammaEBV4-88834, gammaEBV4-89231, gammaEBV4-89456, gammaEBV4-90140, gammaEBV4-90282, gammaEBV4-90294, gammaEBV4-90298, gammaEBV4-90302, gammaEBV4-90304, gammaEBV4-90308, gammaEBV4-90311, gammaEBV4-90312, gammaEBV4-90313, gammaEBV4-90315, gammaEBV4-90317, gammaEBV4-90319, gammaEBV4-90320, gammaEBV4-90321, gammaEBV4-90322, gammaEBV4-90324, gammaEBV4-90332, gammaEBV4-90339, gammaEBV4-90340, gammaEBV4-90341, gammaEBV4-90342, gammaEBV4-90343, gammaEBV4-90347, gammaEBV4-90348, gammaEBV4-90349, gammaEBV4-90350, gammaEBV4-90351, gammaEBV4-90352, gammaEBV4-90353, gammaEBV4-90354, gammaEBV4-90355, gammaEBV4-90356, gammaEBV4-90357, gammaEBV4-90358, gammaEBV4-90359, gammaEBV4-90361, gammaEBV4-90370, gammaEBV4-90371, gammaEBV4-90372, gammaEBV4-90373, gammaEBV4-90376, gammaEBV4-90378, gammaEBV4-91150, gammaEBV4-91422, gammaEBV4-91471, gammaEBV4-91480, gammaEBV4-91705, gammaEBV4-93050, gammaEBV4-93159, gammaEBV4-93490, gammaEBV4-94085, gammaEBV4-94177, gammaEBV4-94212, gammaEBV4-94589, gammaEBV4-95186, gammaEBV4-95199, gammaEBV4-95240, gammaEBV4-95258, gammaEBV4-95261, gammaEBV4-95588, gammaEBV4-95590, gammaEBV4-95666

Row 47: gene = BZLF1

gene: BZLF1

sample: CAEBV1, CAEBV10, CAEBV11, CAEBV13, CAEBV16, CAEBV17, CAEBV18, CAEBV19, CAEBV2, CAEBV20, CAEBV21, CAEBV23, CAEBV24, CAEBV3, CAEBV5, CAEBV6, CAEBV7, CAEBV8, CAEBV9, IM1, IM2, IM10, IM12, IM14, IM15, IM5, IM6, IM7, IM8

pos: gammaEBV4-87870, gammaEBV4-87967, gammaEBV4-88158, gammaEBV4-88365, gammaEBV4-88383, gammaEBV4-88385, gammaEBV4-88388, gammaEBV4-88390, gammaEBV4-88393, gammaEBV4-88397, gammaEBV4-88400, gammaEBV4-88401, gammaEBV4-88402, gammaEBV4-88405, gammaEBV4-88409, gammaEBV4-88410, gammaEBV4-88411, gammaEBV4-88412, gammaEBV4-88413, gammaEBV4-88414, gammaEBV4-88415, gammaEBV4-88416, gammaEBV4-88417, gammaEBV4-88419, gammaEBV4-88420, gammaEBV4-88421, gammaEBV4-88423, gammaEBV4-88424, gammaEBV4-88425, gammaEBV4-88426, gammaEBV4-88427, gammaEBV4-88429, gammaEBV4-88431, gammaEBV4-88432, gammaEBV4-88433, gammaEBV4-88435, gammaEBV4-88436, gammaEBV4-88437, gammaEBV4-88438, gammaEBV4-88439, gammaEBV4-88440, gammaEBV4-88442, gammaEBV4-88444, gammaEBV4-88445, gammaEBV4-88446, gammaEBV4-88447, gammaEBV4-88448, gammaEBV4-88449, gammaEBV4-88450, gammaEBV4-88451, gammaEBV4-88452, gammaEBV4-88453, gammaEBV4-88454, gammaEBV4-88455, gammaEBV4-88456, gammaEBV4-88457, gammaEBV4-88458, gammaEBV4-88459, gammaEBV4-88462, gammaEBV4-88463, gammaEBV4-88464, gammaEBV4-88465, gammaEBV4-88466, gammaEBV4-88467, gammaEBV4-88469, gammaEBV4-88470, gammaEBV4-88471, gammaEBV4-88472, gammaEBV4-88474, gammaEBV4-88476, gammaEBV4-88478, gammaEBV4-88483, gammaEBV4-88486, gammaEBV4-88495, gammaEBV4-88497, gammaEBV4-88498, gammaEBV4-88499, gammaEBV4-88500, gammaEBV4-88501, gammaEBV4-88502, gammaEBV4-88503, gammaEBV4-88504, gammaEBV4-88506, gammaEBV4-88507, gammaEBV4-88508, gammaEBV4-88511, gammaEBV4-88512, gammaEBV4-88514, gammaEBV4-88516, gammaEBV4-88527, gammaEBV4-88528, gammaEBV4-88529, gammaEBV4-88597, gammaEBV4-88834, gammaEBV4-89231, gammaEBV4-89456, gammaEBV4-90140, gammaEBV4-90282, gammaEBV4-90294, gammaEBV4-90298, gammaEBV4-90302, gammaEBV4-90304, gammaEBV4-90308, gammaEBV4-90311, gammaEBV4-90312, gammaEBV4-90313, gammaEBV4-90315, gammaEBV4-90317, gammaEBV4-90319, gammaEBV4-90320, gammaEBV4-90321, gammaEBV4-90322, gammaEBV4-90324, gammaEBV4-90332, gammaEBV4-90339, gammaEBV4-90340, gammaEBV4-90341, gammaEBV4-90342, gammaEBV4-90343, gammaEBV4-90347, gammaEBV4-90348, gammaEBV4-90349, gammaEBV4-90350, gammaEBV4-90351, gammaEBV4-90352, gammaEBV4-90353, gammaEBV4-90354, gammaEBV4-90355, gammaEBV4-90356, gammaEBV4-90357, gammaEBV4-90358, gammaEBV4-90359, gammaEBV4-90361, gammaEBV4-90370, gammaEBV4-90371, gammaEBV4-90372, gammaEBV4-90373, gammaEBV4-90376, gammaEBV4-90378, gammaEBV4-91150, gammaEBV4-91422, gammaEBV4-91471, gammaEBV4-91480, gammaEBV4-91705

Row 48: gene = BRRF1

gene: BRRF1

sample: CAEBV11, CAEBV13, CAEBV14, CAEBV16, CAEBV19, CAEBV2, CAEBV20, CAEBV3, CAEBV5, CAEBV6, CAEBV8, CAEBV9

pos: gammaEBV4-91150, gammaEBV4-91422, gammaEBV4-91471, gammaEBV4-91480, gammaEBV4-91705, gammaEBV4-93050, gammaEBV4-93159, gammaEBV4-93490, gammaEBV4-94085, gammaEBV4-94177, gammaEBV4-94212, gammaEBV4-94589, gammaEBV4-95186, gammaEBV4-95199, gammaEBV4-95240, gammaEBV4-95258, gammaEBV4-95261, gammaEBV4-95588, gammaEBV4-95590, gammaEBV4-95666

Sheet: Sheet3

gene	CAEBV	IM	Mean methylation difference	P value
BOLF1	0.96349068323	0.780008928571	0.183481754658	4.92515e-14
A73	0.964149275362	0.821766666667	0.142382608696	1.11132e-11
BSLF1	0.85374360119	0.526288865546	0.327454735644	1.44318e-11
BdRF1	0.97335	0.873375	0.099975	1.86792e-10
LF2	0.967869565217	0.784882352941	0.182987212276	1.93226e-10
BALF1	0.895416666667	0.537470588235	0.357946078431	3.83252e-10
BNRF1	0.947218055556	0.817769607843	0.129448447712	2.07892e-09
BALF2	0.961985507246	0.826215686275	0.135769820972	3.82999e-08
BALF5	0.901869565217	0.562466666667	0.339402898551	1.06176e-07
BVLF1	0.965456521739	0.81040625	0.155050271739	2.95595e-07
BPLF1	0.73391788998	0.610934186728	0.122983703252	6.69658e-06
BBLF2/BBLF3	0.935739130435	0.803208333333	0.132530797101	4.50107e-05
BFRF2	0.942956521739	0.808882352941	0.134074168798	4.7129e-05
LF1	0.956673913043	0.806823529412	0.149850383632	5.66205e-05
BTRF1	0.952136363636	0.875125	0.0770113636364	0.000103705257081
BBRF2	0.984541666667	0.9470625	0.0374791666667	0.000106701333349
BILF1	0.981217391304	0.937642857143	0.0435745341615	0.00122775435325
BVRF2	0.979714285714	0.91725	0.0624642857143	0.00228092129305
BBLF4	0.844489130435	0.749359375	0.0951297554348	0.0046614158593
BRRF1	0.324159090909	0.140961538462	0.183197552448	0.0486248915154

Table S9. Clinical characteristics and examination parameters of patients with CAEBV before and after treatment

Patients	Treatment	EBV loads in whole blood (copies/mL)		EBV loads in plasma (copies/mL)		Outcome after treatment*
		Before	After	Before	After	
**CAEBV 1**	allo-HSCT	3.49×10^{6}	1.97×10^{3}	3×10^{3}	<500	clinical complete remission for 3 months
**CAEBV 3**	allo-HSCT	3.76×10^{6}	5.31×10^{2}	2.23×10^{4}	<500	clinical complete remission for 4 months
**CAEBV 12**	allo-HSCT	9.08×10^{5}	1.07×10^{3}	5.02×10^{4}	<500	clinical complete remission for 1 year
**CAEBV 19**	allo-HSCT	5.37×10^{6}	2.69×10^{3}	<500	<500	clinical complete remission for 6 months
**CAEBV 10**	Cytotoxic chemotherapy	1.65×10^{6}	5.39×10^{5}	1.3×10^{3}	1.24×10^{3}	clinical partial remission
**CAEBV 11**	Cytotoxic chemotherapy	6.59×10^{6}	4.41×10^{6}	4.15×10^{3}	5.83×10^{2}	stable disease
**CAEBV 16**	Cytotoxic chemotherapy	6.52×10^{5}	1.17×10^{4}	1.72×10^{3}	<500	clinical partial remission
**CAEBV 20**	Cytotoxic chemotherapy	1.4×10^{7}	3.9×10^{6}	6.24×10^{4}	<500	stable disease

CAEBV, chronic active Epstein–Barr virus infection; allo-HSCT, allogeneic hematopoietic stem cell transplantation.

*Outcome at the last follow-up date.
