# Supplementary material for: Integrated EBV and host DNA methylation profiling identifies distinct candidate EBV–host junction patterns in chronic active EBV infection
Source: Microbiol Spectr. 2026 Sep 25;14(11):e01881-26. doi: 10.1128/spectrum.01881-26 (PMC13729105; doi:10.1128/spectrum.01881-26)
Supplement: Supplementary file 3 [file spectrum.01881-26-s0002.docx]

**Supplementary figure legends**

Fig. S1. DNA methylation patterns of the EBV genome.

(A) Representative DNA methylation profiles of EBV latent promoters (Wp and LMPp), latent gene regions (*LMP-2A* and *LMP-2B*), and lytic gene regions (*BLLF1*, *BcRF1*, and *BcLF1*). Each vertical bar represents an individual CpG site plotted according to genomic coordinates. The y-axis indicates methylation density (0–1.00). (B) Principal component analysis (PCA) of EBV DNA methylation profiles in CAEBV patients with and without hemophagocytic lymphohistiocytosis (HLH), showing overall clustering patterns. (C) PCA of host differentially methylated regions (DMRs) in CAEBV patients with and without HLH.

Fig. S2. Functional enrichment analysis of differentially methylated genes.

(A) Gene Ontology (GO) enrichment barplot of differentially methylated site–related genes. The x-axis represents −log10(*P* value), and the y-axis shows GO biological process categories. (B) KEGG pathway classification of differentially methylated site–related genes. The x-axis represents the number of genes, and the y-axis shows KEGG secondary pathway categories. Colors denote KEGG primary pathway classifications. (C) Bubble plot of KEGG pathway enrichment analysis. The x-axis indicates the rich factor, bubble size represents gene number, and color gradient reflects −log10(*Q* value). (D) GO enrichment analysis of promoter-associated differentially methylated genes. The x-axis represents −log10(*P* value), and the y-axis indicates enriched GO terms. (E) KEGG pathway enrichment analysis of promoter-associated differentially methylated genes. The x-axis represents −log10(*P* value), and the y-axis indicates enriched KEGG pathways. GO, Gene Ontology; KEGG, Kyoto Encyclopedia of Genes and Genomes.

Fig. S3. Distribution and functional annotation of candidate EBV–host junctions.

(A) Distribution of candidate EBV–host junctions across the EBV genome in CAEBV samples. (B) Distribution of candidate EBV–host junctions across the EBV genome in IM samples. (C) Circos plot showing recurrently targeted genes (RTGs) in the host genome of IM samples. Each bar denotes a candidate junction mapped onto the 24 human chromosomes. Red bars represent the frequency of candidate junction detection across samples. Genomic loci with relatively high recurrence are labeled. (D) GO enrichment analysis of RTGs identified in IM samples. The x-axis represents −log10(*P* value).

**Supplementary tables**

Table S1. Examination parameters of patients with CAEBV, IM HC and uninfected groups

CAEBV, chronic active Epstein–Barr virus infection; IM, infectious mononucleosis; HC, healthy carriers with EBV; uninfected, EBV uninfected children; M, male; F, Female; VCA, virus capsid antigen; EBNA, EBV nuclear antigen; “–”, not tested; “<500”, below the limit of quantification or detection of the clinical assay.

Table S2. Number of DMRs detected in CAEBV versus IM, HC and uninfected groups

DMRs, differentially methylated regions; CAEBV, chronic active Epstein–Barr virus infection; IM, infectious mononucleosis; HC, healthy carrier with EBV; uninfected, EBV-uninfected child.

Table S3. Genes with significant changes in expression levels between CAEBV and IM

Hypermethylated and downregulated genes are labeled in green; hypomethylated and upregulated genes are labeled in red; hypermethylated and upregulated and hypomethylated and downregulated genes are labeled in blue.

Table S4. Candidate viral-side EBV–host junction coordinates in CAEBV and IM samples

Table S5. Candidate host-side EBV–host junction coordinates in CAEBV and IM samples

Table S6. Host genes recurrently overlapped by candidate EBV–host junctions in CAEBV and IM samples

Table S7. Genomic overlap between host differentially methylated genes and candidate EBV–host junctions

Table S8. Genomic overlap between EBV methylation features and viral-side candidate EBV–host junctions

Table S9. Clinical characteristics and examination parameters of patients with CAEBV before and after treatment
