# Supplementary material for: Integrated EBV and host DNA methylation profiling identifies distinct candidate EBV–host junction patterns in chronic active EBV infection
Source: Microbiol Spectr. 2026 Sep 25;14(11):e01881-26. doi: 10.1128/spectrum.01881-26 (PMC13729105; doi:10.1128/spectrum.01881-26)
Supplement: Supplementary file 4 [file spectrum.01881-26-s0003.docx]

**Supplementary Methods**

**EBV serology and DNA load**

The EBV serology profile was measured using the commercial EBV test kit from EUROIMMUN Corporation (Lubeck). EBV DNA load was measured using a PCR-based commercial kit (DAAN Gene). The standard protocols from the manufacturers were followed for these assays.

**DNA methylation of EBV genome profiling by targeted bisulfite sequencing (TBS) and data processing**

Genomic DNA of PBMC samples was extracted using Magnetic Universal Genomic DNA Kit (TIANGEN), and 1 μg of genomic DNA was converted using the ZYMO EZ DNA Methylation-Gold Kit (Zymo Research).^1^ Barcoded libraries from all samples were sequenced using the Illumina platform.^2^ The samples used for TBS included 24 patients with CAEBV (CAEBV 1–24), 17 patients with IM (IM 1–17), and 9 HCs (HC 1–9). The EBV DNA loads reported in Supplemental Table 1 were obtained from clinical testing of plasma or whole blood rather than from PBMC quantification. For each TBS sample, 1 μg of total genomic DNA extracted from PBMCs was used as the starting input. At the current sequencing depth, the HC samples did not yield sufficient EBV-derived reads for reliable viral methylation analysis.

Sequencing adapters and low-quality sequencing data were removed by Trimmomatic (version 0.36). The methylation level of the CpG site was calculated using the Python program for calculating methylation with BSMAP software. The calculation principle is methylation densities = C-reads/(C-reads + T-reads)$\times$100%, where C-reads is the number of methylation-supporting reads covering the site (the site is measured as C reads) and T-reads is the number of reads that do not support methylation covering the site (reads with a T at the site). Finally, according to the methylation densities calculated above, R language was used for statistical and visual display, and the two-tailed *t* test was used to analyze the differences between groups. A difference was considered significant when the *P* value$<$0.05 and the absolute value of the difference in mean methylation between groups was greater than 0.1. The gene-region heatmap was generated based on probe coverage and the distribution of valid reads. Both LMP-2B and EBNA3A were divided into three independently covered fragments, labeled as LMP-2B (1/2/3) and EBNA3A (1/2/3), respectively; the numbers indicate different sequenced fragments.

**DNA methylation profiling by reduced representation bisulfite sequencing (RRBS) and data processing**

Genomic DNA of PBMC samples from 24 CAEBV (CAEBV 1–24), 17 IM (IM 1–17), 9 HC (HC 1–9) and 9 EBV-uninfected children (uninfected 1–9) was extracted using DNeasy Blood and Tissue Kit (Qiagen), and 200 ng–1 μg of DNA was subjected to RRBS library preparation for genome-wide DNA methylation profiling. The FastQC tool was used to perform basic statistics on the quality of the raw reads. Sequencing adapters and low-quality sequencing data were removed by Trimmomatic (version 0.36).^3^

BSMAP software was used to map the bisulfite sequence to the reference genome with parameters.^4^ The statistical information of the alignment was collected; only the unique mapped reads were retained for the following analysis, and only methylated cytosines with a sequence depth coverage of at least 5 were used. If the base on the alignment is C, methylation occurs; if the base on the alignment is T, no methylation occurs. The methylation levels of individual cytosines were calculated as the ratio of the sequenced depth of the ascertained methylated CpG cytosines to the total sequenced depth of individual CpG cytosines [ML = mC/(mC + umC)], where ML is the methylation level and mC and umC represent the number of reads supporting methylation C and the number of reads supporting unmethylated C, respectively.

Differential methylation regions (DMRs) were identified using Metilene (ver. 0.2–7). A DMR was required to contain at least five cytosine sites < 200 bp in distance between adjacent cytosine sites. CG-DMR candidate regions were required to have average methylation level differences of$>$0.1 between the corresponding populations.^5^ Finally, regions with a 2D KS-test *P* value$<$0.05 and BH (Benjamini & Hochberg) corrected *P* value$<$0.05 were determined as final DMRs. Promoter methylation was calculated as the averaged DNA methylation values based on GENCODE promoter regions (i.e., 1 kb upstream to 500 bp downstream of the annotated TSSs).

**Gene expression analysis through RNA sequencing (RNA-Seq)**

Global gene expression profiling of RNA extracted from 8 CAEBV (CAEBV 25–32) and 7 IM (IM 18–24) samples was performed using SMART-Seq HT Kit. Paired-end libraries were synthesized by using TruSeq RNA Sample Preparation Kit (Illumina) following TruSeq RNA Sample Preparation Guide. Purified libraries were quantified by Qubit 2.0 Fluorometer (Life Technologies). Library construction and sequencing were performed at Shanghai Biotechnology Corporation. HISAT2 was used (version: 2.0.4) to map the cleaned reads to the human hg38 reference genome with two mismatches.^6^ The *P* value significance threshold in multiple tests was set by the false discovery rate (FDR).^7^ The fold changes (FC) were also estimated according to the FPKM in each sample. The differentially expressed genes were selected using the following filter criteria: *P* value < 0.05 and |log2FC| >1. RNA-seq and RRBS were performed using independent patient cohorts; therefore, the cross-omics comparison evaluated only the directions of group-level differences in CAEBV relative to IM.

**Cluster analysis**

Unsupervised hierarchical clustering analysis was performed using R v3.6.3. with the ComplexHeatmap package. The Euclidean distance was used as the distance function both for samples and genes.

**DNA liquid phase hybridization capture sequencing**

Genomic DNA was ultrasonically interrupted to a 180–220 bp main band range. The library was constructed using QuarPrep Ultra DNA Library Kit (Dynasty Gene). DNA was captured with a predesigned EBV probe. Qualified libraries were sequenced on the Illumina Novaseq 6000 sequencing platform, and the sequencing strategy was 150 bp paired-end sequencing. Adaptor sequences and low-quality reads were removed. High-quality reads were aligned to the human (hg19) and EBV (NC_007605.1) genomes using Burrows‒Wheeler Aligner (BWA).^8^ Alignments were converted from a sequence alignment map format to sorted and indexed binary alignment map (BAM) files.^9^

Candidate EBV–host junction-supporting signals, in which one read mapped to the EBV genome and the paired read mapped to the human genome, were extracted. Signals supported by ≥1 read pair were retained as candidate events for exploratory analysis. The HumCFS database was used to annotate candidate junction coordinates within fragile regions.^10^ The UCSC Table Browser was used for genomic annotation.^11^ Representative junction sequences were extracted from capture-derived split reads and aligned to the human and EBV reference genomes for visualization. No junction-specific PCR or Sanger sequencing was performed in this study.

**Data sharing and ethical restrictions for sequencing datasets**

The sequencing libraries were generated from PBMC-derived DNA or RNA obtained from pediatric participants. Although the samples and analytical datasets were de-identified, the raw FASTQ files contain individual-level human genomic or transcriptomic sequence information. The original ethics approvals and informed-consent procedures did not authorize unrestricted public deposition of such data; accordingly, the raw sequencing reads were not deposited in an unrestricted open-access repository.

To support transparency and reproducibility within these ethical constraints, the de-identified processed and derived data underlying the major analyses are provided in the article and accompanying supplementary tables at the level permitted by the ethics approvals. These materials include results from EBV methylation, host DNA methylation, differential gene-expression, and candidate EBV–host junction analyses, together with the software, reference genomes, quality-control procedures, analytical parameters, and filtering criteria described above.

**References**

1.	Gao F, Liang H, Lu H, et al. Global analysis of DNA methylation in hepatocellular carcinoma by a liquid hybridization capture-based bisulfite sequencing approach. *Clin Epigenetics*. 2015;7:86.

2.	Pan X, Gong D, Nguyen DN, et al. Early microbial colonization affects DNA methylation of genes related to intestinal immunity and metabolism in preterm pigs. *DNA Res*. 2018.

3.	Bolger AM, Lohse M, Usadel B. Trimmomatic: a flexible trimmer for Illumina sequence data. *Bioinformatics*. 2014;30(15):2114-2120.

4.	Xi Y, Li W. BSMAP: whole genome bisulfite sequence MAPping program. *BMC Bioinformatics*. 2009;10:232.

5.	Juhling F, Kretzmer H, Bernhart SH, Otto C, Stadler PF, Hoffmann S. metilene: fast and sensitive calling of differentially methylated regions from bisulfite sequencing data. *Genome Res*. 2016;26(2):256-262.

6.	Kim D, Langmead B, Salzberg SL. HISAT: a fast spliced aligner with low memory requirements. *Nat Methods*. 2015;12(4):357-360.

7.	Korthauer K, Kimes PK, Duvallet C, et al. A practical guide to methods controlling false discoveries in computational biology. *Genome Biol*. 2019;20(1):118.

8.	Li H, Durbin R. Fast and accurate short read alignment with Burrows-Wheeler transform. *Bioinformatics*. 2009;25(14):1754-1760.

9.	Li H, Handsaker B, Wysoker A, et al. The Sequence Alignment/Map format and SAMtools. *Bioinformatics*. 2009;25(16):2078-2079.

10.	Kumar R, Nagpal G, Kumar V, Usmani SS, Agrawal P, Raghava GPS. HumCFS: a database of fragile sites in human chromosomes. *BMC Genomics*. 2019;19(Suppl 9):985.

11.	Karolchik D, Hinrichs AS, Furey TS, et al. The UCSC Table Browser data retrieval tool. *Nucleic Acids Res*. 2004;32(Database issue):D493-496.
